# Fluorescent Carbazole‐Derived Aza[5]Helicenes: Synthesis, Functionalization, and Characterization

**DOI:** 10.1002/chem.202501081

**Published:** 2025-04-17

**Authors:** Inka Marten, Melina E. A. Dilanas, Joachim Podlech

**Affiliations:** ^1^ Institute of Organic Chemistry Karlsruhe Institute of Technology (KIT) Kaiserstraße 12 Karlsruhe Germany; ^2^ Institute of Inorganic Chemistry Karlsruhe Institute of Technology (KIT) Kaiserstraße 12 Karlsruhe Germany

**Keywords:** aggregation‐induced emission, azaarenes, cross coupling, fluorescence, helicenes

## Abstract

5,8‐Dihydroindolo[2,3‐*c*]carbazole (ICz), 9*H*‐cinnolino[3,4‐*c*]carbazole (CnCz), and variously alkyl‐, alkenyl‐, and aryl‐substituted indolo[2,3‐*k*]‐ and ‐[3,2‐*a*]phenanthridines (IPs) were synthesized using an *ortho* fusion strategy with Suzuki cross couplings, intramolecular nitrene insertions, diazo couplings, and Morgan–Walls cyclizations as key reactions. The IPs were additionally transformed into organoboranes and helicene conjugates with tetraphenylethylene derivatives. The compounds fluoresce with large Stokes shifts, exhibit strong acidochromism, and show a good to excellent aggregation‐induced emission. Their helical structure was elucidated by x‐ray crystallographic analysis and by quantum chemical calculations. HOMO–LUMO gaps of 3.96−4.06 eV and S_1_‐T_1_ gaps were calculated, with CnCz showing a small singlet‐triplet inversion. Relative p*K*_a_ values of 6.65−9.55 were estimated for the different types of azahelicenes.

## Graphical abstract

Variously substituted indolophenanthridines, indolocarbazoles, and cinnolinocarbazoles synthesized by ortho fusion from teraryl precursors show significant Stokes shifts, especially for the protonated states of indolophenanthridines and cinnolinocarbazole. These compounds combine helical and axial chirality and show prominent aggregation‐induced emission, which is considerably enhanced in conjugates with tetraphenylethylene.

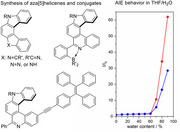

## 1 Introduction

**Scheme 1:**
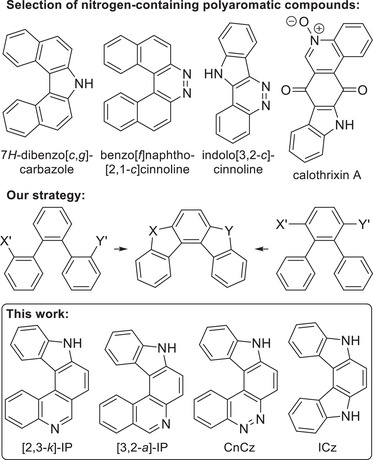
Top: selection of known nitrogen‐containing polyaromatic compounds; middle: *ortho*, *ortho*’ fusion (X, Y, X’, Y’ are suitable substituents and linkages); bottom: basic scaffolds of the helicenes synthesized by this approach.

The first two helicenes ever synthesized (by Meisenheimer and Witte in 1903) were actually azahelicenes: 7*H*‐dibenzo[*c*,*g*]‐carbazole and benzo[*f*]naphtho[2,1‐*c*]cinnoline (Scheme 1, top).^[^ ^1^ ^]^ Since then, a plethora of (aza‐)helicenes have been reported. According to the IUPAC, helicenes are “*ortho*‐fused polycyclic aromatic or heteroaromatic compounds in which all rings (minimum five) are angularly arranged so as to give helically shaped molecules, which are thus chiral.”^[^ ^2^ ^]^ Due to their unique structure, they often show beneficial (chir‐)optical properties compared to similar but planar polycyclic aromatic compounds, such as circular dichroism (CD) and circularly polarized luminescence (CPL).^[^ ^3^ ^]^ Only recently, the Tien‐Lin Wu group presented aza[6]helicenes that exhibit ultra‐long room‐temperature phosphorescence lifetimes.^[^ ^4^ ^]^ Furthermore, azahelicenes are (inter alia) studied and used as chiral organocatalysts,^[^ ^5^, ^6^ ^]^ ligands in metal complexes,^[^ ^7^ ^]^ fluorescent dyes for solar cells,^[^ ^8^ ^]^ circularly polarized organic light‐emitting diodes (CPOL‐EDs),^[^ ^3^, ^9^ ^]^ photoswitches,^[^ ^10^ ^]^ and as biologically active compounds, which interact with DNA^[^ ^11^ ^]^ or show anti‐cancer effects.^[^ ^12^ ^]^ These reports as well as the pharmacologic activities for planar polycyclic aromatic compounds such as calothrixin A and B^[^ ^13^, ^14^, ^15^ ^]^ and indolo[3,2‐*c*]cinnolines^[^ ^16^ ^]^ (Scheme 1, top) drew our attention to structurally related nitrogen‐containing helicenes. Among them, indolocarbazoles (ICz) and indolophenanthridines (IPs) already found interest due to their effect against infectious diseases^[^ ^17^ ^]^ and/or their possible application in (opto‐)electronic devices.^[^ ^18^, ^19^, ^20^, ^21^ ^]^

In the last years, we have developed an *ortho* fusion method for the synthesis of various aza‐, thia‐, and carbohelicenes from *ortho*,*ortho*’‐disubstituted terphenyls or teraryls (Scheme 1, middle).^[^ ^22^, ^23^, ^24^, ^25^, ^26^ ^]^ We recently reported on the application of this synthetic route to a small selection of indolo[2,3‐*k*]phenanthridines ([2,3‐*k*]‐IPs) and indolo[3,2‐*a*]phenanthridines ([3,2‐*a*]‐IPs).^[^ ^27^ ^]^ In the present full paper we demonstrate that the developed route is generally suitable for the synthesis of a variety of derivatives with alkyl, alkenyl, aryl, and heteroaryl electron‐deficient and electron‐donating substituents. It does not require protection groups, enables late‐stage functionalization, allows further transformations, e.g., to helicene conjugates, and furthermore gives quick access to the known 5,8‐dihydroindolo[2,3‐*c*]carbazole (ICz)^[^ ^18^, ^21^, ^28^ ^]^ and the novel 9*H*‐cinnolino[3,4‐*c*]carbazole (CnCz) (see Scheme 1, bottom).

## 2 Results and Discussion

### 2.1 Syntheses

In follow‐up to the recently published synthesis of a small series of helicene derivatives **3** and **9** (R = Me, *t*Bu, (*E*)‐MeCH═CH, Ph, and 3‐pyridinyl),^[^ ^27^ ^]^ we were interested to further investigate the influence of different substituents on optical properties and to provide a basis for the development of functional molecules such as Lewis acid/base complexes or helicene conjugates. Therefore, we introduced larger conjugated systems [R = (*E*,*E*)‐Me(CH═CH)_2_], electron‐withdrawing and ‐donating groups (R = CF_3_, MeO‐C_6_H_4_, F_3_C‐C_6_H_4_) as well as other (hetero‐)aromatic substituents (R = 2‐pyridyl, 1‐naphthalenyl) and functional groups allowing for further transformations (R = CH_2_Cl, CH_2_N_3_).

**Scheme 2:**
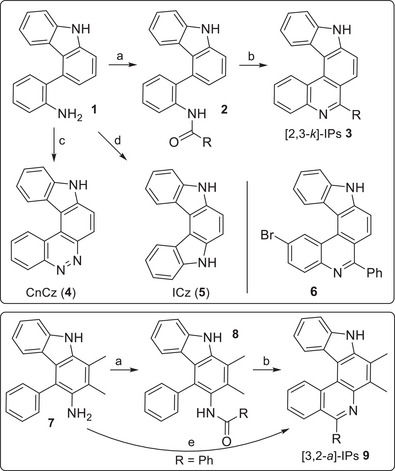
Synthesis of [2,3‐*k*]‐IPs **3** and **6**, CnCz (**4**), and ICz (**5**) (top), and of [3,2‐*a*]‐IPs **9** (bottom). Conditions: a) see Table 1; b) POCl_3_, PhNO_2_, 150°C, 3–65.5 h or Ph_3_PO, Tf_2_O, 0°C, 15 min, then amide, 0°C to rt, 3 h, yields are given in Table 1; c) NaNO_2_, HCl_aq_, 0°C to rt, 2 h (quant.); d) *t*BuONO, TMSN_3_, MeCN, 0°C to rt, 3 h, then *o*‐xylene, 190°C, 15 h (71%); e) PhCHO, cat. *p*‐TosOH·H_2_O, 1,2‐dichloroethane, 85°C, 17.5 h (32%).

Amino‐substituted biaryl precursors **1** and **7** were synthesized as described previously.^[^ ^27^ ^]^ Different amide formation and cyclization methods were required in order to obtain differently substituted IPs **3** and **9** (Scheme 2). Most amide formations turned out to be successful by reacting amines **1** and **7** with either the respective acyl chloride^[^ ^29^, ^30^ ^]^ or the carboxylic acid in presence of propanephosphonic acid anhydride (PPAA) as activating and dehydrating reagent.^[^ ^31^ ^]^ Coupling with pyridinecarboxylic acids required special conditions: triphenyl phosphite and pyridine as coupling agent for the synthesis of 2‐pyridinecarboxamide **2l**,^[^ ^32^ ^]^ and benzotriazol‐1‐yloxytripyrrolidinophosphonium hexafluorophosphate (PyBOP),^[^ ^33^ ^]^ a coupling agent well known from peptide synthesis, for the 3‐pyridinyl‐substituted derivative **2m**  and **8m**. *O* *rtho* fusions (here proceeding as intramolecular S_E_Ar/dehydration reactions) were performed in Morgan–Walls reactions with phosphoryl chloride or, when azide functions were present, with Hendrickson's reagent (Tf_2_O/Ph_3_PO).^[^ ^29^, ^34^ ^]^ Reaction conditions and isolated yields are summarized in Table 1. In general, amides **2** and **8** were obtained in good to excellent yields. Isolated yields of IPs **3** and **9** varied widely and seem to be correlated to the solubility of the respective compounds in the purification process. A direct formation of phenyl‐substituted IP **9h** from amine **7** and benzaldehyde using a Pictet–Spengler‐type reaction as published from the Hashmi group^[^ ^35^ ^]^ was possible with 32% yield (see Scheme 2, reaction e), which is significantly less than the yield observed in the respective two‐step sequence. 2‐Bromo‐substituted helicene **6** was obtained in an analogous approach starting with amine **1** and 4‐bromo‐2‐iodoaniline (see Scheme S1 for synthetic details).

**Table 1:** Conditions and yields for the synthesis of indolophenanthridines.

	[2,3‐*k*] product		[3,2‐*a*] product	
R, compound	Amide 2	Product 3	Amide 8	Product 9
Me, **a**	92%^[a]^	54%^[e]^	86%^[a]^	84%^[e]^
*t*Bu, **b**	99%^[a]^	25%^[^ ^[e]^, ^[g]^ ^]^	84%^[a]^	65%^[e]^
CF_3_, **c**	Quant.^[b]^	14%^[e]^	86%^[b]^	–^[^ ^[e]^, ^[h]^ ^]^
CH_2_Cl, **d**	92%^[b]^	53%^[e]^	87%^[b]^	34%^[e]^
CH_2_N_3_, **e**	97%^[b]^	77%^[f]^	85%^[b]^	85%^[f]^
(*E*)‐MeCH═CH, **f**	86%^[b]^	67%^[e]^	75%^[b]^	62%^[e]^
(*E*,*E*)‐Me(CH═CH)_2_, **g**	80%^[b]^	37%^[e]^	79%^[b]^	43%^[e]^
Ph, **h**	91%^[a]^	96%^[e]^	87%^[a]^	86%^[e]^
4‐MeO‐C_6_H_4_, **i**	quant.^[a]^	76%^[e]^	77%^[a]^	82%^[e]^
4‐F_3_C‐C_6_H_4_, **j**	98%^[a]^	93%^[e]^	82%^[a]^	78%^[e]^
mesityl, **k**	–^[h]^		^[^ ^[b]^, ^[j]^ ^]^	52%^[^ ^[e]^, ^[i]^ ^]^
2‐pyridinyl, **l**	^[c]^	77%^[^ ^[e]^, ^[i]^ ^]^		
3‐pyridinyl, **m**	57%^[d]^	85%^[e]^	82%^[d]^	78%^[e]^
1‐naphthalenyl, **n**	^[a]^	50%^[^ ^[e]^, ^[i]^ ^]^		

^[a]^ RCOCl, Et_3_N, CH_2_Cl_2_, 0°C, 1 h, then rt, overnight.

^[b]^ RCO_2_H, pyridine, PPAA, MeCN/EtOAc, −15 0°C, 1 h, then rt, overnight.

^[c]^ Picolinic acid, P(OPh)_3_, pyridine, 100°C, 18 h.

^[d]^ Niacin, iPr_2_NEt (DIPEA), rt, 5 min, then PyBOP, rt, 4.5 h.

^[e]^ POCl_3_, PhNO_2_, 150°C, 3–65.5 h.

^[f]^ Ph_3_PO, Tf_2_O, 0°C, 15 min, then amide, 0°C to rt, 3 h.

^[g]^ 43% based on recovered starting material (brsm).

^[h]^ No product obtained.

^[i]^ Yield over two steps.

^[j]^ Not purified.

Furthermore, we were successful in using amine **1** for the synthesis of CnCz (**4**) and ICz (**5**), which contain an additional pyrrole or pyridazine unit in place of the pyridine ring (Scheme 2). Their synthesis was achieved by either installation of a diazonium group and intramolecular azo coupling^[^ ^36^ ^]^ (→ **4**) or by formation of an azide in a Sandmeyer‐type reaction and thermal generation and insertion of a nitrene group^[^ ^37^ ^]^ (→ **5**). With this we had three further similarly shaped and arranged compounds in hand: bromo‐substituted IP **6**, which could serve as a possible electrophilic coupling partner, ICz (**5**) being more electron‐rich, and CnCz (**4**), likely being more electron‐deficient than parent [2,3‐k]‐IPs **3**.

**Scheme 3:**
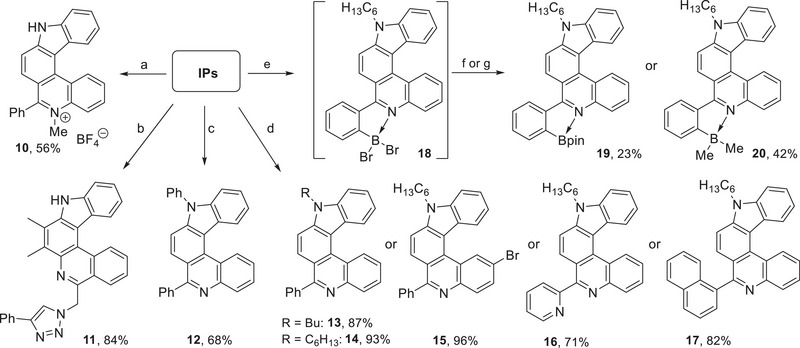
Modification of IPs. Conditions: (a) **3h**, Me_3_O^+^BF_4_^−^, CH_2_Cl_2_, rt, 70 h; (b) **9e**, ethynylbenzene, cat. CuSO_4_·5H_2_O, (+)‐sodium‐L‐ascorbate, CH_2_Cl_2_/THF/H_2_O (1:2:1); (c) **3h**, PhBr, cat. Pd_2_(dba)_3_, [(*t*Bu)_3_PH]BF_4_, Na, NaH, *t*BuOH, *o*‐xylene, 140°C, 14.5 h; (d) **3h**, **3l**, **3n**, or **6**, BuBr or *n*‐C_6_H_13_Br, KOH, DMF, 80°C, 18–19 h; e) **14**, BBr_3_, DIPEA, CH_2_Cl_2_, 50°C, 3.5–4 h; (f) Et_3_N, then pinacol, 50°C, overnight; (g) Et_3_N, then AlMe_3_ (2M in toluene), rt, overnight.

Next, we wanted to demonstrate different methods for modifying the synthesized azahelicenes at the pyrrole or pyridine nitrogen atoms or at other positions. To the best of our knowledge, there has been no comparable study to date. The known optical and DNA‐binding properties of phenanthridinium chromophores^[^ ^38^ ^]^ as well as an expected improved solubility in aqueous systems prompted us to try methylations at the pyridine nitrogen (see Scheme 3, reaction a). Surprisingly, this turned out to be challenging; commonly used methods like the reaction with methyl iodide failed. Similar difficulties have already been reported for the methylation of 5,10‐diaza[5]helicene.^[^ ^39^ ^]^ *N*‐Methylation of [2,3‐*k*]‐IP **3h** to furnish indolophenanthridinium salt **10** could finally be achieved with Meerwein's reagent (Me_3_O^+^BF_4_^−^).^[^ ^39^ ^]^ However, the respective [3,2‐*a*] derivative **9h** could not be methylated with any of the tested methods.

Click reactions are a powerful method for attaching helicenes to further molecules or to molecular ensembles (e.g., to biomolecules or polymers).^[^ ^14^ ^]^ An exemplary reaction of azide **9e** with phenylacetylene proceeded smoothly (see Scheme 3, reaction b) and triazole **11** was obtained in a good yield.^[^ ^40^ ^]^ A functionalization of the pyrrole NH group was studied using aryl‐substituted IPs. Arylation (→ **12**) was realized using a Buchwald–Hartwig amination with bromobenzene in presence of in situ prepared sodium *tert*‐butanolate (NaO*t*Bu) (see Scheme 3, reaction c).^[^ ^41^ ^]^ Alkylations (→ **13**–**17**) could be achieved in good yields with alkaline conditions (see Scheme 3, reactions d).^[^ ^42^ ^]^

The importance and versatility of organoboranes for applications ranging from organic synthesis (cross‐coupling reactions, Matteson homologation, etc.) to fluorescence imaging^[^ ^43^ ^]^ and polymers for biomedicine^[^ ^44^ ^]^ prompted us to exemplarily perform intramolecular C─H borylations^[^ ^45^ ^]^ of phenyl‐substituted and *N*‐alkylated [2,3‐*k*]‐IP **14** (see Scheme 3, reactions e–g). Electrophilic borylation with boron tribromide yielded dibromoborane species **18**, which was in situ transferred into (air‐)stable boronates **19** or **20**.^[^ ^45^ ^]^ Due to an apparent sluggish formation of intermediate **18**, only unsatisfying overall yields were observed for the final boronates.

**Scheme 4:**
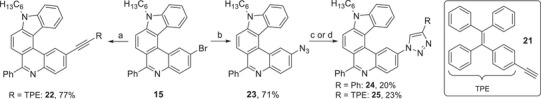
Synthesis of helicene conjugates. Conditions: (a) **21**, cat. PdCl_2_(PPh_3_)_2_, PPh_3_, cat. CuI, THF/Et_3_N (1:1), 60°C, 31 h; (b) *n*BuLi, THF, −78°C, 1 h, then TosN_3_, −78°C to rt, overnight; (c) phenylacetylene, cat. CuSO_4_·5H_2_O, (+)‐sodium‐L‐ascorbate, THF/H_2_O (1:1), 0°C to rt, 64 h; d) **21**, cat. CuSO_4_·5H_2_O, (+)‐sodium‐L‐ascorbate, THF/H_2_O (3:2), 0°C to rt, 64 h.

Previously, we observed a distinct aggregation‐induced emission (AIE) behavior for IPs **3h** and **9h**.^[^ ^27^ ^]^ This prompted us test, whether fluorescence intensities could be further enhanced by suitable modifications of the helicenes, as has already been reported for carbohelicene‐linked compounds.^[^ ^46^ ^]^ Hence, we reacted hexyl‐protected 2‐bromo‐substituted IP **15** with [2‐(4‐ethynylphenyl)ethene‐1,1,2‐triyl]tribenzene (**21**) in a Sonogashira reaction^[^ ^46^, ^47^ ^]^ to yield the helicene tetraphenylethylene (TPE) conjugate **22** with a good yield of 77% (see Scheme 4, reaction a). Furthermore, bromide **15** was subjected to a metal–halogen exchange with *n*BuLi and reacted with tosyl azide to furnish azide **23** in 71% yield. Click reactions with phenyl acetylene or TPE derivative **21**, respectively, led to the corresponding conjugates **24** and **25**. The rather low yields of 20% and 23% are probably due to a defunctionalization of the helicenes as a side reaction.

### 2.2 Characterization

All novel compounds were fully characterized by NMR and IR spectroscopy and by mass spectrometry. Crystal structures were determined of phenyl‐substituted IPs **3h** and **9h**. Absorption and emission spectra, partly while protonation with acids, were measured and AIE behavior of ICz (**5**), CnCz (**4**), [2,3‐*k*]‐IP **3h**, [3,2‐a]‐IP **9h**, and conjugates **24** and **25** was investigated. Results were complemented by quantum chemical calculations. Software packages and methods used for these calculations are given in the Supporting Information.

### 2.3 Structural properties

**Figure 1:**
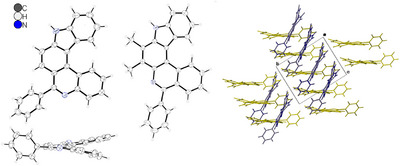
Molecular structure of phenyl‐substituted [2,3‐*k*]‐IP **3h** (left), of phenyl‐substituted [3,2‐*a*]‐IP **9h** (middle), and stacking pattern of **3h** (right) in the crystal.^[^ ^48^ ^]^

Crystals of racemic phenyl‐substituted [2,3‐*k*]‐IP **3h** and of [3,2‐*a*] derivative **9h** were grown by solution in THF/CH_2_Cl_2_ and THF/EtOH, respectively, and slow evaporation of the solvents (see Figure 1). x‐Ray diffraction analyses^[^ ^48^ ^]^ confirmed their expected unique non‐planar, screw‐shaped, and therefore axially chiral structures. (The structure of ICz [**5**] has already been reported.^[^ ^18^ ^]^) [3,2‐*a*] derivative **9h** shows a somewhat more pronounced helicality than [2,3‐*k*]‐IP **3h** (interplanar angles: 38.0° vs. 25.6°; sum of torsion angles: 42.7° vs. 39.2°). Both compounds crystallized as racemates with a columnar arrangement (see Figure 1, Figures S14 and S15).

Stronger hydrogen bonds between pyrrole hydrogen and pyridine nitrogen atoms can be deduced for **3h** (*d*_N─HN_ 2.163 ppm) and weaker ones for **9h** (2.383 ppm). (*M*)‐**3h** gave orthorhombic crystals (space group *Pca*2_1_) with parallel stacking of the molecules and (*M*)‐**9h** gave monoclinic crystals (*P*2_1_/*/* *n*) with anti‐parallel stacking.^[^ ^49^ ^]^ The discussed differences are most likely due to the additional methyl groups in [3,2‐*a*] derivative **9h**. The well‐defined packing in the crystals is consistent with the rather high melting points/ranges of the compounds: 313–314°C for CnCz (**4**), 282–283°C for [2,3‐*k*]‐IP **3h**, and 263–264°C for [3,2‐*a*]‐IP **9h**. A melting range of ICz (**5**) has already been published to be 280–282°C.^[^ ^28^ ^]^

Quantum chemical geometry optimizations revealed helicality for all compounds.Interplanar angles raise from 2.8° for ICz (**5**) to 29.1° for CnCz (**4**), 31.7° for [2,3‐*k*]‐IP **3h**, and 35.4° for [3,2‐*a*]‐IP **9h**. *N*‐Methylated derivative **10** shows a slightly increased interplanar angle of 34.6° compared to other [2,3‐*k*]‐IPs. For **3h** the calculated angle was overestimated compared to the measured one and for **9h** it was underestimated (see Tables S3 and S4). However, the calculations provide a good approximation and reveal that the angles of the [3,2‐*a*]‐fused IPs are generally larger than those of the [2,3‐*k*]‐IPs.

**Figure 2:**
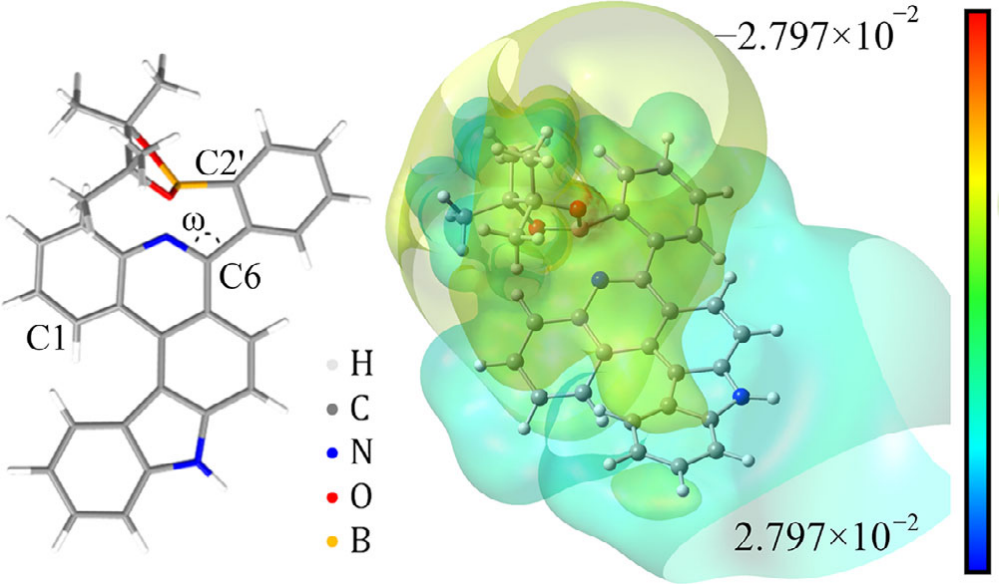
Calculated structure (left) and electron density map (right) of pyridine‐borane complex **19′**.

The calculated structure for Bpin derivative **19′** (as **19** without the *N*‐hexyl group) is depicted in Figure 2 (left). The C‐2′–B distance is 1.610 Å with an N→B distance of 1.709 Å and the torsion angle *ω* to the phenyl group is somewhat smaller due to restrictions arising from the additional N→B interaction (110.1° vs. 115.3° for **3h**). The calculations strongly suggest the presence of a Lewis acid/base borane‐pyridine complex, as has been reported for a number of comparable bi‐ and terphenyls^[^ ^50^ ^]^ and for further aza[5]helicenes.^[^ ^45^ ^]^ Furthermore, a larger dipole moment is predicted (9.95 vs. 5.91 Debye for **3h**). The electron density map shows an asymmetric charge distribution with a positively charged carbazole part and a negatively charged phenanthridine/boron site (Figure 2, right).

### 2.4 Racemization

The here synthesized helical compounds seem very prone to racemization if separated into their enantiomers. To get evidence for this assumption, we calculated zero point‐corrected activation energies for the racemization and obtained values of 25.0 kJ·mol^−1^ (6.0 kcal·mol^−1^) for [2,3‐*k*]‐IP **3**, 21.2 kJ·mol^−1^ (5.1 kcal·mol^−1^) for [3,2‐*a*]‐IP **9**, and 16.0 kJ·mol^−1^ (3.8 kcal·mol^−1^) for CnCz (**4**) (see Figure S17 and Table S11). As expected, ICz (**5**) has a vanishingly low racemization barrier of only 0.8 kJ·mol^−1^ (0.2 kcal·mol^−1^). All values are significantly lower than the barrier of pentahelicene (24.1 kcal·mol^−1^; *t*_½_ = 29 h)^[^ ^51^ ^]^ and calculated half‐lifes (*t*_½_) of enantiomerization are 5.6 ns or lower. Accordingly, no attempts were made for a chiral resolution of the compounds.

### 2.5 FMO analysis and singlet‐triplet‐energies

Calculated HOMO–LUMO gaps for ICz (**5**) and IPs **3h**, **3m**, **9h**, **10**, and **19′** are in the range of 3.96−4.06 eV, except CnCz (**4**) with a gap of 2.98 eV (see Table 2). Inspection of the molecular orbitals reveals a slightly higher contribution of the carbazole moiety to the HOMO, while the phenanthridine and cinnoline moieties are less represented in this MO. The respective contributions to the LUMO are correspondingly inverted (see Tables S5 and S6). Protonation results in smaller HOMO–LUMO gaps for the IPs, e.g., 4.60 eV for **3a** vs. 4.07 eV for **3a**·H^+^. TDA (Tamm–Dancoff approximation) calculations predict positive singlet‐triplet gaps of 0.45−0.68 eV for ICz (**5**) and phenyl‐substituted IPs **3h** and **9h**; *E*_S1_ and *E*_T1_ values for ICz are comparable with published data^[^ ^18^ ^]^ (see Table 2). An *E*_S1_ value calculated for CnCz (**4**) is slightly smaller than the respective *E*_T1_ value, resulting in a small inverted (negative) S_1_‐T_1_ gap (singlet‐triplet inversion).

**Table 2:** Photophysical properties of selected aza[5]helicenes.

Compound	*λ*_abs_ max (THF)	*λ*_em_ max (THF)^[a]^	*λ*_em_ max (TfOH)^[a]^	Stokes shift (TfOH)	HOMO	LUMO	Δ_LUMO–HOMO_	*E*_S1_	*E*_T1_	Δ*E*_ST_
	[nm]				[eV]					
ICz (**5**)	329, 343	394, 416	394, 416	51	−5.57	−1.34	4.23	3.25	2.80	0.45
CnCz (**4**)	314	473	575	261	−4.99	−2.01	2.98	2.50	2.50	−0.0032
[2,3‐*k*]‐IP **3h**	307	404	483	176	−6.20	−1.61	4.59	3.54^[e]^	3.03^[e]^	0.51^[e]^
[2,3‐*k*]‐IP **3m**	309	409	574	265	−6.24	−1.69	4.55	^[d]^	^[d]^	^[d]^
[3,2‐*a*]‐IP **9h**	322	432	593	271	−5.84	−1.60	4.24	3.62^[e]^	2.94^[e]^	0.68^[e]^
**10**	326, 408	526	532	124	−6.93	−2.97	3.96	^[d]^	^[d]^	^[d]^
**19**	308	408	505	197	−6.34^[c]^	−2.28^[c]^	4.06^[c]^	^[d]^	^[d]^	^[d]^
**22**	321	454^[b]^	588^[b]^	267	^[d]^	^[d]^	^[d]^	^[d]^	^[d]^	^[d]^
**25**	318	423^[b]^	511^[b]^	193	^[d]^	^[d]^	^[d]^	^[d]^	^[d]^	^[d]^

^[a]^ *λ*_ex_ = 330 nm.

^[b]^ *λ*_ex_ = 345 nm.

^[c]^ Calculated for **19′**.

^[d]^ Not calculated.

^[e]^ Calculated for the parent framework (R = H).

### 2.6 Optical properties

Solvatochromism was exemplarily studied for the phenyl‐substituted IPs. Emission of [3,2‐*a*] derivative **9h** ranges from 421 nm in toluene to 447 nm in DMSO (see Figure S12), while emission of the corresponding [2,3‐*k*] derivative **3h** is less solvent‐dependent (401 nm in toluene vs. 410 nm in pyridine). These observations are in accordance with the Kamlet–Taft and Reichardt polarizability scales.^[^ ^52^ ^]^

**Figure 3:**
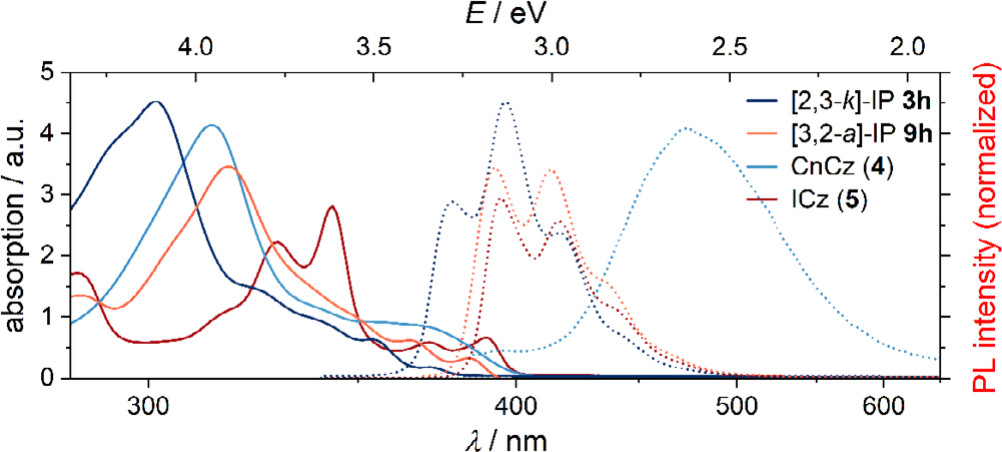
UV/Vis absorbance (solid lines) and fluorescence spectra (dashed lines) of phenyl‐substituted IPs **3h** and **9h**, CnCz (**4**) and ICz (**5**) (THF, *λ*_ex_ = 330 nm).

THF was found to be a suitable solvent for studying the optical properties described in the following. As typical extended π‐conjugated systems, ICz (**5**), CnCz (**4**), [2,3‐*k*]‐IPs **3**, and [3,2‐*a*]‐IPs **9** revealed similar UV/Vis absorption patterns, which are comparable to those of dibenzo[*c*,*g*]phenanthrene, i.e., of the parent [5]helicene^[^ ^53^ ^]^ (see Figure 3; further absorption and emission spectra are given in Figures S8–S11). Absorption maxima range from 302 to 343 nm.

Within the [2,3‐*k*]‐IPs **3**, absorption maxima were increasingly redshifted with increasing conjugation [e.g., 302 nm for **3a**, R = Me vs. 316 nm for **3g**, R = (*E*,*E*)‐Me(CH═CH)_2_]. *N*‐Methylation (→ **10**) resulted in a significant redshift and formation of a new absorption maximum (326 and 408 nm). In contrast, the type of aromatic substituents and a borylation had a minor influence on the maxima (306 nm for **3n**, R = 1‐naphthalenyl, to 310 nm for **3l**, R = 2‐pyridinyl). Similar trends were observed for [3,2‐*a*] derivatives **9** with generally slightly more redshifted absorptions and a shoulder for many compounds at a slightly longer wavelength (e.g., 324 and 371 nm for **9m**, R = 3‐pyridinyl).

Calculated absorption spectra at the PBE0‐D3(BJ) /def2‐TZVP level (for details see Supporting Information) generally agree well with the measured spectra. The absorption bands can be mainly assigned to HOMO–1 → LUMO and HOMO → L+1 transitions with minor contributions of H–1 → L+1 and HOMO → LUMO transitions (see Tables S8–S10).

The azahelicenes display broadband fluorescence, with the emission strongly dependent on the scaffold and substituents. Maxima are observed at 394 and 416 nm (ICz, **5**), 473 nm (CnCz, **4**), 396–526 nm ([2,3‐*k*]‐IPs **3**), and 390–498 nm ([3,2‐*a*]‐IPs **9**) (*λ*_ex_ = 330 nm). TPE conjugates emit at 454 nm (**24**) and 423 nm (**25**) (*λ*_ex_ = 345 nm). While a fluorescence quantum yield of 20.2% for ICz (**5**) has been reported,^[^ ^18^ ^]^ small quantum yields were expected for the IPs^[^ ^54^ ^]^ and therefore no measurements were performed. Photophysical properties for the different types of azahelicenes are summarized in Table 2.

**Figure 4:**
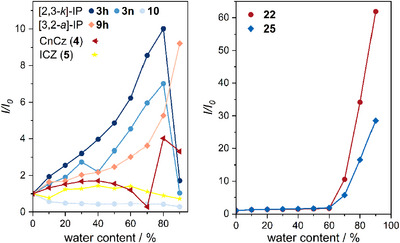
Water fraction‐dependent fluorescence intensity (*I*/*I*_0_) of [2,3‐*k*]‐IPs **3h**, **3n**, and **10**, [3,2‐*a*]‐IP **9h**, CnCz (**4**), and ICz (**5**) (*λ*_ex_ = 330 nm) (left), and of conjugates **22** and **25** (*λ*_ex_ = 345 nm) (right).

Selected aza[5]helicenes were investigated for their emission behavior in THF solutions with 0% up to 90% water fraction (see Figures 4 and S13). Helicene‐TPE conjugates **22** and **25** exhibited an outstanding AIE behavior. Their fluorescence intensities at 90% water content are about 62 and 28.5 times the initial value (*I*/*I*_0_), respectively. For phenyl‐substituted [3,2‐*a*]‐IP **9h** fluorescence intensity increased about 9.2 times. Similarly, a 10‐fold and 7‐fold increase was observed for the phenyl‐ and naphthalenyl‐substituted [2,3‐*k*]‐IPs **3h** and **3n** at 80% water content. The fluorescence intensity of CnCz (**4**) also depends on the solvent composition and increases 3.3–4‐fold, but the relationship is not that clear. In contrast, emissions of ICz (**5**) and of phenanthridinium ion **10** were only slightly affected by the THF/H_2_O ratio.

Our calculations suggest that the IPs racemize rapidly at room temperature (vide supra). We assume that an increasing water content leads to aggregation of the molecules, which impedes their intramolecular motion (i.e., racemization) and thus leads to enhanced emission. This phenomenon is known as aggregation‐enhanced/ ‐induced emission (AEE/ AIE)^[^ ^55^, ^56^ ^]^ and has already been described for helicenes.^[^ ^57^ ^]^ The underlying mechanisms are still subject of ongoing research.^[^ ^58^, ^59^, ^60^ ^]^ We found that AIE seems to be more pronounced for the IPs and for CnCz (**4**), especially when linked with the AIE luminophore TPE. For ICz (**5**), a slightly better AIE behavior in MeOH/H_2_O (as compared with THF/H_2_O) has been reported.^[^ ^18^ ^]^

We assume that a lower solubility in the herein used THF/H_2_O mixture leads to π–π stacking and thus to efficient quenching during aggregation.^[^ ^58^ ^]^ It remains an open question whether the fluorescence intensity of the IPs decreases at high water contents because it is confined to the outermost molecules in the aggregates, or because the compounds become insoluble and precipitate.^[^ ^58^ ^]^ In contrast to the other investigated compounds, phenanthridinium salt **10** should be significantly more soluble in all THF/H_2_O mixtures, what might prevent an aggregation of the molecules and thus a change in its emission behavior.

### 2.7 Basicity of the azahelicenes

**Figure 5:**
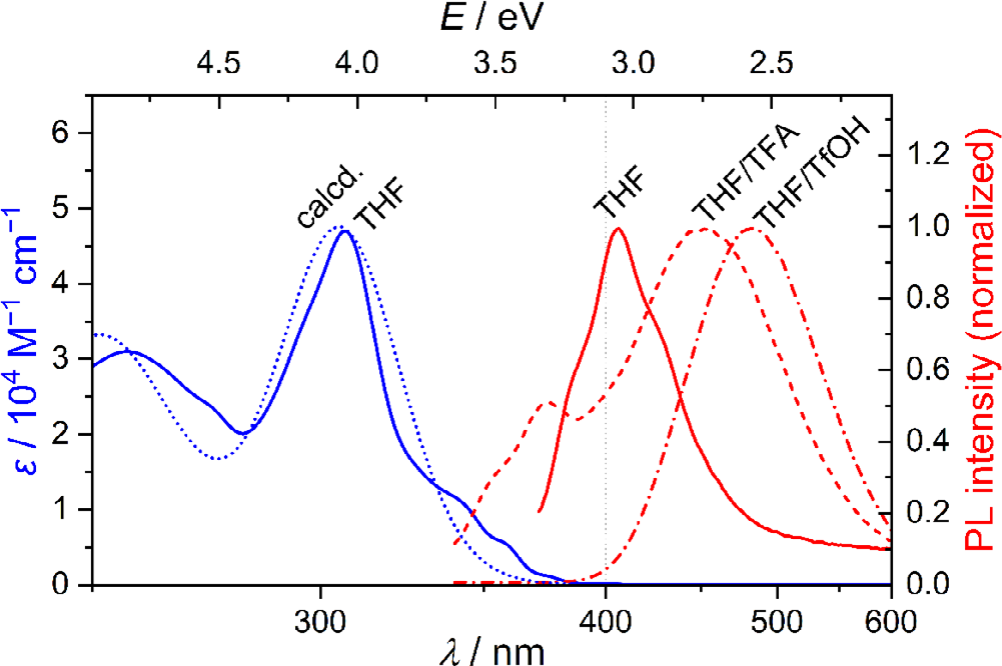
Calculated and measured UV/Vis absorbance (blue) and fluorescence spectra in THF, THF/TFA, and THF/TfOH (red) of phenyl‐substituted IP **3h** (*λ*_ex_ = 330 nm).

We were able to confirm that the sp^2^ lone pair of the pyridine and pyridazine nitrogen atoms is protonatable, where a protonation leads to changes in absorption and emission spectra (acidochromism). Figure 5 displays calculated and measured UV/Vis (blue) and emission spectra (red) of [2,3‐*k*]‐IP **3h** in THF before and after addition of trifluoroacetic acid (TFA, p*K*_a_ = 0.23) or trifluoromethanesulfonic acid (TfOH, p*K*_a_ = −5.21) (*λ*_ex_ = 330 nm); further spectra are given in (Figures S2–S7).

Upon titration of different IPs, we made three important observations: (1) Addition of TfOH changed the UV/Vis spectrum of methyl‐substituted IP **3a**, indicating its partial protonation. However, no changes were observed for the [3,2‐*a*] derivative **9a** upon addition of TFA. (2) Addition of 1 eq. TfOH led to the evolution of new bands at higher wavelengths for all investigated IPs and CnCz (**4**) (e.g., 319 and 382 nm [**3a**]; 332 and 412 nm [**9a**]), which can be attributed to the evolving pyridinium cations. As expected, ICz (**5**) and **10** were not protonable. (3) In general, addition of TfOH led to stronger bathochromic shifts of the emission than addition of TFA. The most significant shifts were observed for 3‐pyridinyl‐substituted [2,3‐*k*]‐IP **3m**, phenyl‐substituted [3,2‐*a*]‐IP **9h**, and TPE conjugate **22** (see Table 2), leading to emissions in the yellow visible range. We conclude that these compounds, in particular the [2,3‐*k*]‐ and [3,2‐*a*]‐IPs, show a different protonation behavior. Relevant factors might include basicity of the nitrogen atoms and the pH of the solutions, hydrogen bonding, and stability of the polar (protonated) excited states. A similar observation has already been made for other aza‐ and diazahelicenes by Šolínová et al.^[^ ^61^ ^]^

For a better understanding, we calculated the basicities of all azahelicene types using the proton exchange method^[^ ^62^ ^]^ and obtained relative p*K*_a_ values of 6.65 for CnCz (**4**), 10.0 for [2,3‐*k*]‐IP **3a** (R = Me), and 9.55 for [3,2‐*a*]‐IP **9a** (R = Me). These values were determined by correlating the compounds’ solvent‐dependent free energies with an experimental p*K*_a_ value of pyridinium as reference (Ref‐H^+^; p*K*_a_ = 5.23 at 25°C^[^ ^63^ ^]^) (Equation 1).

(1)

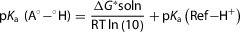

$$\mathrm{IP}+H^{+}\to \mathrm{IP}-H^{+}$$

(2) $$PA=E\left(\mathrm{IP}\right)+E\left(H^{+}\right)-E\left(\mathrm{IP}-H^{+}\right)$$

(3) $$\Delta PA=PA_{[3,2-a]}-PA_{[2,3-k]}$$

**Scheme 5:**
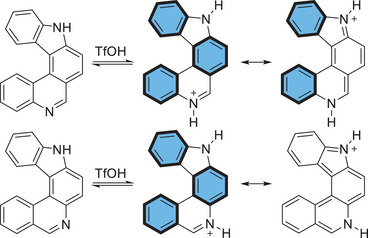
Resonance formulas of protonated [2,3‐*k*]‐IP **3** (top) and [3,2‐*a*]‐IP **9** (bottom). (Only the parent ring systems are given; fully intact benzene rings are highlighted).

We used a further method for comparison and determined proton affinities (*PA*) for gas phase reactions of neutral IPs with a proton (Equations 2 and 3).^[^ ^64^ ^]^ A value of 34.3 kJ·mol^−1^ (8.2 kcal·mol^−1^) was obtained for the difference in proton affinities (Δ*PA*) of IPs **3a** and **9a** (R = Me). p*K*_a_ and *PA* values indicate that [3,2‐*a*]‐IP **9a** is the weaker base, what is consistent with the experimental observation that [3,2‐*a*] derivatives required a stronger acid for protonation than [2,3‐*k*]‐IPs. This finding is easily understood by applying the rule of *Clar*:^[^ ^65^ ^]^ Protonated [2,3‐*k*]‐IPs are more stable since resonance formulas with more fully intact benzene rings can be written (Scheme 5).

## 3 CONCLUSIONS

Four different types of carbazole‐derived aza[5]helicenes were synthesized in an efficient approach using an *ortho* fusion strategy: variously substituted indolo[2,3‐*k*]‐ and ‐[3,2‐*a*]phenanthridines, an indolocarbazole, and a cinnolinocarbazole. Subsequent reactions gave organoboranes, a phenanthridinium salt, triazoles, and conjugates with tetraphenylethylene. Results were complemented by XRD and quantum chemical calculations. The compounds and their protonated forms exhibit significant Stokes shifts of up to 271 nm, resulting in emission shifts into the yellow visible region. Aggregation‐induced emission was observed and is particularly pronounced for the conjugates: Fluorescence intensities were found to be up to 62 times stronger in 9:1 THF/water mixtures than in pure THF. These findings are very promising for possible applications in the field of optoelectronics or sensing.

## Supporting Information

The authors have cited additional references within the Supporting Information.^[^ ^66^, ^67^, ^68^, ^69^, ^70^, ^71^, ^72^, ^73^, ^74^, ^75^, ^76^, ^77^, ^78^, ^79^, ^80^, ^81^, ^82^, ^83^, ^84^, ^85^, ^86^, ^87^, ^88^, ^89^, ^90^, ^91^ ^]^

## Conflicts of Interest

The authors declare no conflicts of interest.

## Supporting information



Supporting Information

## Data Availability

The data that support the findings of this study are available in the supporting information of this article.
