# Supplementary material for: Fluorescent Carbazole‐Derived Aza[5]Helicenes: Synthesis, Functionalization, and Characterization
Source: Chemistry. 2025 Apr 17;31(27):e202501081. doi: 10.1002/chem.202501081 (PMC12080310; doi:10.1002/chem.202501081)
Supplement: Supplementary file 1 — Supporting Information [file CHEM-31-e202501081-s001.pdf]

Supporting Information

Fluorescent Carbazole-derived Aza[5]helicenes: Synthesis, Functionalization, and Characterization

Inka Marten,[a] Melina E. A. Dilanas,[b] and Joachim Podlech[a]*

[a] Institut für Organische Chemie, Karlsruher Institut für Technologie (KIT), 76131 Karlsruhe, Kaiserstraße 12, Germany

[b] Institut für Anorganische Chemie, Karlsruher Institut für Technologie (KIT), 76131 Karlsruhe, Kaiserstraße 12, Germany

* Email: joachim.podlech@kit.edu

Table of Contents

1. **Possible Indolophenanthridines** **3**
2. **General Information** **8**
3. **Syntheses** **9**
   3.1 General Procedures 9
   3.2 Cinnolinocarbazole and Indolocarbazole 10
   3.3 Indolo[2,3-*k*]phenanthridines 3 11
   3.4 Indolo[3,2-*a*]phenanthridines 9 20
   3.5 Modification of Indolophenanthridines 3 and 9 27
   3.6 2-Bromo-indolo[2,3-*k*]phenanthridine 6 33
   3.7 Modifications of Indolophenanthridine 6 35
4. **Optical Properties** **39**
   4.1 Calibration Spectra 39
   4.2 UV/Vis Titration Spectra 40
   4.3 UV/Vis Absorbance and Fluorescence Spectra 44
      4.3.1 Indolocarbazole (5) and Cinnolinocarbazole (4) 44
      4.3.2 Indolo[2,3-*k*]phenanthridines 44
      4.3.3 Indolo[3,2-*a*]phenanthridines 47
      4.3.4 Helicene-TPE conjugates 22 and 25 49
   4.4 Solvatochromism 49
   4.5 Emission Behavior in THF/H_2O Solutions 50
5. **XRD – Structural Data** **51**
6. **Computational Studies** **52**
   6.1 Computational Methods 52
   6.2 Calculated Structures 53
   6.3. Chiroptic Properties and Racemization Barriers 63
7. **Appendix** **65**
   7.1. ^{1}H NMR and ^{13}C NMR Spectra 65
   7.2 Computational Data 99
      7.2.1 Indolocarbazole (ICz, 5) 99
      7.2.2 Cinnolinocarbazole (4) 101
      7.2.3 Indolo[2,3-*k*]phenanthridines 103
      7.2.4 Indolo[3,2-*a*]phenanthridines 115
      7.2.5 Pyridine 126
8. **References** **127**

1. Possible Indolophenanthridines

In alphabetical order (not counting for the indicated hydrogens, which are placed here at the nitrogen atoms of the 5-membered rings or arbitrarily). The phenanthridine moiety is always depicted in a uniform orientation and the indole is attached accordingly.

Table S1: Possible indolophenanthridines.

#	Indolophenanthridine	IUPAC name	#	Indolophenanthridine	IUPAC name
1	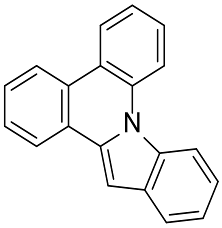	indolo[1,2-*f*] phenanthridine	2	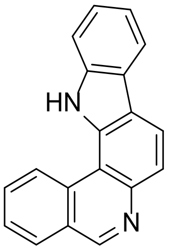	13*H*-indolo[2,3-*a*] phenanthridine
3	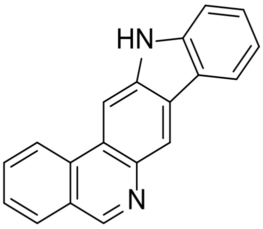	12*H*-indolo[2,3-*b*] phenanthridine	4	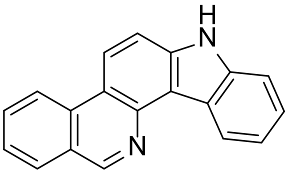	7*H*-indolo[2,3-*c*] phenanthridine
5	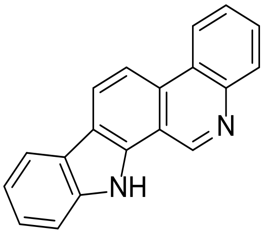	13*H*-indolo[2,3-*i*] phenanthridine	6	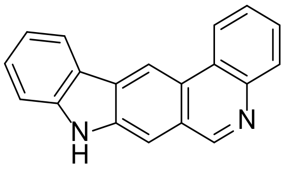	8*H*-indolo[2,3-*j*] phenanthridine
7	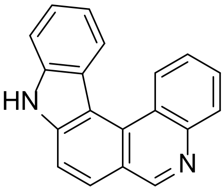	9*H*-indolo[2,3-*k*] phenanthridine	8	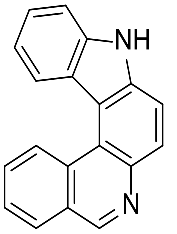	9*H*-indolo[3,2-*a*] phenanthridine
9	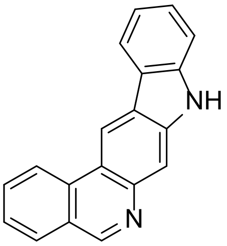	8*H*-indolo[3,2-*b*] phenanthridine	10	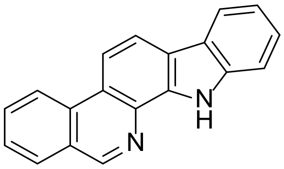	13*H*-indolo[3,2-*c*] phenanthridine

11 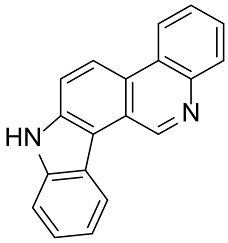 7*H*-indolo[3,2-*i*] phenanthridine

12 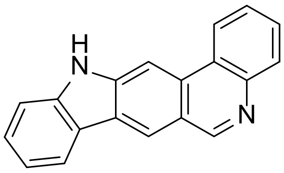 12*H*-indolo[3,2-*j*] phenanthridine

13 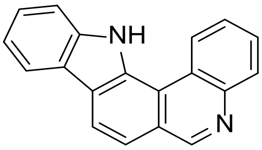 13*H*-indolo[3,2-*k*] phenanthridine

14 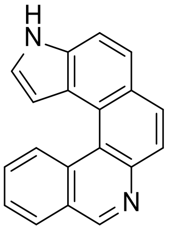 7*H*-indolo[4,5-*a*] phenanthridine

15 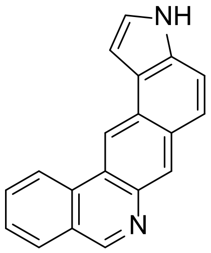 3*H*-indolo[4,5-*b*] phenanthridine

16 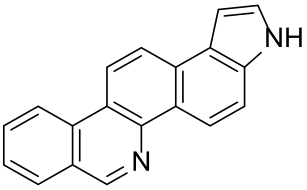 3*H*-indolo[4,5-*c*] phenanthridine

17 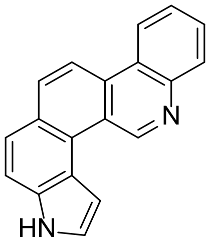 3*H*-indolo[4,5-*i*] phenanthridine

18 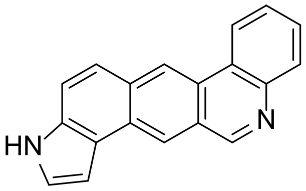 3*H*-indolo[4,5-*j*] phenanthridine

19 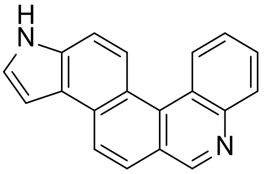 1*H*-indolo[4,5-*k*] phenanthridine

20 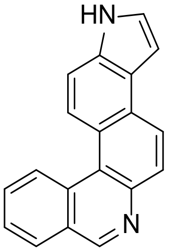 1*H*-indolo[5,4-*a*] phenanthridine

21 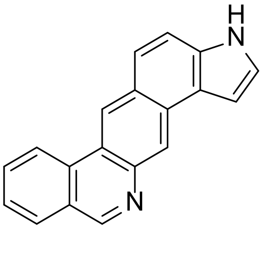 3*H*-indolo[5,4-*b*] phenanthridine

22 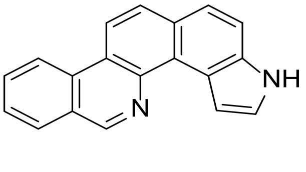 3*H*-indolo[5,4-*c*] phenanthridine

23 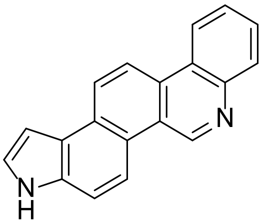 3*H*-indolo[5,4-*i*] phenanthridine

24 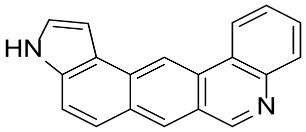 3*H*-indolo[5,4-*j*] phenanthridine

25 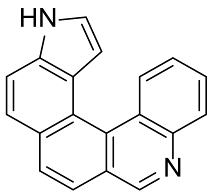 7*H*-indolo[5,4-*k*] phenanthridine

26 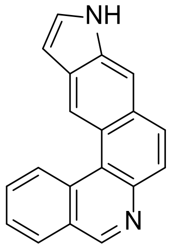 10*H*-indolo[5,6-*a*] phenanthridine

27 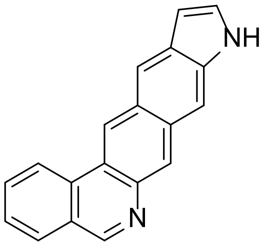 9*H*-indolo[5,6-*b*] phenanthridine

28 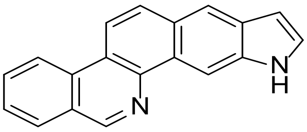 10*H*-indolo[5,6-*c*] phenanthridine

29 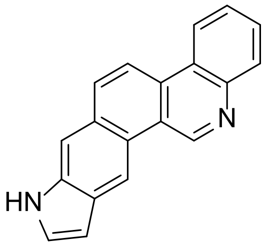 8*H*-indolo[5,6-*i*] phenanthridine

30 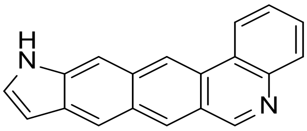 11*H*-indolo[5,6-*j*] phenanthridine

31 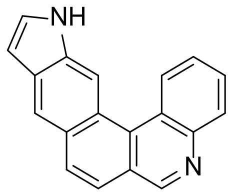 12*H*-indolo[5,6-*k*] phenanthridine

32 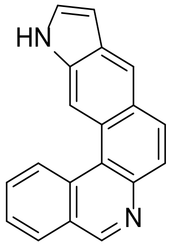 12*H*-indolo[6,5-*a*] phenanthridine

33 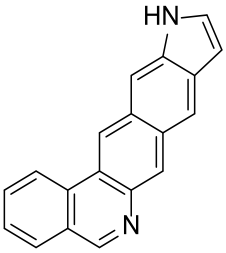 11*H*-indolo[6,5-*b*] phenanthridine

34 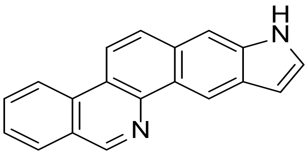 8*H*-indolo[6,5-*c*] phenanthridine

35 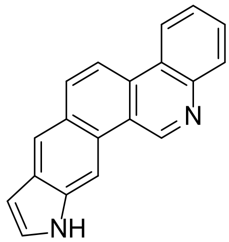 10*H*-indolo[6,5-*i*] phenanthridine

36 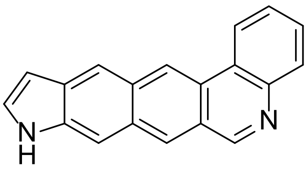 9*H*-indolo[6,5-*j*] phenanthridine

37

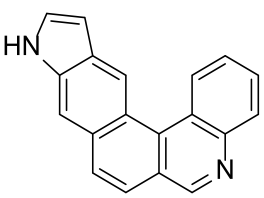

10*H*-indolo[6,5-*k*] phenanthridine

38

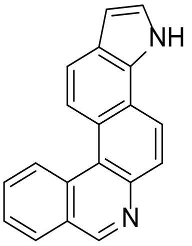

11*H*-indolo[6,7-*a*] phenanthridine

39

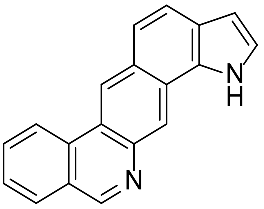

1*H*-indolo[6,7-*b*] phenanthridine

40

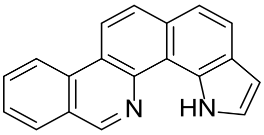

5*H*-indolo[6,7-*c*] phenanthridine

41

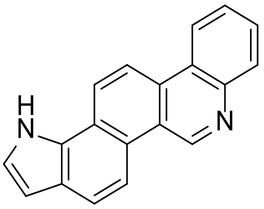

1*H*-indolo[6,7-*i*] phenanthridine

42

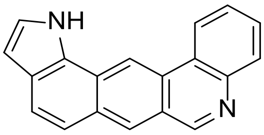

1*H*-indolo[6,7-*j*] phenanthridine

43

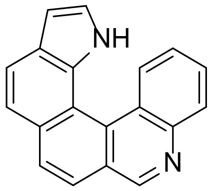

9*H*-indolo[6,7-*k*] phenanthridine

44

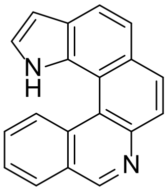

1*H*-indolo[7,6-*a*] phenanthridine

45

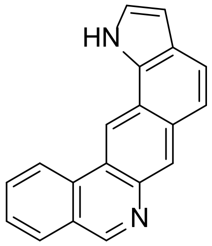

1*H*-indolo[7,6-*b*] phenanthridine

46

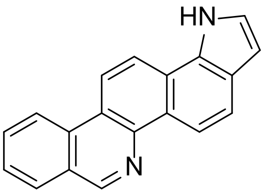

1*H*-indolo[7,6-*c*] phenanthridine

47

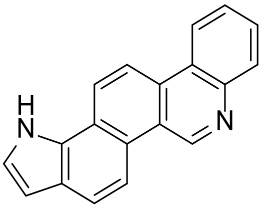

1*H*-indolo[6,7-*i*] phenanthridine

48

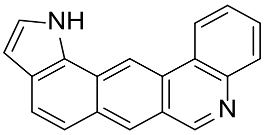

1*H*-indolo[6,7-*j*] phenanthridine

49

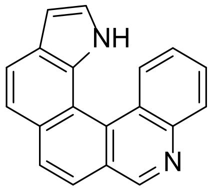

9*H*-indolo[6,7-*k*] phenanthridine

50

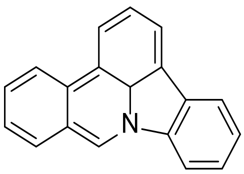

3a[1]*H*-indolo[3,2,1-*de*] phenanthridine

51
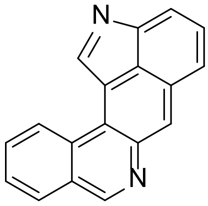

indolo[3,4-*ab*]
phenanthridine

52
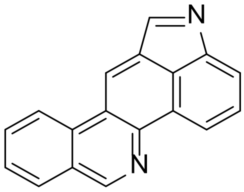

indolo[3,4-*bc*]
phenanthridine

53
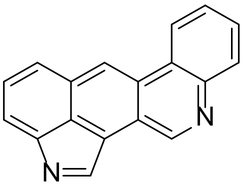

indolo[3,4-*ij*]
phenanthridine

54
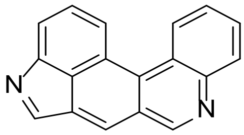

indolo[3,4-*jk*]
phenanthridine

55
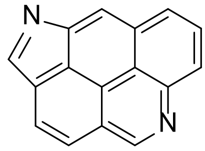

indolo[3,4,5,6-
klmn]
phenanthridine

56
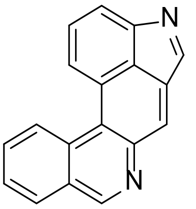

indolo[4,3-*ab*]
phenanthridine

57
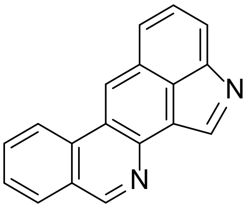

indolo[4,3-*bc*]
phenanthridine

58
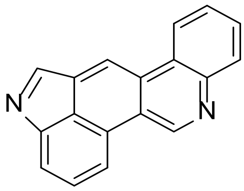

indolo[4,3-*ij*]
phenanthridine

59
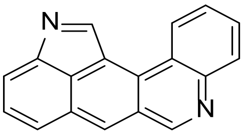

indolo[4,3-*jk*]
phenanthridine

60
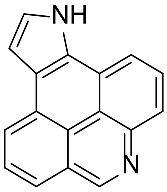

11*H*-
indolo[4,5,6,7-
lmn]
phenanthridine

61
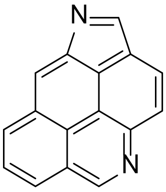

indolo[6,5,4,3-
lmna]
phenanthridine

62
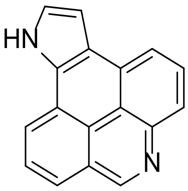

9*H*-indolo[7,6,5,4-
lmn]
phenanthridine

2. General Information

Unless otherwise noted, all solvents, reagents, and starting materials were purchased from commercial suppliers and used without further purification. Syntheses of amine **1**, amides **2a**, **2b**, **2f**, **2h**, **2m**, and indolo[2,3-*k*]phenanthridines **3a**, **3b**, **3f**, **3h**, **3n** as well as amine **7**, amides **8a**, **8b**, **8f**, **8h**, **8m**, and indolo[3,2-*a*]phenanthridines **9a**, **9b**, **9f**, **9h**, and **9m** have already been reported in a preceding short communication.[27] [2-(4-Ethynylphenyl)ethene-1,1,2-triyl]tribenzene (**21**) was synthesized following a published protocol.[70] THF and 1,4-dioxane were distilled from sodium and CH_2Cl_2 was distilled from CaH_2 prior to use. All moisture-sensitive reactions were carried out under an oxygen-free argon atmosphere using oven-dried glassware and a vacuum line (Schlenk technique). Flash column chromatography was carried out using Merck silica gel 60 (230–400 mesh). Analytical thin layer chromatography (TLC) was performed on commercially available Merck F_{254} precoated plates and visualized by fluorescence quenching and staining in a basic $KMnO_4$ solution (mixture of 3.00 g $KMnO_4$, 20.0 g K_2CO_3, and 5 mL 5% NaOH solution in 300 mL H_2O). ^1H and ^{13}C NMR spectra were recorded on a Bruker Avance 400 and a Bruker Avance DRX 500 spectrometer. The spectra were calibrated using the residual solvent signals and chemical shifts were reported in parts per million (ppm) referenced to 0.0 ppm for the signals of tetramethylsilane. Data were reported as follows: chemical shift, multiplicity (s = singlet, d = doublet, t = triplet, q = quartet, m = multiplet, br = broad), coupling constants *J* (Hz), integration, and assignment. ^{13}C NMR spectra were recorded with broadband decoupling and signals were assigned by COSY, DEPT, HSQC, and HMBC experiments. IR spectra were recorded on a Bruker Alpha FT-IR spectrometer using attenuated total reflection (ATR) on diamond; absorbance frequencies are reported in reciprocal centimeters (cm^{-1}). FAB and EI mass spectra were recorded with a Finnigan MAT-95 spectrometer (analyzer type: double-focusing sector field mass spectrometer with reverse Nier-Johnson geometry); a Q Exactive Orbitrap spectrometer from Thermo Fisher Scientific was used for ESI mass spectra. Quantitative UV/Vis spectra were measured with a Cary 60 UV/Vis spectrophotometer from Agilent in quartz glass cuvettes with a pathlength of 1.00 cm from Hellma, which were tempered to 20 °C. Positive displacement pipettes MICROMAN E M1000E, M100E, and M10E from Gilson were used. Linearity of the results was checked according to the Lambert-Beer law. Fluorescence spectra were recorded with a Fluoromax-4 from HORIBA. Probes were measured with concentrations of 5–30 µM at 20 °C in quartz glass cuvettes. The device was calibrated with the signal from Raman scattering of water. Melting points were determined using the capillary method with the OptiMelt MPA100 melting point analyser from Stanford Research Systems with a ramp rate of 1 °C/min. The samples were previously dried in a high vacuum and pulverized. The average of two non-corrected measurements is given. Single crystals for X-ray crystallography were mounted in perfluoropolyalkyl ether oil on a cryo loop and then brought into the cold nitrogen stream of a low-temperature device (Oxford Cryosystems Cryostream unit) so that the oil solidified. Diffraction data were collected using a Stoe IPDS II diffractometer and graphite-monochromated Mo-Kα (0.71073 Å) radiation. The structures were solved by intrinsic phasing with SHELXT[66] followed by full-matrix least-squares refinement using SHELXL-2014/7[67] and OLEX2.[68] All non-hydrogen atoms were refined anisotropically. The contribution of the hydrogen atoms in their calculated positions, was included in the refinement using a riding model.

3. Syntheses

3.1 General Procedures

GP1: Synthesis of Amides 2, 8 with Acid Chlorides (Method a)

Following published protocols,[29,30] acid chloride in anhydrous CH_2Cl_2 (~1/10 of total amount) is added under an argon atmosphere to a cooled (0 °C) solution of amine and Et_3N in anhydrous CH_2Cl_2 (~9/10 of total amount). The reaction mixture is stirred at 0 °C for 1 h, warmed to room temperature, and stirred overnight. Saturated aqueous $NaHCO_3$ solution (10 mL) is added, and the aqueous layer is extracted with CH_2Cl_2 (3×20 mL). The combined organic layers are washed with H_2O (50 mL). If a precipitate arises, MeOH (max. 10% v/v) is added until complete dissolution. The organic layer is dried ($MgSO_4$), concentrated under reduced pressure, and purified by column chromatography or reacted without further purification.

GP2: Synthesis of Amides 2, 8 with Carboxylic Acids (Method b)

Following a published protocol,[31] PPAA (propanephosphonic acid anhydride; ≥50% w/w in MeCN) is slowly added under an argon atmosphere to a cooled (−15 °C) suspension of an amine (1.00 equiv.), a carboxylic acid and pyridine in MeCN/EtOAc placed in a pyrex tube. After sealing the tube, the reaction mixture is stirred at 0 °C for 1 h, warmed to room temperature, and stirred overnight. 1M HCl (5 mL) is added and the mixture is stirred for 10 min. The layers are separated, and the aqueous layer is extracted with CH_2Cl_2 (3×20 mL). The combined organic layers are washed with H_2O (2×50 mL), dried ($MgSO_4$), concentrated under reduced pressure, and purified by column chromatography.

GP3: Cyclization of Amides 2, 8 with $POCl_3$

Following a published protocol,[29] $POCl_3$ in $PhNO_2$ (~1/6 of total amount) is slowly added to a degassed solution (ultrasonication, 10 min) of amide **2** or **8** (1.00 equiv.) in $PhNO_2$ (~5/6 of total amount) and heated to 150 °C for 3–65.5 h. After cooling to room temperature, saturated aqueous $NaHCO_3$ solution is added (~25 mL) and the aqueous layer is extracted with CH_2Cl_2 (3×20 mL). The combined organic layers are washed with H_2O (50 mL). If a precipitate arises, MeOH (max. 10% v/v) is added until complete dissolution. The organic layer is dried ($MgSO_4$), concentrated under reduced pressure, and purified by column chromatography.

GP4: Alkylation of Indolophenanthridines

According to a published protocol,[42] a degassed (ultrasonication, 10 min) suspension of indolophenanthridine **3** or **9** (1.00 equiv.), alkyl bromide and mortared KOH in anhydrous DMF was stirred at 80 °C for 16–40 h. After cooling, CH_2Cl_2 (30 mL) were added. The mixture was washed with H_2O (3×50 mL), dried ($MgSO_4$) and the solvent was removed under reduced pressure, and purified by column chromatography.

3.2 Cinnolinocarbazole and Indolocarbazole

9*H*-Cinnolino[3,4-*c*]carbazole (4)

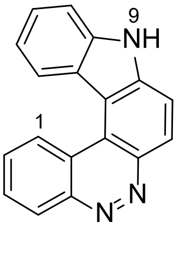

According to a published protocol,[36] $NaNO_2$ (43.2 mg, 626 µmol, 1.29 equiv.) in 1 mL H_2O was slowly added to a cooled (0 °C) solution of 4-(2-aminophenyl)-9*H*-carbazole (**4**, 125 mg, 485 µmol, 1.00 equiv.) in 3 mL 1M $HCl_{(aq)}$. The mixture was stirred for 2 h while slowly warming to room temperature. H_2O (40 mL) and EtOAc (40 mL) followed by sat. $NaHCO_3$ solution were added, until no further gas formation was seen (pH ~ 8). The aqueous layer was extracted with EtOAc (3 × 50 mL). The combined organic layers were washed with H_2O (20 mL), dried ($MgSO_4$) and concentrated under reduced pressure. **14** was obtained as yellow powder (132 mg, 490 µmol, quant.); recrystallization from EtOH/EtOAc (~20:1) afforded fine yellow crystals.

m.p. 313–314 °C; ^1H NMR (500 MHz, DMSO-d_6, ppm): δ = 12.56 (s, 1 H, NH), 9.30–9.26 (m, 1 H, H_{ar}), 8.76–8.68 (m, 2 H, H_{ar}), 8.63 (d, ^3J = 8.8 Hz, 1 H, H_{ar}), 8.20 (d, ^3J = 8.8 Hz, 1 H, H_{ar}), 8.14–8.09 (m, 1 H, H_{ar}), 8.09–8.05 (m, 1 H, H_{ar}), 7.83–7.77 (m, 1 H, H_{ar}), 7.60–7.55 (m, 1 H, H_{ar}), 7.41–7.36 (m, 1 H, H_{ar}); ^{13}C NMR (125 MHz, DMSO-d_6, ppm): δ = 145.3 (C_q), 142.5 (C_q), 141.7 (C_q), 140.1 (C_q), 130.0 (CH), 129.7 (CH), 129.7 (CH), 128.9 (CH), 125.9 (CH), 125.4 (CH), 123.2 (C_q), 122.4 (CH), 120.1 (C_q), 119.9 (CH), 118.5 (C_q), 116.2 (CH), 112.8 (C_q), 112.6 (CH); IR (ATR, cm^{-1}): $\tilde{\nu}$ = 3109 (w), 3081 (w), 3053 (w), 3031 (w), 2955 (w), 2905 (w), 2819 (w), 2751 (w), 2742 (w), 2673 (w), 1619 (w), 1585 (w), 1560 (w), 1507 (w), 1452 (w), 1425 (w), 1375 (w), 1329 (m), 1285 (w), 1273 (m), 1264 (m), 1227 (w), 1177 (w), 1143 (w), 1111 (m), 1096 (w), 1074 (w), 1031 (w), 950 (w), 866 (w), 826 (s), 802 (w), 786 (w), 765 (s), 722 (vs); UV/Vis [THF, nm ($mol^{-1}dm^3cm^{-1}$)]: λ_{max} (ε) = 314 (41,900), 222 (57,800); fluorescence (THF, nm): λ_{ex} = 330; λ_{em} = 473; MS (FAB): *m/z* (%) = 307 (11), 271 (24) $[M+2]^+$, 270 (100) $[M+1]^+$, 269 (27) $[M]^+$, 89 (10); HRMS (FAB): *m/z* calcd. for $C_{18}H_{12}N_3^+$: 270.1026 $[M+1]^+$; found: 270.1026.

5,8-Dihydroindolo[2,3-*c*]carbazole (5)

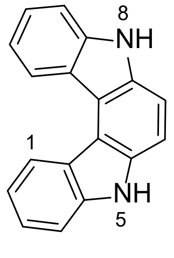

Following a published protocol,[37] azidotrimethylsilane (67.8 mg, 78 µL, 553 µmol, 1.43 equiv.) were added to a cooled (0 °C) solution of 4-(2-aminophenyl)-9*H*-carbazole (**4**, 100 mg, 387 µmol, 1.00 equiv.) and *t*BuONO (62.4 mg, 545 µmol, 1.41 equiv.) in 3 mL anhydrous MeCN. The mixture was stirred for 3 h while slowly warming to room temperature. The solvent was removed under reduced pressure, the residue was dissolved in 3 mL *o*-xylene and heated to 190 °C for 15 h. After cooling, the solvent was removed under reduced pressure. Purification by column chromatography (silica gel, hexane/EtOAc, 1:0 → 10:1 → 5:1) yielded **13** as crystalline beige solid (70.0 mg, 273 µmol, 71%). The NMR data are in agreement with published data.[18]

R_f = 0.37 (hexane/EtOAc 2:1).

3.3 Indolo[2,3-*k*]phenanthridines 3

N-[2-(9*H*-Carbazol-4-yl)phenyl]-2,2,2-trifluoroacetamide (2c)

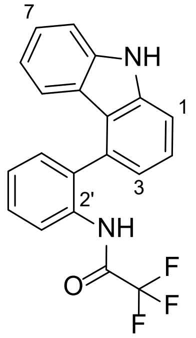

GP 2: 4-(2-Aminophenyl)-9*H*-carbazole (**1**; 99.9 mg, 387 µmol, 1.00 equiv.), 2,2,2-trifluoroacetic acid (67.8 µL, 100 mg, 880 µmol, 2.27 equiv.), pyridine (126 µL, 124 mg, 1.56 mmol, 4.04 equiv.), PPAA (≥50% w/w in MeCN; 511 mg, 803 µmol, 2.08 equiv.), MeCN/EtOAc (2:1; 3 mL); purification: silica gel, hexane/CH_2Cl_2, 1:1 and drying in high vacuum (70 °C, 4 h); **5c**: pale yellow solid (137 mg, 385 µmol, quant.).

R_f = 0.36 (hexane/CH_2Cl_2 1:1); ^{1}H NMR (400 MHz, DMSO-d$_6$, ppm): δ = 11.39 (s, 1 H, 9-NH), 10.65 (s, 1 H, NHCO), 7.73–7.45 (m, 6 H, 6×H_{ar}), 7.40 (d, ^3J = 7.70 Hz, 1 H, H_{ar}), 7.30 (t, ^3J = 7.6 Hz, 1 H, H_{ar}), 7.06 (d, ^3J = 7.9 Hz, 1 H, H_{ar}), 6.92 (d, ^3J = 7.2 Hz, 1 H, H_{ar}), 6.87 (t, ^3J = 7.5 Hz, 1 H, H_{ar}), 6.92 (d, ^3J = 7.2 Hz, 1 H, H_{ar}); ^{13}C NMR (125 MHz, DMSO-d$_6$, ppm): δ = 155.2 (q, $^2J_{CF}$ = 36.5 Hz, C_q, CO), 140.1 (C_q), 140.0 (C_q), 137.4 (C_q), 132.7 (C_q), 132.3 (C_q), 131.1 (CH), 128.5 (CH), 127.6 (CH), 127.4 (CH), 125.3 (CH), 124.9 (CH), 121.9 (C_q), 121.6 (CH), 120.1 (C_q), 119.8 (CH), 118.1 (CH), 115.7 (q, $^1J_{CF}$ = 289 Hz, C_q, CF_3), 110.8 (CH), 110.4 (CH); IR (ATR, cm^{-1}): $\tilde{\nu}$ = 3373 (m), 3065 (vw), 2923 (w), 2852 (w), 1705 (m), 1604 (w), 1588 (w), 1542 (m), 1451 (m), 1431 (w), 1390 (w), 1322 (w), 1272 (m), 1156 (m), 901 (w), 868 (w), 797 (w), 763 (m), 748 (w), 725 (m); MS (FAB): *m/z* (%) = 356 (14) $[M+2]^+$, 355 (68) $[M+1]^+$, 354 (100) $[M]^+$, 133 (25); HRMS (FAB): *m/z* calcd. for $C_{20}H_{13}F_3N_2O^+$: 354.0974 $[M]^+$; found: 354.0977.

N-[2-(9*H*-Carbazol-4-yl)phenyl]-2-chloroacetamide (2d)

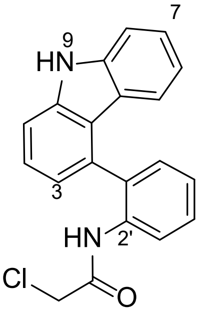

GP 2: 4-(2-Aminophenyl)-9*H*-carbazole (**1**; 201 mg, 778 µmol, 1.00 equiv.), 2-chloroacetic acid (154 mg, 1.63 mmol, 2.09 equiv.), pyridine (146 µL, 143 mg, 1.81 mmol, 2.33 equiv.), PPAA (≥50% w/w in MeCN, 1.40 g, 2.19 mmol, 2.82 equiv.), MeCN/EtOAc (1:1, 4 mL); purification: hexane/CH_2Cl_2, 1:0 → 1:2 → 1:2 + 1% Et_3N, and drying in high vacuum (50 °C, 5 h); **2d**: colorless crystalline solid (240 mg, 716 µmol, 92%).

R_f = 0.34 (hexane/CH_2Cl_2 1:2 + 1% Et_3N); ^{1}H NMR (400 MHz, DMSO-d$_6$, ppm): δ = 11.44 (s, 1 H, 9-NH), 8.86 (s, 1 H, NHCO), 8.07 (d, ^3J = 8.2 Hz, 1 H, H_{ar}), 7.59–7.51 (m, 2 H, 2×H_{ar}), 7.50–7.41 (m, 3 H, 3×H_{ar}), 7.39–7.33 (m, 1 H, H_{ar}), 7.33–7.27 (m, 1 H, H_{ar}), 7.03–6.91 (m, 2 H, 2×H_{ar}), 6.90–6.81 (m, 1 H, H_{ar}), 3.95–3.83 (m, 2 H, CH_2); ^{13}C NMR (125 MHz, DMSO-d$_6$, ppm): δ = 164.4 (CO), 140.1 (C_q), 140.0 (C_q), 134.8 (C_q), 133.1 (C_q), 131.7 (C_q), 130.4 (CH), 128.4 (CH), 125.5 (CH), 125.5 (CH), 125.2 (CH), 122.9 (CH), 121.8 (C_q), 121.0 (CH), 120.3 (C_q), 120.2 (CH), 118.3 (CH), 110.9 (CH), 110.7 (CH), 42.8 (CH_2); IR (ATR, cm^{-1}): $\tilde{\nu}$ = 3347 (w), 3053 (w), 1672 (m), 1601 (w), 1582 (m), 1525 (m), 1446 (m), 1388 (m), 1322 (m), 1262 (m), 1221 (m), 1168 (w), 1150 (w), 1114 (w), 1041 (w), 999 (w), 918 (w), 797 (w), 752 (m), 728 (m); MS (FAB): *m/z* (%) = 337 (30) $[C_{20}H_{15}{}^{37}ClN_2O+1]^+$, 336 (50) $[C_{20}H_{15}{}^{37}ClN_2O]^+$, 335 (91) $[C_{20}H_{15}{}^{35}ClN_2O+1]^+$, 334 (100) $[C_{20}H_{15}{}^{35}ClN_2O]^+$, 259 (24) $[C_{20}H_{15}{}^{37}ClN_2O-C_2H_2{}^{35}ClO]$, 258 (16) $[M+1-C_2H_2ClO]^+$, 257 (12) $[M-C_2H_2{}^{35}ClO]^+$; HRMS (FAB): *m/z* calcd. for $C_{20}H_{15}{}^{35}ClN_2O^+$: 334.0867 $[M]^+$; found: 334.0869.

N-[2-(9*H*-Carbazol-4-yl)phenyl]-2-azidoacetamide (2e)

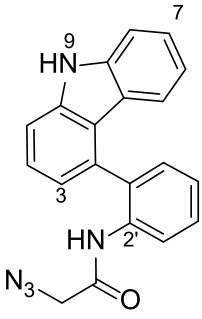

GP 2: 4-(2-Aminophenyl)-9*H*-carbazole (**1**; 90.1 mg, 349 µmol, 1.00 equiv.), 2-azidoacetic acid (65.2 mg, 645 µmol, 1.85 equiv.), pyridine (88.5 µL, 86.7 mg, 1.10 mmol, 3.14 equiv.), PPAA (≥50% w/w in MeCN; 492 mg, 773 µmol, 2.22 equiv.), EtOAc (0.5 mL); purification: silica gel, hexane/EtOAc, 6:1 → 2:1 and drying in high vacuum (30 °C, 4 h); **2e**: pale yellow crystalline solid (116 mg, 340 µmol, 97%).

R_f = 0.24 (hexane/EtOAc 3:1); ^{1}H NMR (400 MHz, DMSO-d$_6$, ppm): δ = 11.44 (s, 1 H, 9-NH), 8.83 (s, 1 H, NHCO), 8.02 (d, ^3J = 8.1 Hz, 1 H, H$_{ar}$), 7.58–7.39 (m, 5 H, 5×H$_{ar}$), 7.38–7.33 (m, 1 H, H$_{ar}$), 7.33–7.27 (m, 1 H, H$_{ar}$), 7.04–6.93 (m, 2 H, 2×H$_{ar}$), 6.90–6.82 (m, 1 H, H$_{ar}$), 3.66 (d, ^2J = 16.2 Hz, 1 H, CH$_a$*H$_b$*), 3.51 (d, ^2J = 16.1 Hz, 1 H, C*H$_a$*H$_b$); ^{13}C NMR (125 MHz, DMSO-d$_6$, ppm): δ = 166.1 (CO), 140.1 (C$_q$), 140.0 (C$_q$), 134.9 (C$_q$), 133.4 (C$_q$), 132.0 (C$_q$), 130.5 (CH), 128.3 (CH), 125.5 (CH), 125.5 (CH), 125.2 (CH), 123.6 (CH), 121.8 (C$_q$), 121.1 (CH), 120.3 (C$_q$), 120.3 (CH), 118.3 (CH), 110.9 (CH), 110.6 (CH), 51.1 (CH$_2$); IR (ATR, cm^{-1}): $\tilde{\nu}$ = 3326 (m), 3054 (w), 2916 (w), 2208 (vw), 2104 (s), 1921 (vw), 1808 (vw), 1670 (s), 1601 (m), 1582 (m), 1522 (s), 1446 (s), 1425 (m), 1388 (m), 1321 (s), 1273 (s), 1221 (m), 1041 (m), 998 (m), 866 (w), 797 (m), 752 (s), 728 (s), 655 (m), 617 (m); MS (FAB): *m/z* (%) = 343 (21) [M+2]$^+$, 342 (90) [M+1]$^+$, 341 (100) [M]$^+$, 286 (25) [M+1−CH$_2$N$_3$]$^+$, 285 (50) [M−CH$_2$N$_3$]$^+$, 269 (24), 258 (30) [M+1−C$_2$H$_2$N$_3$O]$^+$, 255 (10), 111 (14), 109 (21), 97 (30), 95 (35); HRMS (FAB): *m/z* calcd. for $C_{20}H_{15}N_5O^+$: 341.1271 [M]$^+$; found: 341.1273.

(2*E*,4*E*)-*N*-[2-(9*H*-Carbazol-4-yl)phenyl]hexa-2,4-dienamide (2g)

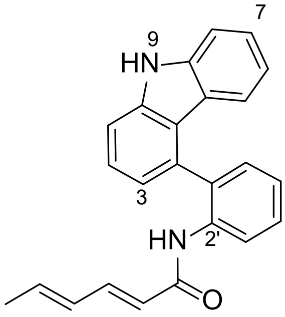

GP 2: 4-(2-Aminophenyl)-9*H*-carbazole (**1**; 101 mg, 389 µmol, 1.00 equiv.), (2*E*,4*E*)-hexa-2,4-dienoic acid (93.3 mg, 832 µmol, 2.14 equiv.), pyridine (96.3 µL, 94.4 mg, 1.19 mmol, 3.06 equiv.), PPAA (≥50% w/w in MeCN; 499 mg, 785 µmol, 2.02 equiv.), MeCN/EtOAc (1:2; 2 mL), purification: silica gel, hexane/EtOAc, 5:1 → 3:1; **2g**: yellow crystalline solid (110 mg, 311 µmol, 80%).

R_f = 0.34 (hexane/EtOAc 3:1); ^{1}H NMR (400 MHz, DMSO-d$_6$, ppm): δ = 11.41 (s, 1 H, 9-NH), 8.66 (s, 1 H, NHCO), 8.03 (d, ^3J = 8.2 Hz, 1 H, H$_{ar}$), 7.54–7.40 (m, 4 H, 4×H$_{ar}$), 7.38–7.34 (m, 1 H, H$_{ar}$), 7.32–7.25 (m, 2 H, 2×H$_{ar}$), 7.02 (d, ^3J = 8.0 Hz, 1 H, H$_{ar}$), 6.97–6.91 (m, 1 H, H$_{ar}$), 6.91–6.80 (m, 2 H, H$_{diene}$, H$_{ar}$), 6.10–5.96 (m, 2 H, H$_{diene}$), 5.77 (d, $^3J_{trans}$ = 15.1 Hz, 1 H, H$_{diene}$), 1.72 (m, 3 H, CH$_3$); ^{13}C NMR (125 MHz, DMSO-d$_6$, ppm): δ =164.2 (CO), 140.3 (CH), 140.1 (C$_q$), 139.9 (C$_q$), 137.2 (CH), 135.8 (C$_q$), 133.5 (C$_q$), 132.6 (C$_q$), 130.5 (CH), 129.7 (CH), 128.0 (CH), 125.4 (CH), 125.3 (CH), 124.6 (CH), 124.3 (CH), 122.8 (CH), 121.9 (C$_q$), 121.3 (CH), 120.4 (C$_q$), 120.3 (CH), 118.2 (CH), 110.7 (CH), 110.4 (CH), 18.3 (CH$_3$); IR (ATR, cm^{-1}): $\tilde{\nu}$ = 3379 (w), 3294 (w), 3024 (w), 2924 (w), 2906 (w), 2848 (w), 1666 (m), 1636 (m), 1615 (m), 1580 (m), 1514 (m), 1441 (m), 1339 (m), 1323 (m), 1282 (m), 1241 (w), 1139 (m), 993 (m), 930 (w), 863 (w), 799 (w), 753 (w), 728 (s); MS (FAB): *m/z* (%) = 354 (22) [M+2]$^+$, 353 (79) [M+1]$^+$, 352 (42) [M]$^+$, 259 (13) [M+2−C$_6$H$_7$O]$^+$, 258 (29) [M+1−C$_6$H$_7$O]$^+$, 257 (10) [M−C$_6$H$_7$O]$^+$, 111 (12), 109 (19), 97 (28), 95 (100); HRMS (FAB): *m/z* calcd. for $C_{24}H_{21}N_2O^+$: 353.1648 [M+1]$^+$; found: 353.1649.

***N*-[2-(9H-Carbazol-4-yl)phenyl]-4-methoxybenzamide (2i)**

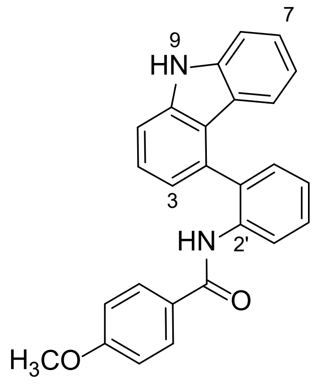

GP 1: 4-(2-Aminophenyl)-9*H*-carbazole (**1**; 90.4 mg, 350 µmol, 1.00 equiv.), 4-methoxybenzoyl chloride (61.3 µL, 67.4 mg, 395 µmol, 1.13 equiv.), Et_3N (65.5 µL, 47.8 mg, 472 µmol, 1.35 equiv.), anhydrous CH_2Cl_2 (10 mL); purification: silica gel, hexane/EtOAc, 6:1 → 3:1 and drying in high vacuum (70 °C, 6 h); **5i**: yellow crystalline solid (137 mg, 349 µmol, quant.).

R_f = 0.17 (hexane/EtOAc 3:1); ^{1}H NMR (400 MHz, DMSO-d$_6$, ppm): δ = 11.43 (s, 1 H, 9-NH), 8.90 (s, 1 H, NHCO), 8.04 (d, ^3J = 8.1 Hz, 1 H, H_{ar}), 7.59–7.41 (m, 5 H, 5×H_{ar}), 7.41–7.35 (m, 1 H, H_{ar}), 7.33–7.26 (m, 1 H, H_{ar}), 7.26–7.19 (m, 1 H, H_{ar}), 7.13 (d, ^3J = 8.0 Hz, 1 H, H_{ar}), 7.10–7.04 (m, 1 H, H_{ar}), 6.92–6.84 (m, 1 H, H_{ar}), 6.83–6.72 (m, 2 H, 2×H_{ar}), 3.71 (s, 3 H, OCH_3); ^{13}C NMR (125 MHz, DMSO-d$_6$, ppm): δ = 164.5 (CO), 161.6 (C_q), 140.1 (C_q), 140.0 (C_q), 135.8 (C_q), 134.4 (C_q), 132.6 (C_q), 130.4 (CH), 128.7 (2×CH), 128.3 (CH), 126.6 (C_q), 125.5 (CH), 125.4 (CH), 125.1 (CH), 124.5 (CH), 121.7 (C_q), 121.4 (CH), 120.2 (CH), 120.1 (C_q), 118.3 (CH), 113.5 (2×CH), 110.8 (CH), 110.4 (CH), 55.3 (OCH_3); IR (ATR, cm^{-1}): $\tilde{\nu}$ = 3403 (w), 3252 (w), 3055 (w), 2960 (w), 2928 (w), 2836 (w), 1654 (m), 1602 (m), 1602 (m), 1580 (m), 1524 (m), 1500 (m), 1440 (m), 1302 (m), 1246 (m), 1172 (m), 1116 (m), 1023 (m), 893 (w), 844 (w), 797 (w), 753 (m), 729 (m); MS (FAB): *m/z* (%) = 394 (9) [M+2]$^+$, 393 (34) [M+1]$^+$, 392 (29) [M]$^+$, 135 (100) [3-NBA–H_2O]; HRMS (FAB): *m/z* calcd. for $C_{26}H_{21}N_2O_2^+$: 393.1598 [M+1]$^+$; found: 393.1598.

***N*-[2-(9*H*-Carbazol-4-yl)phenyl]-4-(trifluoromethyl)benzamide (2j)**

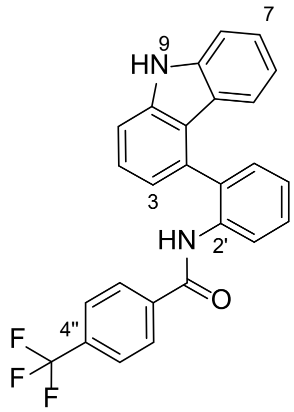

GP 1: 4-(2-Aminophenyl)-9*H*-carbazole (**1**; 90.3 mg, 350 µmol, 1.00 equiv.), 4-(trifluoromethyl)benzoyl chloride (85.8 µL, 94.4 mg, 453 µmol, 1.30 equiv.), Et_3N (64.7 µL, 47.2 mg, 466 µmol, 1.33 equiv.), anhydrous CH_2Cl_2 (10 mL); purification: silica gel, hexane/EtOAc, 6:1 → 3:1 and drying in high vacuum (70 °C, 6 h); **2j**: yellow crystalline solid (148 mg, 343 µmol, 98%).

R_f = 0.52 (hexane/EtOAc 3:1); ^{1}H NMR (400 MHz, $CDCl_3$, ppm): δ = 8.71 (d, ^3J = 8.3 Hz, 1 H, H_{ar}), 8.39 (s, 1 H, 9-NH), 7.97 (s, 1 H, NHCO), 7.63–7.58 (m, 1 H, H_{ar}), 7.57–7.54 (m, 2 H, 2×H_{ar}), 7.52 (dd, ^3J = 7.6 Hz, ^4J = 1.6 Hz, 1 H, H_{ar}), 7.48–7.44 (m, 1 H, H_{ar}), 7.41–7.33 (m, 4 H, 4×H_{ar}), 7.24 (d, ^3J = 8.1 Hz, 1 H, H_{ar}), 7.18 (q, ^2J = 4.4 Hz, 1 H, H_{ar}), 7.14–7.07 (m, 2 H, 2×H_{ar}), 7.05–6.98 (m, 1 H, H_{ar}); ^{13}C NMR (125 MHz, $CDCl_3$, ppm): δ = 164.0 (CO), 139.9 (C_q), 139.8 (C_q), 138.3 (C_q), 135.5 (C_q), 133.1 (q, ^2J = 32.7 Hz, C_q, C-4”), 131.7 (C_q), 131.0 (C_q), 130.3 (CH), 129.3 (CH), 127.0 (2×CH), 126.7 (CH), 126.6 (CH), 125.7-125.5 (m, 2×CH), 124.9 (CH), 123.6 (q, ^1J = 273 Hz, C_q, CF_3), 122.1 (C_q), 122.0 (CH), 121.7 (CH), 121.0 (C_q), 120.5 (CH), 120.2 (CH), 111.0 (CH), 110.7 (CH); IR (ATR, cm^{-1}): $\tilde{\nu}$ = 3390 (w), 3286 (w), 3054 (w), 2919 (w), 1671 (m), 1601 (w), 1583 (m), 1527 (m), 1450 (m), 1405 (w), 1321 (m), 1161 (m), 1126 (m), 1064 (m), 1015 (m), 934 (w), 895 (w), 856 (m), 796 (w), 766 (w), 749 (m), 730 (m); MS (FAB): *m/z* (%) = 432 (25) [M+2]$^+$, 431 (100) [M+1]$^+$, 430 (99) [M]$^+$, 307 (10), 173 (64); HRMS (FAB): *m/z* calcd. for $C_{26}H_{17}F_3N_2O^+$: 430.1287 [M]$^+$; found: 430.1287.

***N*-(2-(9*H*-carbazol-4-yl)phenyl)picolinamide (2l)**

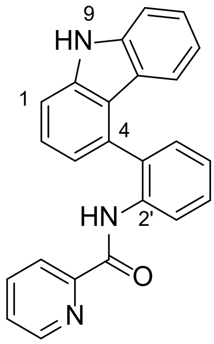

Following a published protocol,[32] triphenylphosphite (442 mg, 1.42 mmol, 1.00 equiv.) were added at 100 °C to a solution of 4-(2-aminophenyl)-9*H*-carbazole (**1**; 366 mg, 1.42 mmol, 1.00 equiv.) and pyridine-2-carboxylic acid (196 mg, 1.59 mmol, 1.12 equiv.) in 20 mL anhydrous pyridine and stirred at 100 °C for 18 h. After cooling down, half saturated NaCl solution (20 mL) and CH_2Cl_2 (50 mL) were added. The layers were separated, and the aqueous layer was extracted with CH_2Cl_2 (3×20 mL). The combined organic layers were dried ($MgSO_4$), concentrated under reduced pressure, and purified by column chromatography (silica gel, hexane/EtOAc 1:0 →10:1 → 4:1 → 1:1) to yield **2l** crystalline solid (537 mg), which contained minor impurities.

R_f = 0.15 (hexane/EtOAc 3:1); ^{1}H NMR (500 MHz, DMSO-d$_6$, ppm): δ = 11.51 (s, 1 H, 9-NH), 10.02 (s, 1 H, NHCO), 8.66 (dd, ^3J = 8.3 Hz, ^4J = 1.2 Hz, 1 H, H_{ar}), 8.05–8.00 (m, 1 H, H_{ar}), 7.98 (dt, ^3J = 7.9 Hz, ^4J = 1.1 Hz, 1 H, H_{ar}), 7.88 (td, ^3J = 7.7 Hz, ^4J = 1.7 Hz, 1 H, H_{ar}), 7.64 (dd, ^3J = 8.2 Hz, ^4J = 1.0 Hz, 1 H, H_{ar}), 7.63–7.58 (m, 1 H, H_{ar}), 7.58–7.52 (m, 1 H, H_{ar}), 7.47 (dd, ^3J = 7.5 Hz, ^4J = 1.6 Hz, 1 H, H_{ar}), 7.45–7.42 (m, 1 H, H_{ar}), 7.43–7.38 (m, 1 H, H_{ar}), 7.36 (td, ^3J = 7.5 Hz, ^4J = 1.2 Hz, 1 H, H_{ar}), 7.25 (ddd, ^3J = 8.1 Hz, ^3J = 7.1 Hz, ^4J = 1.2 Hz, 1 H, H_{ar}), 7.11 (dd, ^3J = 7.2 Hz, ^4J = 0.9 Hz, 1 H, H_{ar}), 6.95–6.91 (m, 1 H, H_{ar}), 6.85–6.80 (m, 1 H, H_{ar}); ^{13}C NMR (125 MHz, DMSO-d$_6$, ppm): δ = 161.2 (CO), 148.7 (C_q), 147.9 (CH), 140.2 (C_q), 140.0 (C_q), 138.2 (CH), 135.3 (C_q), 131.2 (C_q), 131.1 (C_q), 130.1 (CH), 128.8 (CH), 126.8 (CH), 125.9 (CH), 125.5 (CH), 124.3 (CH), 121.7 (CH), 121.7 (C_q), 120.7 (CH), 120.3 (C_q), 120.2 (CH), 119.7 (CH), 118.4 (CH), 111.0 (CH), 110.9 (CH); IR (ATR, cm^{-1}): $\tilde{\nu}$ = 3405 (vw), 3281 (w), 3054 (w), 1737 (vw), 1669 (m), 1601 (w), 1680 (m), 1520 (m), 1448 (m), 1430 (m), 1322 (m), 1221 (w), 1150 (w), 1118 (w), 1040 (w), 998 (w), 898 (w), 814 (vw), 797 (w), 747 (m), 728 (m); MS (ESI): *m/z* (%) = 575 (14), 574 (47), 564 (16), 521 (11), 520 (32), 520 (13), 492 (18), 472 (25), 471 (100), 458 (17), 448 (26), 446 (41), 424 (17), 410 (22), 406 (11), 376 (30), 364 (89) [M+1]$^+$, 327 (12), 282 (13), 221 (11), 134 (23), 109 (18), 100 (65). HRMS (ESI): *m/z* calcd. for $C_{24}H_{18}N_3O^+$: 364.1444 [M+1]$^+$, found: 364.1440.

***N*-(2-(9*H*-carbazol-4-yl)phenyl)-1-naphthamide (2n)**

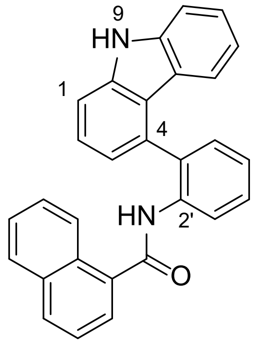

GP 1: 4-(2-Aminophenyl)-9*H*-carbazole (**1**; 400 mg, 1.55 mmol, 1.00 equiv.), 1-naphthoic acid (445 mg, 2.33 mmol, 1.51 equiv.), Et_3N (238 mg, 326 µL, 2.35 mmol, 1.52 equiv.), anhydrous CH_2Cl_2 (20 mL); purification: silica gel, hexane/CH_2Cl_2, 1:0 → 1:1 → 1:3; **2n**: colorless crystalline solid, containing minor impurities (638 mg, 1.55 mmol, quant.).

R_f = 0.34 (hexane/CH_2Cl_2 1:2); ^{1}H NMR (400 MHz, DMSO-d$_6$, ppm): δ = 11.38 (s, 1 H, 9-NH), 9.45 (s, 1 H, NHCO), 7.99 (m, 1 H, H_{ar}), 7.87–7.79 (m, 2 H, 2×H_{ar}), 7.64–7.58 (m, 1 H, H_{ar}), 7.54–7.39 (m, 7 H, 7×H_{ar}), 7.35–7.29 (m, 1 H, H_{ar}), 7.29–7.24 (m, 1 H, H_{ar}), 7.24–7.19 (m, 1 H, H_{ar}), 7.16 (d, ^3J = 8.0 Hz, 1 H, H_{ar}), 7.09 (dd, ^3J = 7.2 Hz, ^4J = 1.0 Hz, 1 H, H_{ar}), 6.98 (dd, ^3J = 7.0 Hz, ^4J = 1.2 Hz, 1 H, H_{ar}), 6.94–6.89 (m, 1 H, H_{ar}); ^{13}C NMR (125 MHz, DMSO-d$_6$, ppm): δ = 167.2 (CO), 140.0 (C_q), 140.0 (C_q), 136.0 (C_q), 135.8 (C_q), 134.7 (C_q), 133.2 (C_q), 132.8 (C_q), 130.5 (CH), 129.6 (CH), 129.4 (C_q), 128.2 (CH), 127.9 (CH), 126.4 (CH), 126.1 (CH), 126.0 (CH), 125.9 (CH), 125.4 (CH), 125.2 (CH), 125.1 (CH), 124.7 (CH), 124.6 (CH), 122.1 (C_q), 121.7 (CH),

120.5 (CH), 120.3 (C_q), 118.3 (CH), 110.7 (CH), 110.2 (CH); IR (ATR, cm^{-1}): $\tilde{\nu}$ = 3388 (vw), 3284 (w), 3052 (vw), 2920 (vw), 2846 (vw), 1895 (vw), 1730 (vw), 1655 (w), 1579 (w), 1515 (m), 1442 (m), 1391 (w), 1322 (w), 1303 (w), 1244 (w), 1141 (w), 1042 (w), 999 (w), 896 (w), 797 (w), 778 (m), 754 (m), 728 (m); MS (ESI): *m/z* (%) = 461 (10), 413 (21) $[M+1]^+$, 412 (5) $[M]^+$, 287 (6) $[M+2- C_{10}H_7^{\cdot}]^+$, 285 (17) $[M-C_{10}H_7^{\cdot}]^+$, 277 (30), 282 (15), 278 (6), 204 (13), 203 (100), 158 (12), 145 (21), 122 (31), 100 (7); HRMS (ESI): *m/z* calcd. for $C_{29}H_{21}N_2O^+$: 413.1648 $[M+1]^+$, found: 413.1646.

6-(Trifluoromethyl)-9*H*-indolo[2,3-*k*]phenanthridine (3c)

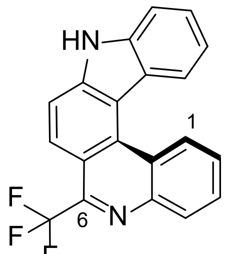

GP 3: *N*-[2-(9*H*-Carbazol-4-yl)phenyl]-2,2,2-trifluoroacetamide (**2c**; 58.7 mg, 166 µmol, 1.00 equiv.), $POCl_3$ (103 mg, 62 µL, 674 µmol, 4.07 equiv.), $PhNO_2$ (10 mL); 150 °C, 65.5 h; two-fold purification: silica gel, hexane/EtOAc, 1:0 → 1:1, and drying in high vacuum (70 °C); **3c**: redbrown crystalline solid, containing minor impurities (7.6 mg, 23 µmol, 14%).

R_f = 0.29 (hexane /EtOAc 4:1); ^{1}H NMR (400 MHz, DMSO-d$_6$, ppm): δ = 12.55 (s, 1 H, NH), 9.31–9.26 (m, 1 H, H_{ar}), 8.66 (d, ^3J = 8.2 Hz, 1 H, H_{ar}), 8.34–8.29 (m, 2 H, 2×H_{ar}), 8.13 (d, ^3J = 9.0 Hz, 1 H, H_{ar}), 7.99–7.92 (m, 2 H, 2×H_{ar}), 7.81–7.76 (m, 1 H, H_{ar}), 7.62–7.52 (m, 1 H, H_{ar}), 7.37–7.31 (m, 1 H, H_{ar}); ^{13}C NMR (125 MHz, DMSO-d$_6$, ppm): δ = 145.2 (q, $^2J_{CF}$ = 31 Hz, C_q-CF_3), 141.9 (C_q), 141.9 (C_q), 140.0 (C_q), 132.0 (C_q), 130.0 (CH), 129.8 (CH), 127.6 (CH), 126.5 (CH), 126.2 (CH), 124.1 (C_q), 122.6 (CH), 122.5 (C_q), 122.3 (d, CH), 122.5 (q, $^1J_{CF}$ = 277 Hz, CF_3)*, 119.6 (CH), 116.0 (C_q), 115.1 (CH), 114.8 (C_q), 112.4 (CH); IR (ATR, cm^{-1}): $\tilde{\nu}$ = 3165 (w), 2927 (w), 2851 (w), 1723 (w), 1584 (w), 1530 (w), 1459 (w), 1378 (w), 1312 (w), 1248 (m), 1117 (m), 1076 (m), 1022 (m), 982 (m), 869 (w), 819 (w), 801 (w), 746 (m), 711 (m); MS (FAB): *m/z* (%) = 338 (24) $[M+2]^+$, 337 (100) $[M+1]^+$, 336 (43) $[M]^+$, 95 (12); HRMS (FAB): *m/z* calcd. for $C_{20}H_{12}F_3N_2^+$: 337.0947 $[M+1]^+$; found: 337.0948. *Not all peaks of the quartet signal are visible.

6-(Chloromethyl)-9*H*-indolo[2,3-*k*]phenanthridine (3d)

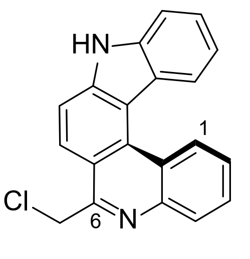

GP 3: *N*-(2-(9*H*-Carbazol-4-yl)phenyl)-2-chloroacetamide (**2d**; 226 mg, 675 µmol, 1.00 equiv.), $POCl_3$ (94.5 µL, 159 mg, 1.04 mmol, 1.53 equiv.), $PhNO_2$ (3 mL), 150 °C, 62 h; purification: hexane/EtOAc, 1:0 → 1:1 → EtOAc/CH_2Cl_2, 1:1 + 10% MeOH; **3d**: yellow crystalline solid (114 mg, 359 µmol, 53%).

R_f = 0.16 (hexane/EtOAc 4:1); ^{1}H NMR (400 MHz, DMSO-d$_6$, ppm): δ = 12.56 (s, 1 H, NH), 9.22 (d, ^3J = 8.2 Hz, 1 H, H_{ar}), 8.65 (d, ^3J = 8.2 Hz, 1 H, H_{ar}), 8.47 (d, ^3J = 8.8 Hz, 1 H, H_{ar}), 8.22 (d, ^3J = 8.1 Hz, 1 H, H_{ar}), 8.07 (d, ^3J = 8.8 Hz, 1H, H_{ar}), 7.90 (t, ^3J = 7.5 Hz, 1 H, H_{ar}), 7.84 (t, ^3J = 7.8 Hz, 1 H, H_{ar}), 7.76 (d, ^3J = 8.1 Hz, 1 H, H_{ar}), 7.55 (t, ^3J = 7.6 Hz, 1 H, H_{ar}), 7.33 (t, ^3J = 7.6 Hz, 1 H, H_{ar}), 5.49 (s, 2 H, CH_2); ^{13}C NMR (125 MHz, DMSO-d$_6$, ppm): δ = 155.9 (C_q), 143.4 (C_q), 141.9 (C_q), 140.0 (C_q), 131.2 (C_q), 129.2 (CH), 128.7 (CH), 126.4 (CH), 125.8 (CH), 125.8 (CH), 124.3 (CH), 123.5 (C_q), 122.6 (C_q), 122.5 (CH), 119.3 (CH), 118.8 (C_q), 114.8 (C_q), 114.1 (CH), 112.3 (CH), 46.4 (CH_2); IR (ATR, cm^{-1}): $\tilde{\nu}$ = 3138 (vw), 2958 (vw), 2921 (w), 2849 (w), 1582 (w), 1526 (w), 1481 (vw), 1455 (w), 1364 (w), 1254 (w), 1190 (w), 1142 (w), 930 (vw), 852 (vw), 800 (w), 770 (w), 729 (m); UV/Vis [THF, nm ($mol^{-1}dm^3cm^{-1}$)]: λ_{max} (ε) = 308 (41,000),

250 (24,700), 225 (52,000); fluorescence (THF, nm): λ_{ex} = 330; λ_{em} = 417, 399, 382; MS (FAB): *m/z* (%) = 320 (10), 319 (37), 318 (36) $[M+2]^+$, 317 (100) $[M+1]^+$, 316 (17) $[M]^+$, 297 (13), 283 (34) $[M+2-^{35}Cl]^+$, 281 (48) $[M-^{35}Cl]^+$, 280 (19), 267 (15), 241 (12), 207 (13), 193 (17), 167 (12), 165 (18), 109 (49), 97 (55), 95 (85); HRMS (FAB): *m/z* calcd. for $C_{20}H_{14}{}^{35}ClN_2{}^+$: 317.0840 $[M+1]^+$; found: 317.0842.

6-(Azidomethyl)-9*H*-indolo[2,3-*k*]phenanthridine (3e)

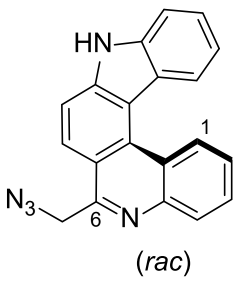

(*rac*)

Following a published protocol,[34] Tf_2O (156 µL, 261 mg, 925 µmol, 3.00 equiv.) was added dropwise to a cooled (0 °C) solution of Ph_3PO (130 mg, 469 µmol, 1.52 equiv.) in anhydrous CH_2Cl_2 (3 mL) and the mixture was stirred at 0 °C for 15 min. A solution of *N*-[2-(9*H*-carbazol-4-yl)phenyl]-2-azidoacetamide (**2e**; 105 mg, 308 µmol, 1.00 equiv.) in anhydrous CH_2Cl_2 (5 mL) was added dropwise and the mixture was stirred at 0 °C for 1 h, warmed to room temperature, and stirred until full conversion (TLC, 2.5 h). Saturated aqueous $NaHCO_3$ solution (15 mL) was added and the mixture was stirred for 10 min. The layers were separated and the aqueous layer was extracted with CH_2Cl_2 (3×20 mL). The combined organic layers were dried ($MgSO_4$), concentrated under reduced pressure, and purified by column chromatography (silica gel, hexane/EtOAc 3:1 → 1:1). Traces of solvent were removed in high vacuum (30 °C, 6 h) to yield **3e** (76.8 mg, 238 µmol, 77%) as an orange crystalline solid.

R_f = 0.31 (hexane/EtOAc 1:1); ^{1}H NMR (400 MHz, DMSO-d$_6$, ppm): δ = 12.33 (s, 1 H, NH), 9.22 (d, ^3J = 8.2 Hz, 1 H, H_{ar}), 8.67 (d, ^3J = 8.2 Hz, 1 H, H_{ar}), 8.30 (d, ^3J = 8.8 Hz, 1 H, H_{ar}), 8.17 (dd, ^3J = 8.1 Hz, ^4J = 1.4 Hz, 1 H, H_{ar}), 8.00 (d, ^3J = 8.8 Hz, 1 H, H_{ar}), 7.90–7. 82 (m, 1 H, H_{ar}), 7.84–7.75 (m, 1 H, H_{ar}), 7.75 (d, ^3J = 8.1 Hz, 1 H, H_{ar}), 7.58–7.50 (m, 1 H, H_{ar}), 7.36–7.28 (m, 1 H, H_{ar}), 5.19 (s, 2 H, CH_2); ^{13}C NMR (125 MHz, DMSO-d$_6$, ppm): δ = 155.3 (C_q), 143.7 (C_q), 141.8 (C_q), 140.0 (C_q), 130.8 (C_q), 129.1 (CH), 129.0 (CH), 126.4 (CH), 125.8 (CH), 125.4 (CH), 123.6 (CH), 123.4 (C_q), 122.7 (C_q), 122.5 (CH), 119.2 (C_q), 119.0 (CH), 114.8 (C_q), 114.0 (CH), 112.2 (CH), 53.7 (CH_2); IR (ATR, cm^{-1}): $\tilde{\nu}$ = 2922 (w), 2096 (w), 1703 (vw), 1582 (w), 1525 (w), 1480 (vw), 1456 (w), 1365 (w), 1324 (w), 1264 (w), 1188 (w), 1152 (w), 1123 (w), 1033 (vw), 935 (vw), 854 (vw), 798 (w), 772 (w), 744 (m); UV/Vis [THF, nm (mol^{-1}dm^{3}cm^{-1})]: λ_{max} (ε)= 307 (35,000), 248 (16,600), 225 (36,700); fluorescence (THF, nm): λ_{ex} = 330; λ_{em} = 467, 423, 400; MS (FAB): *m/z* (%) = 419 (14), 418 (44), 325 (15) $[M+2]^+$, 324 (61) $[M+1]^+$, 323 (17) $[M]^+$, 297 (20) $[M+2-N_2]^+$, 296 (29) $[M+1-N_2]^+$, 295 (14) $[M-N_2]^+$, 268 (18) $[M-CH_2N_3{}^{\cdot}]^+$, 267 (15), 266 (10), 154 (100) [3-NBA], 120 (13), 90 (15); HRMS (FAB): *m/z* calcd. for $C_{20}H_{14}N_5{}^+$: 324.1244 $[M+1]^+$; found: 324.1243.

6-[(1*E*,3*E*)-Penta-1,3-dien-1-yl]-9*H*-indolo[2,3-*k*]phenanthridine (3g)

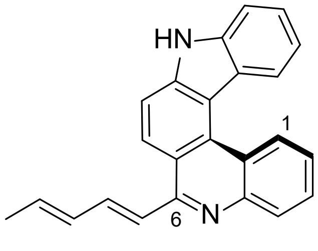

(*rac*)

GP 3: (2*E*,4*E*)-*N*-[2-(9*H*-Carbazol-4-yl)phenyl]hexa-2,4-dienamide (**2g**; 107 mg, 305 µmol, 1.00 equiv.), $POCl_3$ (47.5 µL, 79.8 mg, 520 µmol, 1.71 equiv.), $PhNO_2$ (3 mL), 150 °C, 3.5 h; purification: silica gel, hexane/CH_2Cl_2, 4:1 → 1:1 and silica gel, hexane/EtOAc, 0:1 → 5:1 → 1:1, and drying in high vacuum (70 °C, 7 h); **3g**: yellow crystalline solid (37.4 mg, 112 µmol, 37%).

R_f = 0.22 (hexane/CH_2Cl_2 1:1 + 1% MeOH); ^{1}H NMR (400 MHz, DMSO-d$_6$, ppm): δ = 12.26 (s, 1 H, NH), 9.13 (d, ^3J = 8.5 Hz, 1 H, H_{ar}), 8.64 (d, ^3J = 8.2 Hz, 1 H, H_{ar}), 8.52 (d, ^3J = 9.0 Hz, 1 H, H_{ar}), 8.11 (dd, ^3J = 8.2 Hz, ^4J = 1.3 Hz, 1 H, H_{ar}), 7.96 (d, ^3J = 8.9 Hz, 1 H, H_{ar}), 7.83–7.76 (m, 1 H, H_{ar}), 7.76–7.61 (m, 4 H, 4×H_{ar}), 7.56–7.48 (m, 1 H, H_{alkene}), 7.34–7.26 (m, 1 H, H_{alkene}), 6.62–6.50 (m, 1 H, H_{alkene}), 6.19 (m, 1 H, H_{alkene}), 1.97–1.80 (m, 3 H, CH_3); ^{13}C NMR (125 MHz, DMSO-d$_6$, ppm): Analysis of the data was not pursued due to the presence of trace impurities and a possible occurrence of rotamers; IR (ATR, cm^{-1}): $\tilde{\nu}$ = 3057 (w), 2915 (w), 2848 (w), 1720 (vw), 1638 (w), 1589 (w), 1511 (w), 1478 (w), 1454 (m), 1364 (m), 1322 (w), 1273 (m), 1154 (w), 1035 (w), 978 (m), 867 (w), 798 (w), 769 (w), 738 (m); UV/Vis [THF, nm (mol^{-1}dm^{3}cm^{-1})]: λ_{max} (ε) = 326 (44,600), 316 (48,500), 270 (29,900), 217 (40,800); fluorescence (THF, nm): λ_{ex} = 330; λ_{em} = 398, 386, 365; MS (FAB): *m/z* (%) = 353 (10), 336 (31) [M+2]$^+$, 335 (100) [M+1]$^+$, 334 (17) [M]$^+$, 307 (13), 281 (20), 267 (13) [M–C_5H_7]$^+$, 258 (10), 207 (14), 121 (27), 109 (48), 95 (80); HRMS (FAB): *m/z* calcd. for $C_{24}H_{19}N_2^+$: 335.1543 [M+1]$^+$; found: 335.1545.

6-Phenyl-9*H*-indolo[2,3-*k*]phenanthridine (3h)

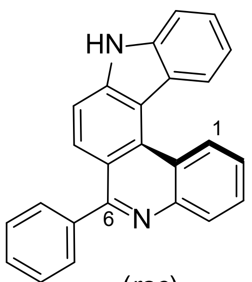

(*rac*)

According to a published protocol,[35] 4-(2-aminophenyl)-9*H*-carbazole (**1**; 100 mg, 387 mmol, 1.00 equiv.), benzaldehyde (130 mg, 1.23 mmol, 3.16 equiv.) and *p*-TosOH·H_2O (9.6 mg, 50 µL, 0.13 equiv.) in 10 mL anhydrous *o*-dichloroethane were heated under argon-atmosphere to 85 °C for 17.5 h. After cooling down, sat. $NaHCO_3$-Lösung (10 mL) were added and the layers were separated. The aqueous layer was extracted with CH_2Cl_2 (3 × 20 mL). The combined organic layers were dried ($MgSO_4$), concentrated under reduced pressure, and purified by column chromatography (silica gel, hexane/EtOAc, 4:1 → 2:1 → 2:1 + 1% MeOH). **3h** was obtained as beige solid (42.3 mg, 123 µmol, 32%).

m.p. 282–283 °C; R_f = 0.56 (hexane/CH_2Cl_2 1:1); ^{1}H NMR (400 MHz, DMSO-d$_6$, ppm): δ = 12.33 (s, 1 H, NH), 9.25 (d, ^3J = 8.1 Hz, ^4J = 1.4 Hz, 1 H, H_{ar}), 8.70 (d, ^3J = 8.2 Hz, 1 H, H_{ar}), 8.17 (d, ^3J = 8.2 Hz, ^4J = 1.4 Hz, 1 H, H_{ar}), 7.98 (d, ^3J = 8.8 Hz, 1 H, H_{ar}), 7.91 (d, ^3J = 8.8 Hz, 1 H, H_{ar}), 7.88–7.84 (m, 1 H, H_{ar}), 7.82–7.76 (m, 1 H, H_{ar}), 7.74 (d, ^3J = 8.1 Hz, 1 H, H_{ar}), 7.74–7.68 (m, 2 H, 2×H_{ar}), 7.64–7.57 (m, 3 H, 3×H_{ar}), 7.53 (t, ^3J = 8.1 Hz, 1 H, H_{ar}), 7.37–7.30 (m, 1 H, H_{ar}); ^{13}C NMR (100 MHz, DMSO-d$_6$, ppm): δ = 160.6 (C_q), 144.3 (C_q), 141.7 (C_q), 140.4 (C_q), 140.0 (C_q), 131.1 (C_q), 129.9 (2×CH), 129.1 (CH), 129.0 (CH), 128.5 (CH), 128.2 (2×CH), 126.2 (CH), 126.1 (CH), 125.7 (CH), 125.0 (CH), 122.9 (C_q), 122.8 (C_q), 122.5 (CH), 119.7 (C_q), 119.2 (CH), 114.7 (C_q), 113.6 (CH), 112.2 (CH); IR (ATR, cm^{-1}): $\tilde{\nu}$ = 2747 (w), 1581 (w), 1525 (w), 1477 (w), 1454 (w), 1359 (m), 1275 (m), 1188 (w), 1147 (w), 1131 (w), 874 (w), 823 (w), 803 (w), 769 (w), 745 (m), 705 (m); UV/Vis [THF, nm (mol^{-1}dm^{3}cm^{-1})]: λ_{max} (ε)= 307 (47,600), 257 (31,100), 224 (52,900); fluorescence (THF, nm): λ_{ex} = 330; λ_{em} = 404, 365; MS (FAB): *m/z* (%) = 347 (27) [M+3]$^+$, 346 (45) [M+2]$^+$, 345 (100) [M+1]$^+$, 344 (35) [M]$^+$, 343 (17), 283 (16); HRMS (FAB): *m/z* calcd. for $C_{25}H_{17}N_2^+$: 345.1386 [M+1]$^+$; found: 345.1387.

6-(4-Methoxyphenyl)-9*H*-indolo[2,3-*k*]phenanthridine (3i)

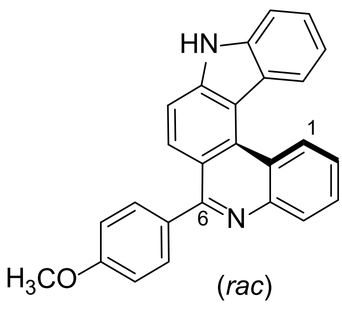

GP 3: *N*-[2-(9*H*-Carbazol-4-yl)phenyl]-4-methoxybenzamide (**2i**; 103 mg, 263 µmol, 1.00 equiv.), $POCl_3$ (42.2 µL, 70.9 mg, 462 µmol, 1.76 equiv.), $PhNO_2$ (3 mL), 150 °C, 3.5 h; purification: silica gel, hexane/CH_2Cl_2, 1:0 → 1:1 → 1:1 + 2% MeOH and silica gel, hexane/CH_2Cl_2, 1:0 → 1:1 → 0:1 + 0.3% MeOH; **3i**: pale yellow solid (74.8 mg, 200 µmol, 76%).

R_f = 0.20 (hexane/CH_2Cl_2 1:1 + 2% MeOH); ^{1}H NMR (400 MHz, DMSO-d_6, ppm): δ = 12.31 (s, 1 H, NH), 9.22 (d, ^3J= 8.1 Hz, 1 H, H_{ar}), 8.69 (d, ^3J = 8.1 Hz, 1 H, H_{ar}), 8.81–8.13 (m, 1 H, H_{ar}), 8.05 (d, ^3J = 1 H, H_{ar}), 7.90 (d, ^3J = 8.8 Hz, 1 H, H_{ar}), 7.88–7.80 (m, 1 H, H_{ar}), 7.78–7.73 (m, 2 H, 2×H_{ar}), 7.69–7.62 (m, 2 H, 2×H_{ar}), 7.56–7.49 (m, 1 H, H_{ar}), 7.36–7.28 (m, 1 H, H_{ar}), 7.18–7.12 (m, 2 H, 2×H_{ar}), 3.88 (s, 3 H, OCH_3); ^{13}C NMR (125 MHz, DMSO-d_6, ppm): δ = 160.2 (C_q), 159.6 (C_q), 144.4 (C_q), 141.7 (C_q), 140.0 (C_q), 132.7 (C_q), 131.4 (2×CH), 131.2 (C_q), 129.0 (CH), 129.0 (CH), 126.3 (CH), 126.2 (CH), 125.7 (CH), 124.8 (CH), 122.8 (2×C_q), 122.5 (CH), 119.8 (C_q), 119.1 (CH), 114.7 (C_q), 113.6 (2×CH), 113.4 (CH), 112.2 (CH), 55.2 (OCH_3); IR (ATR, cm^{-1}): $\tilde{\nu}$ = 2951 (w), 2913 (w), 2832 (w), 1592 (w), 1509 (w), 1452 (w), 1356 (m), 1300 (w), 1279 (w), 1245 (m), 1174 (w), 1148 (w), 1032 (w), 972 (w), 838 (w), 803 (w), 764 (w), 743 (m), 730 (m); UV/Vis [THF, nm (mol^{-1}dm^{3}cm^{-1})]: λ_{max} (ε)= 307 (57,500), 274 (29,500), 268 (29,700), 226 (67,300); fluorescence (THF, nm): λ_{ex} = 330; λ_{em} = 408, 365; MS (FAB): *m/z* (%) = 376 (34) [M+2]$^+$, 375 (100) [M+1]$^+$, 374 (20) [M]$^+$, 373 (13), 217 (17), 97 (19), 95 (29); HRMS (FAB): *m/z* calcd. for $C_{26}H_{19}N_2O^+$: 375.1492 [M+1]$^+$; found: 375.1491.

6-[4-(Trifluoromethyl)phenyl]-9*H*-indolo[2,3-*k*]phenanthridine (3j)

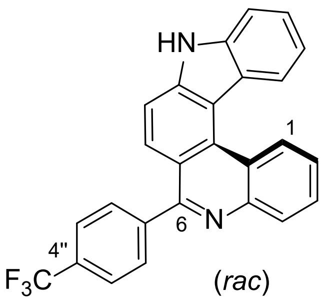

GP 3: *N*-[2-(9*H*-Carbazol-4-yl)phenyl]-4-(trifluoromethyl)benzamide (**2j**; 122 mg, 282 µmol, 1.00 equiv.), $POCl_3$ (54.3 µL, 91.3 mg, 595 µmol, 2.11 equiv.), $PhNO_2$ (3 mL), 150 °C, 3 h; purification: silica gel, hexane/CH_2Cl_2, 1:1 → 1:2; **3j**: yellow solid (108 mg, 263 µmol, 93%).

R_f = 0.26 (hexane/EtOAc 1:2); ^{1}H NMR (500 MHz, DMSO-d_6, ppm): δ = 12.35 (s, 1 H, NH), 9.25 (d, ^3J = 8.3 Hz, 1 H, H_{ar}), 8.70 (d, ^3J = 8.2 Hz, 1 H, H_{ar}), 8.22–8.13 (m, 1 H, H_{ar}), 7.97–7.90 (m, 4 H, 4×H_{ar}), 7.90–7.88 (m, 2 H, 2×H_{ar}), 7.88–7.83 (m, 1 H, H_{ar}), 7.81–7.77 (m, 1 H, H_{ar}), 7.75 (d, ^3J = 8.1 Hz, 1 H, H_{ar}), 7.57–7.51 (m, 1 H, H_{ar}), 7.36–7.29 (m, 1 H, H_{ar}); ^{13}C NMR (125 MHz, DMSO-d_6, ppm): δ = 159.2 (C_q), 144.4 (C_q), 144.1 (C_q), 141.8 (C_q), 140.0 (C_q), 131.0 (C_q), 130.7 (2×CH), 129.2 (CH), 129.2 (CH), 128.9 (q, ^2J = 32 Hz, C-4''), 126.3 (CH), 125.8 (CH), 125.8 (CH), 125.3 (CH), 125.2 (q, ^3J = 3.7 Hz, 2×CH), 124.3 (q, ^1J = 272 Hz, CF_3), 123.1 (C_q), 122.8 (C_q), 122.5 (CH), 119.4 (C_q), 119.3 (CH), 114.7 (C_q), 113.8 (CH), 112.2 (CH); IR (ATR, cm^{-1}): $\tilde{\nu}$ = 3059 (w), 2917 (w), 2833 (w), 1616 (w), 1582 (w), 1523 (w), 1455 (w), 1408 (w), 1361 (w), 1320 (m), 1164 (m), 1127 (m), 1104 (m), 1061 (m), 1016 (m), 973 (w), 860 (w), 840 (w), 803 (w), 767 (w), 744 (m); UV/Vis [THF, nm (mol^{-1}dm^{3}cm^{-1})]: λ_{max} (ε)= 309 (46,900), 260 (32,600), 228 (50,400), 206 (10,600); fluorescence (THF, nm): λ_{ex} = 330; λ_{em} = 434, 365; MS (FAB): *m/z* (%) = 414 (30) [M+2]$^+$, 413 (100) [M+1]$^+$, 412 (32) [M]$^+$, 411 (13), 307 (22), 289 (12), 120 (11), 90 (18); HRMS (FAB): *m/z* calcd. for $C_{26}H_{16}F_3N_2^+$: 412.1260 [M+1]$^+$; found: 413.1261.

6-(Pyridine-2-yl)-9*H*-indolo[2,3-*k*]phenanthridine (3l)

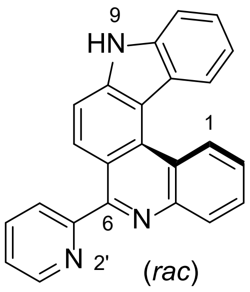

GP 3: N-(2-(9*H*-carbazol-4-yl)phenyl)picolinamide (**2l**; 465 mg, 1.28 mmol, 1.00 equiv.), $POCl_3$ (318 mg, 254 µL, 2.07 mmol, 1.62 equiv.), $PhNO_2$ (12 mL), 150 °C, 16.5 h; two fold purification: silica gel, hexane/EtOAc, 1:0 → 1:1 → 0:1 → 0:1 + 1% MeOH; **3l**: yellow solid (381 mg, 1.10 mmol, 77% over two steps).

R_f = 0.21 (hexane/EtOAc 1:1 + 1% MeOH); ^{1}H NMR (500 MHz, DMSO-d_6, ppm): δ = 12.47 (s, 1 H, 9-NH), 10.38 (s, 1 H, H_{ar}), 9.27 (d, ^3J = 8.2 Hz, 1 H, H_{ar}), 8.88–8.77 (m, 1 H, H_{ar}), 8.70 (d, ^3J = 8.2 Hz, 1 H, H_{ar}), 8.29 (d, ^3J = 8.8 Hz, 1 H, H_{ar}), 8.22 (d, ^3J = 8.0 Hz, 1 H, H_{ar}), 8.09 (t, ^3J = 7.6 Hz, 1 H, H_{ar}), 7.90–7.86 (m, 2 H, 2×H_{ar}), 7.83 (t, ^3J = 7.5 Hz, 1 H, H_{ar}), 7.75 (d, ^3J = 8.1 Hz, 1 H, H_{ar}), 7.65–7.58 (m, 1 H, H_{ar}), 7.54 (t, ^3J = 7.5 Hz, 1 H, H_{ar}), 7.33 (t, ^3J = 7.6 Hz, 1 H, H_{ar}); ^{13}C NMR (125 MHz, DMSO-d_6, ppm): δ = 158.3 (C_q), 158.1 (C_q), 148.4 (CH), 143.6 (C_q), 141.8 (C_q), 140.0 (C_q), 137.3 (CH), 131.3 (C_q), 129.2 (CH), 128.9 (CH), 126.5 (CH), 126.4 (CH), 125.7 (CH), 125.5 (CH), 125.3 (CH), 123.7 (CH), 123.2 (C_q), 122.8 (C_q), 122.5 (CH), 119.4 (C_q), 119.2 (CH), 114.5 (C_q), 113.7 (CH), 112.2 (CH); IR (ATR, cm^{-1}): ν = 3367 (w), 3143 (m), 3073 (m), 3055 (m), 2977 (m), 2837 (w), 2726 (w), 2665 (m), 1619 (w), 1580 (m), 1502 (w), 1459 (m), 1436 (m), 1370 (m), 1279 (w), 1190 (m), 1138 (m), 1036 (m), 991 (m), 868 (w), 818 (m), 799 (m), 728 (s); UV/Vis [THF, nm (mol^{-1}dm^{3}cm^{-1})]: λ_{max} (ε) =310 (26,000), 262 (19,200), 227 (30,400); fluorescence (THF, nm): λ_{ex} = 330; λ_{em} = 434; MS (ESI, neg.): *m/z* (%) = 382 (10), 381 (18), 380 (32), 362 (14), 363 (40), 361 (100), 359 (67), 357 (30), 358 (17), 345 (23) [M]$^-$, 344 (93) [M−1]$^-$; HRMS (ESI, neg.): *m/z* calcd. for $C_{24}H_{14}N_3^-$: 344.1193 [M−H]$^-$; found: 344.1195.

6-(Naphthalene-1-yl)-9*H*-indolo[2,3-*k*]phenanthridine (3n)

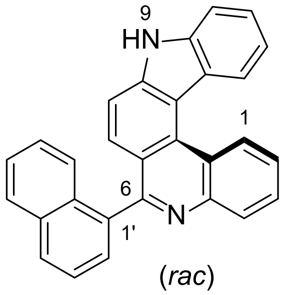

GP 3: *N*-(2-(9*H*-carbazol-4-yl)phenyl)-1-naphthamide (**2n**; 575 mg, 1.39 mmol, 1.00 equiv.), $POCl_3$ (323 mg, 258 µL, 2.11 mmol, 1.51 equiv.), $PhNO_2$ (15 mL), 150 °C, 41.5 h; purification: silica gel, hexane/EtOAc 1:0 → 1:1 → 0:1 → 0:1 + 1% MeOH). Dissolving in CH_2Cl_2, washing with H_2O (2×50 mL), drying over $MgSO_4$ and removing the solvent yielded **3n** as yellow solid (307 mg, 779 µmol, 50% over two steps).

R_f = 0.18 (hexane/EtOAc 4:1); ^{1}H NMR (500 MHz, DMSO-d_6, ppm): δ = 12.30 (s, 1 H, 9-NH), 9.33 (dd, ^3J = 8.0 Hz, ^4J = 1.6 Hz, 1 H, H_{ar}), 8.77 (d, ^3J = 8.2 Hz, 1 H, H_{ar}), 8.20–8.12 (m, 1 H, H_{ar}), 8.12–8.06 (m, 1 H, H_{ar}), 7.93–7.86 (m, 1 H, H_{ar}), 7.89–7.82 (m, 1 H, H_{ar}), 7.77 (d, ^3J = 8.8 Hz, 1 H, H_{ar}), 7.76–7.70 (m, 2 H, 2×H_{ar}), 7.68 (dd, ^3J = 7.0 Hz, ^4J = 1.2 Hz, 1 H, H_{ar}), 7.58–7.52 (m, 2 H, 2×H_{ar}), 7.48 (d, ^3J = 8.8 Hz, 1 H, H_{ar}), 7.39–7.31 (m, 2 H, 2×H_{ar}), 7.28–7.24 (m, 1 H, H_{ar}); ^{13}C NMR (125 MHz, DMSO-d_6, ppm): δ = 160.1 (C_q), 144.4 (C_q), 141.8 (C_q), 140.0 (C_q), 137.9 (C_q), 133.1 (C_q), 131.8 (C_q), 130.6 (C_q), 129.1 (2×CH), 128.5 (CH), 128.3 (CH), 127.2 (CH), 126.5 (CH), 126.4 (CH), 126.1 (CH), 126.1 (CH), 125.8 (CH), 125.7 (CH), 125.4 (CH), 125.2 (CH), 123.2 (C_q), 122.8 (C_q), 122.6 (CH), 121.2 (C_q), 119.3 (CH), 114.6 (C_q), 113.8 (CH), 112.2 (CH); IR (ATR, cm^{-1}): $\tilde{\nu}$ = 3044 (w), 2918 (w), 2838 (w), 1582 (w), 1523 (m), 1479 (w), 1455 (w), 1398 (w), 1358 (m), 1279 (w), 1259 (w), 1186 (w), 1137 (w), 1033 (w), 957 (w), 871 (w), 800 (m), 774 (m), 741 (m), 702 (m); UV/Vis [THF, nm (mol^{-1}dm^{3}cm^{-1})]: λ_{max} (ε) = 306 (51,600), 272 (25,400), 223 (100,000); fluorescence (THF, nm): λ_{ex} = 330; λ_{em} = 385, 400; MS (ESI): *m/z* (%) = 435 (12), 396 (31)

[M+2]$^+$, 395 (100) [M+1]$^+$; HRMS (ESI): *m/z* calcd. for $C_{29}H_{19}N_2^+$: 395.1543 [M+1]$^+$; found: 395.1538.

3.4 Indolo[3,2-*a*]phenanthridines 9

N-(1,2-Dimethyl-4-phenyl-9*H*-carbazol-3-yl)-2,2,2-trifluoroacetamide (8c)

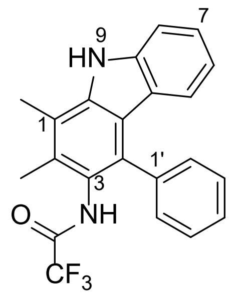

GP 2: 1,2-Dimethyl-4-phenyl-9*H*-carbazol-3-amine (**7**; 120 mg, 419 µmol, 1.00 equiv.), TFA (70.1 µL, 104 mg, 910 µmol, 2.17 equiv.), pyridine (132 µL, 129 mg, 1.63 mmol, 3.89 equiv.), PPAA (≥50% w/w in MeCN; 534 mg, 839 µmol, 2.00 equiv.), MeCN/EtOAc (2:1; 3 mL); purification: silica, gel, hexane/EtOAc, 3:1 and drying in high vacuum (70 °C); **8c**: orange solid (137 mg, 358 µmol, 86%).

R_f = 0.28 (hexane/EtOAc 3:1); ^{1}H NMR (400 MHz, DMSO-d$_6$, ppm): δ = 11.32 (s, 1 H, 9-NH), 10.62 (s, 1 H, NHCO), 7.60–7.44 (m, 4 H, 4×H_{ar}), 7.44–7.18 (m, 3 H, 3×H_{ar}), 6.85–6.77 (m, 1 H, H_{ar}), 6.70 (d, ^3J = 7.9 Hz, 1 H, H_{ar}), 2.57 (s, 3 H, CH_3), 2.25 (s, 3 H, CH_3); ^{13}C NMR (125 MHz, DMSO-d$_6$, ppm): δ = 155.8 (q, $^2J_{CF}$ = 35.7 Hz, CO), 140.4 (C_q), 138.9 (C_q), 137.4 (C_q), 132.0 (C_q), 130.6 (C_q), 128.9 (2×CH), 128.2 (2×CH), 127.7 (CH), 125.2 (CH), 122.9 (C_q), 122.5 (C_q), 120.9 (CH), 118.4 (CH), 118.3 (C_q), 118.2 (C_q), 116.1 (q, $^1J_{CF}$ = 289 Hz, CF_3), 111.1 (CH), 14.3 (CH_3), 14.1 (CH_3); IR (ATR, cm^{-1}): $\tilde{\nu}$ = 3370 (w), 3057 (vw), 2924 (vw), 2851 (vw), 1720 (m), 1524 (w), 1454 (w), 1391 (w), 1302 (w), 1147 (m), 1019 (w), 908 (w), 894 (w), 764 (w), 751 (w), 736 (w), 702 (m); MS (FAB): *m/z* (%) = 384 (13) [M+2]$^+$, 383 (56) [M+1]$^+$, 382 (100) [M]$^+$, 381 (14), 286 (15) [M+1−C_2F_3NO]$^+$, 270 (13) [M−C_2F_3NO]$^+$, 97 (14), 95 (20); HRMS (FAB): *m/z* calcd. for $C_{22}H_{17}F_3N_2O^+$: 382.1287 [M]$^+$; found: 382.1286.

2-Chloro-*N*-(1,2-dimethyl-4-phenyl-9*H*-carbazol-3-yl)acetamide (8d)

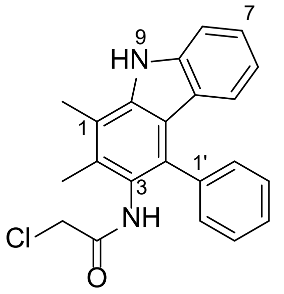

GP 2: 1,2-Dimethyl-4-phenyl-9*H*-carbazol-3-amine (**7**; 120 mg, 419 µmol, 1.00 equiv.), 2-chloroacetic acid (81.9 mg, 867 µmol, 2.07 equiv.), pyridine (107 µL, 105 mg, 1.33 mmol, 3.17 equiv.), PPAA (≥50% w/w in MeCN; 534 mg, 839 µmol, 2.00 equiv.), MeCN/EtOAc (1:2; 3 mL); purification: silica gel, hexane/EtOAc, 0:1 → 3:1 → 1:1 and drying in high vacuum (70 °C, 3 h); **8d**: beige solid (133 mg, 367 µmol, 87%).

R_f = 0.18 (hexane/EtOAc 2:1); ^{1}H NMR (500 MHz, DMSO-d$_6$, ppm): δ = 11.22 (s, 1 H, 9-NH), 9.43 (s, 1 H, NHCO), 7.57–7.43 (m, 4 H, 4×H_{ar}), 7.38–7.20 (m, 3 H, 3×H_{ar}), 6.80–6.74 (m, 1 H, H_{ar}), 6.67 (d, ^3J = 7.9 Hz, 1 H, H_{ar}), 3.96 (s, 2 H, CH_2), 2.55 (s, 3 H, CH_3), 2.25 (s, 3 H, CH_3); ^{13}C NMR (125 MHz, DMSO-d$_6$, ppm): δ = 165.7 (CO), 140.3 (C_q), 138.5 (C_q), 138.1 (C_q), 132.1 (C_q), 131.5 (C_q), 129.1 (2×CH), 128.2 (2×CH), 127.4 (CH), 125.2 (C_q), 124.9 (CH), 122.7 (C_q), 120.9 (CH), 118.3 (C_q), 118.1 (CH), 117.8 (C_q), 110.9 (CH), 42.4 (CH_2), 14.6 (CH_3), 14.2 (CH_3); IR (ATR, cm^{-1}): $\tilde{\nu}$ = 3342 (w), 3298 (w), 2922 (vw), 1681 (w), 1500 (w), 1455 (w), 1392 (w), 1302 (w), 1230 (w), 790 (w), 761 (w), 700 (w); MS (FAB): *m/z* (%) = 365 (28) [M+3]$^+$, 364 (50) [M+2]$^+$, 363 (84) [M+1]$^+$, 362 (100) [M]$^+$, 361 (17), 286 (29) [M+1−C_2H_2ClO]$^+$, 270 (23) [M−C_2H_3ClNO]$^+$, 269 (10), 165 (12); HRMS (FAB): *m/z* calcd. for $C_{22}H_{19}{}^{35}ClN_2O$: 362.1180 [M]$^+$; found: 362.1180.

2-Azido-*N*-(1,2-dimethyl-4-phenyl-9*H*-carbazol-3-yl)acetamide (8e)

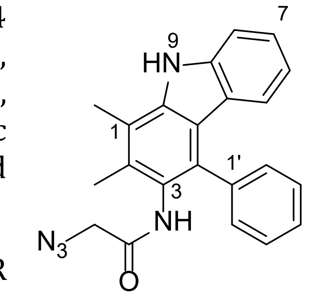

GP 2: 1,2-Dimethyl-4-phenyl-9*H*-carbazol-3-amine (**7**; 150 mg, 524 µmol, 1.00 equiv.), 2-azidoacetic acid (80.5 µL, 109 mg, 1.08 mmol, 2.05 equiv.), pyridine (145 µL, 142 mg, 1.80 mmol, 3.44 equiv.), PPAA (≥50% w/w in MeCN; 733 mg, 1.15 mmol, 2.20 equiv.), EtOAc (1 mL); purification: silica gel, hexane/EtOAc, 6:1 → 1:1 → 1:2 and drying in high vacuum; **8e**: yellow solid (164 mg, 444 µmol, 85%).

R_f = 0.12 (hexane/EtOAc 2:1); R_f = 0.19 (pentane/Et_2O 1:2); ^{1}H NMR (500 MHz, DMSO-d_6, ppm): δ = 11.21 (s, 1 H, 9-NH), 9.31 (s, 1 H, NHCO), 7.54–7.44 (m, 4 H, 4×H_{ar}), 7.37–7.27 (m, 2 H, 2×H_{ar}), 7.27–7.22 (m, 1 H, H_{ar}), 6.78 (t, ^3J = 7.6 Hz, 1 H, H_{ar}), 6.67 (d, ^3J = 7.9 Hz, 1 H, H_{ar}), 3.72 (bs, 2 H, CH_2), 2.55 (s, 3 H, CH_3), 2.25 (s, 3 H, CH_3); ^{13}C NMR (125 MHz, DMSO-d_6, ppm): δ = 167.1 (CO), 140.3 (C_q), 138.5 (C_q), 138.2 (C_q), 132.1 (C_q), 131.4 (C_q), 129.1 (CH), 128.2 (2×CH), 127.4 (CH), 125.2 (C_q), 124.9 (2×CH), 122.7 (C_q), 120.9 (CH), 118.3 (C_q), 118.1 (CH), 117.8 (C_q), 110.9 (CH), 50.7 (CH_2), 14.8 (CH_3), 14.2 (CH_3); IR (ATR, cm^{-1}): $\tilde{\nu}$ = 3388 (w), 3330 (w), 3059 (vw), 2922 (vw), 2109 (m), 1680 (m), 1497 (w), 1454 (w), 1394 (w), 1279 (w), 1234 (m), 930 (w), 753 (m), 740 (m), 701 (m); MS (FAB): *m/z* (%) = 371 (24) $[M+2]^+$, 370 (94) $[M+1]^+$, 369 (100) $[M]^+$, 368 (15), 327 (13) $[M-N_3]^+$, 313 (23), 312 (28), 286 (28) $[M+1-C_2H_2N_3O]^+$, 270 (26) $[M-C_2H_3N_4O]^+$, 269 (15), 111 (25); HRMS (FAB): *m/z* calcd. for $C_{22}H_{19}N_5O^+$: 369.1584 $[M]^+$; found: 369.1584.

(2*E*,4*E*)-*N*-(1,2-dimethyl-4-phenyl-9*H*-carbazol-3-yl)hexa-2,4-dienamide (8g)

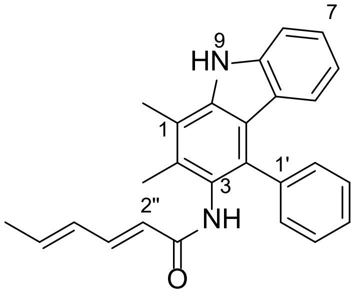

GP 2: 1,2-Dimethyl-4-phenyl-9*H*-carbazol-3-amine (**7**; 110 mg, 384 µmol, 1.00 equiv.), sorbic acid (87.7 mg, 782 µmol, 2.04 equiv.), pyridine (105 µL, 103 mL, 1.30 mmol, 3.37 equiv.), PPAA (≥50% w/w in MeCN; 495 mg, 778 µmol, 2.03 equiv.), EtOAc/MeCN (3:2; 2.5 mL); purification: silica gel, hexane/EtOAc, 5:1 → 1:1 → 0:1 and drying in high vacuum; **8g**: beige powder (116 mg, 305 µmol, 79%).

R_f = 0.42 (hexane/EtOAc 1:1); ^{1}H NMR (400 MHz, DMSO-d_6, ppm): δ = 11.18 (s, 1 H, 9-NH), 9.08 (s, 1 H, NHCO), 7.56–7. 37 (m, 4 H, 4×H_{ar}), 7.37–7.15 (m, 3 H, 3×H_{ar}), 6.96 (dd, ^3J = 15.2 Hz, ^3J = 10.7, 1 H, 3''-H), 6.77 (t, ^3J = 7.5 Hz, 1 H, H_{ar}), 6.65 (d, ^3J = 7.9 Hz, 1 H, H_{ar}), 6.23–6.12 (m, 1 H, 4''-H), 6.12–5.99 (m, 1 H, 5''-H), 5.89 (d, ^3J = 15.2 Hz, 1 H, 2''-H), 2.54 (s, 3 H, CH_3), 2.21 (s, 3 H, CH_3), 1.78 (d, ^3J = 6.5 Hz, 3 H, 6-H_3); ^{13}C NMR (125 MHz, DMSO-d_6, ppm): δ = 165.3 (CO), 140.3 (C_q), 139.6 (CH), 138.5 (C_q), 138.3 (C_q), 136.7 (CH), 132.1 (C_q), 131.7 (C_q), 129.9 (CH), 129.1 (CH), 128.1 (CH), 127.3 (CH), 126.1 (C_q), 124.8 (CH), 122.7 (C_q), 122.7 (CH), 120.9 (CH), 118.2 (C_q), 118.0 (CH), 117.6 (C_q), 110.9 (CH), 18.3 (CH_3), 14.8 (CH_3), 14.2 (CH_3)*; IR (ATR, cm^{-1}): $\tilde{\nu}$ =3377 (vw), 3235 (vw), 3054 (vw), 3025 (vw), 2913 (vw), 1658 (w), 1635 (w), 1602 (w), 1489 (w), 1454 (w), 1392 (w), 1336 (w), 1298 (w), 1248 (w), 1151 (w), 995 (w), 863 (vw), 794 (vw), 751 (w), 738 (m), 701 (m); MS (FAB): *m/z* (%) = 382 (28) $[M+2]^+$, 381 (100) $[M+1]^+$, 380 (85) $[M]^+$, 379 (11), 286 (23) $[M+1-C_6H_7O]^+$, 270 (18) $[M-C_6H_8NO]^+$, 223 (22), 95 (23), 89 (43); HRMS (FAB): *m/z* calcd. for $C_{26}H_{25}N_2O^+$: 381.1961 $[M+1]^+$; found: 381.1960.
*Two signals of CH groups are covered and could not be identified.

N-(1,2-Dimethyl-4-phenyl-9*H*-carbazol-3-yl)-4-methoxybenzamide 8i)

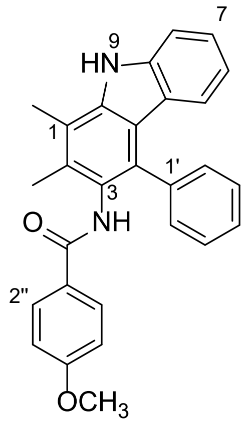

GP 1: 1,2-Dimethyl-4-phenyl-9*H*-carbazol-3-amine (**7**; 80.2 mg, 280 µmol, 1.00 equiv.), 4-methoxybenzoyl chloride (70.2 mg, 412 µmol, 1.47 equiv.), Et_3N (51.6 µL, 37.7 mg, 373 µmol, 1.33 equiv.), anhydrous CH_2Cl_2 (10 mL); purification: silica gel, hexane/EtOAc, 0:1 → 3:1 → 1:1 and drying in high vacuum; **8i**: yellow powder (91.0 mg, 216 µmol, 77%).

R_f = 0.41 (hexane/EtOAc 1:1); ^{1}H NMR (400 MHz, DMSO-d$_6$, ppm): δ = 11.21 (s, 1 H, 9-NH), 9.41 (s, 1 H, NHCO), 7.70–7.63 (m, 2 H, 2×H$_{ar}$), 7.58–7.29 (m, 6 H, 6×H$_{ar}$), 7.28–7.21 (m, 1 H, H$_{ar}$), 6,96–6.90 (m, 2 H, 2×H$_{ar}$), 6.82–6.75 (m, 1 H, H$_{ar}$), 6.69 (d, ^3J = 7.9 Hz, 1 H, H$_{ar}$), 3.78 (s, 3 H, OCH_3), 2.57 (s, 3 H, CH_3), 2.28 (s, 3 H, CH_3); ^{13}C NMR (125 MHz, DMSO-d$_6$, ppm): δ = 165.9 (CO), 161.5 (C$_q$), 140.3 (C$_q$), 138.6 (C$_q$), 138.4 (C$_q$), 132.5 (C$_q$), 132.0 (C$_q$), 129.1 (3×CH), 128.0 (CH), 127.2 (C$_q$), 127.0 (CH), 126.4 (C$_q$), 124.8 (CH), 122.8 (C$_q$), 120.9 (CH), 118.3 (C$_q$), 118.0 (CH), 117.6 (C$_q$), 113.4 (3×CH), 110.9 (CH), 55.3 (OCH_3), 14.8 (CH_3), 14.3 (CH_3)*; IR (ATR, cm^{-1}): $\tilde{\nu}$ = 3390 (w), 3222 (w), 1739 (vw), 1643 (m), 1603 (m), 1576 (w), 1509 (w), 1454 (m), 1392 (w), 1302 (w), 1250 (m), 1183 (m), 1113 (w), 1029 (w), 839 (w), 783 (vw), 752 (m), 741 (m), 702 (m); MS (FAB): *m/z* (%) = 422 (30) [M+2]$^+$, 421 (100) [M+1]$^+$, 420 (99) [M]$^+$, 419 (12), 270 (17) [M−$C_8H_8NO_2$]$^+$; HRMS (FAB): *m/z* calcd. for $C_{28}H_{25}N_2O_2^+$: 421.1911 [M+1]$^+$; found: 421.1910. *The signal of one CH group is covered and could not be identified.

N-(1,2-Dimethyl-4-phenyl-9*H*-carbazol-3-yl)-4-(trifluoromethyl)benzamide (8j)

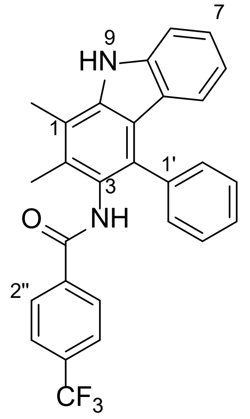

GP 1: 1,2-Dimethyl-4-phenyl-9*H*-carbazol-3-amine (**7**; 110 mg, 384 µmol, 1.00 equiv.), 4-(trifluoromethyl)benzoyl chloride (71.2 µL, 99.9 mg, 479 µmol, 1.25 equiv.), Et_3N (58.8 µL, 42.9 mg, 424 µmol, 1.10 equiv.), anhydrous CH_2Cl_2 (10 mL); purification: silica gel, hexane/EtOAc, 3:1 → 1:2 and drying in high vacuum; **8j**: beige powder (154 mg, 336 µmol, 87%).

R_f = 0.29 (hexane/EtOAc 2:1); ^{1}H NMR (400 MHz, DMSO-d$_6$, ppm): δ = 11.25 (s, 1 H, 9-NH), 9.82 (s, 1 H, NHCO), 7.89–7.75 (m, 4 H, 4×H$_{ar}$), 7.64–7.31 (m, 6 H, 6×H$_{ar}$), 7.31–7.23 (m, 1 H, H$_{ar}$), 6.82–6.77 (m, 1 H, H$_{ar}$), 6.71 (d, ^3J = 7.9 Hz, 1 H, H$_{ar}$), 2.58 (s, 3 H, CH_3), 2.31 (s, 3 H, CH_3); ^{13}C NMR (125 MHz, DMSO-d$_6$, ppm): δ = 165.4 (CO), 140.3 (C$_q$), 138.7 (C$_q$), 138.6 (C$_q$), 138.4 (C$_q$), 132.4 (C$_q$), 131.7 (C$_q$), 131.0 (q, $^2J_{CF}$ = 32 Hz, C-2''), 128.1 (6×CH), 127.3 (CH), 125.8 (C$_q$), 125.3 (q, 2×CH), 124.9 (CH), 123.9 (q, $^1J_{CF}$ = 272 Hz, CF_3), 122.7 (C$_q$), 120.9 (CH), 118.3 (C$_q$), 118.1 (CH), 117.8 (C$_q$), 111.0 (CH), 14.8 (CH_3), 14.2 (CH_3); IR (ATR, cm^{-1}): $\tilde{\nu}$ = 3393 (vw), 3063 (vw), 2927 (vw), 3292 (vw), 1662 (w), 1579 (w), 1519 (w), 1484 (w), 1455 (w), 1393 (w), 1322 (m), 1164 (m), 1124 (m), 1064 (m), 1016 (w), 853 (w), 770 (w), 742 (m), 702 (m); MS (FAB): *m/z* (%) = 460 (22) [M+2]$^+$, 459 (82) [M+1]$^+$, 458 (100) [M]$^+$, 457 (15), 286 (15) [M−$C_8H_4F_3O$]$^+$, 270 (20) [M−$C_8H_5F_3NO$]$^+$, 173 (17) [$C_8H_4F_3O$]; HRMS (FAB): *m/z* calcd. for $C_{28}H_{21}F_3N_2O$: 458.1600 [M]$^+$; found: 458.1599.

N-(1,2-Dimethyl-4-phenyl-9*H*-carbazol-3-yl)-2,4,6-trimethylbenzamide (8k)

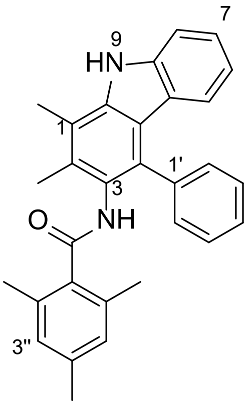

GP 2: 1,2-Dimethyl-4-phenyl-9*H*-carbazol-3-amine (**7**; 110 mg, 384 µmol, 1.00 equiv.), 2,4,6-trimethylbenzoic acid (126 mg, 768 µmol, 2.00 equiv.), pyridine (102 µL, 100 mg, 1.27 mmol, 3.30 equiv.), PPAA (≥50% w/w in MeCN; 513 mg, 806 µmol, 2.10 equiv.), EtOAc (0.6 mL). Purification: The crude product was heated to 80 °C in H_2O (50 mL) and filtered while hot. Traces of solvent were removed in high vacuum (rt, 5 h) to yield **8k** as a red brown, poorly soluble powder (163 mg), which was used without further purification.

^{1}H NMR (400 MHz, DMSO-d$_6$, ppm): δ = 11.32 (s, 1 H, 9-NH), 9.24 (bs, 1 H, NHCO), 7.77–7.60 (m, 3 H, 3×H$_{ar}$), 7.51–7.34 (m, 3 H, 3×H$_{ar}$), 7.26 (t, ^3J = 7.7 Hz, 1 H, H$_{ar}$), 6.87 (s, 2 H, 2×H$_{ar}$), 6.78 (t, ^3J = 7.7 Hz, 1 H, H$_{ar}$), 6.49 (d, ^3J = 8.0 Hz, 1 H, H$_{ar}$), 2.56 (s, 3 H, CH_3), 2.42 (s, 3 H, CH_3), 2.23 (s, 9 H, 3×CH_3); ^{13}C NMR (125 MHz, DMSO-d$_6$): Analysis of the data was not pursued due to the presence of trace impurities and a possible occurrence of rotamers; IR (ATR, cm^{-1}): $\tilde{\nu}$ = 3279 (vw), 3246 (w), 2920 (w), 2853 (w), 2648 (w), 2537 (w), 1681 (w), 1609 (w), 1575 (w), 1500 (w), 1435 (w), 1395 (w), 1294 (w), 1178 (w), 1098 (w), 935 (w), 855 (w), 781 (w), 739 (w), 706 (w); MS (ESI): *m/z* (%) = 434 (33) [M+2]$^+$, 433 (100) [M+1]$^+$, 408 (16), 389 (10).

5-(Chloromethyl)-7,8-dimethyl-9*H*-indolo[3,2-*a*]phenanthridine (9d)

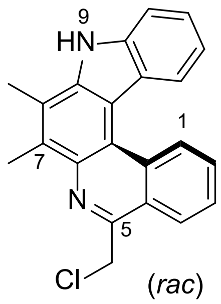

GP 3: 2-Chloro-*N*-(1,2-dimethyl-4-phenyl-9*H*-carbazol-3-yl)acetamide (**8d**; 119 mg, 329 µmol, 1.00 equiv.), $POCl_3$ (49.8 µL, 83.6 mg, 545 µmol, 1.66 equiv.), $PhNO_2$ (3 mL), 150 °C, 3.5 h; purification: silica gel, hexane/CH_2Cl_2 3:1; **9d**: yellow crystal solid (38.7 mg, 112 µmol, 34%).

R_f = 0.16 (hexane/CH_2Cl_2 2:1); ^{1}H NMR (400 MHz, DMSO-d$_6$, ppm): δ = 11.86 (s, 1 H, H$_{ar}$); 9.23 (d, ^3J = 8.4 Hz, 1 H, H$_{ar}$), 8.55–8.44 (m, 2 H, 2×H$_{ar}$), 8.00–7.93 (m, 1 H, H$_{ar}$), 7.85 (t, ^3J = 7.3 Hz, 1 H, H$_{ar}$), 7.72 (d, ^3J = 8.1 Hz, 1 H, H$_{ar}$), 7.46 (t, ^3J = 7.6 Hz, 1 H, H$_{ar}$), 7.30–7.15 (m, 1 H, H$_{ar}$), 5.54 (s, 2 H, CH_2), 2.88 (s, 3 H, CH_3), 2.73 (s, 3 H, CH_3); ^{13}C NMR (125 MHz, DMSO-d$_6$, ppm): δ = 149.9 (C$_q$), 139.8 (2×C$_q$), 137.5 (C$_q$), 132.4 (C$_q$), 132.0 (C$_q$), 128.9 (CH), 127.3 (CH), 126.5 (CH), 125.6 (CH), 124.8 (CH), 123.4 (C$_q$), 123.3 (C$_q$), 122.3 (C$_q$), 122.0 (CH), 119.3 (C$_q$), 118.7 (CH), 112.1 (CH), 46.3 (CH_2), 14.7 (CH_3), 13.9 (CH_3)*; IR (ATR, cm^{-1}): $\tilde{\nu}$ = 3337 (w), 2851 (w), 2920 (w), 1672 (m), 1580 (w), 1517 (w), 1456 (w), 1365 (w), 1346 (w), 1331 (w), 1308 (w), 1255 (w), 1136 (w), 1059 (m), 955 (w), 909 (w), 871 (vw), 799 (w), 763 (w), 742 (m), 731 (m); UV/Vis [THF, nm (mol^{-1}dm^{3}cm^{-1})]: λ_{max} (ε) = 395 (11,300), 327 (28,300), 224 (40,000); fluorescence (THF, nm): λ_{ex} = 330; λ_{em} = 491, 420, 402, 365; MS (FAB): *m/z* (%) = 447 (7,) 446 (20), 345 (5) [M+1]$^+$, 344 (5) [M]$^+$, 326 (6), 325 (15), 307 (21), 289 (14), 273 (5), 217 (9), 195 (10), 165 (8), 154 (100) [3-NBA], 120 (13); HRMS (FAB): *m/z* calcd. for $C_{22}H_{17}{}^{35}ClN_2^+$: 344.1075 [M]$^+$; found: 344.1075. *The signal of one C$_q$ is covered and could not be identified.

5-(Azidomethyl)-7,8-dimethyl-9*H*-indolo[3,2-*a*]phenanthridine (9e)

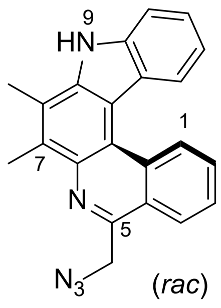

Following a published protocol,[34] Tf_2O (195 µL, 327 mg, 1.16 mmol, 3.00 equiv.) was added dropwise to a cooled (0 °C) solution of Ph_3PO (161 mg, 579 µmol, 1.50 equiv.) in anhydrous CH_2Cl_2 (3 mL) and the mixture was stirred at 0 °C for 15 min. A solution of 2-azido-*N*-(1,2-dimethyl-4-phenyl-9*H*-carbazol-3-yl)acetamide (**8e**; 143 mg, 387 µmol, 1.00 equiv.) in anhydrous CH_2Cl_2 (5 mL) was added dropwise and the mixture was stirred at 0 °C for 1 h, warmed to room temperature, and stirred until full conversion (TLC, 2 h). Saturated aqueous $NaHCO_3$ solution (10 mL) was added, and the mixture was stirred for 10 min. The layers were separated, and the aqueous layer was extracted with CH_2Cl_2 (3×20 mL). The combined organic layers were dried ($MgSO_4$), concentrated under reduced pressure, and purified by column chromatography (silica gel, hexane/EtOAc, 2:1). Traces of solvent were removed in high vacuum (30 °C, 6 h) to yield **9e** (115 mg, 327 mmol, 85%) as a yellow crystalline solid.

R_f = 0.29 (hexane/EtOAc 5:1); ^{1}H NMR (400 MHz, DMSO-d$_6$, ppm): δ = 11.83 (s, 1 H, NH), 9.24 (d, ^3J = 8.4 Hz, 1 H, H$_{ar}$), 8.52 (d, ^3J = 8.2 Hz, 1 H, H$_{ar}$), 8.35 (d, ^3J = 8.2 Hz, 1 H, H$_{ar}$), 7.96 (t, ^3J = 7.6 Hz, 1 H, H$_{ar}$), 7.82 (t, ^3J = 7.6 Hz, 1 H, H$_{ar}$), 7.72 (d, ^3J = 8.1 Hz, 1 H, H$_{ar}$), 7.46 (t, ^3J = 7.5 Hz, 1 H, H$_{ar}$), 7.23 (t, ^3J = 7.6 Hz, 1 H, H$_{ar}$), 5.16 (s, 2 H, CH$_2$), 2.92 (s, 3 H, CH$_3$), 2.75 (s, 3 H, CH$_3$); ^{13}C NMR (125 MHz, DMSO-d$_6$, ppm): δ = 149.3 (C$_q$), 139.8 (C$_q$), 139.5 (C$_q$), 137.6 (C$_q$), 132.3 (C$_q$), 131.8 (C$_q$), 127.3 (CH), 126.5 (CH), 124.8 (CH), 124.7 (CH), 123.4 (C$_q$), 123.4 (C$_q$), 122.2 (C$_q$), 122.0 (CH), 118.8 (C$_q$), 118.6 (CH), 112.1 (C$_q$), 112.0 (CH), 52.3 (CH$_2$), 14.6 (CH$_3$), 14.0 (CH$_3$); IR (ATR, cm^{-1}): $\tilde{\nu}$ = 3342 (w) (NH), 2918 (w) (CH$_2$), 2097 (m) (N$_3$), 1582 (w), 1528 (w), 1444 (w), 1307 (w), 1281 (w), 1240 (w), 1139 (w), 1024 (w), 919 (w), 762 (w), 746 (m), 735 (m); UV/Vis [THF, nm (mol^{-1}dm^{3}cm^{-1})]: λ_{max} (ε) = 320 (33,900), 269 (16,200), 230 (36,600); fluorescence (THF, nm): λ_{ex} = 330; λ_{em} = 421, 400, 365; MS (FAB): *m/z* (%) = 353 (18) [M+2]$^+$, 352 (65) [M+1]$^+$, 351 (61) [M]$^+$, 326 (12) [M+3−N$_2$]$^+$, 325 (29) [M+2−N$_2$]$^+$, 324 (43) [M+1−N$_2$]$^+$, 309 (50) [M−N$_3$]$^+$, 295 (26) [M−CH$_2$N$_3$•]$^+$, 293 (17), 281 (13), 167 (11), 133 (100) [3-NBA−OH], 109 (37), 97 (44), 95 (68); HRMS (FAB): *m/z* calcd. for $C_{22}H_{18}N_5^+$: 352.1557 [M+1]$^+$; found: 352.1556.

7,8-Dimethyl-5-[(1*E*,3*E*)-penta-1,3-dien-1-yl]-9*H*-indolo[3,2-*a*]phenanthridine (9g)

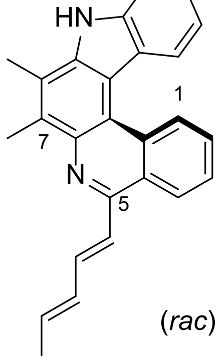

GP 3: *N*-(1,2-Dimethyl-4-phenyl-9*H*-carbazol-3-yl)hexa-2,4-dienamide (**8g**; 105 mg, 277 µmol, 1.00 equiv.), $POCl_3$ (41.7 µL, 70.0 mg, 457 µmol, 1.65 equiv.), $PhNO_2$ (3 mL), 150 °C, 3.5 h; purification: silica gel, hexane/CH_2Cl_2, 1:0 → 4:1 → 1:1 → 1:2; **9g**: yellow crystalline solid (43.0 mg, 119 µmol, 43%).

R_f = 0.28 (hexane/CH_2Cl_2 1:1); ^{1}H NMR (500 MHz, DMSO-d$_6$, ppm): δ = 11.79 (s, 1 H, NH); 9.18 (d, ^3J = 8.2 Hz, 1 H, H$_{ar}$), 8.57 (d, ^3J = 8.3 Hz, 1 H, H$_{ar}$), 8.49 (d, ^3J = 8.2 Hz, 1 H, H$_{ar}$), 7.94–7.89 (m, 1 H, H$_{ar}$), 7.83–7.76 (m, 1 H, H$_{ar}$), 7.74–7.66 (m, 2 H, 2×H$_{diene}$), 7.65–7.59 (m, 1 H, H$_{ar}$), 7.46–7.40 (m, 1 H, H$_{ar}$), 7.24–7.18 (m, 1 H, H$_{ar}$), 6.61–6.51 (m, 1 H, H$_{diene}$), 6.20-6.09 (m, 1 H, H$_{diene}$), 2.93 (s, 3 H, CH$_3$), 2.73 (s, 3 H, CH$_3$), 1.95–1. 84 (m, 3 H, CH$_3$); ^{13}C NMR (125 MHz, DMSO-d$_6$, ppm): δ = 148.5 (C$_q$), 139.7 (C$_q$), 139.2 (C$_q$), 138.3 (C$_q$), 135.1 (CH), 133.3 (CH), 132.3 (C$_q$), 132.0 (CH), 131.7 (C$_q$), 128.3 (CH), 127.1 (CH), 126.3 (CH), 125.0

(CH), 124.6 (CH), 124.5 (CH), 124.1 (C_q), 123.5 (C_q), 122.1 (C_q), 122.0 (CH), 118.5 (CH), 118.4 (C_q), 112.1 (C_q), 112.0 (CH), 18.5 (CH_3), 14.7 (CH_3), 13.9 (CH_3); IR (ATR, cm^{-1}): $\tilde{\nu}$ = 3411 (w), 3047 (vw), 3006 (vw), 2960 (vw), 2908 (w), 1638 (vw), 1581 (w), 1560 (w), 1477 (w), 1452 (w), 1365 (w), 1310 (w), 1249 (w), 1145 (w), 1022 (w), 985 (w), 931 (w), 914 (w), 863 (w), 761 (w), 746 (m); UV/Vis [THF, nm ($mol^{-1}dm^3cm^{-1}$)]: λ_{max} (ε)= 323 (21,200), 225 (33,400); fluorescence (THF, nm): λ_{ex} = 330; λ_{em} = 495, 415, 365; MS (FAB): *m/z* (%) = 364 (30) $[M+2]^+$, 363 (100) $[M+1]^+$, 362 (56) $[M]^+$, 307 (12) $[M-CH_3{}^{\cdot}-C_3H_5{}^{\bullet}]^+$; HRMS (FAB): *m/z* calcd. for $C_{26}H_{23}N_2{}^+$: 363.1856 $[M+1]^+$; found: 363.1853.

5-(4-Methoxyphenyl)-7,8-dimethyl-9*H*-indolo[3,2-*a*]phenanthridine (9i)

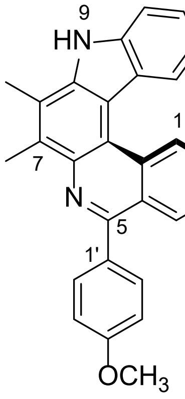

(*rac*)

GP 3: *N*-(1,2-Dimethyl-4-phenyl-9*H*-carbazol-3-yl)-4-methoxybenzamide (**8i**; 66.1 mg, 157 µmol, 1.00 equiv.), $POCl_3$ (42.6 µL, 71.5 mg, 466 µmol, 2.97 equiv.), $PhNO_2$ (3 mL), 150 °C, 15 h; purification: silica gel, hexane/CH_2Cl_2, 1:0 → 1:1 → 1:3) and drying in high vacuum (70 °C, 8 h); **9i**: yellow crystalline solid (52.0 mg, 129 µmol, 82%).

R_f = 0.35 (hexane/CH_2Cl_2 1:2); ^1H NMR (400 MHz, DMSO-d6, ppm): δ = 11.81 (s, 1 H, 9-NH), 9.24 (d, ^3J = 8.3 Hz, 1 H, H_{ar}), 8.55 (d, ^3J = 8.1 Hz, 1 H, H_{ar}), 8.23 (dd, ^4J = 8.3 Hz, ^4J = 1.3 Hz, 1 H, H_{ar}), 7.98–7.84 (m, 3 H, 3×H_{ar}), 7.78–7.66 (m, 2 H, 2×H_{ar}), 7.50–7.39 (m, 1 H, H_{ar}), 7.28–7.22 (m, 1 H, H_{ar}), 7.22–7.16 (m, 2 H, 2×H_{ar}), 3.89 (s, 3 H, OCH_3), 2.90 (s, 3 H, CH_3), 2.75 (s, 3 H, CH_3); ^{13}C NMR (125 MHz, DMSO-d6, ppm): δ = 159.6 (C_q), 153.7 (C_q), 139.7 (C_q), 139.3 (C_q), 137.8 (C_q), 132.4 (C_q), 132.3 (C_q), 131.9 (C_q), 131.3 (2×CH), 128.5 (CH), 127.2 (CH), 127.1 (CH), 126.2 (CH), 124.5 (CH), 123.7 (C_q), 123.5 (C_q), 122.0 (C_q), 121.9 (CH), 118.5 (CH), 117.8 (C_q), 113.9 (2×CH), 112.0 (CH), 111.9 (C_q), 55.3 (OCH_3), 14.6 (CH_3), 13.9 (CH_3); IR (ATR, cm^{-1}): $\tilde{\nu}$ = 3446 (w), 3048 (vw), 2919 (w), 2852 (w), 1605 (w), 1509 (w), 1452 (w), 1367 (w), 1309 (w), 1242 (m), 1171 (w), 1106 (w), 1029 (m), 980 (w), 840 (m), 781 (w), 762 (w), 743 (m), 733 (m); UV/Vis [THF, nm ($mol^{-1}dm^3cm^{-1}$)]: λ_{max} (ε) = 371 (10,500), 324 (30,500), 275 (18,000), 219 (39,200); fluorescence (THF, nm): λ_{ex} = 330; λ_{em} = 429, 365; MS (FAB): *m/z* (%) = 404 (31) $[M+2]^+$, 403 (100) $[M+1]^+$, 402 (76) $[M]^+$, 401 (27), 133 (41), 97 (14), 95 (23); HRMS (FAB): *m/z* calcd. for $C_{28}H_{23}N_2O^+$: 403.1805 $[M+1]^+$; found: 403.1804.

7,8-Dimethyl-5-[4-(trifluoromethyl)phenyl]-9*H*-indolo[3,2-*a*]phenanthridine (9j)

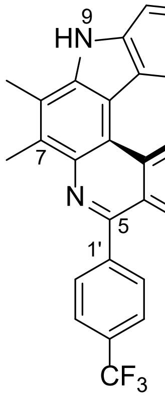

(*rac*)

GP 3: *N*-(1,2-Dimethyl-4-phenyl-9*H*-carbazol-3-yl)-4-(trifluoromethyl)benzamide (**8j**; 129 mg, 282 µmol, 1.00 equiv.), $POCl_3$ (46.4 µL, 78.0 mg, 509 µmol, 1.81 equiv.), $PhNO_2$ (3 mL), 150 °C, 3.5 h; purification: silica gel, hexane/CH_2Cl_2, 3:1; **9j**: yellow powder (103 mg, 235 µmol, 83%).

R_f = 0.20 (hexane/CH_2Cl_2 2:1); ^1H NMR (400 MHz, DMSO-d6, ppm): δ =11.89 (s, 1 H, NH), 9.28 (d, ^3J = 8.3 Hz, 1 H, H_{ar}), 8.56 (d, ^3J = 8.2 Hz, 1 H, H_{ar}), 8.16 (d, ^3J = 8.0 Hz, 3 H, 3×H_{ar}), 8.05–7.88 (m, 3 H, 3×H_{ar}), 7.81–7.69 (m, 2 H, 2×H_{ar}), 7.47 (t, ^3J = 7.6 Hz, 1 H, H_{ar}), 7.26 (t, ^3J = 7.6 Hz, 1 H, H_{ar}), 2.91 (s, 3 H, CH_3), 2.76 (s, 3 H, CH_3); ^1H NMR (400 MHz, $CDCl_3$/DMSO-d6 (2:3), ppm): δ = 11.60 (s, 1 H, NH), 9.27 (d, ^3J = 8.3 1 H, H_{ar}), 8.53 (d, ^3J = 8.3 Hz, 1 H, H_{ar}), 8.17–8.12 (m, 1 H, H_{ar}), 8.09 (d, ^3J = 8.0 Hz, 2 H, H_{ar}),

7.88–7.78 (m, 3 H, H_{ar}), 7.70–7.59 (m, 2 H, H_{ar}), 7.42–7.34 (m, 1 H, H_{ar}), 7.22–7.13 (m, 1 H, H_{ar}), 2.88 (s, 3 H, CH_3), 2.73 (s, 3 H, CH_3); ^{13}C NMR (100 MHz, $CDCl_3$/DMSO-d_6 (2:3, ppm): δ = 152.2 (C_q), 143.3 (C_q), 139.6 (C_q), 139.6 (C_q), 137.5 (C_q), 132.4 (C_q), 132.2 (C_q), 130.4 (2×CH), 129.2 (q, $^2J_{CF}$ = 31.8 Hz, C-4', only two peaks of the quartet were visible), 128.3 (CH), 126.8 (CH), 126.5 (CH), 126.3 (CH), 124.9 (CH), 124.9 (CH), 124.3 (CH), 124.0 (q, $^1J_{CF}$ = 272 Hz, CF_3, only two peaks of the quartet were visible), 123.5 (C_q), 123.4 (C_q), 122.0 (C_q), 121.8 (CH), 118.4 and 118.3 (CH and C_q), 111.9 (CH), 111.7 (C_q), 14.5 (CH_3), 13.7 (CH_3); IR (ATR, cm^{-1}): $\tilde{\nu}$ = 3463 (vw), 2913 (vw), 1584 (w), 1520 (w), 1456 (w), 1403 (w), 1320 (m), 1255 (w), 1162 (m), 1108 (m), 1065 (m), 1015 (w), 981 (w), 851 (m), 782 (w), 746 (m), 727 (m); UV/Vis [THF, nm (mol^{-1}dm^{3}cm^{-1})]: λ_{max} (ε) = 372 (13,300), 324 (38,600), 226 (57,300); fluorescence (THF, nm): λ_{ex} = 330; λ_{em} = 459, 365; MS (FAB): *m/z* (%) = 442 (28) [M+2]$^+$, 441 (100) [M+1]$^+$, 440 (90) [M]$^+$, 439 (25), 97 (12), 95 (14); HRMS (FAB): *m/z* calcd. for $C_{28}H_{20}F_3N_2^+$: 441.1573 [M+1]$^+$; found: 441.1575.

5-Mesityl-7,8-dimethyl-9*H*-indolo[3,2-*a*]phenanthridine (9k)

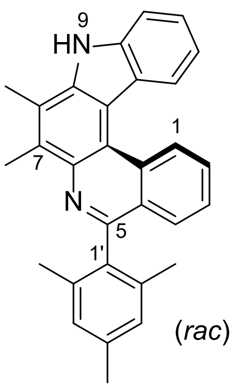

GP 3: *N*-(1,2-Dimethyl-4-phenyl-9*H*-carbazol-3-yl)-2,4,6-trimethylbenzamide (**8k**; crude, non-purified material; 125 mg, 290 µmol, 1.00 equiv.), $POCl_3$ (43.6 µL, 73.3 mg, 478 µmol, 1.65 equiv.), $PhNO_2$ (3 mL), 150 °C, 3.5 h; purification: silica gel, hexane/CH_2Cl_2, 1:0 → 4:1 and drying in high vacuum; **9k**: yellow powder (64.0 mg, 154 µmol, 52%, two steps).

R_f = 0.27 (hexane/CH_2Cl_2 1:1); ^{1}H NMR (400 MHz, DMSO-d_6, ppm): δ = 11.82 (s, 1 H, NH), 9.28 (d, ^3J = 8.3 Hz, 1 H, H_{ar}), 8.59 (d, ^3J = 8.2 Hz, 1 H, H_{ar}), 7.98–7.87 (m, 1 H, H_{ar}), 7.73 (d, ^3J = 8.1 Hz, 1 H, H_{ar}), 7.67 (t, ^3J = 7.5 Hz, 1 H, H_{ar}), 7.59–7.52 (m, 1 H, H_{ar}), 7.46 (t, ^3J = 7.6 Hz, 1 H, H_{ar}), 7.24 (t, ^3J = 7.6 Hz, 1 H, H_{ar}), 7.06 (s, 2 H, 3'-H, 5'-H), 2.82 (s, 3 H, CH_3), 2.75 (s, 3 H, CH_3), 2.38 (s, 3 H, 4'-CH_3), 1.88 (s, 6 H, 2'-CH_3, 6'-CH_3); ^{13}C NMR (125 MHz, DMSO-d_6, ppm): δ = 155.0 (C_q), 139.8 (C_q), 139.4 (C_q), 138.5 (C_q), 136.9 (C_q), 136.1 (C_q), 135.9 (2×C_q), 132.4 (C_q), 131.6 (C_q), 128.7 (CH), 128.2 (2×CH), 127.4 (CH), 126.4 (CH), 126.0 (CH), 124.8 (C_q), 124.6 (CH), 123.6 (C_q), 122.1 (CH), 122.0 (C_q), 118.5 (CH), 118.2 (C_q), 112.2 (C_q), 112.0 (CH), 20.8 (CH_3), 19.7 (2×CH_3), 14.6 (CH_3), 14.1 (CH_3); IR (ATR, cm^{-1}): $\tilde{\nu}$ = 3242 (vw), 2917 (w), 1611 (vw), 1569 (w), 1519 (vw), 1454 (w), 1363 (w), 1308 (w), 1254 (w), 1191 (w), 1148 (w), 1028 (w), 981 (w), 930 (w), 852 (w), 778 (w), 747 (m), 683 (w), 661 (w);UV/Vis [THF, nm (mol^{-1}dm^{3}cm^{-1})]: λ_{max} (ε) = 320 (40,300), 268 (20,300), 230 (42,700); fluorescence (THF, nm): λ_{ex} = 330; λ_{em} = 415, 366; MS (FAB): *m/z* (%) = 416 (33) [M+2]$^+$, 415 (100) [M+1]$^+$, 414 (57) [M]$^+$, 413 (35), 307 (23), 289 (11); HRMS (FAB): *m/z* calcd. for $C_{30}H_{27}N_2^+$: 415.2169 [M+1]$^+$; found: 415.2170.

3.5 Modification of Indolophenanthridines 3 and 9

5-Methyl-6-phenyl-9*H*-indolo[2,3-*k*]phenanthridin-5-ium Tetrafluoroborate (10)

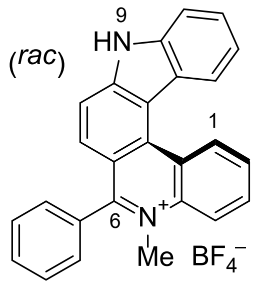

Following a published protocol,[39] trimethyloxonium tetrafluoroborate (47.2 mg, 319 µmol, 1.10 equiv.) was added under argon to a solution of 6-phenyl-9*H*-indolo[2,3-*k*]phenanthridine (**3h**; 99.8 mg, 290 µmol, 1.00 equiv.) in 20 mL anhydrous CH_2Cl_2. The mixture was stirred at room temperature for 70 h and the solvent was removed under reduced pressure. The residue was heated in a little EtOH and the supernatant was decanted off. Drying the residue in vacuum yielded **10** (72.6 mg, 163 µmol, 56%) as yellow powder.

^{1}H NMR (400 MHz, DMSO-d$_6$, ppm): δ = 13.02 (s, 1 H, 9-NH), 9.46 (dd, ^3J = 8.1 Hz, ^4J = 1.5 Hz, 1 H, H_{ar}), 8.65–8.61 (m, 2 H, 2×H_{ar}), 8.28–8.21 (m, 1 H, H_{ar}), 8.17 (t, ^3J = 7.6 Hz, 1 H, H_{ar}), 8.07 (d, ^3J = 9.1 Hz, 1 H, H_{ar}), 7.91–7.72 (m, 6 H, 6×H_{ar}), 7.68 (t, ^3J = 7.6 Hz, 1 H, H_{ar}), 7.53 (d, ^3J = 9.0 Hz, 1 H, H_{ar}), 7.47 (t, ^3J = 7.7 Hz, 1 H, H_{ar}), 4.22 (s, 1 H, CH_3); ^{13}C NMR (125 MHz, DMSO-d$_6$, ppm: δ = 161.2 (C_q), 145.1 (C_q), 140.6 (C_q), 135.2 (C_q), 133.6 (C_q), 132.5 (CH), 132.2 (C_q), 131.3 (CH), 129.4 (CH), 129.3 (2×CH), 129.2 (2×CH), 128.2 (CH), 127.5 (CH), 127.4 (CH), 123.5 (C_q), 122.4 (CH), 122.2 (C_q), 121.1 (CH), 119.9 (CH), 119.6 (C_q), 116.2 (CH), 114.8 (C_q), 113.2 (CH), 42.5 (CH_3); IR (ATR, cm^{-1}): $\tilde{\nu}$ = 3335 (vw), 1587 (m), 1524 (w), 1486 (w), 1458 (w), 1395 (m), 1359 (w), 1287 (w), 1182 (w), 1015 (m), 867 (w), 818 (w), 797 (w), 752 (s), 710 (m); UV/Vis [THF, nm (mol^{-1}dm^{3}cm^{-1})]: λ_{max} (ε) = 408 (13,300), 326 (30,100), 218 (57,900); fluorescence (THF, nm): λ_{ex} = 330; λ_{em} = 526; MS (ESI): *m/z* (%) = 467 (0.6) [$C_{26}H_{19}N_2{}^+BF_4{}^-$]$^+$, 360 (28) [M+1]$^+$, 359 (100) [M$^+$], 345 (6) [M+1−$CH_3$]$^+$; HRMS (ESI): *m/z* calcd. for $C_{26}H_{19}N_2{}^+$: 359.1537 [M]$^+$; found: 359.1540.

7,8-Dimethyl-5-((4-phenyl-1*H*-1,2,3-triazol-1-yl)methyl)-9*H*-indolo[3,2-*a*]phenanthridine (11)

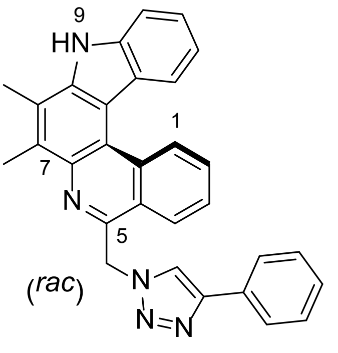

Following a published protocol,[40] 5-(azidomethyl)-7,8-dimethyl-9*H*-indolo[3,2-*a*]phenanthridine (**9e**; 60.4 mg, 172 µmol, 1.00 equiv.), phenylacetylene (21.9 mg, 24 µL, 214 µmol, 1.25 equiv.), $CuSO_4·5H_2O$ (6.7 mg, 27 µmol, 0.16 equiv.) and (+)-sodium-L-ascorbate (11.4 mg, 58 µmol, 0.34 equiv.) were suspended in THF/CH_2Cl_2/H_2O (1.5 mL, 2:1:1) and stirred at room temperature for 67 h. CH_2Cl_2 (5 mL) and H_2O (5 mL) were added and the layers were separated. The aqueous layer was extracted with CH_2Cl_2 (3×20 mL), dried ($MgSO_4$) and the solvent was removed under reduced pressure. Purification (silica gel, $CH_2Cl_2 \rightarrow CH_2Cl_2$ +1% MeOH) yielded **11** (65.1 mg, 114 µmol, 84%) as yellow crystalline solid.

R_f = 0.31 (CH_2Cl_2 + 1% MeOH); ^{1}H NMR (500 MHz, DMSO-d$_6$, ppm): δ = 11.82 (s, 1 H, 9-NH), 9.26 (d, ^3J = 8.3 Hz, 1 H, H_{ar}), 8.72 (s, 1 H, 5'-H), 8.58–8.48 (m, 2 H, 2×H_{ar}), 8.03–7.95 (m, 1 H, H_{ar}), 7.94–7.83 (m, 3 H, 3×H_{ar}), 7.70 (d, ^3J = 8.0 Hz, 1 H, H_{ar}), 7.48–7.41 (m, 3 H, 3×H_{ar}), 7.36–7.30 (m, 1 H, H_{ar}), 7.26–7.20 (m, 1 H, H_{ar}), 6.49 (s, 2 H, CH_2), 2.68 (s, 3 H, CH_3), 2.63 (s, 3 H, CH_3); ^{13}C NMR (125 MHz, DMSO-d$_6$, ppm): δ = 148.1 (C_q), 146.3 (C_q), 139.7 (C_q), 139.5 (C_q), 137.4 (C_q), 132.3 (C_q), 131.8 (C_q), 131.0 (C_q), 129.0 (CH), 128.9 (2×CH), 127.7 (CH), 127.4 (CH), 126.5 (CH), 125.2 (2×CH), 124.7 (CH), 124.6 (CH), 123.4 (C_q), 123.2 (C_q), 123.1 (CH), 122.2 (C_q), 122.0 (CH), 118.8 (C_q), 118.6 (CH), 112.0 (C_q), 112.0

(CH), 53.1 (CH_2), 14.5 (CH_3), 13.6 (CH_3); IR (ATR, cm^{-1}): $\tilde{\nu}$ = 3277 (vw), 3145 (vw), 1587 (w), 1529 (w), 1440 (w), 1306 (w), 1229 (w), 1138 (w), 1076 (w), 1056 (w), 973 (w), 822 (w), 763 (m), 747 (m); MS (FAB): *m/z* (%) = 454 (8), 453 (5), 154 (100) (3-NBA), 89 (15); HRMS (FAB): *m/z* calcd. for $C_{30}H_{24}N_5^+$: 454.2026 [M+1]$^+$; found: 454.2027.

6,9-Diphenyl-9*H*-indolo[2,3-*k*]phenanthridine (12)

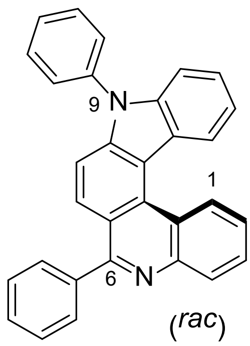

According to a patent procedure,[41] 6-phenyl-9*H*-indolo[2,3-*k*]phenanthridine (**3h**; 100 mg, 290 µmol, 1.00 equiv.), bromobenzene (67.4 mg, 429 µmol, 1.48 eq), [(*t*Bu)$_3$PH]BF_4 (13.7 mg, 46 µmol, 0.16 equiv.) and $Pd_2(dba)_3$ (7.0 mg, 8 µmol, 3 mol%) were added under argon to a freshly prepared suspension of NaH (40.3 mg, 1.68 µmol, 5.78 equiv.) and Na (40.0 mg, 1.74 mmol, 6.00 equiv.) in 5 mL anhydrous *o*-xylene and 1 mL *t*BuOH. The mixture was heated to 140 °C for 14.5 h. After cooling, it was quenched with sat. $NaHCO_3$ (20 mL) and H_2O (20 mL) and the aqueous layer was extracted with CH_2Cl_2 (3×30 mL). The combined organic layers were washed with H_2O (50 mL), dried ($MgSO_4$) and the solvent was removed under reduced pressure. Purification (silica gel, hexane/CH_2Cl_2 1:0 → 2:1 → 1:2 → 0:1) yielded **12** (83.0 mg, 197 µmol, 68%) as orange solid.

R_f = 0.12 (hexane/CH_2Cl_2 1:2); ^{1}H NMR (400 MHz, DMSO-d$_6$, ppm): δ = 9.26 (d, ^3J = 8.2 Hz, 1 H, H_{ar}), 8.79 (d, ^3J = 8.2 Hz, 1 H, H_{ar}), 8.20 (dd, ^3J = 8.2 Hz, ^4J = 1.4 Hz, 1 H, H_{ar}), 7.99 (d, ^3J = 9.0 Hz, 1 H, H_{ar}), 7.93–7.86 (m, 1 H, H_{ar}), 7.85–7.78 (m, 1 H, H_{ar}), 7.78–7.72 (m, 2 H, 2×H_{ar}), 7.72–7.66 (m, 4 H, 4×H_{ar}), 7.66–7.60 (m, 2 H, 2×H_{ar}), 7.60–7.53 (m, 4 H, 4×H_{ar}), 7.48–7.41 (m, 2 H, 2×H_{ar}); ^{1}H NMR (400 MHz, DMSO-d$_6$/$CDCl_3$, 2:1, ppm): δ = 9.29 (d, ^3J = 8.2 Hz, 1 H, H_{ar}), 8.72 (d, ^3J = 8.1 Hz, 1 H, H_{ar}), 8.32 (d, ^3J = 8.2 Hz, 1 H, H_{ar}), 8.01–7.95 (m, 2 H, 2×H_{ar}), 7.89 (t, ^3J = 7.6 Hz, 1 H, H_{ar}), 7.78–7.74 (m, 2 H, 2×H_{ar}), 7.72 (t, ^3J = 7.7 Hz, 2 H, 2×H_{ar}), 7.68–7.62 (m, 5 H, 5×H_{ar}), 7.60 (d, ^3J = 7.7 Hz, 2 H, 2×H_{ar}), 7.56 (t, ^3J = 7.7 Hz, 1 H, H_{ar}), 7.48–7.39 (m, 2 H, 2×H_{ar}); ^{13}C NMR (125 MHz, $CDCl_3$, ppm): δ = 159.1 (C_q), 145.1 (C_q), 142.0 (C_q), 136.3 (C_q) 135.7 (C_q), 134.6 (C_q), 132.3 (C_q), 131.6 (CH), 131.5 (CH), 130.9 (2×CH), 130.5 (2×CH), 129.6 (CH), 128.9 (CH), 128.8 (2×CH), 127.9 (2×CH), 127.5 (CH), 127.3 (CH), 126.9 (CH), 123.5 (C_q), 123.5 (CH), 123.3 (CH), 123.1 (C_q), 121.6 (CH), 119.2 (C_q), 116.6 (C_q), 113.6 (CH), 111.5 (CH); IR (ATR, cm^{-1}): $\tilde{\nu}$ = 3053 (vw), 3025 (vw), 2952 (vw), 2923 (vw), 2852 (vw), 1582 (w), 1502 (w), 1454 (w), 1392 (vw), 1372 (w), 1329 (w), 1283 (w), 1216 (vw), 1189 (w), 1153 (w), 1026 (vw), 972 (vw), 938 (vw), 827 (vw), 801 (vw), 774 (w), 746 (w); MS (ESI): *m/z* (%) = 421 (100) [M+1]$^+$, 422 (34) [M+2]$^+$, 423 (5) [M+3]$^+$. HRMS (ESI): *m/z* calcd. for $C_{31}H_{21}N_2^+$: 421.1699 [M+1]$^+$; found: 421.1693.

9-Butyl-6-phenyl-9*H*-indolo[2,3-*k*]phenanthridine (13)

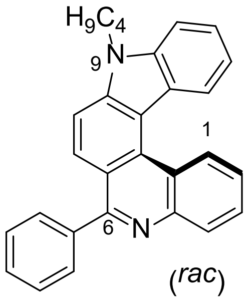

GP 4: 6-Phenyl-9*H*-indolo[2,3-*k*]phenanthridine (**3h**; 100 mg, 290 µmol, 1.00 equiv.), 1-bromobutane (182 mg, 1.33 mmol, 4.57 equiv.), KOH (325 mg, 5.79 mmol, 20.0 equiv.), 6 mL anhydrous DMF, 80 °C, 18 h; purification: silica gel, hexane/EtOAc, 1:0 → 4:1; **13**: yellow crystalline solid (101 mg, 251 µmol, 87%).

R_f = 0.38 (hexane/EtOAc 8:1); ^{1}H NMR (500 MHz, DMSO-d$_6$, ppm): δ = 9.26–9.19 (m, 1 H, H$_{ar}$), 8.73 (d, ^3J = 8.0 Hz, 1 H, H$_{ar}$), 8.19–8.14 (m, 1 H, H$_{ar}$), 8.08 (d, ^3J = 9.0 Hz, 1 H, H$_{ar}$), 8.00 (d, ^3J = 8.0 Hz, 1 H, H$_{ar}$), 7.90–7.83 (m, 2 H, 2×H$_{ar}$), 7.80–7.74 (m, 1 H, H$_{ar}$), 7.74–7.69 (m, 2 H, 2×H$_{ar}$), 7.61–7.56 (m, 4 H, 4×H$_{ar}$), 7.40–7.33 (m, 1 H, H$_{ar}$), 4.62 (t, ^3J = 7.2 Hz, 2 H, CH$_2$), 1.87–1.78 (m, 2 H, CH$_2$), 1.42–1.32 (m, 2 H, CH$_2$), 0.90 (t, ^3J = 7.4 Hz, 3 H, CH$_3$); ^{13}C NMR (125 MHz, DMSO-d$_6$, ppm): δ = 160.5 (C$_q$), 144.2 (C$_q$), 141.7 (C$_q$), 140.2 (2 C$_q$), 130.9 (C$_q$), 129.9 (2×CH), 129.2 (CH), 129.0 (CH), 128.6 (CH), 128.2 (2×CH), 126.3 (CH), 126.2 (CH), 125.9 (CH), 125.0 (CH), 122.7 (C$_q$), 122.5 (CH), 122.4 (C$_q$), 119.7 (C$_q$), 119.4 (CH), 114.5 (C$_q$), 111.7 (CH), 110.6 (CH), 40.4 (CH$_2$), 30.9 (CH$_2$), 19.8 (CH$_2$), 13.7 (CH$_3$); IR (ATR, cm^{-1}): $\tilde{\nu}$ = 3056 (vw), 2950 (w), 2927 (w), 2867 (w), 1609 (vw), 1581 (w), 1518 (w), 1455 (w), 1396 (w), 1357 (m), 1333 (w), 1287 (w), 1199 (w), 1157 (w), 1028 (w), 951 (w), 909 (w), 823 (w), 799 (w), 765 (m), 742 (m); MS (ESI): *m/z* (%) = 402 (31) [M+2]$^+$, 401 (100) [M+1]$^+$; HRMS (ESI): *m/z* calcd. for $C_{29}H_{25}N_2^+$: 401.2012 [M+1]$^+$; found: 401.2009.

9-Hexyl-6-phenyl-9*H*-indolo[2,3-*k*]phenanthridine (14)

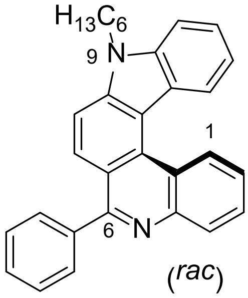

GP 4: 6-Phenyl-9*H*-indolo[2,3-*k*]phenanthridine (**3h**; 401 mg, 1.16 mmol, 1.00 equiv.), 1-bromohexane (743 mg, 4.50 mmol, 3.87 equiv.), KOH (1.30 g, 23.2 mmol, 19.9 equiv.), 24 mL anhydrous DMF, 80 °C, 19 h; purification: silica gel, hexane/EtOAc, 1:0 → 6:1 → 4:1 and hexane/EtOAc, 1:0 → 10:1; **14**: yellow crystalline solid (412 mg, 962 µmol, 93%).

R_f = 0.39 (hexane/EtOAc 4:1); ^{1}H NMR (500 MHz, DMSO-d$_6$, ppm): δ = 9.22 (d, ^3J = 8.2 Hz, 1 H, H$_{ar}$), 8.72 (d, ^3J = 8.1 Hz, 1 H, H$_{ar}$), 8.17 (dd, ^3J = 8.2 Hz, ^4J = 1.4 Hz, 1 H, H$_{ar}$), 8.06 (d, ^3J = 9.0 Hz, 1 H, H$_{ar}$), 8.00 (d, ^3J = 9.0 Hz, 1 H, H$_{ar}$), 7.89–7.83 (m, 2 H, 2×H$_{ar}$), 7.79–7.74 (m, 1 H, H$_{ar}$), 7.73–7.68 (m, 2 H, 2×H$_{ar}$), 7.63–7.56 (m, 4 H, 4×H$_{ar}$), 7.41–7.31 (m, 1 H, H$_{ar}$), 4.60 (t, ^3J = 7.2 Hz, 2 H, CH$_2$), 1.83 (quint., ^3J = 7.4 Hz, 2 H, CH$_2$), 1.40–1.31 (m, 2 H, CH$_2$), 1.30–1.17 (m, 4 H, 2×CH$_2$), 0.80 (t, ^3J = 7.1 Hz, 3 H, CH$_3$); ^{13}C NMR (125 MHz, DMSO-d$_6$, ppm: δ = 160.6 (C$_q$), 144.3 (C$_q$), 141.6 (C$_q$), 140.2 (C$_q$), 140.2 (C$_q$), 130.9 (C$_q$), 129.8 (2×CH), 129.2 (CH), 129.1 (CH), 128.6 (CH), 128.2 (2×CH), 126.2 (CH), 126.2 (CH), 125.8 (CH), 125.0 (CH), 122.7 (C$_q$), 122.5 (CH), 122.4 (C$_q$), 119.7 (C$_q$), 119.3 (CH), 114.5 (C$_q$), 111.7 (CH), 110.6 (CH), 42.6 (CH$_2$), 30.9 (CH$_2$), 28.7 (CH$_2$), 26.1 (CH$_2$), 22.0 (CH$_2$), 13.8 (CH$_3$); IR (ATR, cm^{-1}): $\tilde{\nu}$ = 3053 (vw), 2951 (w), 2924 (w), 2852 (w), 1611 (vw), 1581 (w), 1517 (w), 1455 (w), 1398 (w), 1353 (m), 1195 (w), 1157 (w), 1132 (w), 1028 (w), 971 (w), 920 (vw), 846 (vw), 798 (w), 770 (w), 743 (m); MS (ESI): *m/z* (%) = 431 (5) [M+3]$^+$, 430 (33) [M+2]$^+$, 429 (100) [M+1]$^+$, 374 (8) [M+3–$C_4H_9^{\cdot}$], 373 (27) [M+2–$C_4H_9^{\cdot}$]; HRMS (ESI): *m/z* calcd. for $C_{31}H_{29}N_2^+$: 429.2325 [M+1]$^+$; found: 429.2322.

9-Hexyl-7,8-dimethyl-5-phenyl-9*H*-indolo[3,2-*a*]phenanthridine

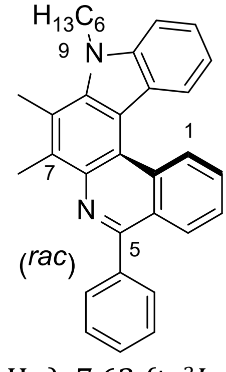

GP 4: 7,8-Dimethyl-5-phenyl-9*H*-indolo[3,2-*a*]phenanthridine (**9h**; 50.3 mg, 135 µmol, 1.00 equiv.), 1-bromohexane (92.1 mg, 78 µL, 558 µmol, 4.13 equiv.), KOH (170 mg, 3.03 mmol, 22.4 equiv.) in 4 mL anhydrous DMF; 80 °C, 19 h; purification: silica gel, hexane/EtOAc, 1:0 → 10:1 → 6:1); product: yellow crystalline solid (47.8 mg, 105 µmol, 78%).

m.p. 263–264 °C; R_f = 0.73 (hexane/EtOAc, 4:1); ^{1}H NMR (400 MHz, DMSO-d$_6$, ppm): δ = 9.13 (d, ^3J = 8.3 Hz, 1 H, H$_{ar}$), 8.51 (d, ^3J = 8.1 Hz, 1 H, H$_{ar}$), 8.17 (d, ^3J = 8.2 Hz, 1 H, H$_{ar}$), 7.96–7.90 (m, 2 H, 2×H$_{ar}$), 7.87 (t, ^3J = 7.7 Hz, 1 H, H$_{ar}$), 7.78 (d, ^3J = 8.3 Hz, 1 H, H$_{ar}$), 7.73 (t, ^3J = 6.9 Hz, 1 H, H$_{ar}$), 7.63 (t, ^3J = 7.3 Hz, 2 H, 2×H$_{ar}$), 7.60–7.54 (m, 1 H, H$_{ar}$), 7.50 (t, ^3J = 7.7 Hz, 1 H, H$_{ar}$), 7.24 (t, ^3J = 7.5 Hz, 1 H, H$_{ar}$), 4.71 (t, ^3J = 7.9 Hz, 2 H, CH$_2$), 2.93 (s, 3 H, CH$_3$), 2.91 (s, 3 H, CH$_3$), 1.88–1.76 (m, 2 H, CH$_2$), 1.49–1.35 (m, 2 H, CH$_2$), 1.35–1.22 (m, 4 H, 2×CH$_2$), 0.85 (t, ^3J = 6.9 Hz, 3 H, CH$_3$); ^{13}C NMR (125 MHz, DMSO-d$_6$, ppm: δ = 154.6 (C$_q$), 141.0 (C$_q$), 139.4 (C$_q$), 139.1 (C$_q$), 137.6 (C$_q$), 133.9 (C$_q$), 132.0 (C$_q$), 129.9 (2×CH), 128.6 (CH), 128.6 (CH), 128.5 (2×CH), 127.4 (CH), 127.1 (CH), 126.3 (CH), 125.0 (CH), 123.8 (C$_q$), 122.9 (C$_q$), 122.3 (C$_q$), 121.6 (CH), 118.8 (CH), 117.6 (C$_q$), 113.7 (C$_q$), 110.6 (CH), 45.1 (CH$_2$), 30.9 (CH$_2$), 30.3 (CH$_2$), 25.9 (CH$_2$), 22.1 (CH$_2$), 16.4 (CH$_3$), 14.1 (CH$_3$), 13.8 (CH$_3$); IR (ATR, cm^{-1}): $\tilde{\nu}$ = 3055 (vw), 2955 (w), 2922 (w), 2868 (w), 2849 (w), 1606 (vw), 1552 (w), 1516 (vw), 1444 (w), 1354 (w), 1316 (m), 1184 (w), 1155 (w), 1116 (w), 1078 (w), 1030 (w), 958 (w), 784 (w), 771 (w), 761 (w), 745 (m), 730 (m); MS (ESI): *m/z* (%) = 459 (6) [M+3]$^+$, 458 (36) [M+2]$^+$, 457 (100) [M+1]$^+$; HRMS (ESI): m/z calcd. for $C_{33}H_{33}N_2^+$: 457.2638 [M+1]$^+$, found: 457.2635.

9-Hexyl-6-(pyridin-2-yl)-9*H*-indolo[2,3-*k*]phenanthridine (16)

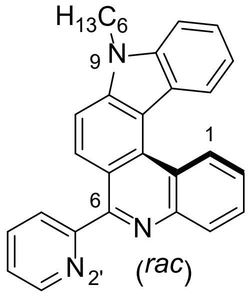

GP 4: 6-(Pyridine-2-yl)-9*H*-indolo[2,3-*k*]phenanthridine (**3l**, 307 mg, 887 µmol, 1.00 equiv.), 1-bromohexane (572 mg, 3.47 mmol, 3.90 equiv.), KOH (1.05 g, 18.7 mmol 21.1 equiv.) in 25 mL anhydrous DMF, 80 °C, 16 h; purification: silica gel, hexane/EtOAc, 1:0 → 5:1 → 2:1; **16**: orange crystalline solid (219 mg (634 µmol, 71%).

R_f = 0.36 (hexane/EtOAc 2:1); ^{1}H NMR (500 MHz, DMSO-d$_6$, ppm): δ = 9.26–9.20 (m, 1 H, H$_{ar}$), 8.82–8.78 (m, 1 H, H$_{ar}$), 8.72 (d, ^3J = 8.1 Hz, 1 H, H$_{ar}$), 8.37 (d, ^3J = 9.0 Hz, 1 H, H$_{ar}$), 8.21 (dd, ^3J = 8.2 Hz, ^4J = 1.3 Hz, 1 H, H$_{ar}$), 8.11–8.05 (m, 2 H, H$_{ar}$), 8.04–8.00 (m, 1 H, H$_{ar}$), 7.91–7.85 (m, 2 H, 2×H$_{ar}$), 7.84–7.77 (m, 1 H, H$_{ar}$), 7.63–7.57 (m, 2 H, 2×H$_{ar}$), 7.39–7.33 (m, 1 H, H$_{ar}$), 4.62 (t, ^3J = 7.2 Hz, 2 H, CH$_2$), 1.84 (quint., ^3J = 7.2 Hz, 2 H, CH$_2$), 1.41–1.32 (m, 2 H, CH$_2$), 1.31–1.19 (m, 4 H, 2×CH$_2$), 0.80 (t, ^3J = 7.1 Hz, 3 H, CH$_3$); ^{13}C NMR (125 MHz, DMSO-d$_6$, ppm: δ = 158.5 (C$_q$), 158.1 (C$_q$), 148.3 (CH), 144.1 (C$_q$), 141.6 (C$_q$), 140.1 (C$_q$), 137.2 (CH), 131.0 (C$_q$), 129.2 (CH), 129.2 (CH), 126.6 (CH), 126.4 (CH), 125.8 (CH), 125.4 (CH), 125.2 (CH), 123.6 (CH), 123.0 (C$_q$), 122.5 (CH), 122.4 (C$_q$), 119.5 (C$_q$), 119.4 (CH), 114.2 (C$_q$), 111.7 (CH), 110.6 (CH), 42.6 (CH$_2$), 31.0 (CH$_2$), 28.7 (CH$_2$), 26.1 (CH$_2$), 22.0 (CH$_2$), 13.8 (CH$_3$); IR (ATR, cm^{-1}): $\tilde{\nu}$ = 3048 (vw), 2954 (w), 2922 (w), 2855 (w), 1614 (w), 1582 (m), 1525 (w), 1469 (m), 1429 (w), 1402 (w), 1367 (m), 1240 (w), 1197 (w), 1167 (w), 1037 (w), 994 (w), 924 (w), 815 (w), 777 (m), 744 (m), 728 (m), 703 (m); MS (ESI): *m/z* (%) = 431 (32) [M+2]$^+$, 430 (100) [M+1]$^+$; HRMS (ESI): *m/z* calcd. for $C_{30}H_{28}N_3^+$: 430.2278 [M+1]$^+$; found: 430.2274.

9-Hexyl-6-(naphthalen-1-yl)-9-indolo[2,3-*k*]phenanthridine (17)

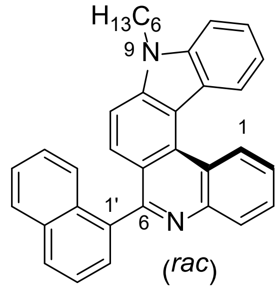

GP 4: 6-(Naphthalene-1-yl)-9*H*-indolo[2,3-*k*]phenanthridine (**3n**; 199 mg, 504 µmol, 1.00 equiv.), 1-bromohexane (153 mg, 130 µL, 927 µmol, 1.84 equiv.), KOH (570 mg, 10.2 mmol, 20.2 equiv.), 10 mL anhydrous DMF, 80 °C, 40 h; purification: silica gel, hexane/EtOAc, 1:0 → 100:1 → 10:1 → 4:1; **17**: yellow solid (198 mg, 415 µmol, 82%).

R_f = 0.42 (hexane/EtOAc 4:1); ^{1}H NMR (500 MHz, DMSO-d$_6$, ppm): δ = 9.30 (d, ^3J = 7.8 Hz, 1 H, H$_{ar}$), 8.79 (d, ^3J = 8.2 Hz, 1 H, H$_{ar}$), 8.20–8.13 (m, 2 H, 2×H$_{ar}$), 8.09 (d, ^3J = 1 H, H$_{ar}$), 7.92 (d, ^3J = 9.0 Hz, 1 H, H$_{ar}$), 7.90–7.80 (m, 3 H, 3×H$_{ar}$), 7.76–7.70 (m, 1H, H$_{ar}$), 7.70–7.65 (m, 1 H, H$_{ar}$), 7.61 (t, ^3J = 7.7 Hz, 1 H, H$_{ar}$), 7.57–7.51 (m, 1 H, H$_{ar}$), 7.50 (d, ^3J = 8.9 Hz, 1 H, H$_{ar}$), 7.39 (t, ^3J = 7.6 Hz, 1 H, H$_{ar}$), 7.35–7.30 (m, 1 H, H$_{ar}$), 7.26 (t, ^3J = 8.5 Hz, 1 H, H$_{ar}$), 4.55 (t, ^3J = 7.2 Hz, 2 H, CH$_2$), 1.79 (quint., ^3J = 7.3 Hz, 2 H, CH$_2$), 1.32 (quint., ^3J = 7.4 Hz, 2 H, CH$_2$), 1.27–1.13 (m, 4 H, 2×CH$_2$), 0.77 (t, ^3J = 7.0 Hz, 3 H, CH$_3$); ^{13}C NMR (125 MHz, DMSO-d$_6$, ppm): δ = 160.1 (C$_q$), 144.5 (C$_q$), 141.7 (C$_q$), 140.2 (C$_q$), 137.8 (C$_q$), 133.1 (C$_q$), 131.8 (C$_q$), 130.4 (C$_q$), 129.4 (CH), 129.2 (CH), 128.5 (CH), 128.3 (CH), 127.2 (CH), 126.5 (CH), 126.5 (CH), 126.2 (CH), 126.1 (CH), 125.9 (CH), 125.7 (CH), 125.4 (CH), 125.2 (CH), 123.0 (C$_q$), 122.6 (CH), 122.4 (C$_q$), 121.2 (C$_q$), 119.4 (CH), 114.5 (C$_q$), 111.9 (CH), 110.6 (CH), 42.6 (CH$_2$), 30.9 (CH$_2$), 28.7 (CH$_2$), 26.1 (CH$_2$), 22.0 (CH$_2$), 13.8 (CH$_3$); IR (ATR, cm^{-1}): $\tilde{\nu}$ = 3047 (vw), 2925 (w), 2853 (w), 1582 (w), 1519 (w), 1465 (w), 1400 (w), 1349 (m), 1256 (w), 1194 (w), 1158 (w), 1033 (w), 955 (w), 798 (w), 772 (m), 746 (m), 708 (w); MS (ESI): *m/z* (%) = 480 (38) [M+2]$^+$, 479 (100) [M+1]$^+$; HRMS (ESI): *m/z* calcd. for $C_{35}H_{31}N_2^+$: 479.2582 [M+1]$^+$; found: 479.2475.

9-Hexyl-6-(2-(4,4,5,5-tetramethyl-1,3,2-dioxaborolan-2-yl)phenyl)-9*H*-indolo[2,3-*k*]phenanthridine (19)

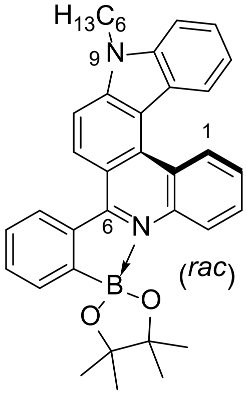

Following a published protocol,[45] BBr$_3$ (1M in CH$_2$Cl$_2$, 1.4 mL, 351 mL, 1.40 mmol, 3.08 equiv.) was dropwise added under argon to a solution of 9-hexyl-6-phenyl-9*H*-indolo[2,3-*k*]phenanthridine (**14**; 195 mg, 455 µmol, 1.00 equiv.) and DIPEA (67.8 mg, 89 µL, 525 µmol, 1.15 equiv.) in 20 mL anhydrous CH$_2$Cl$_2$. The mixture was heated to 50 °C for 4 h. After cooling slightly down, Et$_3$N (472 mg, 647 µL, 4.66 mmol, 10.3 equiv.) followed by pinacol (232 mg, 1.96 mmol, 4.32 equiv.) were added. The mixture was heated to 50 °C for another 16 h. H$_2$O (20 mL) was added, the layers were separated, and the aqueous layer was extracted with CH$_2$Cl$_2$ (3×20 mL), dried (MgSO$_4$) and the solvent was removed under reduced pressure. Purification (silica gel, hexane/EtOAc, 1:0 → 10:1 → 6:1) yielded **19** (69.5 mg, 136 µmol, 23%, 30% brsm) as yellow crystalline solid.

R_f = 0.34 (hexane/EtOAc 4:1); ^{1}H NMR (400 MHz, CDCl$_3$, ppm): δ = 9.35 (d, ^3J = 8.2 Hz, 1 H, H$_{ar}$), 8.89 (d, ^3J = 8.2 Hz, 1 H, H$_{ar}$), 8.34 (bs, 1 H, H$_{ar}$), 7.95 (d, ^3J = 7.4 Hz, 1 H, H$_{ar}$), 7.83 (d, ^3J = 8.9 Hz, 2 H, 2×H$_{ar}$), 7.76–7.48 (m, 7 H, 7×H$_{ar}$), 7.36 (t, ^3J = 7.5 Hz, 1 H, H$_{ar}$), 4.48 (t, ^3J= 7.4 Hz, 2 H, CH$_2$), 1.93 (quint., ^3J = 7.5 Hz, 2 H, CH$_2$), 1.43 (quint., ^3J = 7.3 Hz, ^3J = 6.9 Hz, 2 H, CH$_2$), 1.39–1.22 (m, 4 H, 2×CH$_2$), 0.86 (t, ^3J = 6.9 Hz, 3 H, CH$_3$), 0.73 (s, 12 H, 4×CH$_3$); ^{13}C NMR (100 MHz, CDCl$_3$, ppm): δ = 162.6 (C$_q$), 145.1 (C$_q$), 143.5 (C$_q$), 142.2 (C$_q$), 140.5 (C$_q$), 134.9 (CH), 131.8 (C$_q$), 130.4 (CH), 129.4 (CH), 129.0 (CH), 128.5 (CH), 128.0 (CH),

127.3 (CH), 126.8 (CH), 125.7 (CH), 124.7 (CH), 123.6 (C_q), 123.5 (C_q), 123.4 (CH), 121.9 (C_q), 119.3 (CH), 115.6 (C_q), 110.3 (CH), 109.6 (CH), 83.3 (2×OC_q), 43.4 (CH_2), 31.6 (CH_2), 29.0 (CH_2), 26.9 (CH_2), 24.3 (4×CH_3), 22.5 (CH_2), 14.0 (CH_3)*; IR (ATR, cm^{-1}): $\tilde{\nu}$ = 3054 (vw), 2954 (w), 2926 (w), 2855 (w), 1583 (w), 1519 (vw), 1465 (w), 1348 (m), 1142 (w), 1113 (w), 1081 (w), 1059 (w), 1034 (w), 961 (w), 859 (w), 824 (vw), 800 (w), 771 (w), 746 (m), 709 (w);UV/Vis [THF, nm ($mol^{-1}dm^3cm^{-1}$)]: λ_{max} (ε) = 308 (45,200), 226 (58,500); fluorescence (THF, nm): λ_{ex} = 330; λ_{em} = 429, 408; MS (ESI): *m/z* (%) = 557 (7) $[M+3]^+$, 556 (37) $[M+2]^+$, 555 (100) $[M+1]^+$, 554 (22) $[M]^+$; HRMS (ESI): *m/z* calcd. for $C_{37}H_{40}BN_2O_2^+$: 555.3177 $[M+1]^+$; found: 555.3186. *C_q–B is not visible, most likely due to quadrupolar broadening.[45]

6-[2-(Dimethylboranyl)phenyl]-9-hexyl-9*H*-indolo[2,3-*k*]phenanthridine (20)

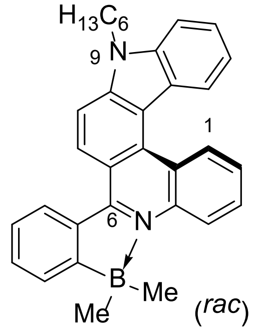

Following a published protocol,[45] BBr_3 (1.4 mL, 1M in CH_2Cl_2, 351 mg, 1.40 mmol, 4.86 equiv.) were slowly added under argon to a solution of 9-hexyl-6-phenyl-9*H*-indolo[2,3-*k*]phenanthridine (**14**; 123 mg, 288 µmol, 1.00 equiv.) and DIPEA (76.0 mg, 100 µL, 588 µmol, 2.04 equiv.) in 10 mL anhydrous CH_2Cl_2, and heated to 50 °C for 3.5 h. Then, Et_3N (0.5 mL, 365 mg, 3.61 mmol, 12.5 equiv.) followed by $AlMe_3$ (1.5 mL, 2M in toluene, 216 mg, 3.00 mmol, 10.4 equiv.) were added. The mixture was stirred at rt for 15.5 h. It was carefully quenched with H_2O (20 mL) and extracted with CH_2Cl_2 (3 × 50 mL). Combined organic layers were washed with H_2O (2×50 mL), dried ($MgSO_4$) and the solvent was removed under reduced pressure. Twofold purification (silica gel, hexane/EtOAc, 1:0 → 100:1 → 10:1) yielded **20** (56.9 mg, 122 µmol, 42%) as yellow crystalline solid.

R_f = 0.40 (hexane/EtOAc 8:1); ^{1}H NMR (400 MHz, DMSO-d_6, ppm): δ =9.30 (t, ^3J = 8.7 Hz, 2 H, 2×H_{ar}), 8.71 (d, ^3J = 8.6 Hz, 1 H, H_{ar}), 8.63 (d, ^3J = 8.1 Hz, 2 H, 2×H_{ar}), 8.32 (d, ^3J = 9.2 Hz, 1 H, H_{ar}), 8.00 (t, ^3J = 7.9 Hz, 1 H, H_{ar}), 7.94 (d, ^3J = 8.3 Hz, 1 H, H_{ar}), 7.82 (t, ^3J = 7.6 Hz, 1 H, H_{ar}), 7.69 (d, ^3J = 7.2 Hz, 1 H, H_{ar}), 7.65 (t, ^3J = 7.7 Hz, 1 H, H_{ar}), 7.51 (t, ^3J = 7.2 Hz, 1 H, H_{ar}), 7. 41–7.35 (m, 2 H, 2×H_{ar}), 4.72 (t, ^3J = 7.2 Hz, 2 H, CH_2), 1.90 (quint., ^3J = 7.3 Hz, 2 H, CH_2), 1. 49–1.36 (m, 2 H, CH_2), 1.35–1. 19 (m, 4 H, 2×CH_2), 0.83 (t, ^3J = 7.0 Hz, 3 H, CH_3), 0.29 (s, 6 H, 2×CH_3); ^{13}C NMR (100 MHz, DMSO-d_6, ppm): δ = 157.1 (C_q), 142.6 (C_q), 140.3 (C_q), 137.1 (C_q), 136.3 (C_q), 133.0 (C_q), 130.1 (CH), 130.1 (CH), 128.2 (CH), 127.3 (CH), 127.0 (CH), 126.5 (CH), 125.6 (CH), 125.5 (CH), 125.5 (CH), 123.1 (CH), 122.9 (C_q), 122.6 (CH), 122.3 (C_q), 120.0 (CH), 117.6 (C_q), 114.6 (C_q), 113.0 (CH), 110.9 (CH), 42.8 (CH_2), 30.9 (CH_2), 28.7 (CH_2), 26.1 (CH_2), 22.0 (CH_2), 13.8 (2×CH_3), 10.5 (CH_3)*; IR (ATR, cm^{-1}): $\tilde{\nu}$ = 3047 (vw), 2918 (m), 1582 (m), 1494 (w), 1458 (m), 1384 (m), 1329 (m), 1298 (m), 1206 (w), 1151 (m), 1121 (w), 1008 (m), 990 (w), 944 (w), 816 (w), 794 (w), 770 (w), 737 (m); MS (ESI): *m/z* (%) = 485 (25)$[C_{33}H_{33}BN_2O+1]^+$, 484 (6) $[C_{33}H_{33}BN_2O]^+$, 467 (8) $[M-1]^+$, 453 (13) $[M-CH_3^{\cdot}]^+$, 445 (10) $[C_{33}H_{33}BN_2O_2+2-C_4H_9]^+$, 444 (34) $[C_{33}H_{33}BN_2O_2+1-C_4H_9]^+$, 443 (100) $[C_{33}H_{33}BN_2O_2-C_4H_9]^+$, 203 (14); HRMS (ESI): *m/z* calcd. for $C_{33}H_{34}BN_2^+$: 469.2810 $[M+1]^+$; found: 469.2810. *C_q–B is not visible, most likely due to quadrupolar broadening.[45]

3.6 2-Bromo-indolo[2,3-*k*]phenanthridine 6

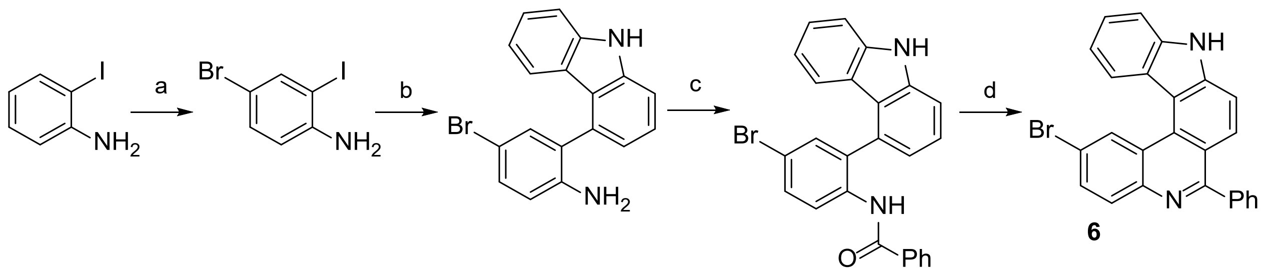

Scheme S1. Synthesis of 2-bromo-substituted [2,3-*k*]-IP **6**. Conditions: a) NBS, CH_2Cl_2, 0 °C to rt, 18.5 h (81%); b) 4-(4,4,5,5-tetramethyl-1,3,2-dioxaborolane-2-yl)-9*H*-carbazol, cat. $Pd(PPh_3)_4$, K_3PO_4, dioxane/H_2O (6:1), 100 °C, 19 h (53%); c) PhCOCl, CH_2Cl_2, 0 °C, 1 h, then rt, overnight (85%); d) $PhNO_2$, 150 °C, 15 h (71%).

4-Bromo-2-iodoaniline

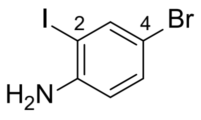

According to a published protocol,[69] NBS (1.64 g, 9.22 mmol, 1.05 equiv.) were added to a solution of 2-iodoaniline (1.93 g, 8.81 mmol, 1.00 equiv.) in 10 mL CH_2Cl_2 and stirred for 66 h at room temperature. H_2O (30 mL) was added, and the aqueous layer was extracted with CH_2Cl_2 (3×20 mL). The combined organic layers were washed with saturated $Na_2S_2O_5$ solution (20 mL), dried ($MgSO_4$), concentrated under reduced pressure. Recrystallization from hexane yielded **23** (2.12 g, 7.13 mmol, 81%) as pale orange crystalline solid. The NMR data are in agreement with published data.[69]

^{1}H NMR (400 MHz, $CDCl_3$, ppm): δ = 7.73 (d, ^4J = 2.2 Hz, 1 H, 3-H), 7.22 (dd, ^3J = 8.5 Hz, 1 H, 5-H), 6.62 (d, ^3J = 8.5 Hz, 1 H, 6-H).

4-Bromo-2-(9*H*-carbazol-4-yl)aniline

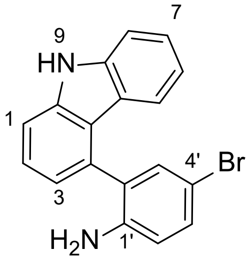

Following a patent protocol,[29] $Pd(PPh_3)_4$ (66.3 mg, 57.4 µmol, 5 mol%) was added under positive argon pressure to a degassed solution (ultrasonication, 15 min) of 4-bromo-2-iodoaniline (351 mg, 1.18 mmol, 1.05 equiv.), 4-(4,4,5,5-tetramethyl-1,3,2-dioxaborolane-2-yl)-9*H*-carbazol (66% w/w, 501 mg, 1.12 mmol, 1.00 equiv.) and K_3PO_4 (478 mg, 2.25 mmol, 2.01 equiv.) in dioxane/H_2O (15.3 mL, 6:1). The mixture was stirred at 100 °C for 18.5 h. After cooling H_2O (20 mL) was added, the layers were separated, and the aqueous layer was extracted with CH_2Cl_2 (3×20 mL). The combined organic layers were dried ($MgSO_4$), concentrated under reduced pressure, and purified by column chromatography (silica gel, hexane/EtOAc, 1:0 → 4:1 → 2:1 and pentane/Et_2O, 1:0 → 2:1) to yield (202 mg, 599 µmol, 53%) as an orange oil, which solidified.

R_f = 0.21 (hexane/EtOAc 4:1); ^{1}H NMR (500 MHz, DMSO-d$_6$, ppm): δ = 11.43 (s, 1 H, 9-NH), 7.52 (dd, ^3J = 8.1 Hz, ^4J = 1.0 Hz, 1 H, H_{ar}), 7.50–7.44 (m, 2 H, 2×H_{ar}), 7.36–7.30 (m, 2 H, 2×H_{ar}), 7.17 (d, ^3J = 7.8 Hz, 1 H, H_{ar}), 7.17 (d, ^4J = 2.5 Hz, 1 H, H_{ar}), 6.99–6.93 (m, 2 H, 2×H_{ar}), 6.82 (d, ^3J = 8.6 Hz, 1 H, H_{ar}), 4.68 (s, 2 H, NH_2); ^{13}C NMR (125 MHz, DMSO-d$_6$, ppm): δ = 145.1 (C_q), 140.1 (C_q), 139.9 (C_q), 132.1 (C_q), 131.8 (CH), 131.0 (CH), 127.2 (C_q), 125.8

(CH), 125.4 (CH), 121.8 (C_q), 121.4 (CH), 120.0 (C_q), 119.9 (CH), 118.4 (CH), 116.3 (CH), 110.8 (CH), 110.5 (CH), 106.5 (C_q); IR (ATR, cm^{-1}): $\tilde{\nu}$ = 3398 (w), 3052 (vw), 1603 (w), 1481 (w), 1454 (w), 1428 (w), 1401 (w), 1322 (w), 1280 (w), 1220 (w), 1149 (w), 1117 (w), 999 (vw), 947 (vw), 812 (w), 797 (w), 749 (w), 728 (m); MS (FAB): *m/z* (%) = 339 (4) $[M+1]^+$, 338 (7) $[M]^+$, 308 (10), 307 (39), 289 (17), 154 (100) [3-NBA], 137 (68), 120 (11); HRMS (FAB): *m/z* calcd. for $C_{18}H_{13}{}^{79}BrN_2{}^+$: 336.0257 $[M]^+$; found: 336.0258.

N-(4-Bromo-2-(9*H*-carbazol-4-yl)phenyl)benzamide

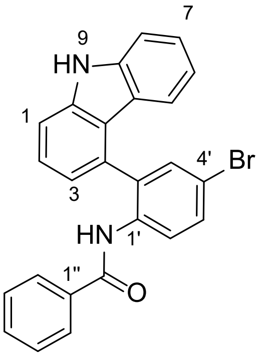

Similar to GP 2, with a cooled (0 °C) solution of 4-bromo-2-(9*H*-carbazol-4-yl)aniline (191 mg, 566 µmol, 1.00 equiv.) in anhydrous CH_2Cl_2 (20 mL), which was added dropwise to a cooled (0 °C) solution of benzoyl chloride (103 mg, 84 µL, 732 µmol, 1.29 equiv.) and Et_3N (73.9 mg, 101 µL, 730 µmol, 1.29 equiv.) in anhydrous CH_2Cl_2 (10 mL); purification: silica gel, pentane/Et_2O, 1:0 → 3:1 → 1:1; product: colorless crystalline solid (213 mg, 483 µmol, 85%).

R_f = 0.44 (pentane/Et_2O 1:1); ^1H NMR (500 MHz, DMSO-d_6, ppm): δ = 11.47 (s, 1 H, 9-NH), 9.21 (s, 1 H, NHCO), 7.96 (d, ^3J = 8.7 Hz, 1 H, H_{ar}), 7.76 (dd, ^3J = 8.7 Hz, ^4J = 2.4 Hz, 1 H, H_{ar}), 7.64 (d, ^4J = 2.4 Hz, 1 H, H_{ar}), 7.53 (dd, ^3J = 8.1 Hz, ^4J = 1.0 Hz, 1 H, H_{ar}), 7.50–7.46 (m, 1 H, H_{ar}), 7.46–7.42 (m, 1 H, H_{ar}), 7.39 (t, ^3J = 6.8 Hz, ^4J = 1.9 Hz, 1 H, H_{ar}), 7.34–7.29 (m, 1 H, H_{ar}), 7.26–7.20 (m, 4 H, 4×H_{ar}), 7.19–7.14 (m, 1 H, H_{ar}), 7.10–7.04 (m, 1 H, H_{ar}), 6.96–6.90 (m, 1 H, H_{ar}); ^{13}C NMR (125 MHz, DMSO-d_6, ppm): δ = 165.2 (CO), 140.1 (C_q), 140.0 (C_q), 137.1 (C_q), 135.2 (C_q), 134.4 (C_q), 132.8 (CH), 131.4 (CH), 131.1 (C_q), 131.0 (CH), 128.2 (2×CH), 127.0 (CH), 126.9 (2×CH), 125.6 (CH), 125.4 (CH), 121.5 (C_q), 121.2 (CH), 120.3 (CH), 119.8 (C_q), 118.4 (CH), 117.4 (C_q), 110.9 (CH), 110.9 (CH); IR (ATR, cm^{-1}): $\tilde{\nu}$ = 3388 (w), 3266 (w), 1658 (m), 1599 (w), 1569 (w), 1508 (m), 1454 (w), 1394 (m), 1308 (m), 1090 (w), 945 (w), 880 (w), 819 (w), 798 (w), 768 (w), 732 (m), 709 (m); MS (FAB): *m/z* (%) = 444 (10) $[M+4]^+$, 442 (48) $[M+2]^+$, 440 (37) $[M]^+$, 307 (25), 289 (15), 154 (100) [3-NBA], 105 (97); HRMS (FAB): *m/z* calcd. for $C_{25}H_{17}{}^{79}BrN_2O^+$: 440.0519 $[M]^+$; found: 440.0520.

2-Bromo-6-phenyl-9*H*-indolo[2,3-*k*]phenanthridine (6)

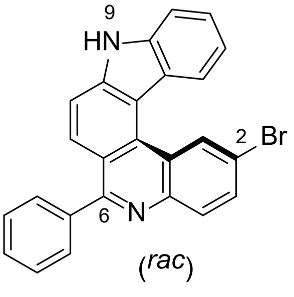

GP 3: *N*-(4-Bromo-2-(9*H*-carbazol-4-yl)phenyl)benzamide (150 mg, 340 µmol, 1.00 equiv.), $POCl_3$ (120 mg, 71 µL, 783 µmol, 2.30 equiv.), $PhNO_2$ (10 mL), 150 °C, 14 h. The solvent was removed under reduced pressure and the residue was suspended in MeOH (ca. 10 mL) and filtrated. The precipitate was dissolved in CH_2Cl_2 and Et_3N and filtrated (*Celite*® and silica gel, hexane → CH_2Cl_2). Removing the solvent under reduced pressure yielded **6** (102 mg, 240 µmol, 71%) as beige crystalline solid.

R_f = 0.27 (CH_2Cl_2); ^1H NMR (500 MHz, DMSO-d_6, ppm): δ = 12.39 (s, 1 H, 9-NH), 9.42 (d, ^4J = 2.2 Hz, 1 H, 1-H), 8.63 (d, ^3J = 8.2 Hz, 1 H, H_{ar}), 8.10 (d, ^3J = 8.7 Hz, 1 H, H_{ar}), 7.94 (d, ^3J = 8.9 Hz, 1 H, H_{ar}), 7.77 (d, ^3J = 8.1 Hz, 1 H, H_{ar}), 7.73–7.67 (m, 2 H, 2×H_{ar}), 7.64–7.58 (m, 3 H, 3×H_{ar}) 7.58–7.53 (m, 1 H, H_{ar}), 7.40–7.33 (m, 1 H, H_{ar}); ^{13}C NMR (125 MHz, DMSO-d_6, ppm): δ = 161.2 (C_q), 143.0 (C_q), 141.8 (C_q), 140.1 (C_q), 140.0 (C_q), 131.7 (CH), 131.2 (CH), 130.0 (C_q), 129.8 (2 CH), 128.7 (CH), 128.2 (2×CH), 126.2 (CH), 126.0 (CH), 124.4 (C_q),

122.4 (C_q), 121.9 (CH), 121.9 (CH), 119.9 (C_q), 119.3 (CH), 117.7 (C_q), 114.5 (C_q), 114.3 (CH), 112.5 (CH)*; IR (ATR, cm^{-1}): $\tilde{\nu}$ = 3120 (w), 3052 (w), 2946 (w), 2911 (w), 2824 (w), 1593 (w), 1519 (w), 1474 (w), 1454 (w), 1356 (m), 1273 (m), 1147 (w), 1069 (w), 1025 (w), 948 (w), 900 (w), 872 (w), 811 (m), 774 (m), 750 (m), 704 (m); MS (FAB): *m/z* (%) = 423 (34) $[M+1]^+$, 422 (10) $[M]^+$, 307 (32), 289 (15), 154 (100) [3-NBA], 120 (12); HRMS (FAB): *m/z* calcd. for $C_{25}H_{16}{}^{79}BrN_2{}^+$: 423.0491 $[M+1]^+$; found: 423.0490. *One CH signal is covered.

3.7 Modifications of Indolophenanthridine 6

2-Bromo-9-hexyl-6-phenyl-9*H*-indolo[2,3-*k*]phenanthridine (15)

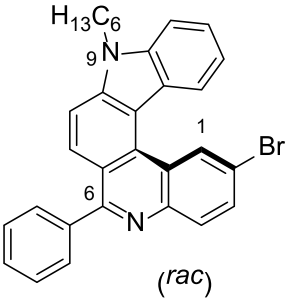

GP 4: 2-Bromo-6-phenyl-9*H*-indolo[2,3-*k*]phenanthridine (**6**; 412 mg, 972 µmol, 1.00 equiv.), 1-bromohexane (333 mg, 282 µL, 2.02 mmol, 2.07 equiv.), KOH (1.10 g, 19.6 mmol, 20.2 equiv.), 10 mL anhydrous DMF, 80 °C, 17.5 h; purification: silica gel, hexane/EtOAc, 1:0 → 10:1 → 5:1; **15**: yellow crystalline solid (473 mg, 993 µmol, 96%).

R_f = 0.59 (hexane/EtOAc 3:1); ^{1}H NMR (400 MHz, CD_2Cl_2, ppm): δ = 9.51 (d, ^4J = 2.2 Hz, 1 H, H_{ar}), 8.79 (d, ^3J = 8.2 Hz, 1 H, H_{ar}), 8.10 (d, ^3J = 7.5 Hz, 1 H, H_{ar}), 8.08 (d, ^3J = 7.8 Hz, 1 H, H_{ar}), 7.87 (dd, ^3J = 8.8 Hz, ^4J = 2.3 Hz, 1 H, H_{ar}), 7.76–7.68 (m, 3 H, 3×H_{ar}), 7.66–7.54 (m, 5 H, 5×H_{ar}), 7.41–7.35 (m, 1 H, H_{ar}), 4.45 (t, ^3J = 7.4 Hz, 2 H, CH_2), 1.92 (quint, ^3J = 7.5 Hz, 2 H, CH_2), 1.48–1.39 (m, 2 H, CH_2), 1.39–1.26 (m, 4 H, 2×CH_2), 0.87 (t, ^3J = 8.7 Hz, 3 H, CH_3); ^{13}C NMR (100 MHz, CD_2Cl_2, ppm): δ = 162.1 (C_q), 144.2 (C_q), 142.5 (C_q), 142.2 (C_q), 141.1 (C_q), 132.3 (CH), 131.6 (CH), 131.4 (C_q), 130.5 (2×CH), 129.6 (CH), 129.1 (CH), 128.7 (2×CH), 127.2 (CH), 126.5 (CH), 125.5 (C_q), 123.6 (C_q), 123.4 (CH), 121.2 (C_q), 120.0 (CH), 118.7 (C_q), 115.9 (C_q), 111.6 (CH), 110.4 (CH), 43.9 (CH_2), 32.1 (CH_2), 29.6 (CH_2), 27.4 (CH_2), 23.1 (CH_2), 14.3 (CH_3); IR (ATR, cm^{-1}): $\tilde{\nu}$ = 3049 (vw), 2956 (vw), 2853 (vw), 2922 (vw), 1581 (vw),1513 (vw), 1464 (w), 1397 (vw), 1360 (w), 1160 (w), 1065 (vw), 1025 (vw), 929 (vw), 905 (vw), 829 (vw), 806 (w), 751 (w); MS (ESI): *m/z* (%) = 511 (4) $[C_{31}H_{27}{}^{81}BrN_2+3]^+$, 510 (33) $[C_{31}H_{27}{}^{81}BrN_2+2]^+$, 509 (100) $[C_{31}H_{27}{}^{81}BrN_2+1]^+$, 508 (33) $[C_{31}H_{27}{}^{79}BrN_2+2]^+$, 507 (98) $[C_{31}H_{27}{}^{79}BrN_2+1]^+$,472 (15), 430 (9) $[C_{25}H_{22}BrN_2{}^{\cdot}+1-C_6H_5]^+$, 429 (29) $[C_{31}H_{27}N_2{}^{\cdot}+2-Br^{\cdot}]^+$; HRMS (ESI): *m/z* calcd. for $C_{31}H_{27}{}^{79}BrN_2{}^+$: 507.1430 $[M+1]^+$; found: 507.1431.

2-Azido-9-hexyl-6-phenyl-9*H*-indolo[2,3-*k*]phenanthridine (23)

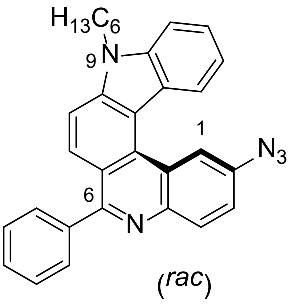

Following a published protocol,[40] 0.5 mL nBuLi solution (2.5M in hexane, 80.1 mg, 1.25 mmol, 2.99 equiv.) were slowly added under argon to a cooled (−78 °C) solution of 2-bromo-9-hexyl-6-phenyl-9*H*-indolo[2,3-*k*]phenanthridine (**15**; 213 mg, 419 µmol, 1.64 equiv.) in 20 mL anhydrous THF. The mixture was stirred at −78 °C for 1 h. *p*-toluenesulfonyl azide (135 mg, 686 µmol, 1.64 equiv.) in 1 mL anhydrous THF were added and the mixture was stirred over night while slowly warming to room temperature. Sat. NH_4Cl solution (30 mL) were added. The aqueous layer was extracted with EtOAc (3×40 mL). The combined organic layers were washed with H_2O (2×50 mL), dried ($MgSO_4$) and the solvent removed under reduced pressure. Twofold purification

(silica gel, hexane/EtOAc, 1:0 → 50:1 → 10:1 and 1:0 → 200:1 → 100:1) yielded **23** (141 mg, 300 µmol, 72%) as orange solid.

*R*f = 0.43 (hexane/EtOAc 4:1); ^{1}H NMR (400 MHz, DMSO-d$_6$, ppm): *δ* = 9.22–8.83 (m, 1 H, H$_{ar}$), 8.74–8.62 (m, 1 H, H$_{ar}$), 8.18–8.12 (m, 1 H, H$_{ar}$), 8.05–7.95 (m, 2 H, 2×H$_{ar}$), 7.87–7.80 (m, 1 H, H$_{ar}$), 7.77–7.71 (m, 1 H, H$_{ar}$), 7.71–7.66 (m, 2 H, 2×H$_{ar}$), 7.62–7.49 (m, 4 H, 4×H$_{ar}$), 7.36–7.29 (m, 1 H, H$_{ar}$), 4.55 (t, ^3J = 7.3 Hz, 2 H, CH$_2$), 1.79 (quint., ^3J = 7.2 Hz, 2 H, CH$_2$), 1.36–1.28 (m, 2 H, CH$_2$), 1.28–1.12 (m, 4 H, 2×CH$_2$), 0.77 (t, ^3J =7.2 Hz, 3 H, CH$_3$); ^{13}C NMR (125 MHz, CDCl$_3$, ppm): *δ* = 161.2 (C$_q$), 142.2 (C$_q$), 140.6 (C$_q$), 132.4 (C$_q$), 130.2 (2×CH), 129.3 (CH), 129.1 (C$_q$), 128.8 (CH), 128.5 (2×CH), 127.2 (CH), 126.9 (CH), 125.9 (CH), 125.0 (CH), 123.7 (C$_q$), 123.6 (C$_q$), 123.5 (CH), 120.4 (C$_q$), 119.4 (CH), 115.8 (C$_q$), 110.4 (CH), 109.7 (CH), 43.5 (CH$_2$), 31.6 (CH$_2$), 29.2 (CH$_2$), 27.1 (CH$_2$), 22.6 (CH$_2$), 14.1 (CH$_3$)*; IR (ATR, cm^{-1}): $\tilde{\nu}$ = 3053 (w), 2925 (m), 2853 (w), 2111 (m), 1612 (w), 1581 (m), 1517 (m), 1464 (m), 1398 (w), 1355 (s), 1331 (m), 1284 (m), 1158 (m), 1028 (w), 971 (w), 798 (m), 770 (m), 745 (s); MS (ESI): *m/z* (%) = 470 (4) [M+1]$^+$, 430 (34) [M+3−N$_3$]$^+$, 429 (100) [M+2−N$_3$]$^+$; HRMS (ESI): *m/z* calcd. for $C_{31}H_{28}N_3^+$: 470.2345 [M+1]$^+$; found: 470.2339. *Two C$_q$ signals are covered.

9-Hexyl-6-phenyl-2-((3-(1,2,2-triphenylvinyl)phenyl)ethinyl)-9*H*-indolo[2,3-*k*]phenanthridine (22)

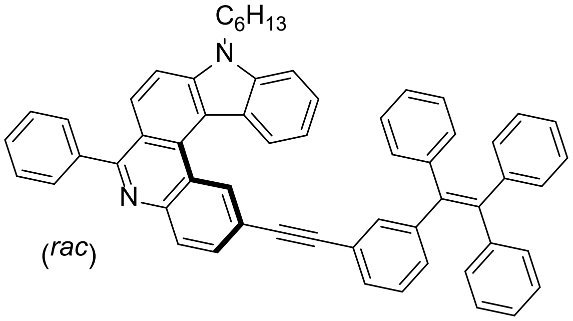

Following a published protocol,[47] PPh$_3$ (9.2 mg, 35 µmol, 0.18 equiv.) and CuI (7.0 mg, 37 µmol, 0.19 equiv.) were added under argon to a solution of 2-bromo-9-hexyl-6-phenyl-9*H*-indolo[2,3-*k*]phenanthridine (**15**; 100 mg, 198 µmol, 1.00 equiv.) and (2-(4-ethinylphenyl)ethen-1,1,2-triyl)tribenzene[70] (**21**, 111 mg, 311 µmol, 1.57 equiv.) in 9 mL THF/Et$_3$N (1:1) and degassed (ultrasonication) for 5 min. Then, PdCl$_2$(PPh$_3$)$_2$ (10.9 mg, 16 µmol, 8 mol%) were added, the mixture was degassed again for 5 min and heated to 60 °C for 41 h. After cooling, H$_2$O (30 mL) were added and the mixture was extracted with EtOAc (3 × 30 mL), washed with H$_2$O (50 mL), dried (MgSO$_4$). The solvent was removed under reduced pressure. Purification (silica gel, hexane/EtOAc, 1:0 → 100:1 → 10:1 → 4:1) yielded **22** (119 mg, 152 µmol, 77%) as yellow powder.

*R*f = 0.32 (hexane/EtOAc 4:1); ^{1}H NMR (400 MHz, DMSO-d$_6$, ppm): *δ* = 9.36 (d, ^4J = 2.2 Hz, 1 H, H$_{ar}$), 8.70 (d, ^3J = 8.2 Hz, 1 H, H$_{ar}$), 8.16 (d, ^3J = 8.5 Hz, 1 H, H$_{ar}$), 8.12 (d, ^3J = 9.1 Hz, 1 H, H$_{ar}$), 8.02 (d, ^3J = 9.0 Hz, 1 H, H$_{ar}$), 7.97–7.86 (m, 2 H, 2×H$_{ar}$), 7.75–7.69 (m, 2 H, 2×H$_{ar}$), 7.65–7.57 (m, 4 H, 4×H$_{ar}$), 7.42–7.33 (m, 3 H, 3×H$_{ar}$), 7.21–7.09 (m, 9 H, 9×H$_{ar}$), 7.05–6.95 (m, 8 H, 8×H$_{ar}$), 4.63 (t, ^3J = 7.1 Hz, 2 H, CH$_2$), 1.84 (quint., ^3J = 7.2 Hz, 2 H, CH$_2$), 1.42–1.29 (m, 2 H, CH$_2$), 1.32–1.15 (m, 4 H, 2×CH$_2$), 0.80 (t, ^3J = 7.1 Hz, 3 H, CH$_3$); ^{13}C NMR (100 MHz, DMSO-d$_6$, ppm): *δ* = 161.4 (C$_q$), 143.9 (C$_q$), 143.9 (C$_q$), 142.9 (C$_q$), 142.9 (C$_q$), 142.7 (C$_q$), 141.7 (C$_q$), 141.4 (C$_q$), 140.3 (C$_q$), 140.0 (C$_q$), 139.8 (C$_q$), 131.3 (CH), 131.1 (2×CH), 130.9 (2×CH), 130.7 (2×CH), 130.6 (2×CH), 130.6 (2×CH), 130.2 (C$_q$), 129.9 (2×CH), 129.6 (CH), 129.5 (CH), 128.7 (CH), 128.2 (2×CH), 128.0 (2×CH), 127.9 (2×CH), 127.8 (2×CH), 126.8 (CH), 126.7 (CH), 126.7 (CH), 126.3 (CH), 126.1 (CH), 122.7 (C$_q$), 122.2 (C$_q$), 122.0 (CH), 120.2 (C$_q$), 119.9 (C$_q$), 119.5 (CH), 118.5 (C$_q$), 114.4 (C$_q$), 112.2 (CH), 110.8 (CH), 89.9 (C$_q$), 89.8 (C$_q$), 42.6 (CH$_2$), 31.0 (CH$_2$), 28.7 (CH$_2$), 26.0 (CH$_2$), 22.0 (CH$_2$), 13.8 (CH$_3$); IR (ATR,

cm^{-1}): $\tilde{\nu}$ = 3051 (vw), 3022 (vw), 2924 (w), 2852 (vw), 1580 (vw), 1506 (vw), 1491 (vw), 1465 (vw), 1442 (vw), 1360 (w), 1330 (w), 1157 (w), 1073 (vw), 1027 (vw), 910 (vw), 837 (vw), 816 (w), 749 (w); MS (ESI): *m/z* (%) = 786 (4) $[M+4]^+$, 785 (20) $[M+3]^+$, 784 (65) $[M+2]^+$, 783 (100) $[M+1]^+$; HRMS (ESI): *m/z* calcd. for $C_{59}H_{47}N_2^+$: 783.3734 $[M+1]^+$; found: 783.3739.

9-Hexyl-6-phenyl-2-(4-phenyl-1*H*-1,2,3-triazol-1-yl)-9*H*-indolo[2,3-*k*]phenanthridine (24)

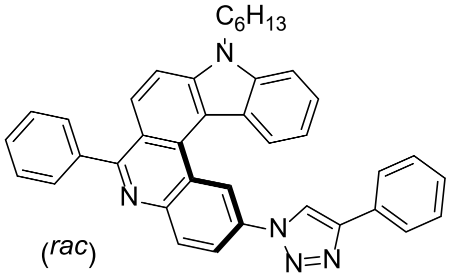

Following a published protocol,[40] $CuSO_4\cdot5H_2O$ (16.6 mg, 66 µmol, 0.29 equiv.) and 22.5 mg (+)-sodium-L-ascorbate (113 µmol, 0.50 equiv.) were added to a cooled (0 °C) suspension of 2-azido-9-hexyl-6-phenyl-9*H*-indolo[2,3-*k*]phenanthridine (**23**; 106 mg, 225 µmol, 1.00 equiv.) and ethynylbenzene (61.5 mg, 602 µmol, 2.68 equiv.) in 4 mL THF/H_2O (1:1) and stirred for 64 h, while slowly warming to room temperature. CH_2Cl_2 (5 mL) and H_2O (5 mL) were added. The mixture was extracted with CH_2Cl_2 (3 × 20 mL), dried ($MgSO_4$) and the solvent was removed under reduced pressure. Purification by chromatography (silica gel, hexane/EtOAc 1:0 → 100:1 → 50:1 → 10:1 → 4:1) yielded **24** (40.7 mg, 45 µmol, 20%) as yellow crystalline solid.

R_f = 0.27 (hexane/EtOAc 4:1); ^{1}H NMR (500 MHz, DMSO-d$_6$, ppm): δ =9.84–9.77 (m, 1 H, H_{ar}), 8.94 (d, ^3J = 8.2 Hz, 1 H, H_{ar}), 8.84 (bs, 1 H, H_{ar}), 8.44 (s, 1 H, H_{ar}), 8.28 (dd, ^3J = 8.9 Hz, ^4J = 2.4 Hz, 1 H, H_{ar}), 8.16 (d, ^3J = 8.9 Hz, 1 H, H_{ar}), 7.99–7.93 (m, 2 H, 2×H_{ar}), 7.85–7.76 (m, 3 H, 3×H_{ar}), 7.69–7.58 (m, 5 H, 5×H_{ar}), 7.53–7.45 (m, 3 H, 3×H_{ar}), 7.40 (t, ^3J = 7.4 Hz, 1 H, H_{ar}), 4.52 (t, ^3J = 7.3 Hz, 2 H, CH_2), 1.97 (quint., ^3J = 7.4 Hz, 2 H, CH_2), 1.46 (quint., ^3J = 7.2 Hz, 2 H, CH_2), 1.41–1.22 (m, 4 H, 2×CH_2), 0.87 (t, ^3J = 7.0 Hz, 3 H, CH_3); ^{13}C NMR (100 MHz, DMSO-d$_6$/$CDCl_3$, ppm): δ = 160.7 (C_q), 147.5 (C_q), 141.9 (C_q), 140.1 (C_q), 133.1 (C_q), 131.0 (C_q), 130.1 (C_q), 129.8 (2×CH), 128.8 (CH), 128.6 (2×CH), 128.0 (2×CH), 127.9 (CH), 126.5 (CH), 126.0 (CH), 125.2 (2×CH), 123.2 (C_q), 122.6 (CH), 122.1 (C_q), 120.7 (CH), 119.6 (C_q), 119.5 (CH), 119.5 (CH), 118.9 (CH), 116.8 (CH), 114.8 (C_q), 111.9 (CH), 110.0 (CH), 42.7 (CH_2), 30.9 (CH_2), 28.5 (CH_2), 26.1 (CH_2), 21.9 (CH_2), 13.6 (CH_3)*; IR (ATR, cm^{-1}): $\tilde{\nu}$ = 3296 (vw), 3138 (vw), 3055 (vw), 2923 (m), 2854 (vw), 1719 (m), 1611 (vw), 1582 (m), 1500 (m), 1464 (m), 1399 (vw), 1360 (m), 1229 (m), 1157 (m), 1025 (m), 910 (vw), 837 (vw), 811 (m), 749 (m); MS (ESI): *m/z* (%) = 1144 (11), 810 (14), 681 (16), 573 (43) $[M+2]^+$, 572 (100) $[M+1]^+$, 571 (19) $[M]^+$, 517 (12), 282 (23), 223 (10), 221 (17), 100 (20); HRMS (ESI): *m/z* calcd. for $C_{39}H_{34}N_5^+$: 572.2809 $[M+1]^+$; found: 572.2818. *One CH and one C_q signal is not visible due to a poor solubility and to the occurance of rotamers.

9-Hexyl-6-phenyl-2-(4-(4-(1,2,2-triphenylvinyl)phenyl)-1*H*-1,2,3-triazol-1-yl)-9*H*-indolo[2,3-*k*]phenanthridine (25)

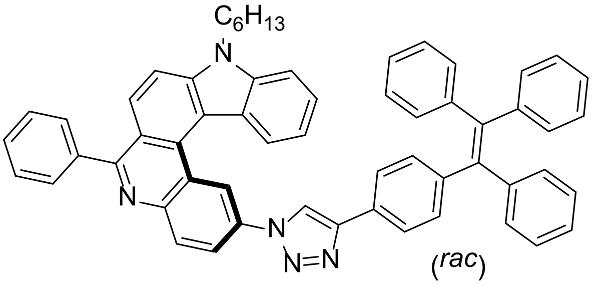

Following a published protocol,[40] $CuSO_4 \cdot 5H_2O$ (14.9 mg, 59 µmol, 0.28 equiv.) and (+)-(+)-sodium-L-ascorbate (20.0 mg, 100 µmol, 0.48 equiv.) were added to a cooled (0 °C) suspension of 2-azido-9-hexyl-6-phenyl-9*H*-indolo[2,3-*k*]phenanthridine (**23**; 99.1 mg, 211 µmol, 1.00 equiv.) and (2-(4-ethynylphenyl)ethene-1,1,2-triyl)tribenzene[70] (**21**; 106 mg, 297 µmol, 1.41 equiv.) in 5 mL THF/H_2O (3:2) and stirred for 64 h, while slowly warming to room temperature. CH_2Cl_2 (5 mL) and H_2O (5 mL) were added. The mixture was extracted with CH_2Cl_2 (3 × 20 mL), dried ($MgSO_4$) and the solvent was removed under reduced pressure. Twofold purification by chromatography (silica gel, hexane/EtOAc 1:0 → 50:1 → 10:1 → 4:1 and hexane/EtOAc 1:0 → 300:1 → 100:1 → 10:1) yielded **25** (40.7 mg, 49 µmol, 23%) as yellow crystalline solid.

R_f = 0.58 (hexane/EtOAc 4:1); ^{1}H NMR (400 MHz, DMSO-d$_6$, ppm): δ = 9.85–9.79 (m, 1 H, H_{ar}), 9.36 (s, 1 H, H_{ar}), 8.84 (d, ^3J = 8.3 Hz, 1 H, H_{ar}), 8.41–8.29 (m, 2 H, 2×H_{ar}), 8.14 (d, ^3J = 9.1 Hz, 1 H, H_{ar}), 8.04 (d, ^3J = 9.0 Hz. 1 H, H_{ar}), 7.89 (d, ^3J = 8.3 Hz, 1 H, H_{ar}), 7.74 (d, ^3J = 7.8 Hz, 4 H, 4×H_{ar}), 7.66–7.54 (m, 4 H, 4×H_{ar}), 7.34 (t, ^3J = 7.7 Hz, 1 H, H_{ar}), 7.22–7.07 (m, 11 H, 11×H_{ar}), 7.06–7.06 (m, 4 H, 4×H_{ar}), 7.01–6.97 (m, 2 H, 2×H_{ar}), 4.63 (t, ^3J = 7.0 Hz, 2 H, CH_2), 1.89–1.79 (m, 2 H, CH_2), 1.42–1.32 (m, 2 H, CH_2), 1.32–1.18 (m, 4 H, 2×CH_2), 0.81 (t, ^3J = 7.0 Hz, 3 H, CH_3); ^{13}C NMR (100 MHz, CD_2Cl_2, ppm): δ = 162.6 (C_q), 148.6 (C_q), 145.0 (C_q), 144.6 (C_q), 144.3 (C_q), 144.2 (C_q), 144.2 (C_q), 142.6 (C_q), 142.0 (C_q), 141.2 (C_q), 141.1 (C_q), 133.8 (C_q), 132.4 (2×CH), 132.0 (C_q), 131.9 (2×CH), 131.8 (2×CH), 131.8 (2×CH), 131.7 (CH), 130.6 (2×CH), 129.2 (CH), 129.0 (C_q), 128.8 (2×CH), 128.3 (2×CH), 128.2 (2×CH), 127.4 (CH), 127.2 (CH), 127.1 (CH), 127.0 (CH), 126.7 (CH), 125.6 (2×CH), 124.6 (C_q), 123.5 (CH and C_q), 121.3 (C_q or CH), 121.2 (CH or C_q), 120.0 (CH), 118.1 (CH), 118.1 (CH), 116.0 (C_q), 111.8 (CH), 110.6 (CH), 44.0 (CH_2), 32.1 (CH_2), 29.6 (CH_2), 27.4 (CH_2), 23.1 (CH_2), 14.3 (CH_3)*; IR (ATR, cm^{-1}): $\tilde{\nu}$ = 3390 (vw), 3052 (vw), 2925 (vw), 2854 (vw), 2253 (vw), 1769 (vw), 1583 (vw), 1491 (w), 1443 (w), 1361 (w), 1157 (w), 1026 (w), 1005 (w), 817 (w), 751 (w); MS (ESI): m/z (%) = 826 (11) [M+1]$^+$, 514 (28) [M+1–C_4H_9–$C_{20}H_5$]$^+$, 318 (18), 274 (100); HRMS (ESI): m/z calcd. for $C_{59}H_{48}N_5^+$: 826.3904; [M+1]$^+$; found: 826.3920. *One C_q signal is covered.

4. Optical Properties

4.1 Calibration Spectra

Calibration spectra for the absorption of selected exemplary compounds (0–100 µM in THF) were measured and Pearson *R* values were calculated for linear regression of 0–20 µM solutions to demonstrate linearity for small concentrations (Beer-Lambert law).

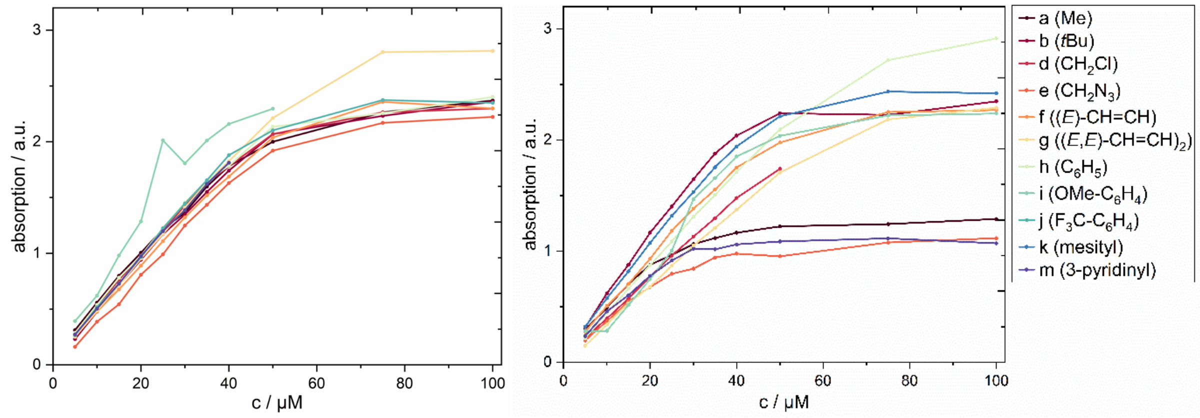

Figure S1: UV/Vis calibration spectra of selected indolo[2,3-*k*]phenanthridines **3** (left) and indolo[3,2-*a*]phenanthridines **9** (right), 0–100 µM in THF.

Table S2: Pearson *R* values for linear regression of 0–20 µM THF solutions of indolo[2,3-*k*]phenanthridines **3** and [3,2-*a*]phenanthridines **9**.

**Compound**	**Indolo[2,3-*k*]phenanthridines 3**<br>**Pearson *R* value**	**Indolo[3,2-*a*]phenanthridines 9**<br>**Pearson *R* value**
**a**, Me	0.9986	0.9978
**b**, *t*Bu	1.000	0.9997
**d**, CH_2Cl	0.9994	0.9996
**e**, CH_2N_3	0.9999	0.9990
**f**, (*E*)-MeCH=CH	0.9994	0.9992
**g**, (*E*,*E*)-Me$(CH=CH)_2$	0.9999	0.9995
**h**, C_4H_5	0.9990	1.000
**i**, MeO-C_6H_4	0.9991	0.9908
**j**, F_3C-C_6H_4	0.9996	0.9992
**k**, mesityl	–	0.9980
**m**, 3-pyridinyl	0.9997	0.9965

4.2 UV/Vis Titration Spectra

Titration with TFA

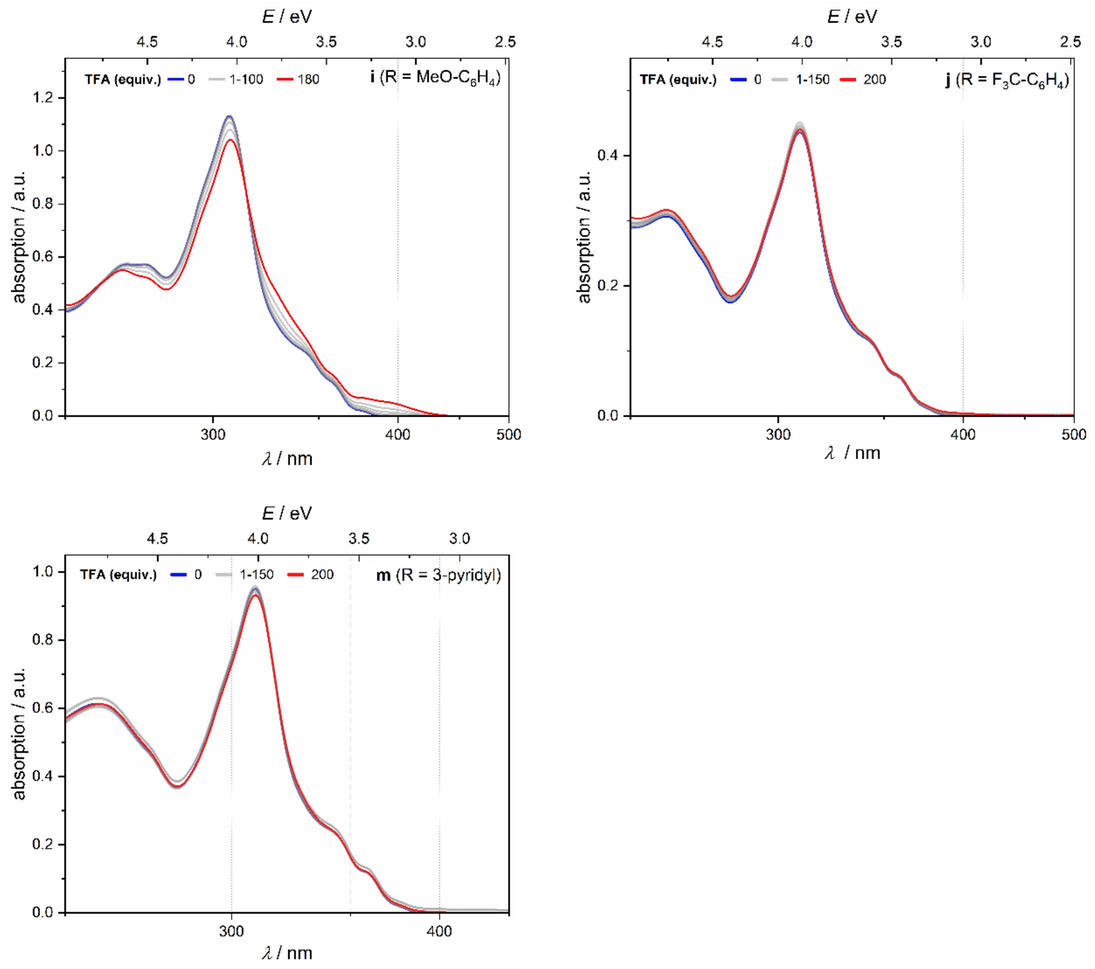

Figure S2: Qualitative UV/Vis titration spectra of indolo[2,3-*k*]phenanthridines **3i**, **3j**, and **3m** (20 µM in THF) with TFA.

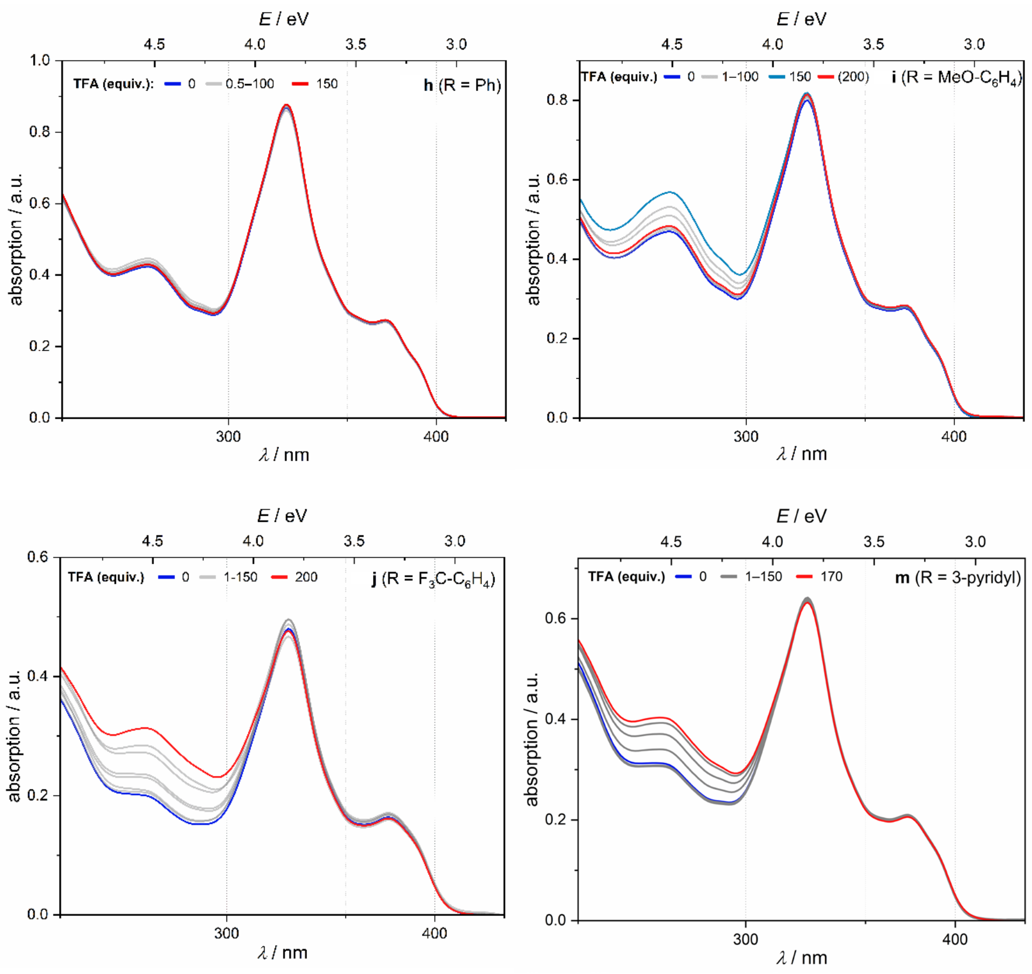

Figure S3: Qualitative UV/Vis titration spectra of indolo[3,2-*a*]phenanthridines **9h–j** and **9m** (20 µM in THF) with TFA.

Comparison between titrations with TFA and TfOH

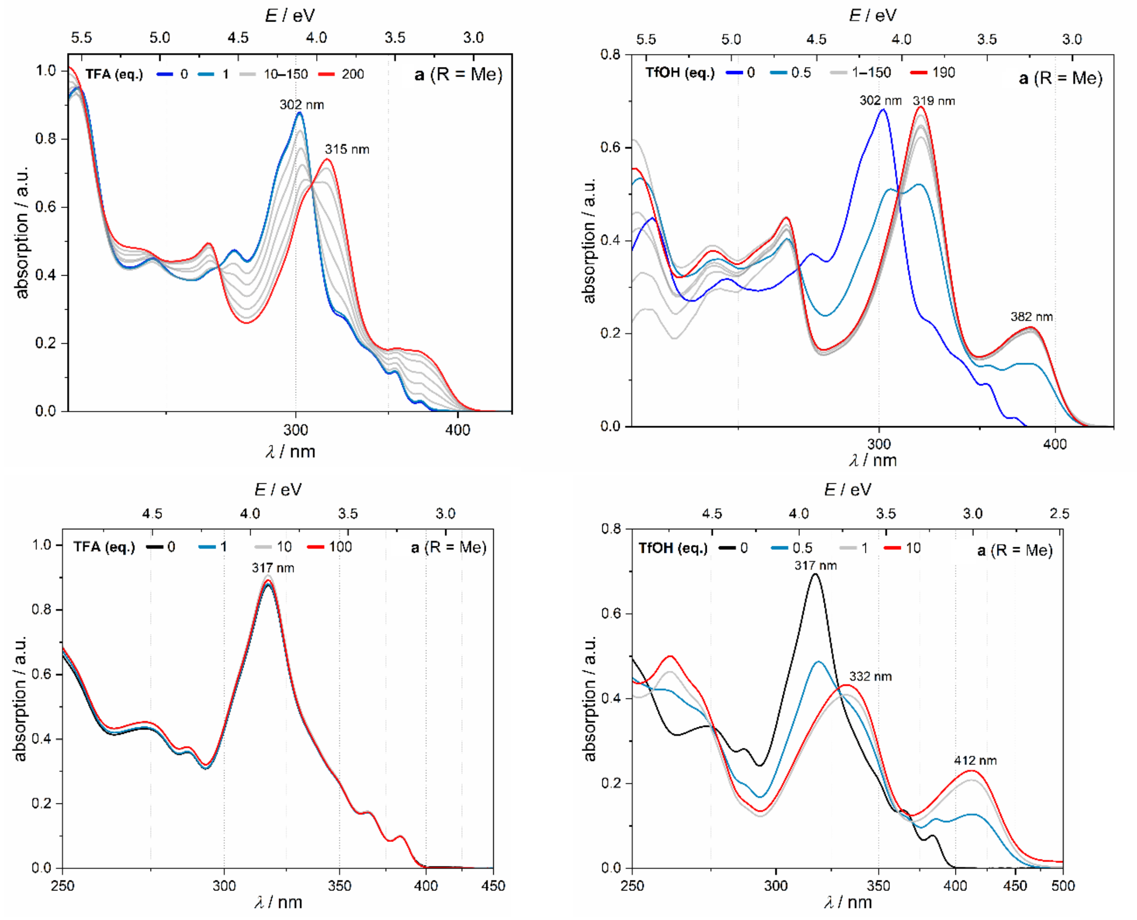

Figure S4: Qualitative UV/Vis titration spectra of indolo[2,3-*k*]phenanthridine **3a** and indolo[3,2-*a*]phenanthridine **9a** (bottom) (20 µM in THF) with TFA (left) and TfOH (right).

Titration with TfOH

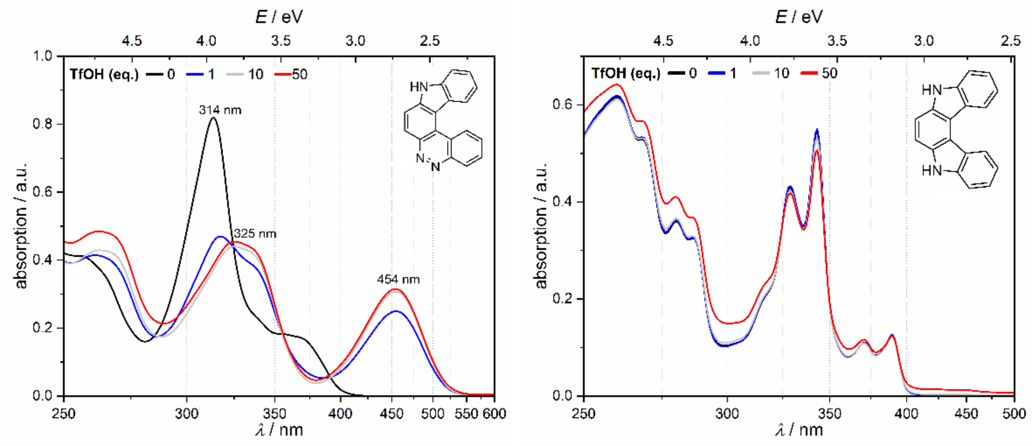

Figure S5: Qualitative UV/Vis titration spectra of CnCz (**4**) and ICz (**5**) (20 µM in THF) with TfOH.

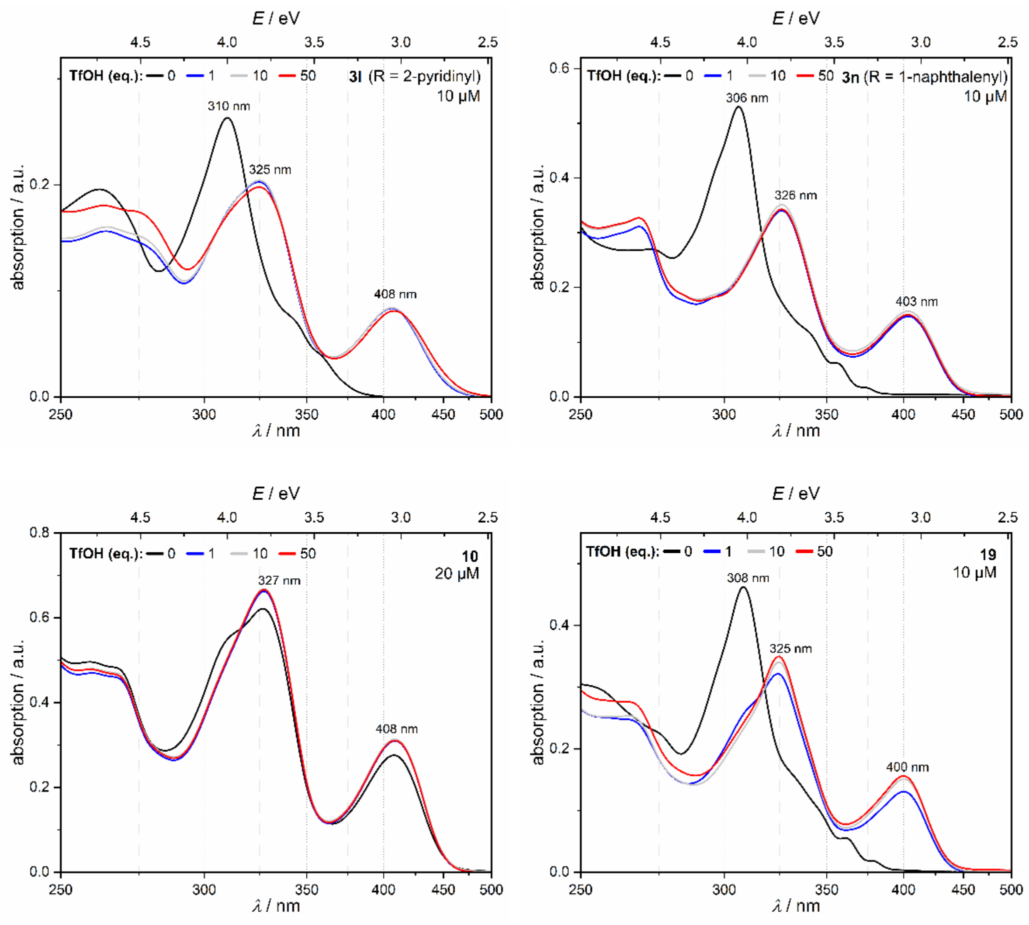

Figure S6: Qualitative UV/Vis titration spectra of indolo[2,3-*k*]phenanthridines **3l**, **3n**, **10** and **19** (10 µM or 20 µM in THF) with TfOH.

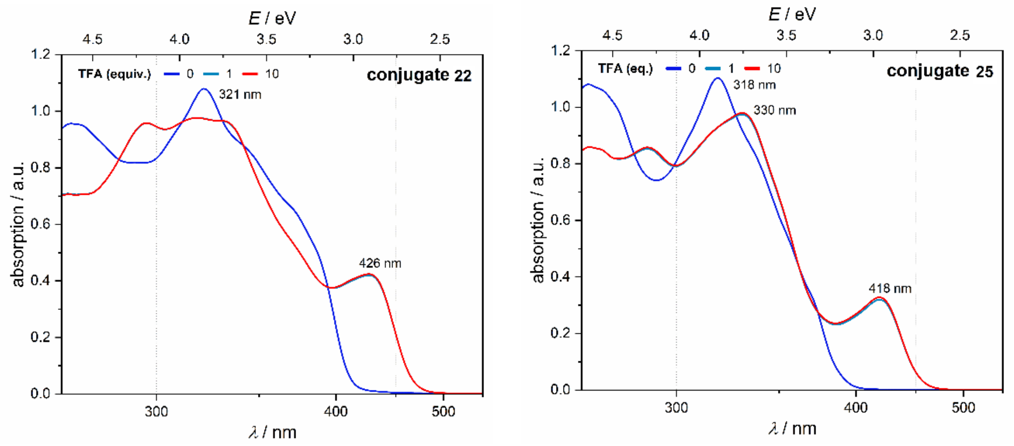

Figure S7: Qualitative UV/Vis titration spectra of helicene-TPE conjugates **22** and **25** (20 µM in THF) with TfOH.

4.3 UV/Vis Absorbance and Fluorescence Spectra

4.3.1 Indolocarbazole (5) and Cinnolinocarbazole (4)

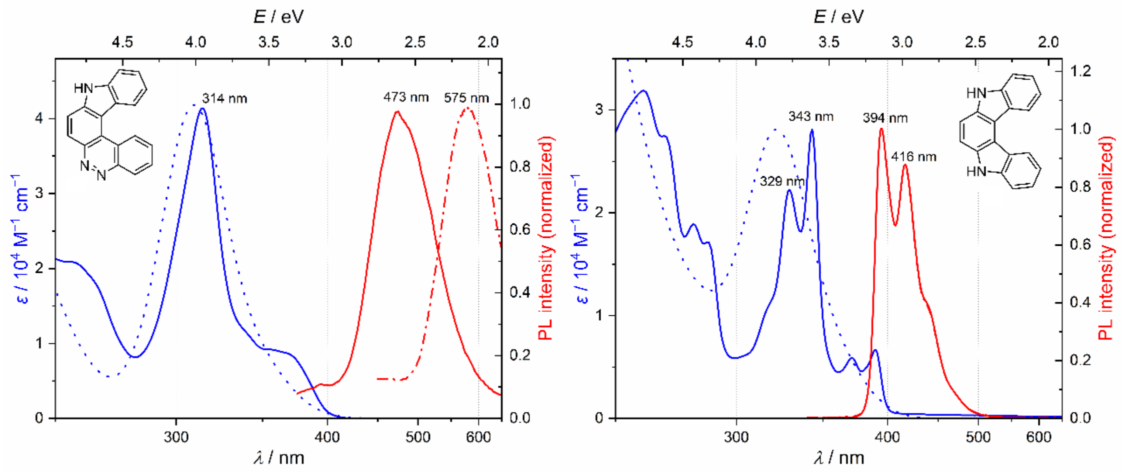

Figure S8: Optical properties of CnCz (**4**) and ICz (**5**): solid blue, measured absorption (THF); dotted blue, calculated absorption (CH_2Cl_2); solid red, normalized emission (THF, λ_{ex} = 330 nm); dash-dotted red, normalized emission after addition of 50 equiv. TfOH (THF, λ_{ex} = 330 nm).

4.3.2 Indolo[2,3-*k*]phenanthridines

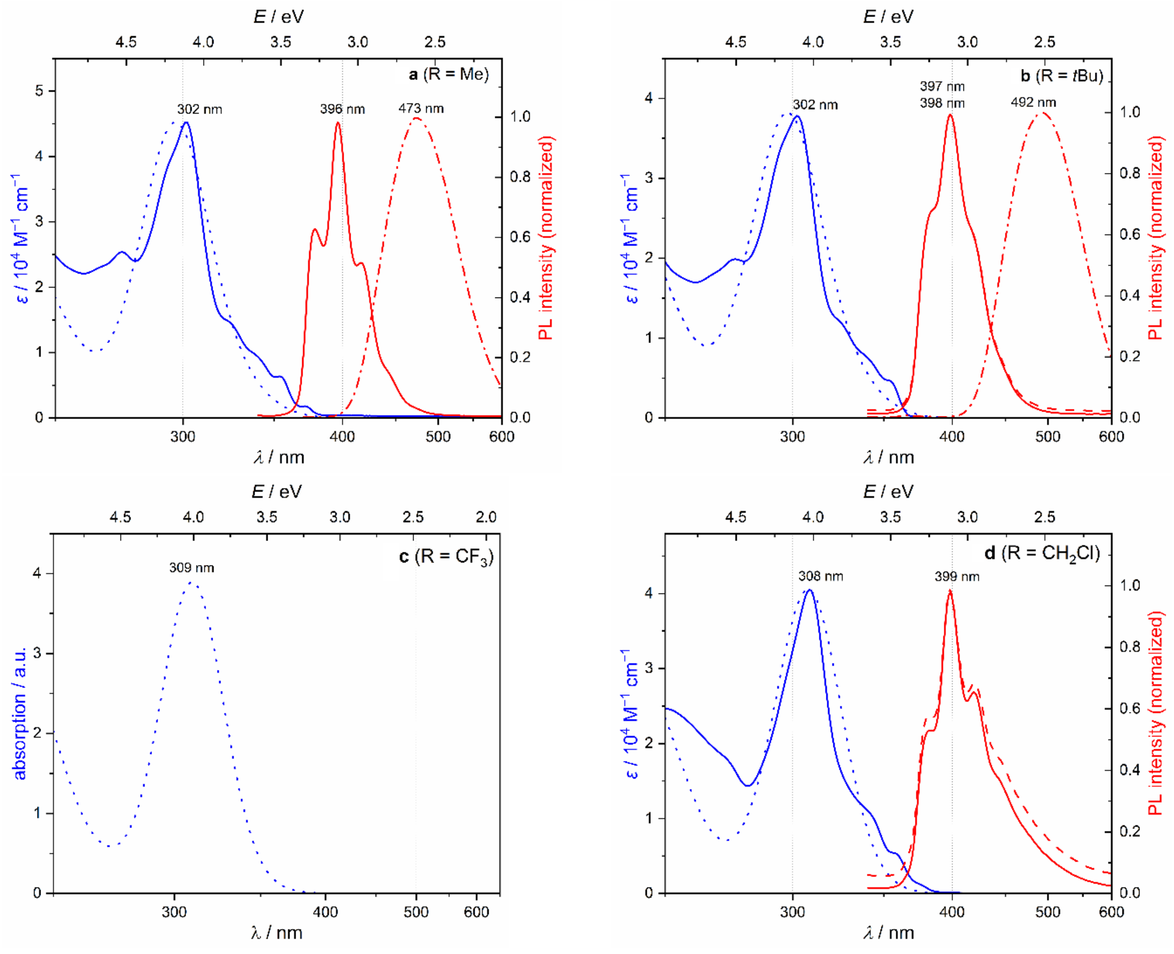

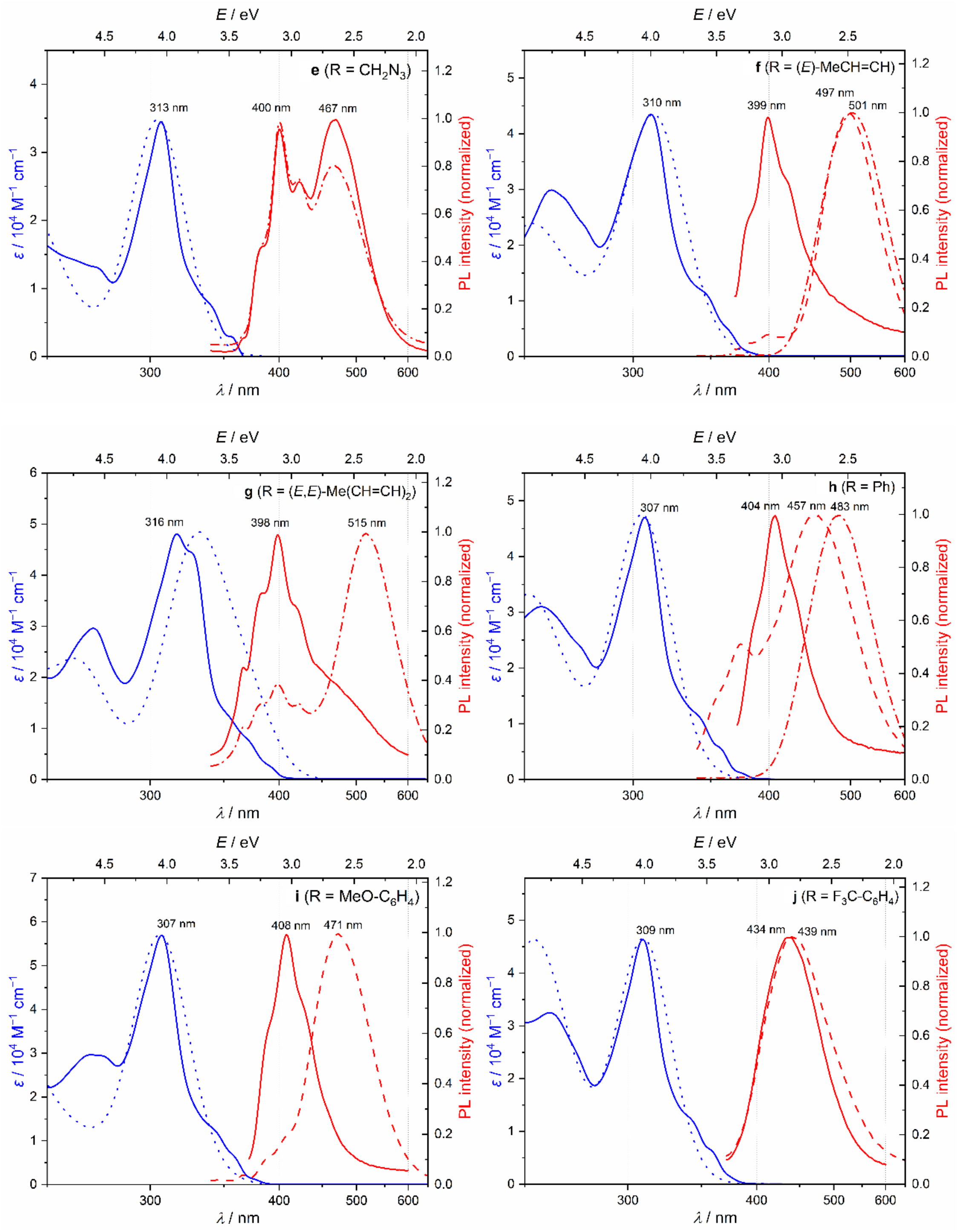
E / eV
λ / nm
ε / 10^4 M^-1 cm^-1
PL intensity (normalized)
e (R = CH2N3)
313 nm
400 nm
467 nm
f (R = (E)-MeCH=CH)
310 nm
399 nm
497 nm
501 nm
g (R = (E,E)-Me(CH=CH)2)
316 nm
398 nm
515 nm
h (R = Ph)
307 nm
404 nm
457 nm
483 nm
i (R = MeO-C6H4)
307 nm
408 nm
471 nm
j (R = F3C-C6H4)
309 nm
434 nm
439 nm

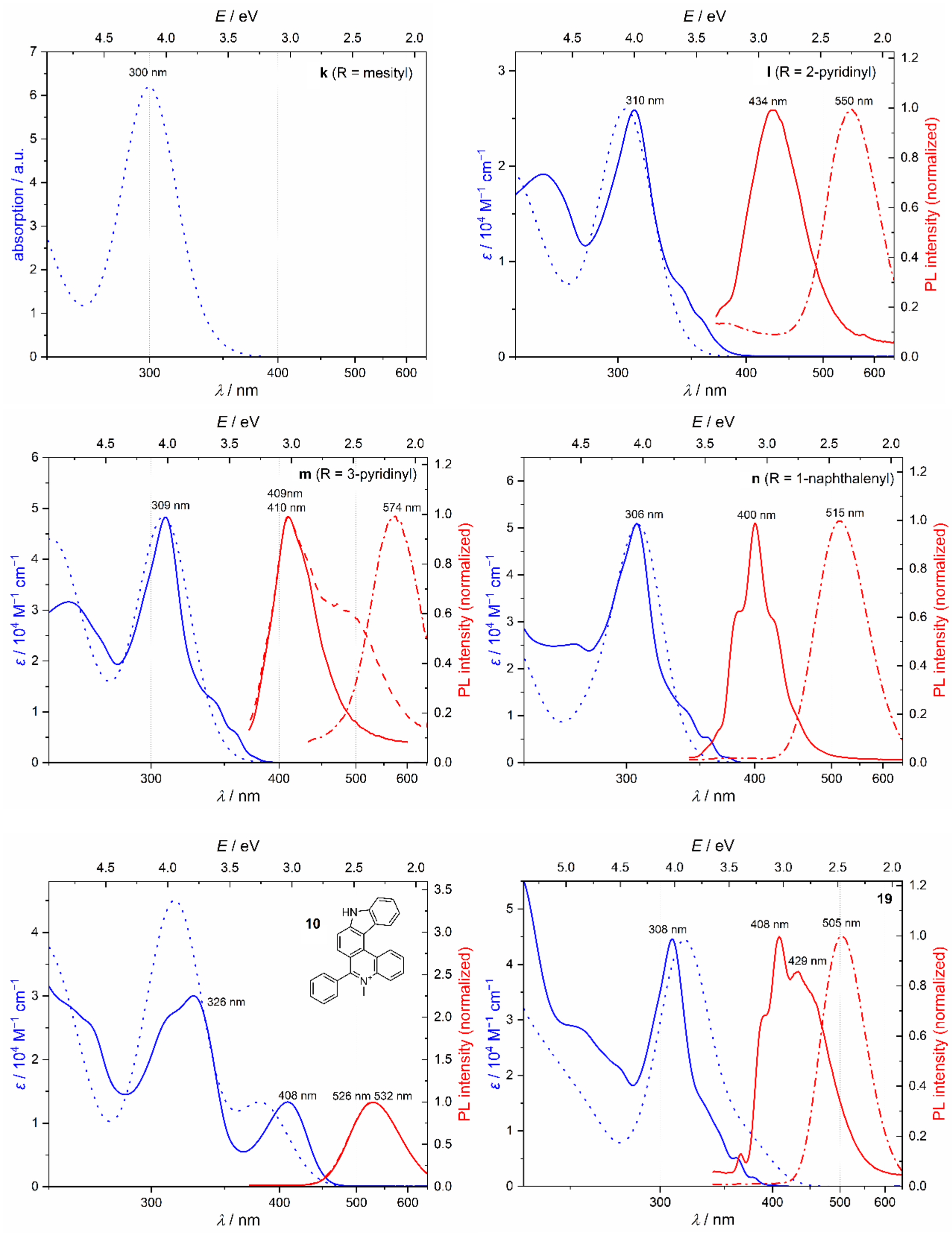

Figure S9: Optical properties of indolo[2,3-*k*]phenanthridines **3a–n**, **10**, and **19**: solid blue, measured absorption (THF); dotted blue, calculated absorption (CH_2Cl_2); solid red, normalized emission (THF, λ_{ex} = 330 nm); dashed red, normalized emission after addition of 50 equiv. TFA (THF, λ_{ex} = 330 nm); dash-dotted red, normalized emission after addition of 50 equiv. TfOH (THF, λ_{ex} = 330 nm).

4.3.3 Indolo[3,2-*a*]phenanthridines

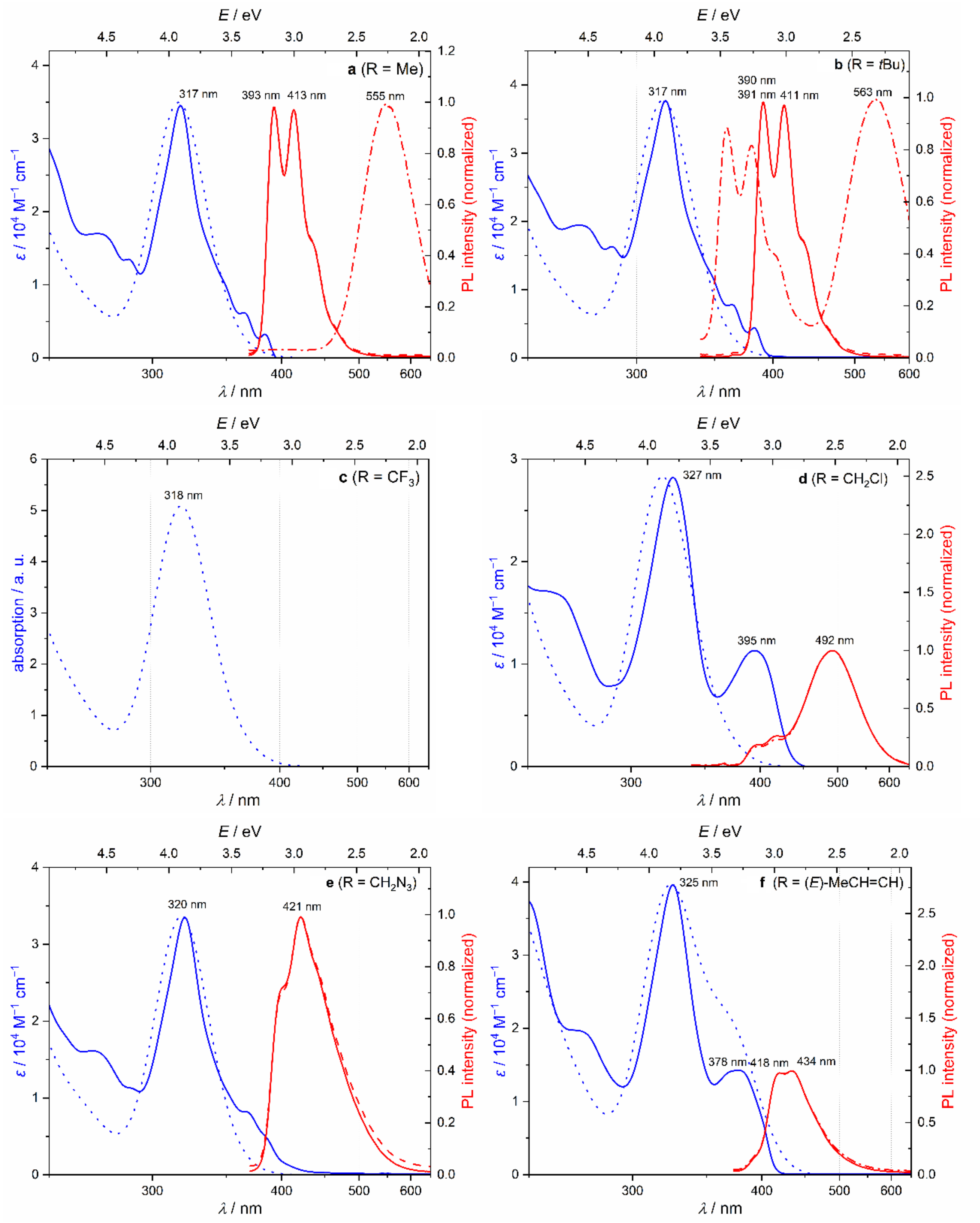

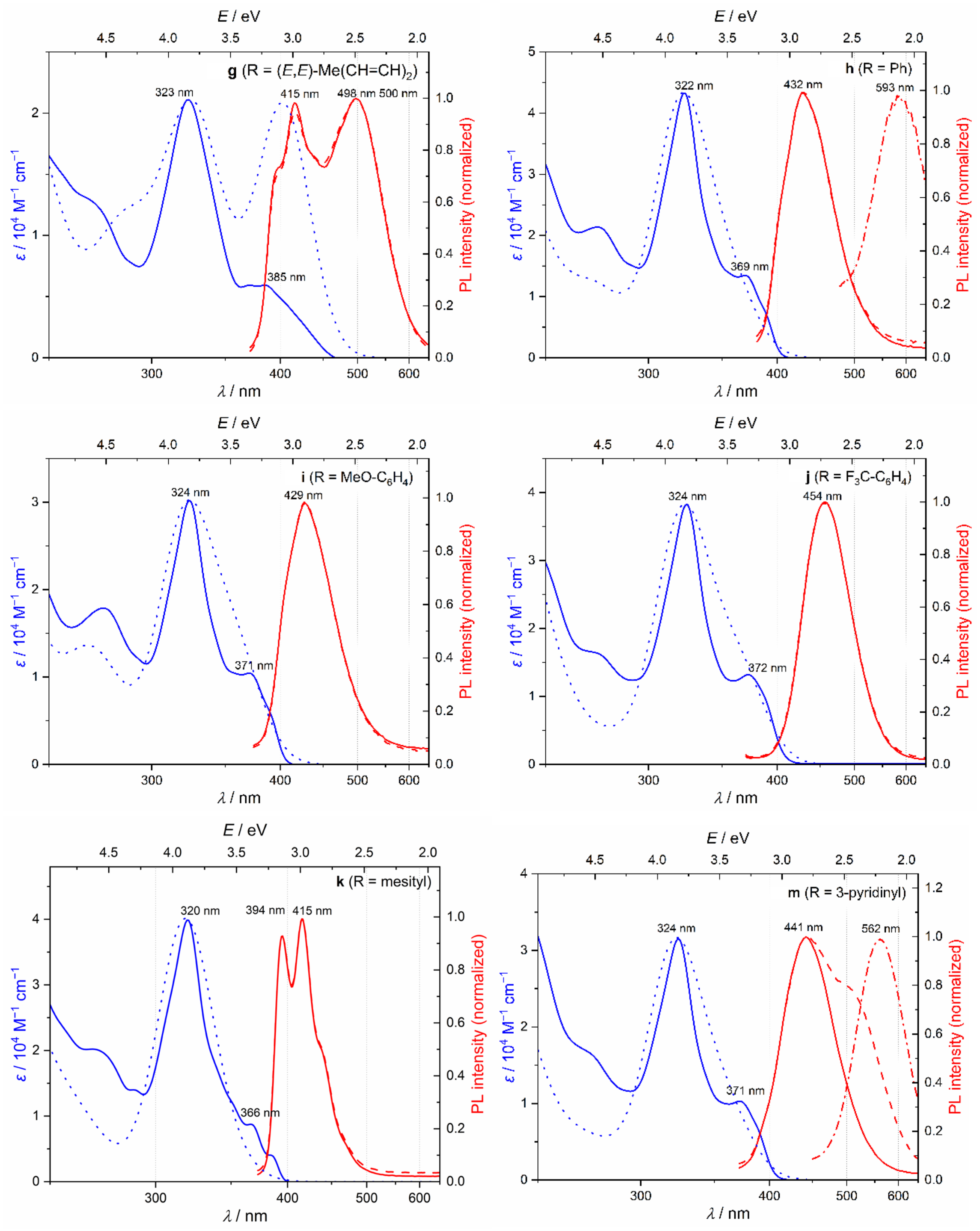

Figure S10: Optical properties of indolo[3,2-*a*]phenanthridines **9**: solid blue, measured absorption (THF); dotted blue, calculated absorption (CH_2Cl_2); solid red, normalized emission (THF, λ_{ex} = 330 nm); dashed red, normalized emission after addition of 50 equiv. TFA (THF, λ_{ex} = 330 nm); dash-dotted red, normalized emission after addition of 50 equiv. TfOH (THF, λ_{ex} = 330 nm).

4.3.4 Helicene-TPE conjugates 22 and 25

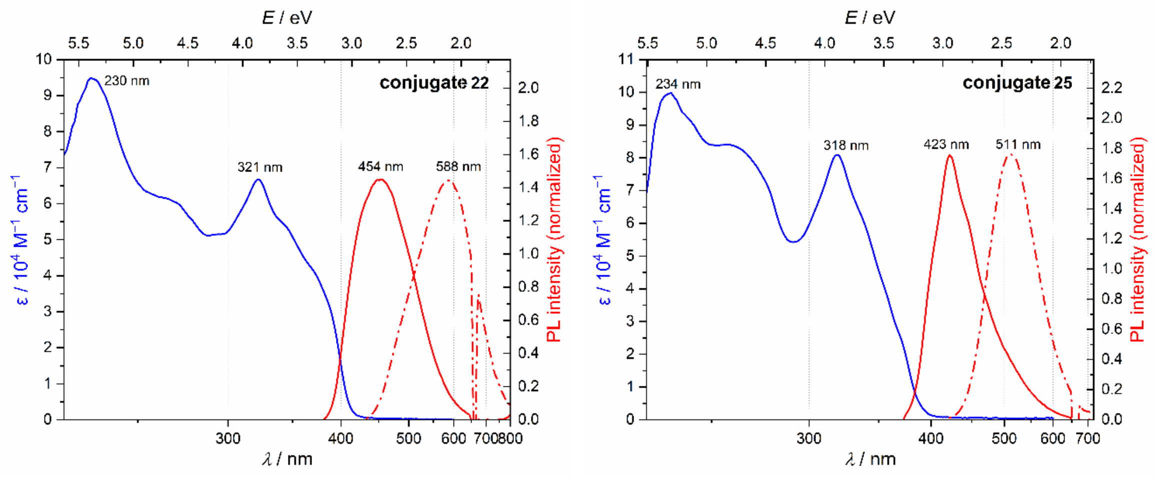

Figure S11: Optical properties of helicene-TPE conjugates **22** and **25**: solid blue, measured absorption (THF); solid red, normalized emission (THF, λ_{ex} = 330 nm); dash-dotted red, normalized emission after addition of 10 equiv. TfOH (THF, λ_{ex} = 330 nm).

4.4 Solvatochromism

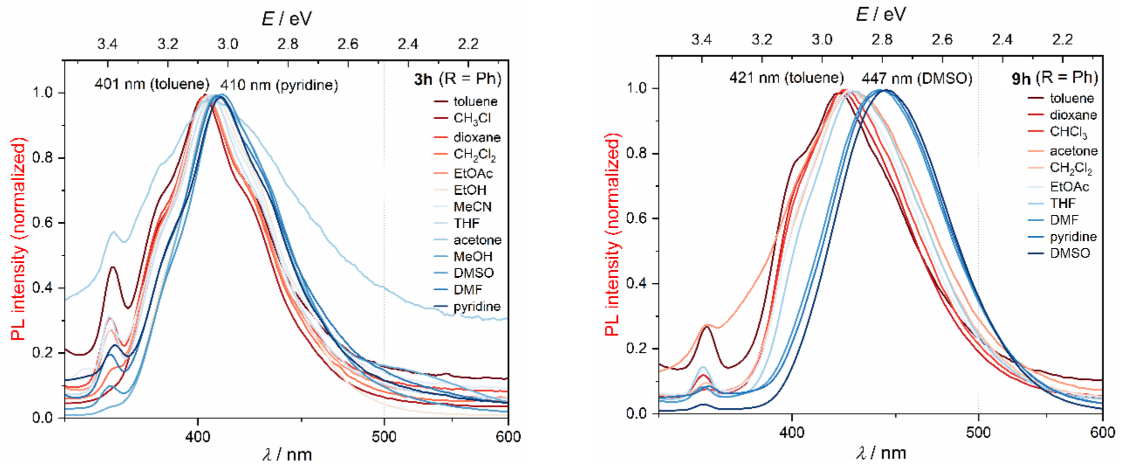

Figure S12: Normalized emission spectra of phenyl-substituted indolo[2,3-*k*]phenanthridine **3h** (left) and indolo[3,2-*a*]phenanthridine **9h** (right) in different solvents (λ_{ex} = 330 nm).

4.5 Emission Behavior in THF/H2O Solutions

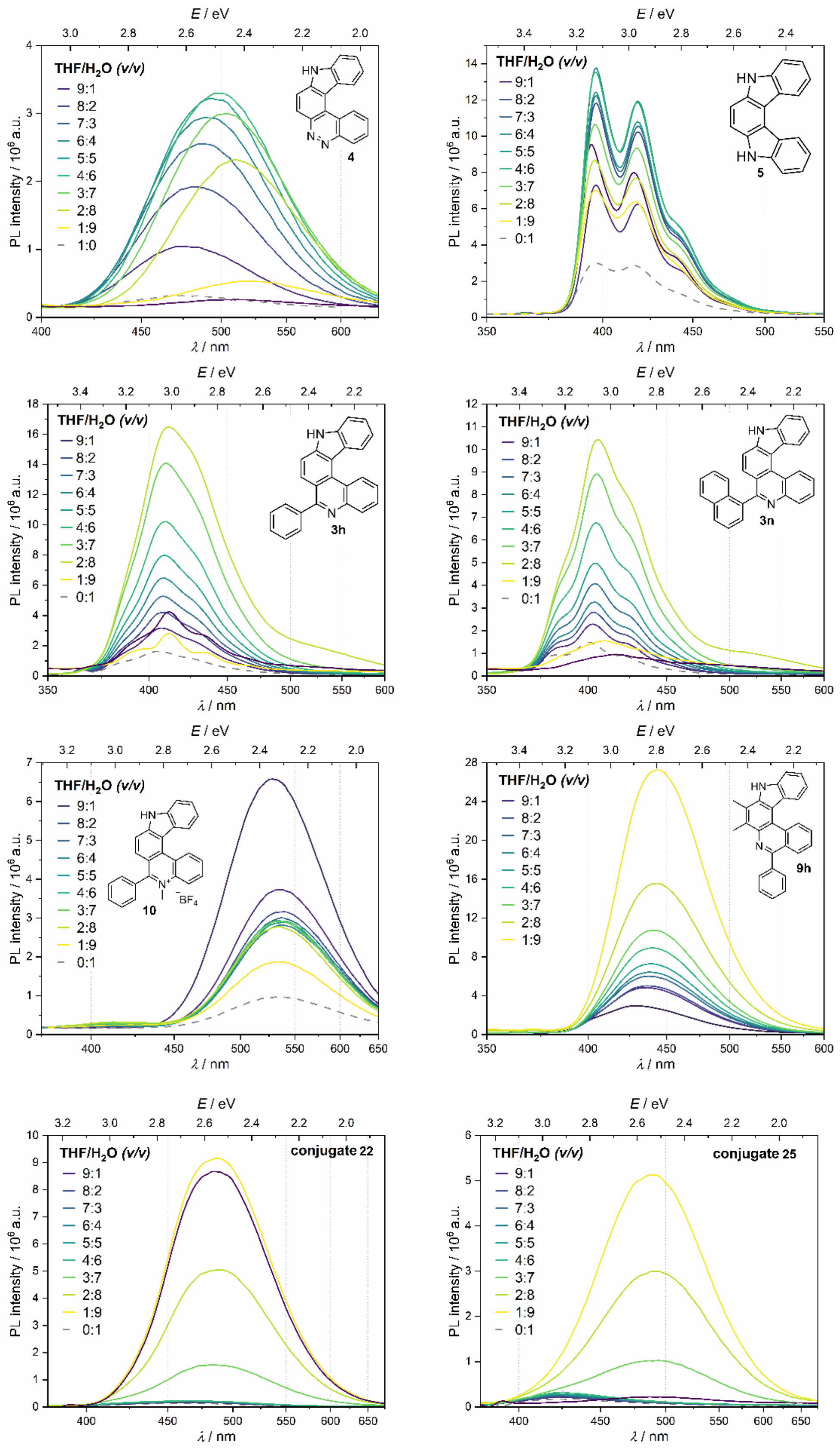

Figure S13: Emission of ICz (**5**), CnCz (**4**), indolo[2,3-*k*]phenanthridines **3h**, **3n,** indolophenanthridinium derivative **10**, indolo[3,2-*a*]phenanthridine **9h** (λ_{ex} = 330 nm) and TPE-conjugates **22** and **25** in THF/H_2O (λ_{ex} = 345 nm).

5. XRD – Structural Data

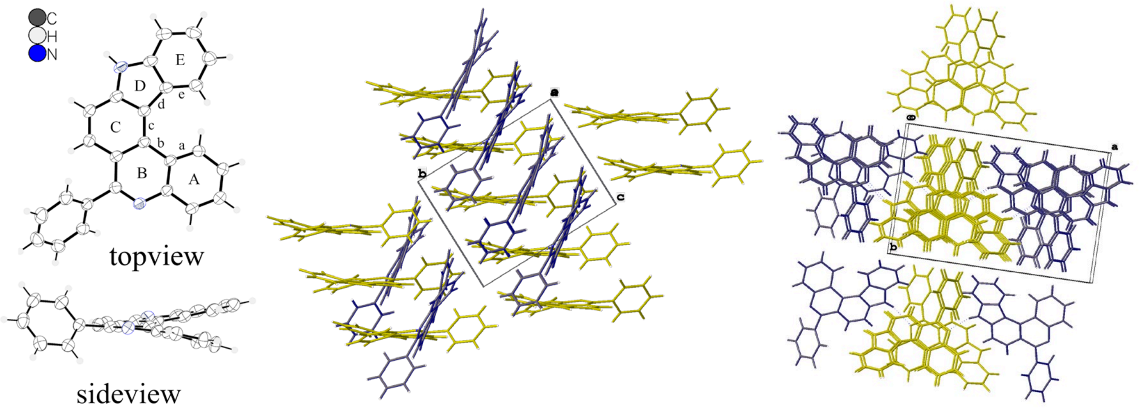

Figure S14: XRD structures of phenyl-substituted indolo[2,3-*k*]phenanthridine **3h**.

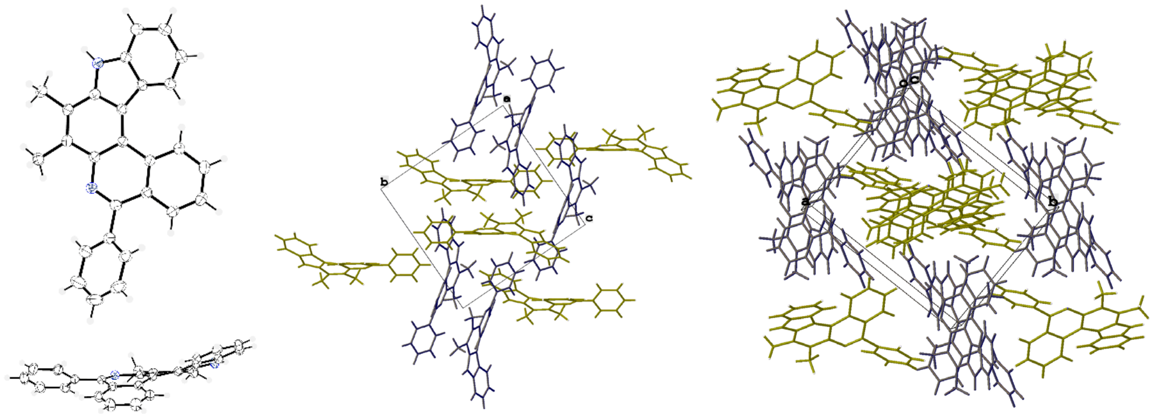

Figure S15: XRD structures of phenyl-substituted indolo[3,2-*a*]phenanthridine **9h**.

Table S3: Structural parameters of phenyl-substituted IPs **3h** and **9h**.

Compound	Interplanar angle ϑ_{AE} [°]	ϑ'_{AR} [°]	Torsion angle φ_{abc} [°]	φ'_{cde} [°]	φ''_{ae} [°]	Sum of torsion angles [°]	*d* [Å]
[2,3-*k*]-IP **3h**	25.6	56.1	16.4	11.6	34.0	39.2	5.55
[3,2-*a*]-IP **9h**	38.0	41.1	16.5	24.5	41.8	42.7	5.58

6. Computational Studies

6.1 Computational Methods

All calculations were performed using the Gaussian 16 software package[71] at the PBE0[72,73]/def2-TZVP[74,75] level with Grimme's dispersion correction at the D3 level[76] and with Becke-Johnson damping (GD3BJ).[77] A modelled solvent field of methylene chloride was simulated using the polarizable conductor calculation model (cpcm-scrf method).[78–80] Frequency calculations[81–83] were carried out at the same level of theory as that used for optimizations; all optimized structures turned out to be minima (no imaginary frequencies) or first-order saddle points (transition states, one imaginary frequency). UV/Vis and ECD spectra were calculated with time-dependent DFT calculations [td=(nstates=50)][84–86] again at that level. S_1 and T_1 states were calculated using TD calculations with the Tamm-Dancoff approximation.

Racemization barriers were determined from zero point-corrected energies of the enantiomers and transition states in the ground state. Intrinsic reaction coordinates were calculated for 200 points in both directions using a step size of 30 and local quadratic approximation[87,88] for the predictor step [irc=(calcfc,maxpoints=200,stepsize=30,lqa)].

Molecules, molecular orbitals, and the ESP spectrum were visualized with GaussView, versions 3.0.9 and 6.1.1,[89] and Mercury (CCDC), version 4.0.[90] Calculated UV/Vis and ECD spectra were processed using GaussSum,[91] where a FWHM of 4000 cm^{-1} (for UV/Vis spectra) and σ values of 0.4 eV (for ECD spectra) turned out to give satisfactory results.

6.2 Calculated Structures

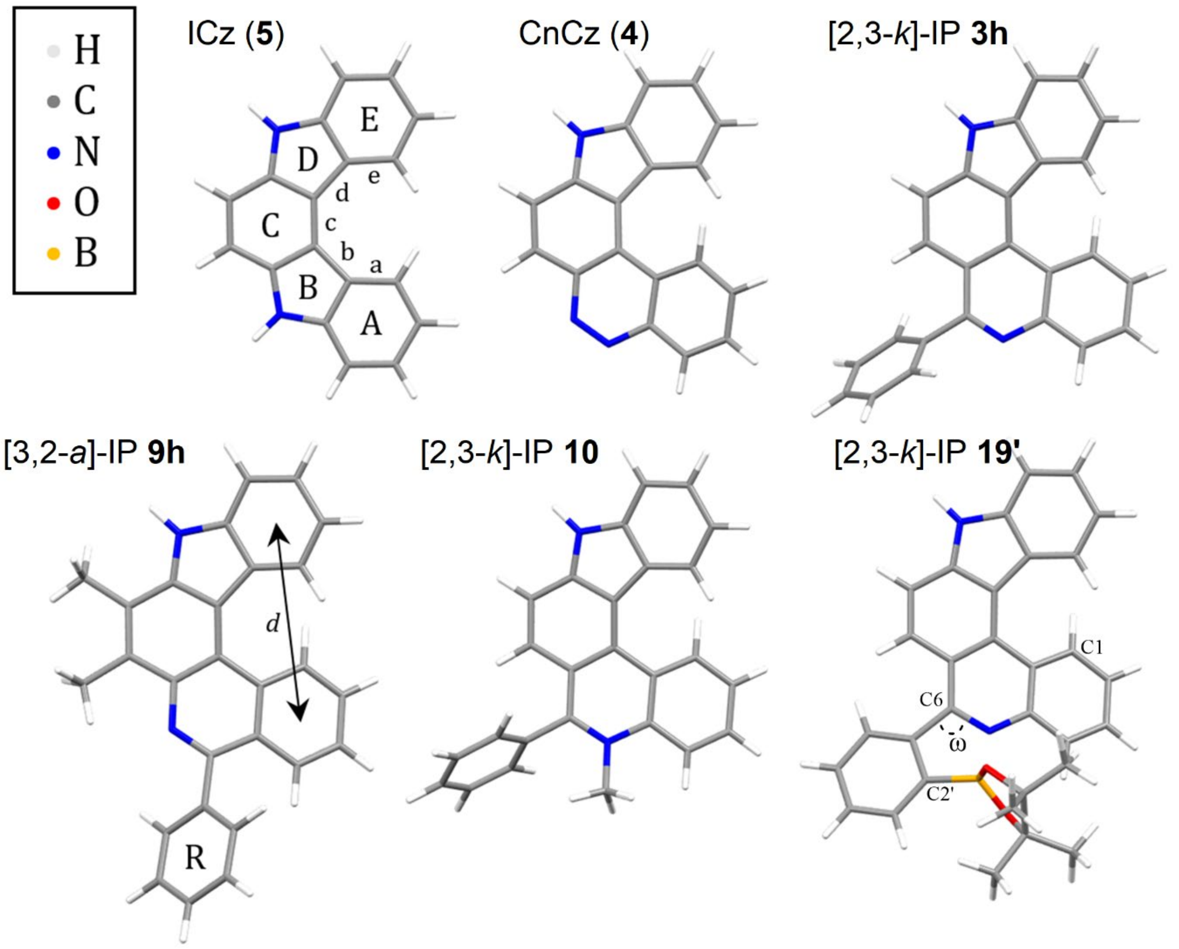

Figure S16: Calculated structures of selected aza[5]helicenes.

Table S4: Calculated structural properties of selected aza[5]helicenes.

Compound	Interplanar angle		Torsion angle			Sum of	*d*
	ϑ_{AE}	ϑ'_{AR}	φ_{abc}	φ'_{cde}	φ''_{ae}	torsion angles	
	[°]	[°]	[°]	[°]	[°]	[°]	[Å]
ICz (**5**)	2.8	–	1.2	1.2	3.2	3.9	6.01
CnCz (**4**)	29.1	–	14.4	7.5	34.4	38.3	5.61
**3a**	32.0	–	17.1	7.4	38.5	41.9	5.52
**3b**	34.4	–	17.9	7.2	41.2	44.4	5.46
**3c**	32.4	–	17.1	7.6	39.0	42.4	5.51
**3d**	32.1	–	17.3	7.4	38.6	42.0	5.52
**3e**	32.1	–	16.6	7.6	38.6	41.9	5.52

**3f**	33.2	–	16.7	7.4	39.8	42.9	5.51
**3g**	33.2	–	16.9	7.3	39.9	43.1	5.51
**3h**	31.7	60.9	19.9	7.2	37.9	41.7	5.50
**3i**	31.6	39.7	19.3	7.1	37.8	41.6	5.49
**3j**	31.7	41.2	18.9	7.2	37.9	41.7	5.50
**3k**	31.7	82.6	16.9	7.3	38.0	41.4	5.53
**3l**	31.6	40.8	18.9	7.2	37.9	41.7	5.50
**3m**	31.6	79.1	18.1	7.3	38.0	41.6	5.51
**3n**	32.2	65.0	16.2	7.5	38.7	41.9	5.53
**10**	34.6	23.1	18.5	7.7	41.3	44.5	5.49
**19'**	34.8	37.5	22.8	6.6	40.6	44.6	5.46
**9a**	33.2	–	16.7	6.3	42.2	39.5	5.52
**9b**	34.4	–	17.4	5.7	40.9	43.0	5.51
**9c**	33.4	–	17.1	6.5	39.9	42.6	5.51
**9d**	32.9	–	16.7	6.6	39.4	42.2	5.52
**9e**	32.8	–	16.9	6.3	39.3	42.0	5.52
**9f**	34.3	–	17.4	6.1	40.6	43.1	5.52
**9g**	34.3	–	17.5	6.0	40.6	43.1	5.52
**9h**	35.4	57.8	18.0	6.0	41.3	43.8	5.53
**9i**	44.3	56.1	17.9	6.0	41.4	44.0	5.53
**9j**	35.4	57.0	18.0	6.0	41.2	43.8	5.52
**9k**	33.3	89.4	16.9	6.2	39.6	42.4	5.52
**9m**	35.2	44.5	17.8	6.1	41.1	43.7	5.53

Table S5: Molecular orbitals and their energies of the different types of helicenes (for [2,3-*k*] and [3,2-*a*]: parent framework) (isocontour value of 0.03 a.u.).

	**HOMO−1**	**HOMO**	**LUMO**	**LUMO+1**	**Δ LUMO−HOMO**
ICz (**5**)	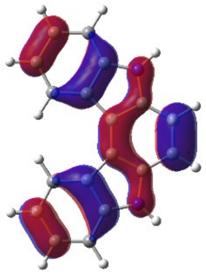<br>−5.96 eV	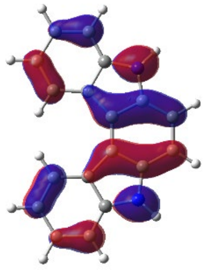<br>−5.57 eV	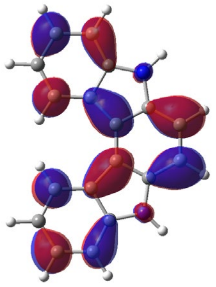<br>−1.34 eV	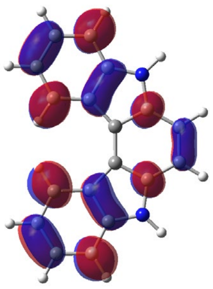<br>−0.35 eV	4.23 eV
CnCz (**4**)	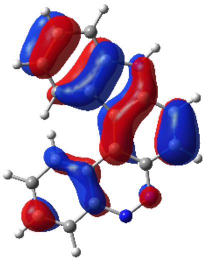<br>−6.26 eV	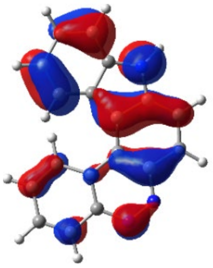<br>−4.99 eV	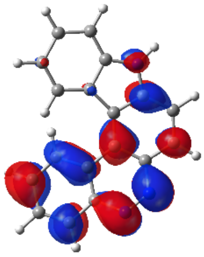<br>−2.01 eV	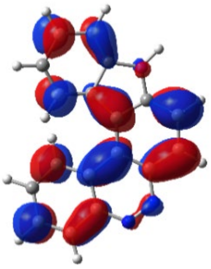<br>−1.47 eV	2.98 eV
[2,3-*k*] (**3**)	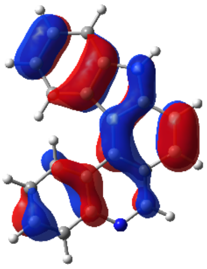<br>−6.30 eV	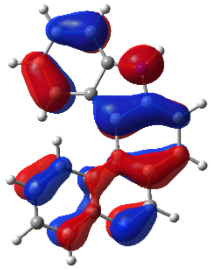<br>−6.21 eV	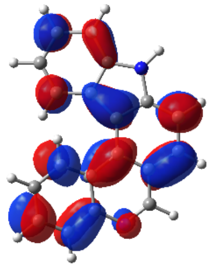<br>−1.61 eV	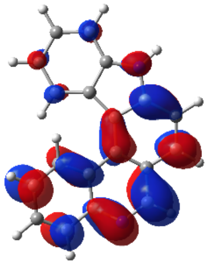<br>−1.43 eV	4.60 eV
[3,2-*a*] (**9**)	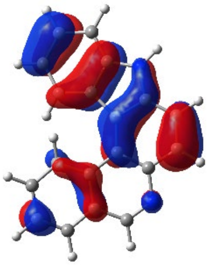<br>−6.28 eV	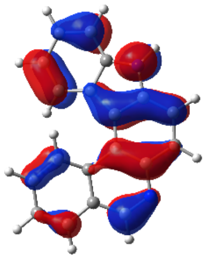<br>−6.04 eV	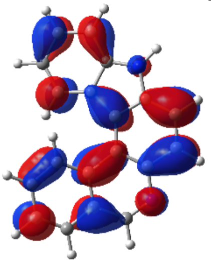<br>−1.60 eV	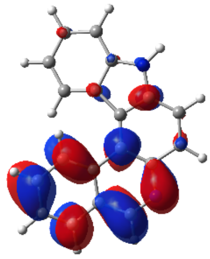<br>−1.51 eV	4.44 eV

Table S6: Molecular orbitals and their energies of **10** and **19’** (isovalue 0.03 a.u.).

	**HOMO-1**	**HOMO**	**LUMO**	**LUMO+1**	**Δ LUMO-HOMO**
**10**	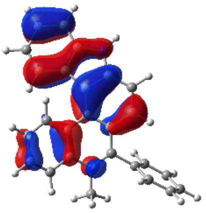 −6.96 eV	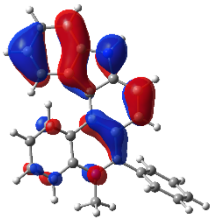 −6.93 eV	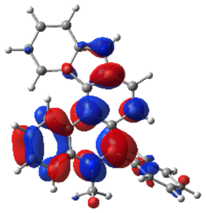 −2.97 eV	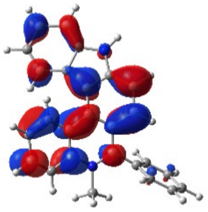 −2.31 eV	3.96 eV
**19’**	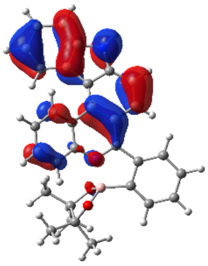 −6.43 eV	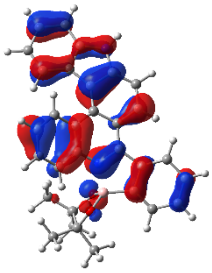 −6.34 eV	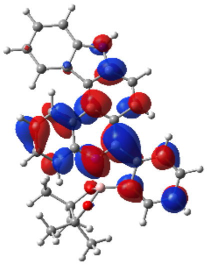 −2.28 eV	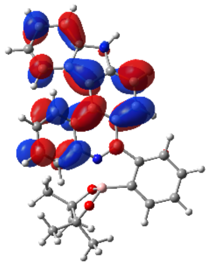 −1.76 eV	4.06 eV

Table S7: Calculated HOMO/LUMO gaps of indolo[2,3-*k*]phenanthridines **3** and indolo[3,2-*a*]phenanthridines **9**.

R	**Compound**	**HOMO/LUMO gap**	**Compound**	**HOMO/LUMO gap**
Me	**3a**	4.60 eV	**9a**	4.42 eV
Me, protonated	**3a**·H^+	4.07 eV	**9a**·H^+	3.73 eV
*t*Bu	**3b**	4.61 eV	**9b**	4.44 eV
CF_3	**3c**	4.51 eV	**9c**	4.23 eV
CH_2Cl	**3d**	4.46 eV	**9d**	4.22 eV
CH_2N_3	**3e**	4.55 eV	**9e**	4.41 eV
(*E*)-MeCH=CH	**3f**	4.40 eV	**9f**	4.03 eV
(*E*,*E*)-Me(CH=CH)$_2$	**3g**	4.03 eV	**9g**	3.96 eV
Ph	**3h**	4.59 eV	**9h**	4.24 eV
4-MeO-C_6H_4	**3i**	4.52 eV	**9i**	4.21 eV
4-F_3C-C_6H_4	**3j**	4.50 eV	**9j**	4.11 eV
mesityl	**k**	4.54 eV	**9k**	4.43 eV
2-pyridinyl	**3l**	4.67 eV	–	–
3-pyridinyl	**3m**	4.55 eV	**9m**	4.19 eV
1-naphthalenyl	**3n**	4.65 eV	–	–

***Table S8**:* Photophysical data of ICz (**5**) and CnCz (**4**) calculated at the TD-DFT/PBE0/def2-TZVP level with implicit solvent field (CPCM, CH_2Cl_2); only selected transitions >230 nm are listed.

**Compound**	**Calcd. absorbance (nm)**	**Oscillator strength *f***	**Assignment [Exp. Abs. (nm)]**	**Transition (S_0–S_1 vertical)**
ICz (**5**)	357	0.0900	343 (max)	HOMO → LUMO (96%)
	321	0.4720	326, 343 (max)	H–1 → LUMO (95%)
	283	0.1469	–	HOMO → L+1 (94%)
CnCz (**4**)	347	0.1197	–	H–1 → L+1 (12%), HOMO-LUMO (78%)
	315	0.1316	314 (max)	H–1 → LUMO (24%), HOMO → L+1 (67%)
	307	0.7244	314 (max)	H–1 → L+1 (81%), HOMO-LUMO (12%)

***Table S9**:* Photophysical data of a number of indolo[2,3-*k*]phenanthridines **3** calculated at the TD-DFT/PBE0/def2-TZVP level with implicit solvent field (CPCM, CH_2Cl_2); only selected transitions >230 nm are listed.

**Compound**	**Calcd. absorbance (nm)**	**Oscillator strength *f***	**Assignment [Exp. Abs. (nm)]**	**Transition (S_0–S_1 vertical)**
**3a**	325	0.0968	–	H–1 → LUMO (75%), HOMO → L+1 (22%)
	297	0.7215	302 (max)	H–1 → LUMO (20%), HOMO → L+1 (73%)
	293	0.2665	302 (max)	H–2 → LUMO (11%), H–1 → L+1 (62%), HOMO → LUMO (20%)
	238	0.1831	245–275 (shoulder)	H–3 → L+1 (57%), HOMO → L+2 (21%)
**3a·H$^+$**	374	0.0525	–	H–1 → LUMO (70%), HOMO → LUMO (22%)
	370	0.1244	–	H–1 → LUMO (19%), HOMO → LUMO (68%)
	322	0.1339	–	H–1 → L+1 (17%), HOMO → L+1 (72%)
	315	0.5641	–	H–1 → L+1 (68%), HOMO → L+1 (15%)
	298	0.1111	–	H–2 → LUMO (90%)

	257	0.2509	–	H–3 → LUMO (22%), H–2 → L+1 (61%)
	250	0.1277	–	H–3 → L+1 (20%), H–1 → L+2 (36%), HOMO → L+2 (24%)
**3b**	336	0.0133	–	H–1 → L+1 (23%), HOMO → LUMO (74%)
	299	0.6627	302 (max)	H–1 → LUMO (20%), HOMO → L+1 (74%)
	294	0.2645	302 (max)	H–2 → LUMO (10%), H–1 → L+1 (64%), HOMO → LUMO (22%)
	244	0.2680	245 (shoulder)	H–1 → L+2 (42%), HOMO → L+3 (19%)
	238	0.2265	–	H–4 → L+1 (28%), H–3 → L+1 (35%),HOMO → L+2 (19%)
**3f**	347	0.0088	–	H–1 → LUMO (30%), HOMO → LUMO (14%), HOMO → L+1 (45%)
	340	0.0640	–	H–1 → L+1 (25%), HOMO → LUMO (52%), HOMO → L+1 (12%)
	314	0.4340	310 (max)	H–1 → L+1 (63%), HOMO → LUMO (30%)
	312	0.5966	310 (max)	H–1 → LUMO (56%), HOMO → L+1 (40%)
	253	0.2952	261 (max)	H–3 → LUMO (17%), H–1 → H+2 (11%), HOMO → L+2 (64%)
	249	0.2004	–	H–1 → L+2 (63%), HOMO → L+3 (10%)
**3h**	330	0.0248		H–1 → LUMO (50%), HOMO → L+1 (40%)
	307	0.4953	307 (max)	H–1 → LUMO (40%), HOMO → L+1 (48%)
	303	0.6165	–	H–1 → L+1 (60%), HOMO → LUMO (30%)
	252	0.3201	–	H–3 → L+1 (27%), H–1 → L+2 (10%), HOMO → L+2 (38%)
**3i**	331	0.0724		H–1 → LUMO (49%), HOMO → L+1 (43%)
	309	0.4098	307 (max)	H–1 → LUMO (39%), HOMO → L+1 (49%)
	305	0.8060	307 (max)	H–1 → L+1 (66%), HOMO → LUMO (28%)
	267	0.1218	274 (max)	H–3 → LUMO (77%)
**3j**	334	0.0197		H–1 → L+1 (32%), HOMO → LUMO (54%)
	311	0.2976		H–1 → LUMO (27%), H–1 → L+1 (24%), HOMO → LUMO (18%), HOMO → L+1 (25%)
	308	0.7114	309 (max)	H–1 → LUMO (24%), H–1 → L+1 (37%), HOMO → LUMO (17%), HOMO → L+1 (18%)

	256	0.5787	–	H–3 → LUMO (56%), H–1 → L+2 (15%)
	250	0.2295	–	H–1 → L+3 (12%), HOMO → L+3 (72%)
**3m**	332	0.0150	–	H–1 → LUMO (18%), H–1 → L+1 (28%), HOMO → LUMO (38%), HOMO → L+1 (13%)
	309	0.3821	309 (max)	H–1 → LUMO (30%), H–1 → L+1 (17%), HOMO → LUMO (17%), HOMO → L+1 (29%)
	306	0.6748	309 (max)	H–1 → LUMO (19%), H–1 → L+1 (42%), HOMO → LUMO (21%), HOMO → L+1 (14%)
	251	0.3699	–	H–3 → LUMO (23%), HOMO → L+3 (42%)
	247	0.3251	–	H–4 → L+1 (10%), H–3 → L+1 (17%), H–1 → L+3 (30%)
**10**	388	0.0623	408 (max)	H–1 → LUMO (61%), HOMO → LUMO (33%)
	380	0.1992	408 (max)	H–1 → LUMO (31%), HOMO → LUMO (60%)
	322	0.0979	326 (max)	H–1 → L+1 (31%), HOMO → L+1 (56%)
	317	0.6098	–	H–1 → L+1 (52%), HOMO → L+1 (33%)
	305	0.1571	–	H–2 → LUMO (85%), H–1 → L+1 (10%)
	296	0.1324	–	H–3 → LUMO (92%)
**19’**	377	0.1230	–	HOMO → LUMO (88%)
	365	0.0889	–	H–1 → LUMO (79%), HOMO → L+1 (11%)
	339	0.0216	–	H–3 → LUMO (61%), H–2 → LUMO (28%)
	338	0.1334	–	H–3 → LUMO (31%), H–2 → LUMO (58%)
	321	0.3111	–	H–1 → LUMO (11%), HOMO → L+1 (76%)
	316	0.5948	317 nm (max)	H–1 → L+1 (76%)
	311	0.148	–	H–4 → LUMO (87%)
	296	0.1159	–	H–5 → LUMO (68%), H–2 → L+1 (23%)
	257	0.1668	–	H–7 → LUMO (19%), H–1 → L+2 (21%), HOMO → L+2 (50%)
	238	0.2548	–	HOMO → L+3 (56%)

***Table S10**: Photophysical data of a number of indolo[3,2-*a*]phenanthridines **9** calculated at the TD-DFT/PBE0/def2-TZVP level with implicit solvent field (CPCM, CH_2Cl_2); only selected transitions >230 nm are listed.

**Compound**	**Calcd. absorbance (nm)**	**Oscillator strength *f***	**Assignment [Exp. Abs. (nm)]**	**Transition (S_0–S_1 vertical)**
**9a**	337	0.0246	–	H–1 → LUMO (30%), HOMO → L+1 (59%)
	316	0.7176	317 (max)	H–1 → LUMO (64%), HOMO → L+1 (33%)
	312	0.2190	317 (max)	H–1 → L+1 (72%), HOMO → LUMO (23%)
	241	0.2932	–	H–4 → LUMO (29%), HOMO → L+3 (52%)
	232	0.1905	230 (max)	H–4 → L+1 (41%), H–1 → L+3 (41%)
**9a·H$^+$**	406	0.0360	–	H–1 → LUMO (74%), HOMO → LUMO (21%)
	400	0.2124	–	H–1 → LUMO (20%), HOMO → LUMO (75%)
	336	0.0861	–	HOMO → L+1 (92%)
	324	0.3288	–	H–1 → L+1 (88%)
	313	0.2270	–	H–2 → LUMO (88%)
	254	0.2173	–	H–4 → LUMO (31%), H–3 → LUMO (18%), H–1 → L+2 (18%)
	233	0.4642	–	H–4 → LUMO (39%), H–1 → L+2 (46%)
	230	0.1833	–	H–5 → LUMO (44%), HOMO → L+3 (28%)
**9b**	336	0.0297	–	H–1 → LUMO (30%), HOMO → L+1 (66%)
	316	0.7676	317 (max)	H–1 → LUMO (66%), HOMO → L+1 (29%)
	311	0.1959	317 (max)	H–1 → L+1 (69%), HOMO → LUMO (25%)
	242	0.2837	–	H–4 → LUMO (20%), HOMO → L+3 (60%)
	233	0.1909	–	H–4 → L+1 (40%), H–1 → L+3 (44%)
**9f**	371 (shoulder)	0.3719	377 (max)	HOMO → LUMO (94%)

	366	0.0251	–	H–1 → LUMO (42%), HOMO → L+1 (55%)
	331	0.1834	–	H–1 → LUMO (55%), HOMO → L+1 (42%)
	320	0.6588	325 (max)	H–1 → L+1 (93%)
	251	0.2156	–	H–5 → LUMO (19%), H–5 → L+1 (11%), H–4 → LUMO (52%)
	243	0.2919	–	H–4 → LUMO (15%), H–1 → L+3 (70%)
	225	0.3389	224 (max)	H–5 → LUMO (47%), H–4 → LUMO (11%)
**9h**	354	0.2654	–	HOMO → LUMO (78%)
	322	0.1903	322 (max)	H–1 → LUMO (56%), HOMO → L+1 (39%)
	319	0.7955	322 (max)	H–1 → L+1 (88%)
	274	0.1588	273 (shoulder)	H–2 → L+1 (30%), H → L+2 (46%)
**9i**	356	0.3121	–	HOMO → LUMO (66%), HOMO → L+1 (20%)
	322	0.1867	324 (max)	H–1→ LUMO (66%), HOMO → L+1 (26%)
	320	0.7472	324 (max)	H–1 → L+1 (85%)
	273	0.1976	275 (max)	HOMO → L+2 (66%), HOMO → L+3 (12%)
	234	0.3805	–	H–5 → LUMO (24%), H–5 → L+1 (14%), H–1 → L+4 (11%), HOMO → L+5 (27%)
**9j**	363	0.2763	–	H–1 → LUMO (12%), HOMO → LUMO (74%)
	327	0.1920	324 (max)	H–1 → LUMO (39%), HOMO → L+1 (57%)
	320	0.7823	–	H–1 → L+1 (91%)
	241	0.2942	275 (shoulder)	H–6 → LUMO (15%), H–5 → LUMO (55%), H–4 → L+1 (14%)
	235	0.3465	275 (shoulder)	H–1 → L+4 (10%), HOMO → L+5 (54%)
**9m**	357	0.2176	–	H–1 → LUMO (17%), HOMO → LUMO (59%), HOMO → L+1 (16%)
	325	0.1888	324 (max)	H–1 → LUMO (47%), HOMO → L+1 (49%)
	320	0.7900	324 (max)	H–1 → L+1 (90%)
	234	0.2775	–	H–2 → L+2 (17%), HOMO → L+5 (42%)

6.3. Chiroptic Properties and Racemization Barriers

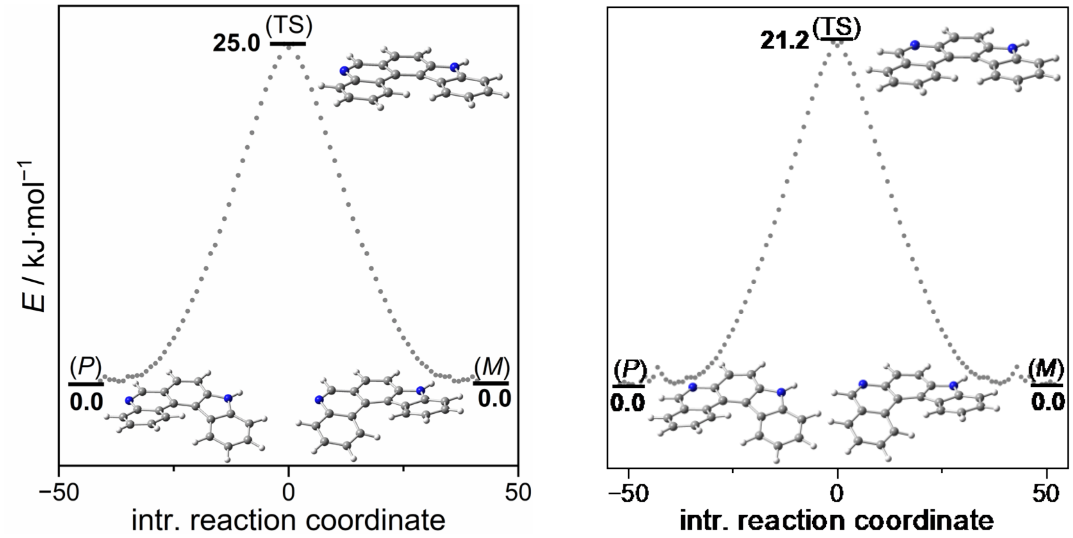

Figure S17: Calculated geometries and energies of enantiomers and transition states for the racemization of parent [2,3-*k*]-IP **3** (left) and [3,2-*a*]-IP **9** (right).

Eyring-Polanyi Equation

Half-life of enantiomerization was calculated by the Eyring-Polanyi equation (SI-1)[51] and equation SI-2 with calculated free Gibbs energies of 25.0·10³ J·mol⁻¹ ([2,3-*k*]-IP, **3**), 21.2·10³ J·mol⁻¹ ([3,2-*a*]-IP, **9**), 0.8·10³ J·mol⁻¹ (ICz, **5**) and 16.0·10³ J·mol⁻¹ (CnCz, **4**).

$$\mathrm{k} = \kappa_e \frac{k_\mathrm{B}T}{h} \exp\left(-\frac{\Delta G^\ddagger}{RT}\right) \qquad \text{(SI-1)}$$

$$t_{½} = \frac{\ln(2)}{\mathrm{k}} \qquad \text{(SI-2)}$$

k: reaction rate constant [s⁻¹]; κ_e: transmission coefficient, $\kappa_e = 0.5$; k_B: Boltzmann constant, $k_B = 1.38 \cdot 10 - 23\ \mathrm{J} \cdot \mathrm{K} - 1$; *T*: temperature, $T = 298.15$ K (25 °C), *h*: Planck constant, $h = 6.63 \cdot 10 - 34\ \mathrm{Js}$; $\Delta G^\ddagger$: Gibbs energy of activation (racemization); *R*: universal gas constant, $R = 8.31\ \mathrm{J \cdot mol^{-1} \cdot K^{-1}}$.

***Table S11**:* Calculated racemization barriers and half-lifes of enantiomerization.

	$\Delta G^\ddagger$ [kJ·mol⁻¹]	$\Delta G^\ddagger$ [kcal·mol⁻¹]	$t_{½}$
Pentahelicene		24.1[51]	29 h
ICz (**5**)	0.8	0.2	0.03 ns
CnCz (**4**)	16.0	3.8	0.14 ns
[2,3-*k*]-IP **3**	25.0	6.0	5.58 ns
[3,2-*a*]-IP **9**	21.2	5.1	1.22 ns

pK_a Values

Relative pK_a values were calculated by the proton exchange method.[62] Values were determined by correlating the compounds' solvent-dependent free energies with an experimental pK_a value of pyridinium as a reference (Ref-H$^+$; pK_a = 5.23 at 25 °C[63]) [R = 8.3144621 J·mol^{-1}·K^{-1}; T = 298.15 K (25 °C)]; eq. SI-3.

$$\mathrm{p}K_\mathrm{a}\,(\text{A-H}) = \frac{\Delta G^*_\mathrm{soln}}{RT\ln(10)} + \mathrm{p}K_\mathrm{a}\,(\text{Ref-H}^+) \qquad \text{(SI-3)}$$

Table S12: Calculated relative pK_a values of selected aza[5]helicenes.

**Compound**	**Relative pK_a**
CnCz (**4**)	6.65
[2,3-*k*]-IP **3a** (R=Me)	10.0
[3,2-*a*]-IP **9a** (R=Me)	9.55

Proton Affinities

Proton affinities *PA* for the gas phase reaction of neutral indolophenanthridines (IP) with a proton can be estimated by eq. SI-4:[64]

$$\text{IP} + \text{H}^+ \rightarrow \text{IP-H}^+$$

$$PA = E(\text{IP}) + E(\text{H}^+) - E(\text{IP-H}^+) \qquad \text{(SI-4)}$$

The difference of the proton affinities Δ*PA* of indolophenanthridines **9** and **3** was calculated using their zero point-corrected energies by eq. SI-5:

$$\Delta PA = PA_{[3,2\text{-}a]} - PA_{[2,3\text{-}k]} \qquad \text{(SI-5)}$$

$$= E(\text{IP}_{[2,3\text{-}k]}) + E(\text{IP}_{[2,3\text{-}k]}\text{-H}^+) - E(\text{IP}_{[3,2\text{-}a]}) - E(\text{IP}_{[3,2\text{-}a]}\text{-H}^+)$$

$$= 0.013 \text{ Hartree} = 8.2 \text{ kcal·mol}^{-1} = 34.3 \text{ kJ·mol}^{-1}$$

7. Appendix

7.1. ¹H NMR and ¹³C NMR Spectra

N-[2-(9*H*-Carbazol-4-yl)phenyl]-2,2,2-trifluoroacetamide (2c)

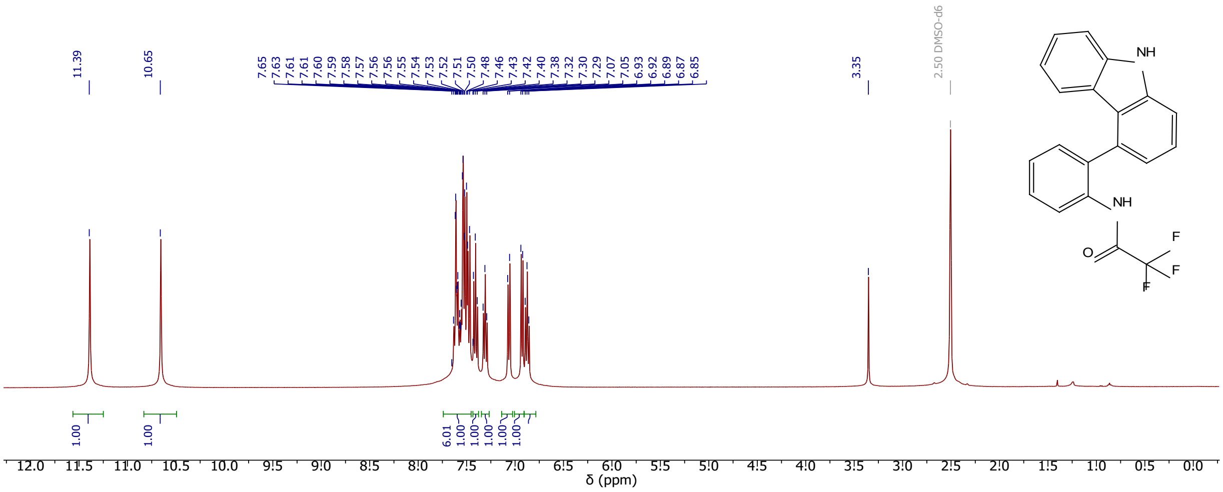

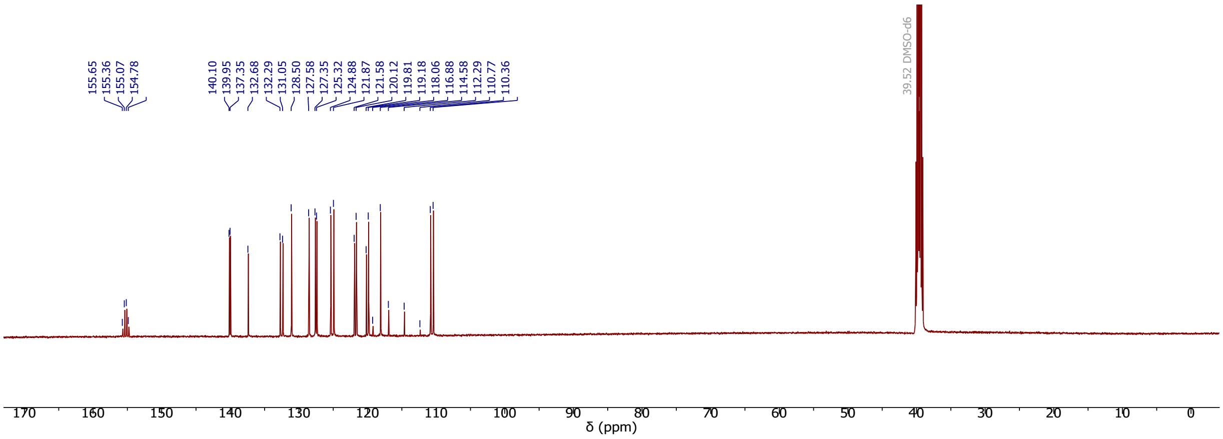

N-[2-(9*H*-Carbazol-4-yl)phenyl]-2-chloroacetamide (2d)

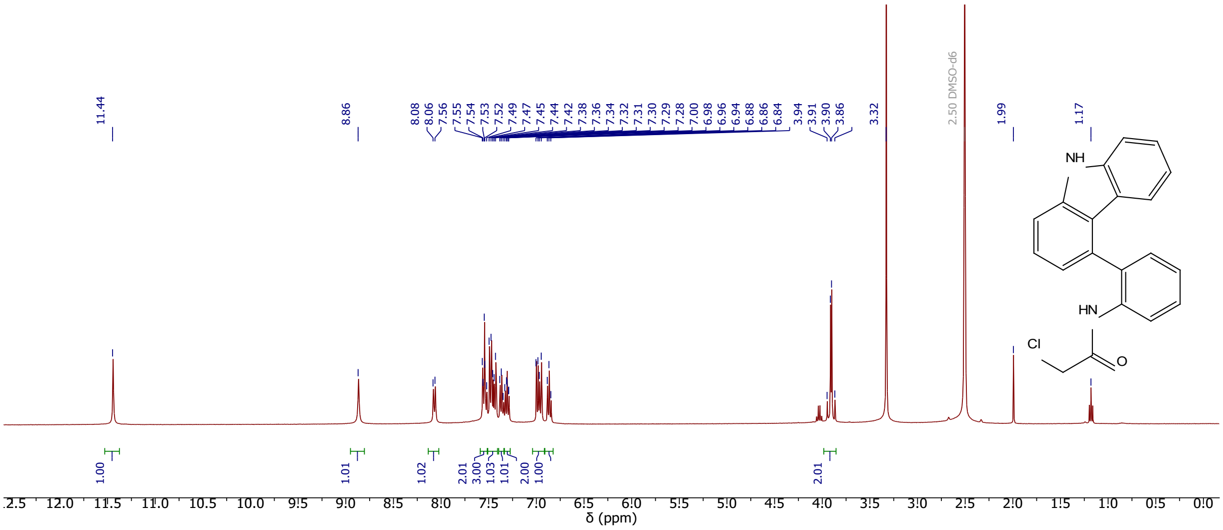

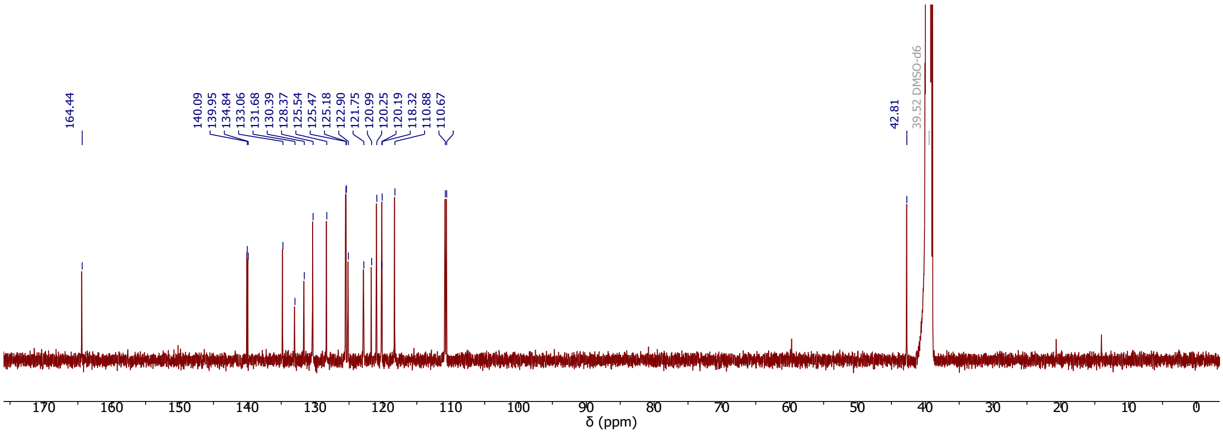

N-[2-(9*H*-Carbazol-4-yl)phenyl]-2-azidoacetamide (2e)

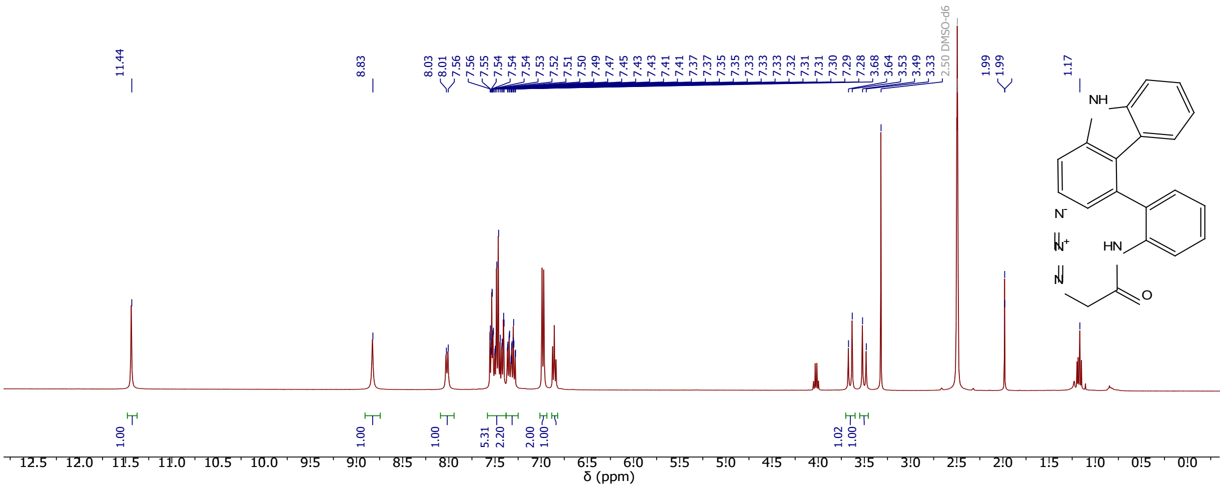

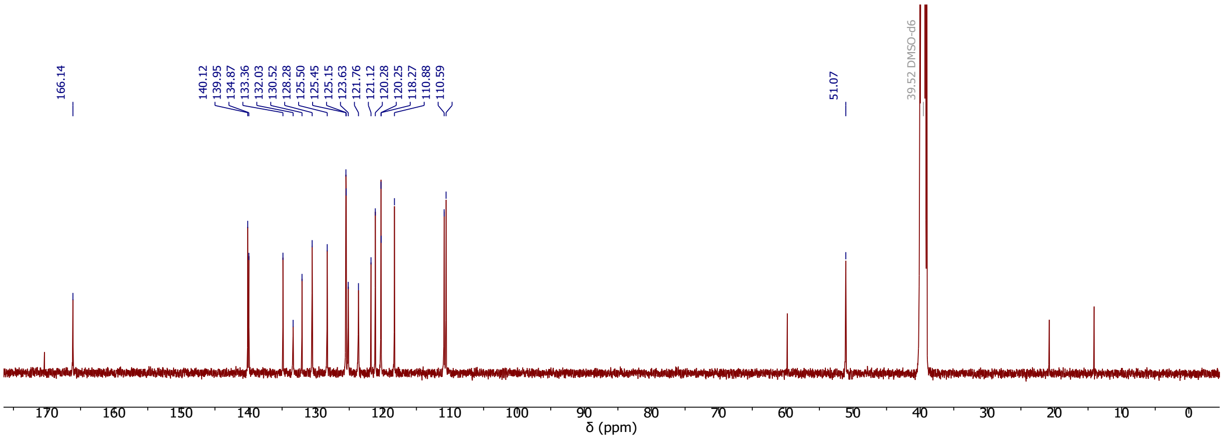

(2*E*,4*E*)-*N*-[2-(9*H*-Carbazol-4-yl)phenyl]hexa-2,4-dienamide (2g)

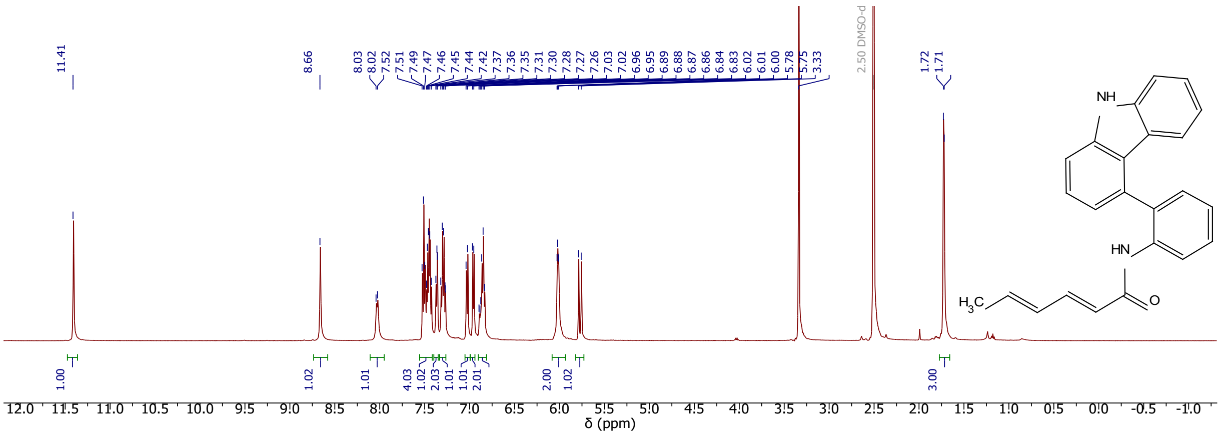

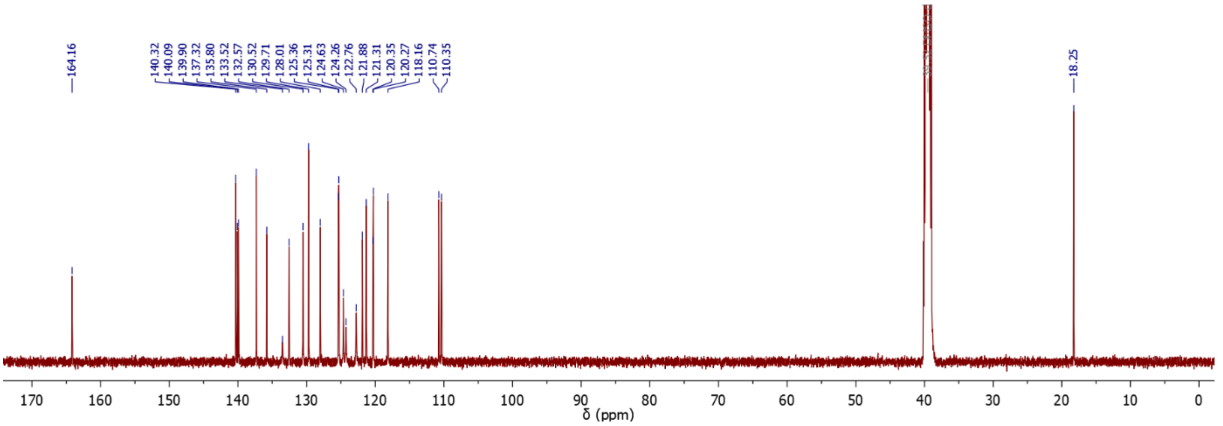

N-[2-(*9*H-Carbazol-4-yl)phenyl]-4-methoxybenzamide (2i)

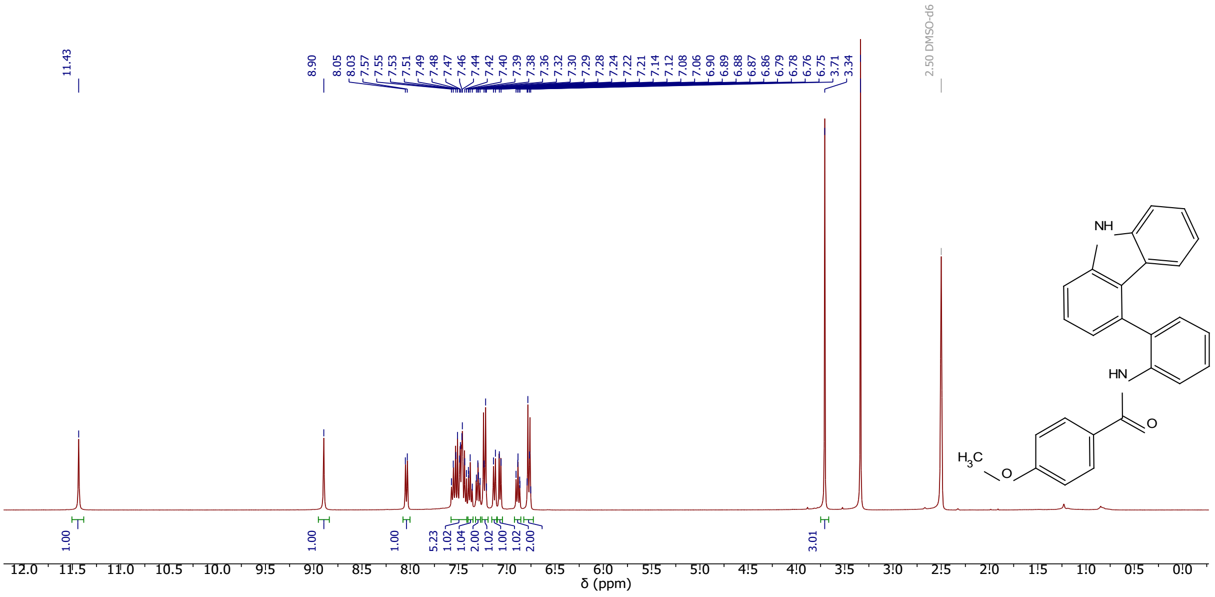

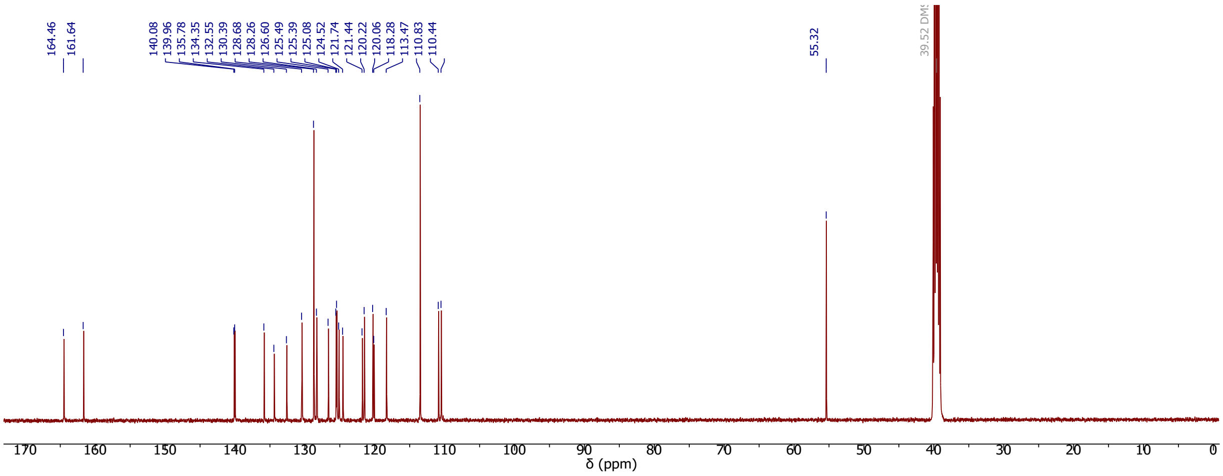

N-[2-(9*H*-Carbazol-4-yl)phenyl]-4-(trifluoromethyl)benzamide (2j)

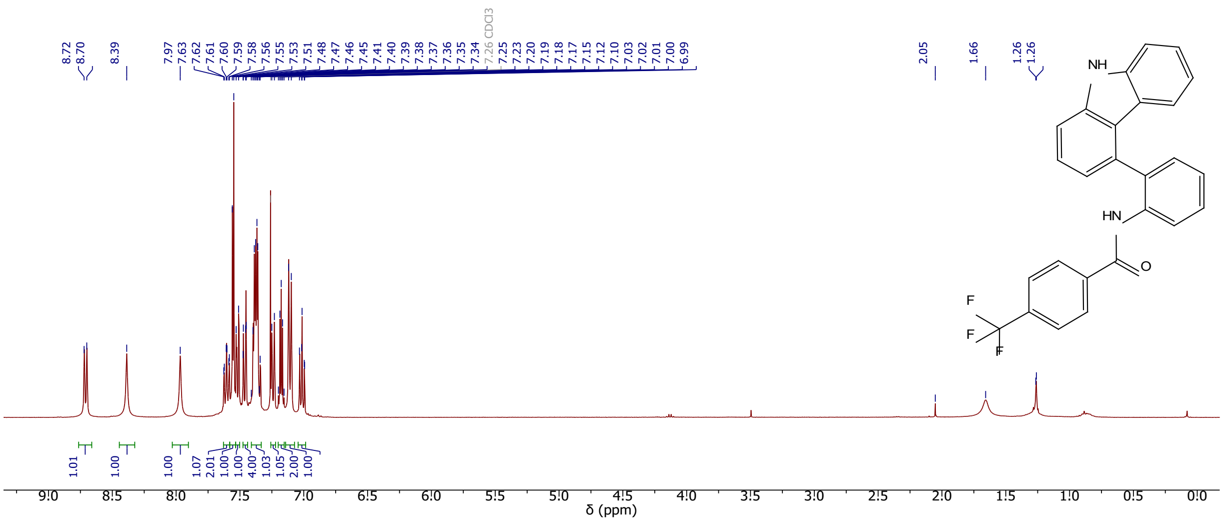

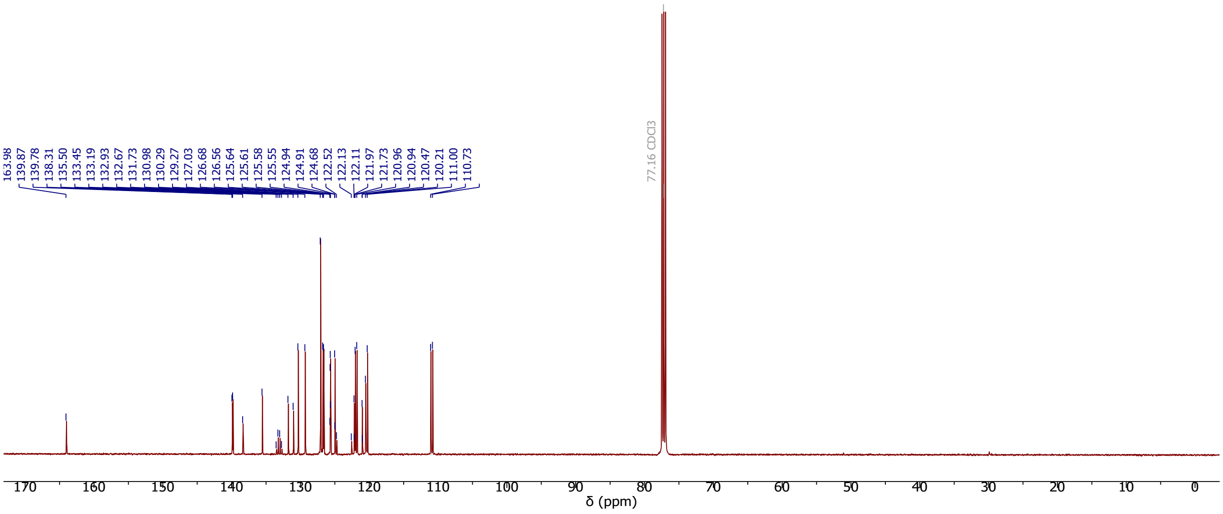

N-(2-(9*H*-carbazol-4-yl)phenyl)picolinamide (2l)

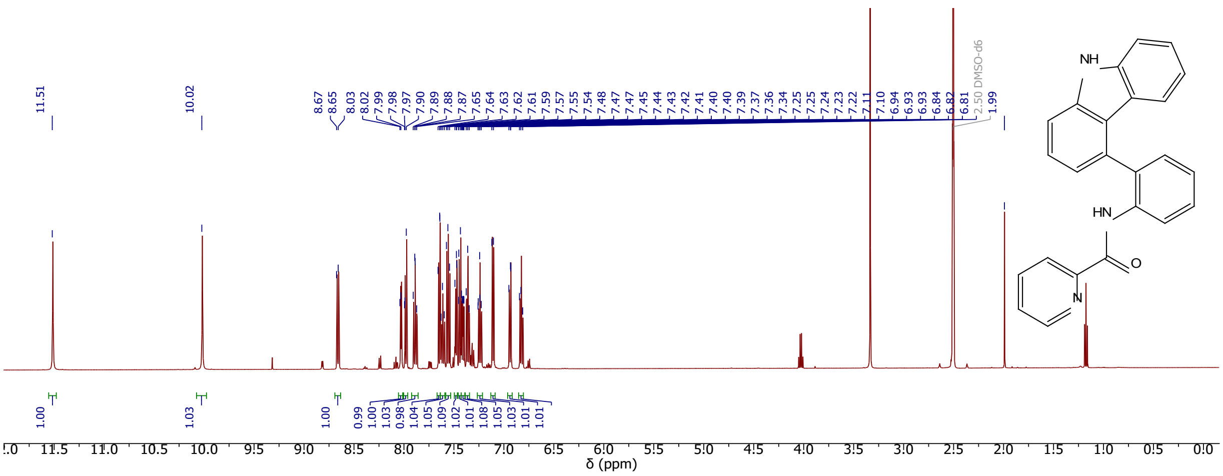

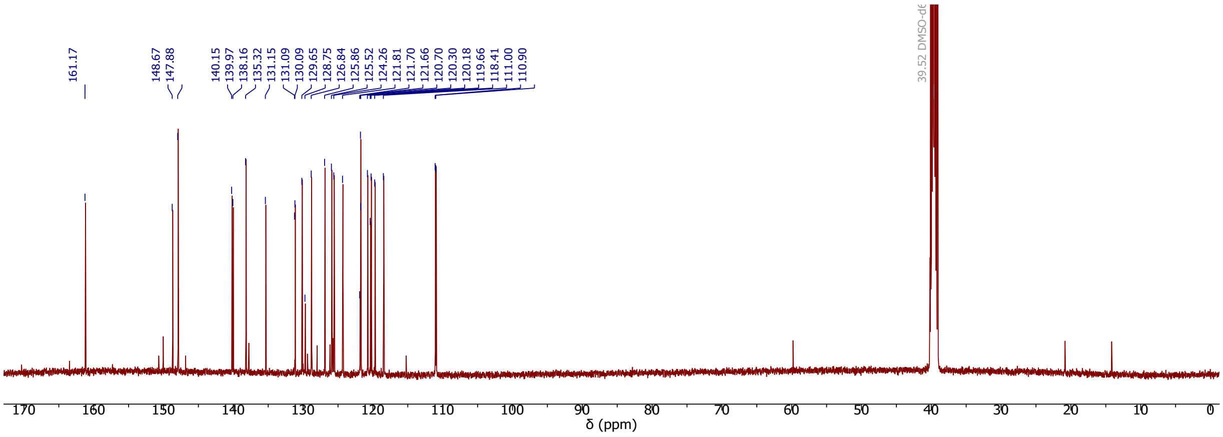

***N*-(2-(9*H*-carbazol-4-yl)phenyl)-1-naphthamide (2n)**

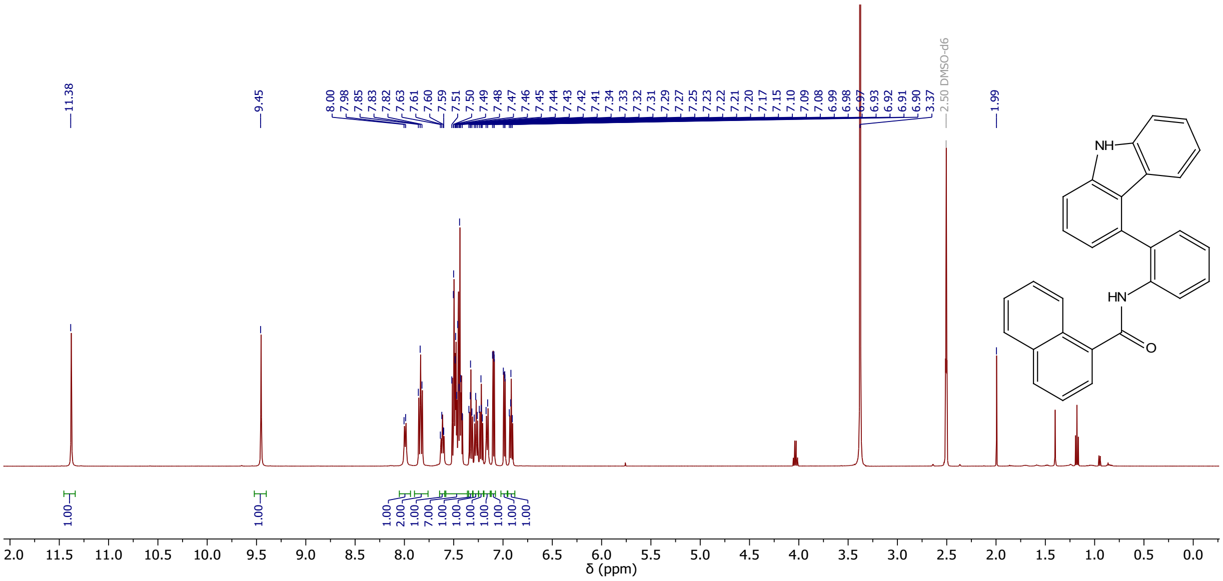

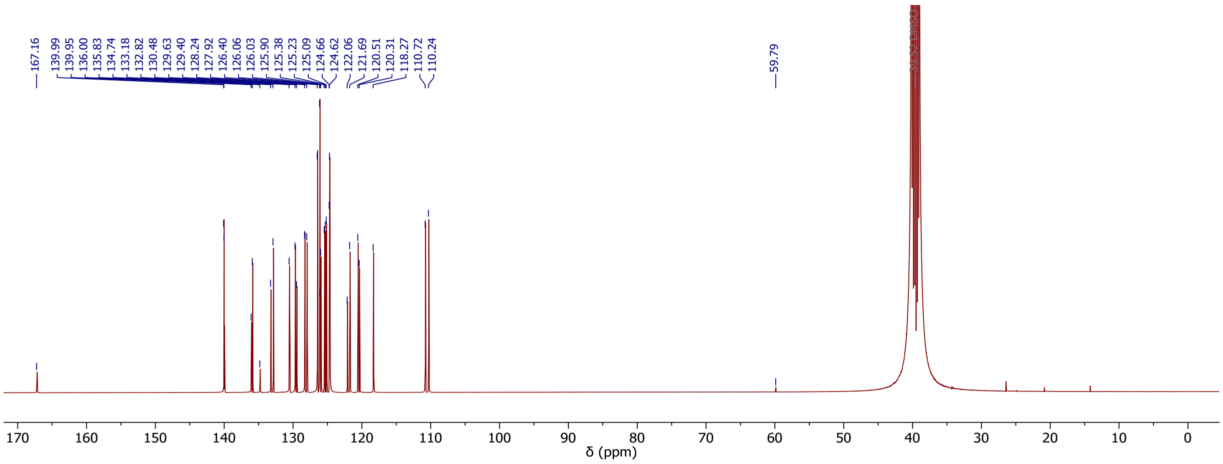

6-(Trifluoromethyl)-9*H*-indolo[2,3-*k*]phenanthridine (3c)

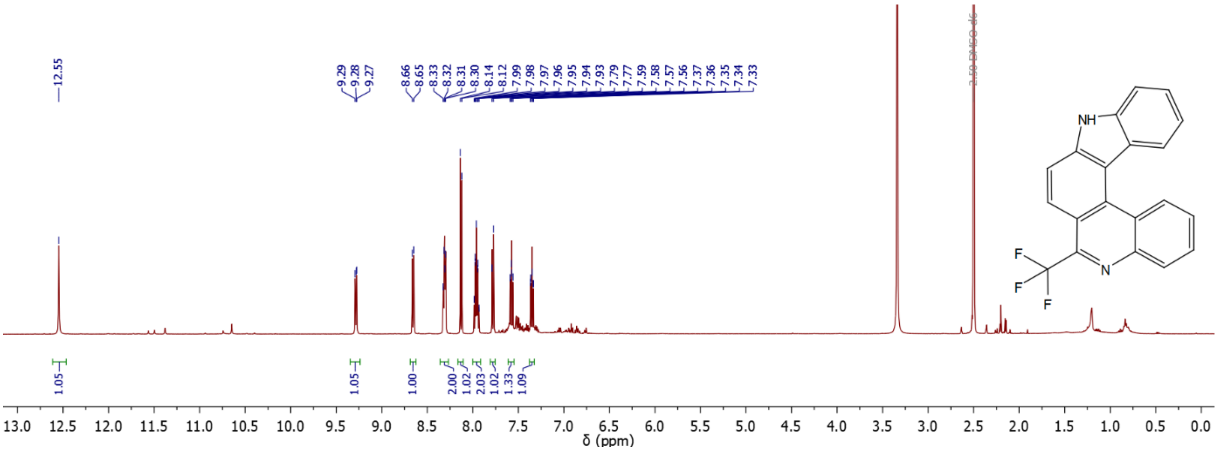

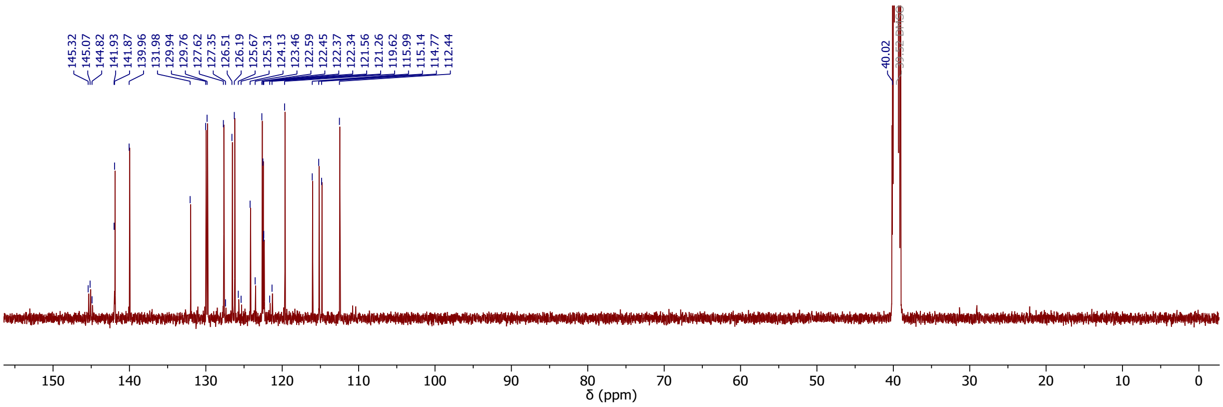

6-(Chloromethyl)-9*H*-indolo[2,3-*k*]phenanthridine (3d)

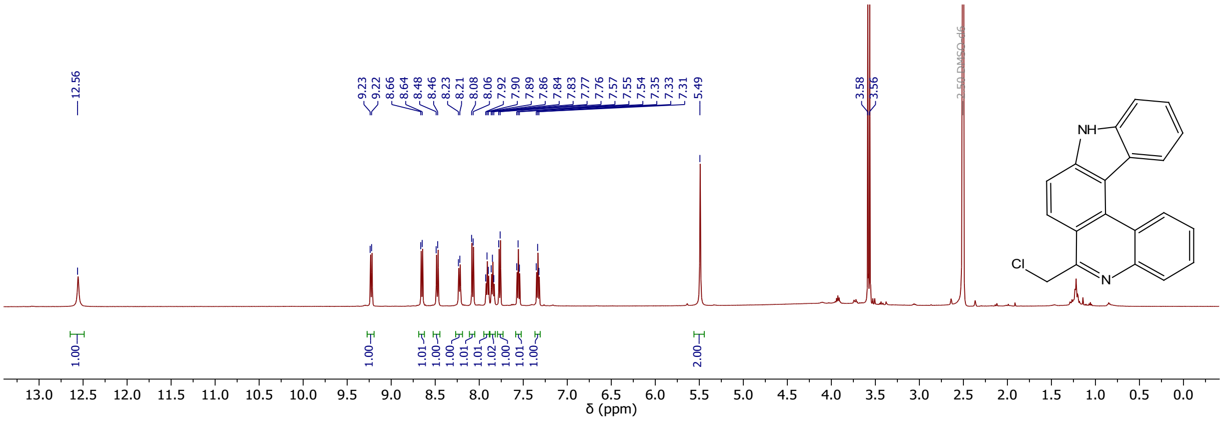

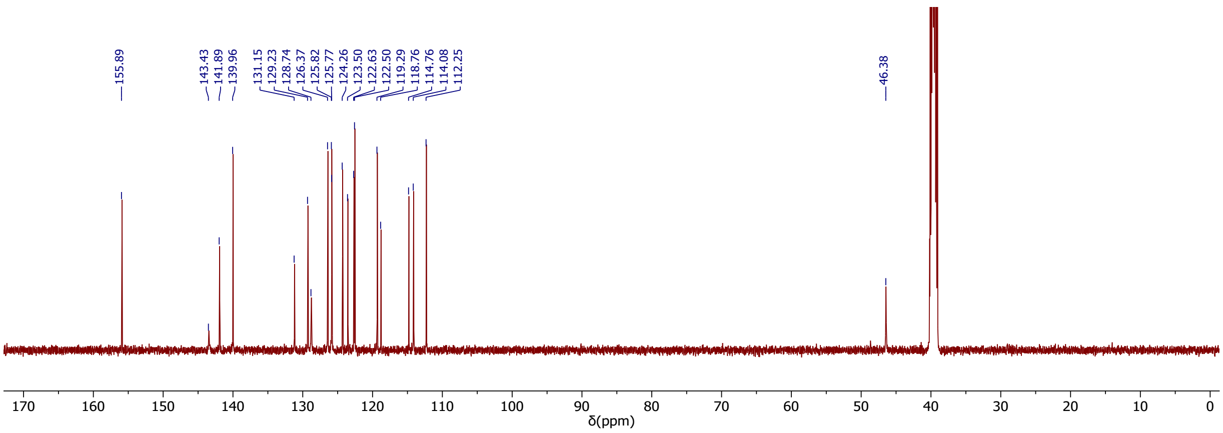

6-(Azidomethyl)-9*H*-indolo[2,3-*k*]phenanthridine (3e)

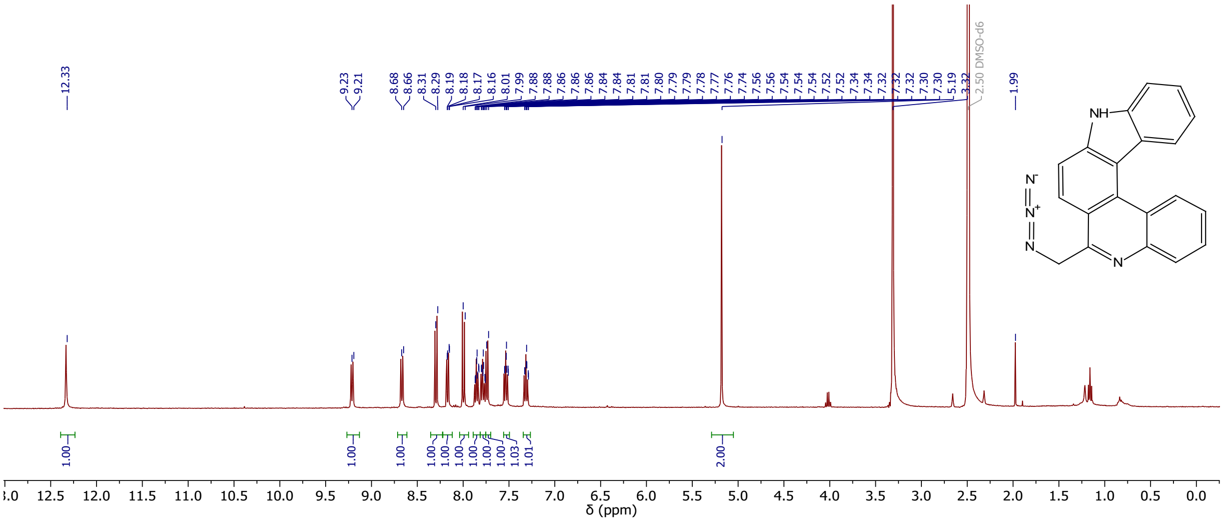

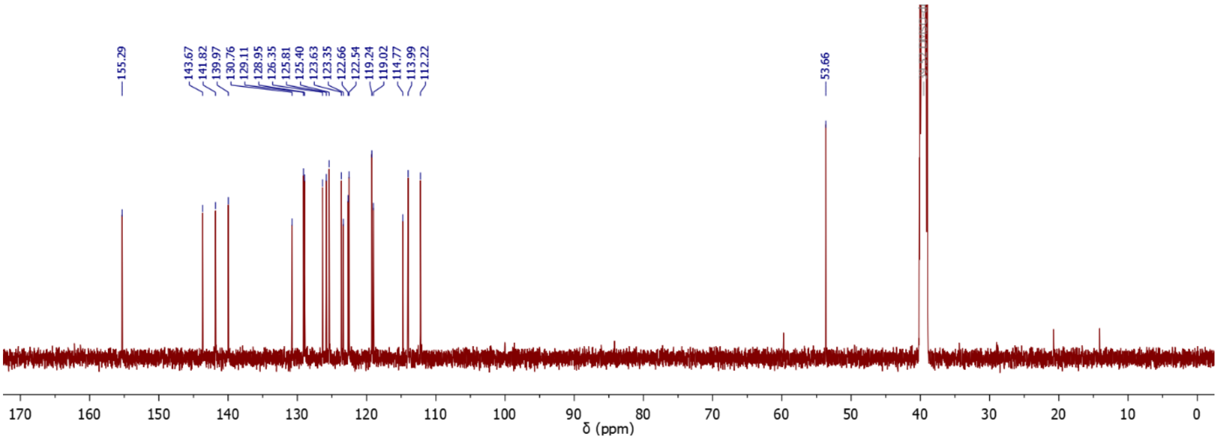

6-[(1*E*,3*E*)-Penta-1,3-dien-1-yl]-9*H*-indolo[2,3-*k*]phenanthridine (3g)

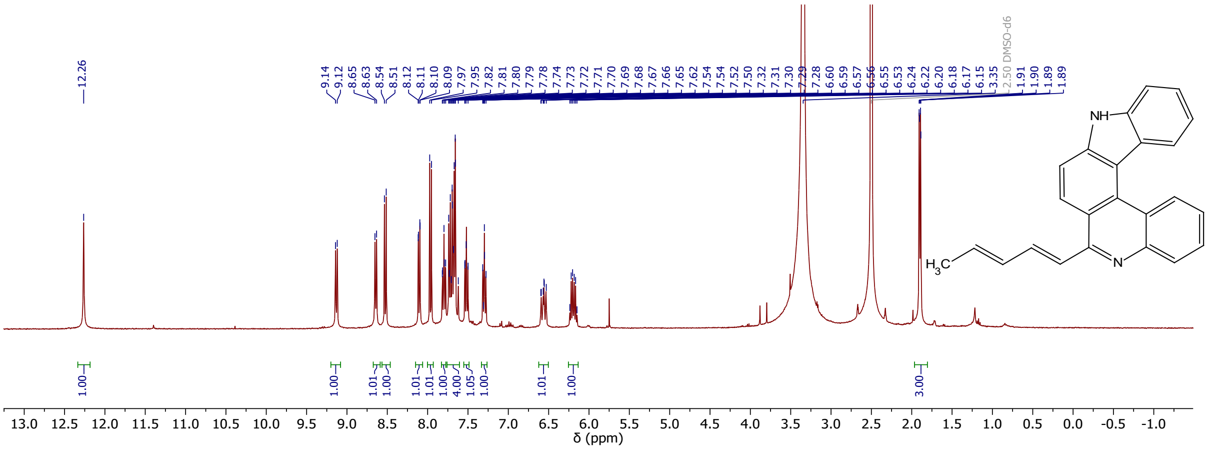

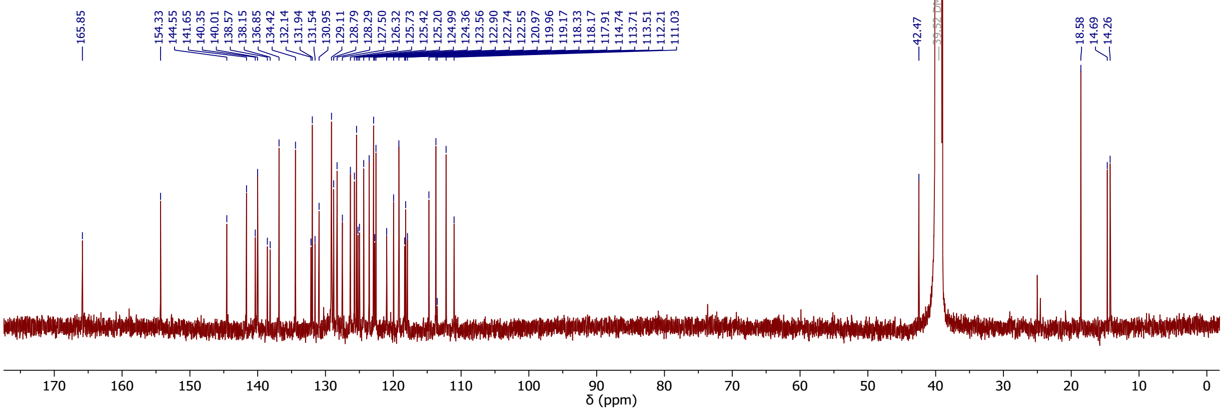

6-(4-Methoxyphenyl)-9*H*-indolo[2,3-*k*]phenanthridine (3i)

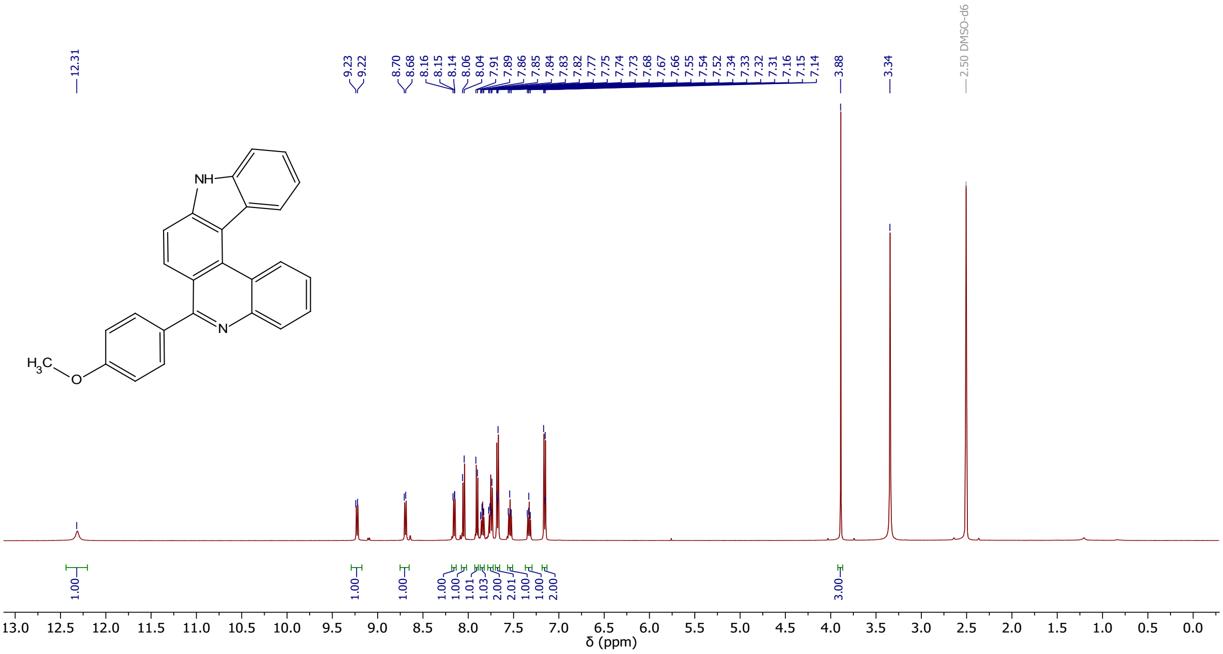

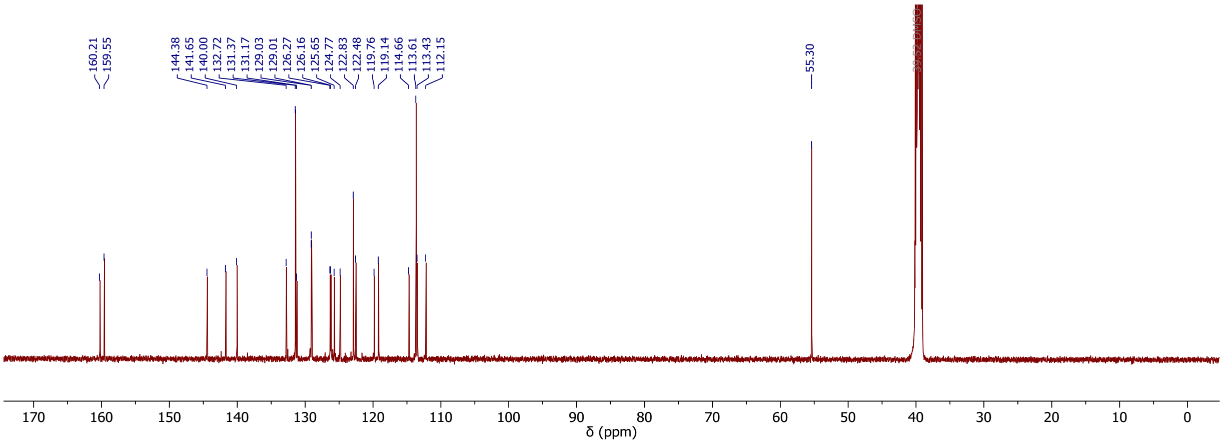

6-[4-(Trifluoromethyl)phenyl]-9*H*-indolo[2,3-*k*]phenanthridine (3j)

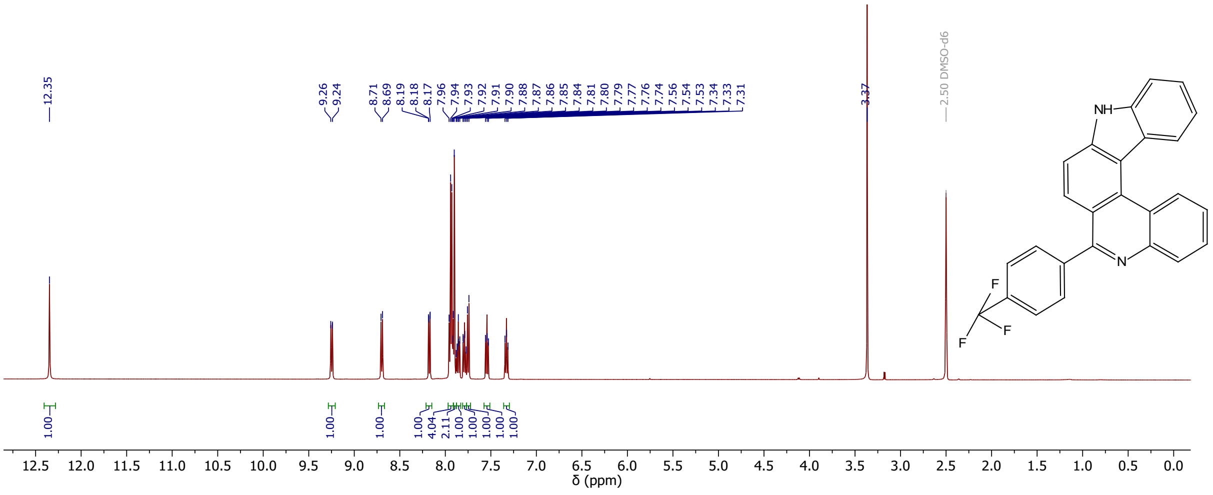

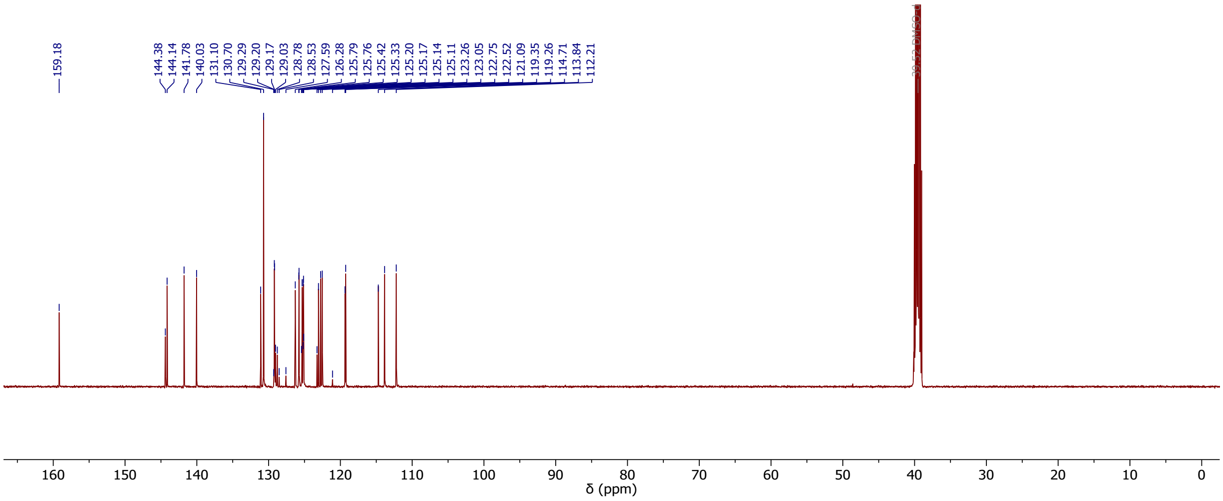

6-(Pyridine-2-yl)-9*H*-indolo[2,3-*k*]phenanthridine (3l)

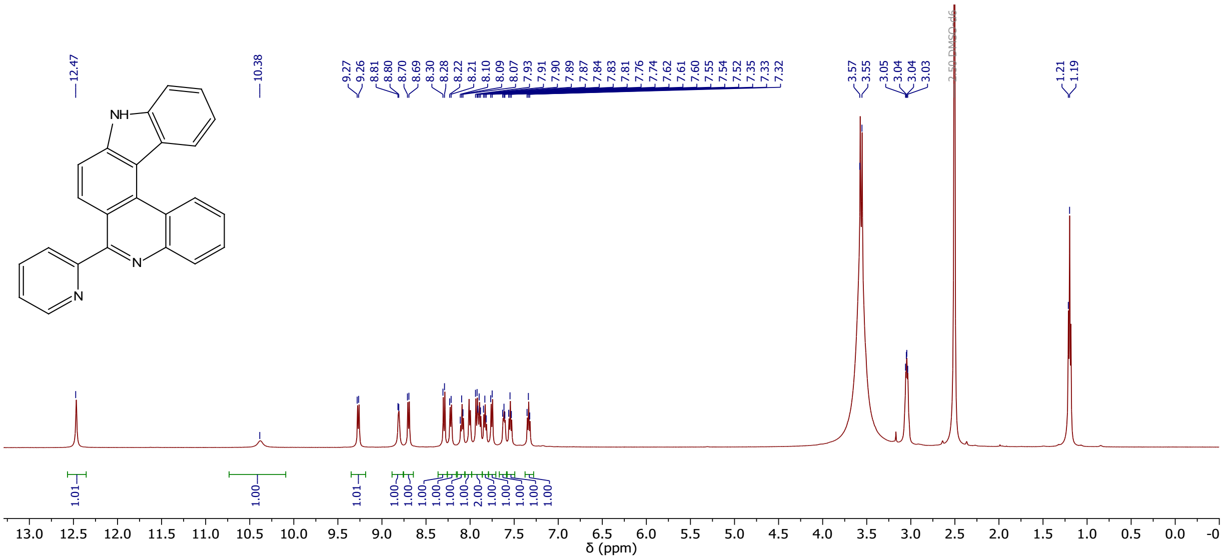

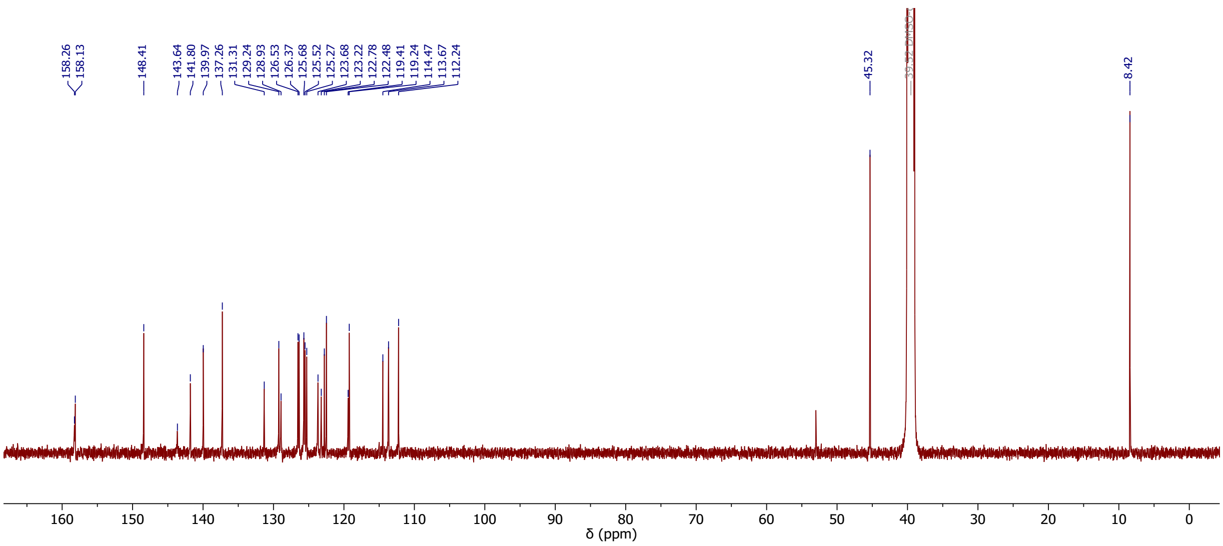

6-(Naphthalene-1-yl)-9*H*-indolo[2,3-*k*]phenanthridine (3n)

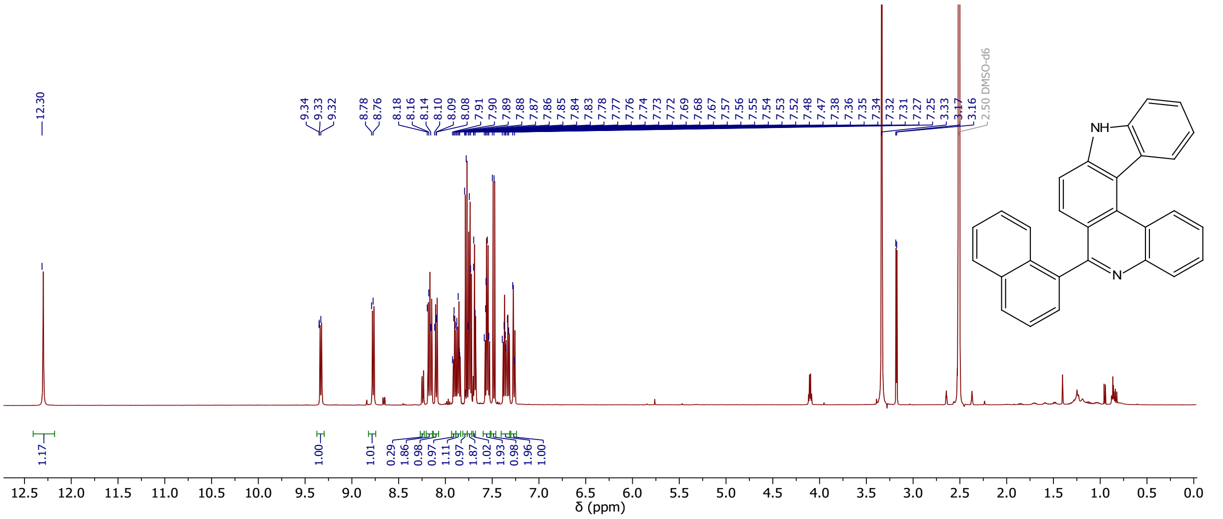

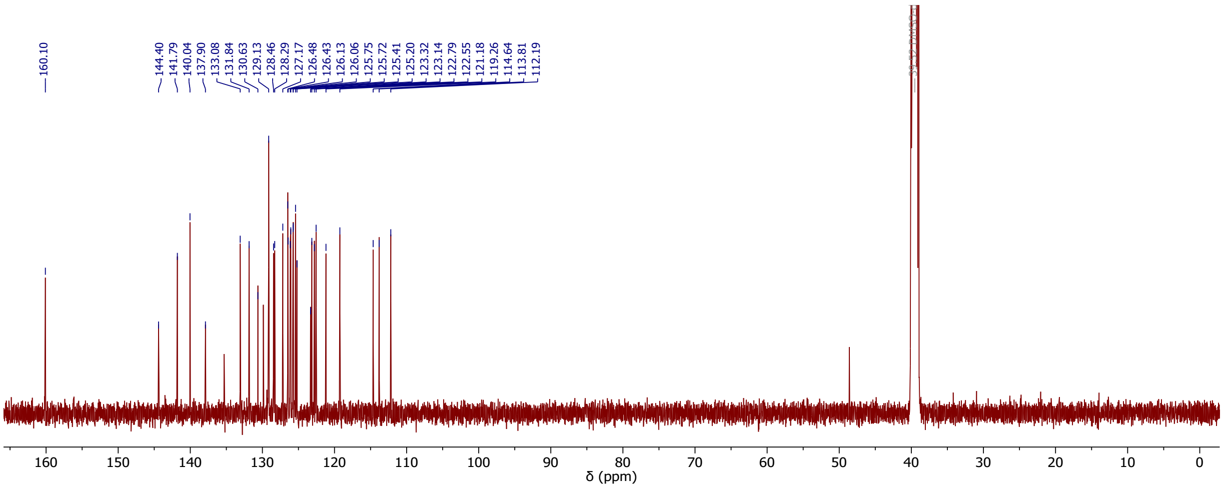

N-(1,2-Dimethyl-4-phenyl-9*H*-carbazol-3-yl)-2,2,2-trifluoroacetamide (8c)

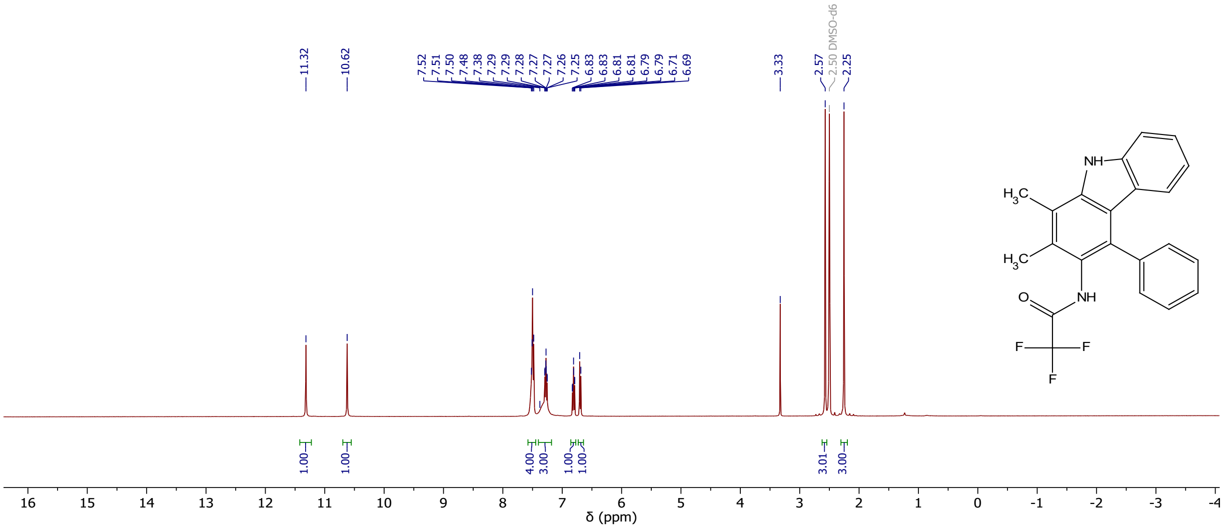

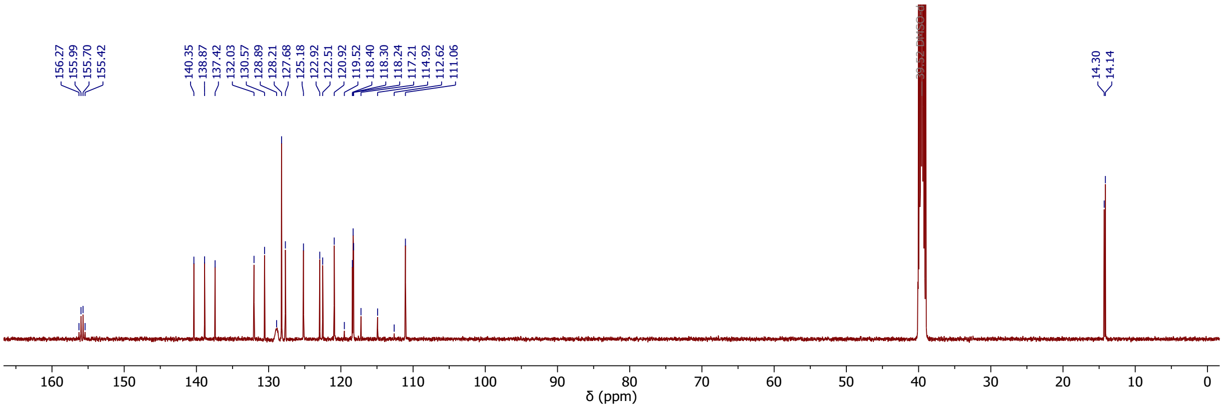

2-Chloro-*N*-(1,2-dimethyl-4-phenyl-9*H*-carbazol-3-yl)acetamide (8d)

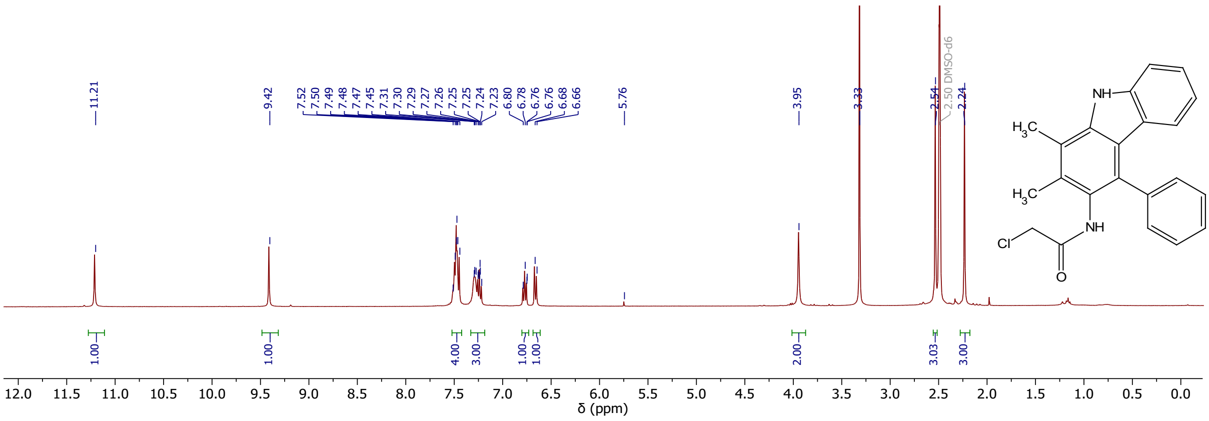

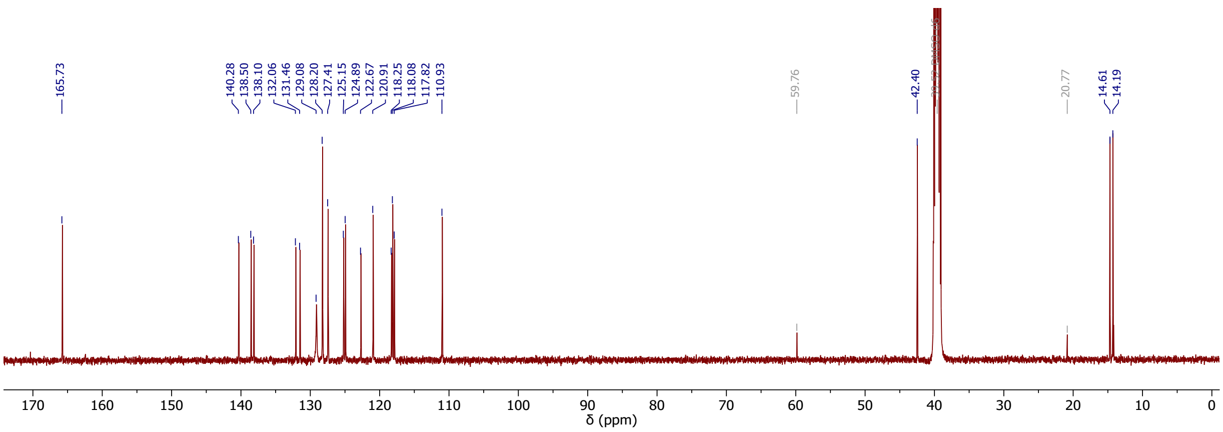

2-Azido-*N*-(1,2-dimethyl-4-phenyl-9*H*-carbazol-3-yl)acetamide (8e)

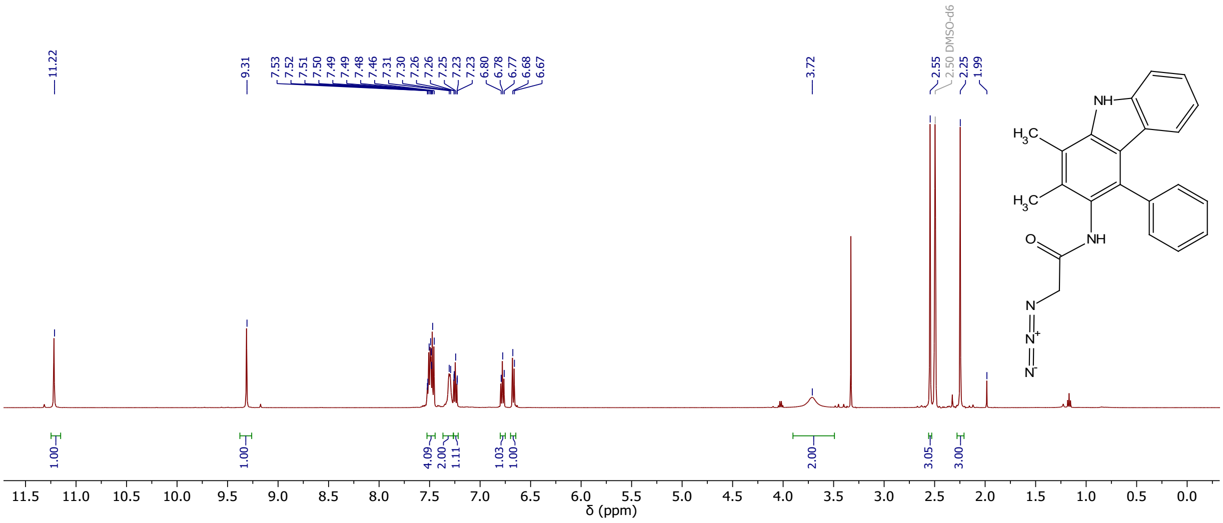

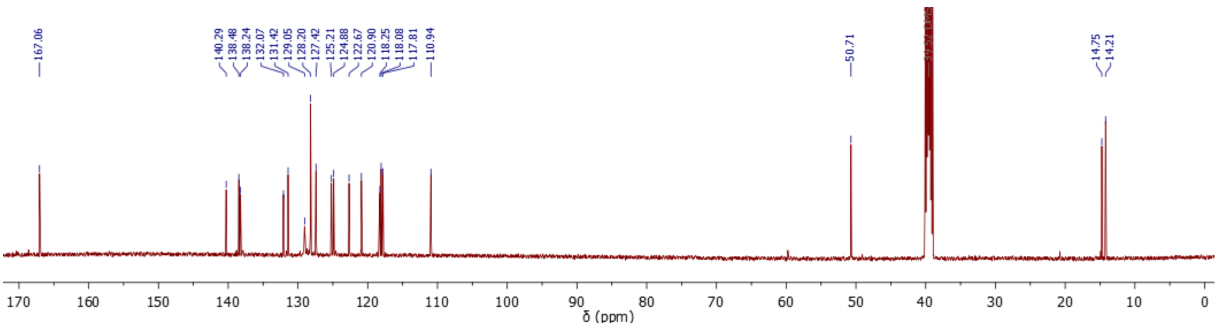

(2*E*,4*E*)-*N*-(1,2-dimethyl-4-phenyl-9*H*-carbazol-3-yl)hexa-2,4-dienamide (8g)

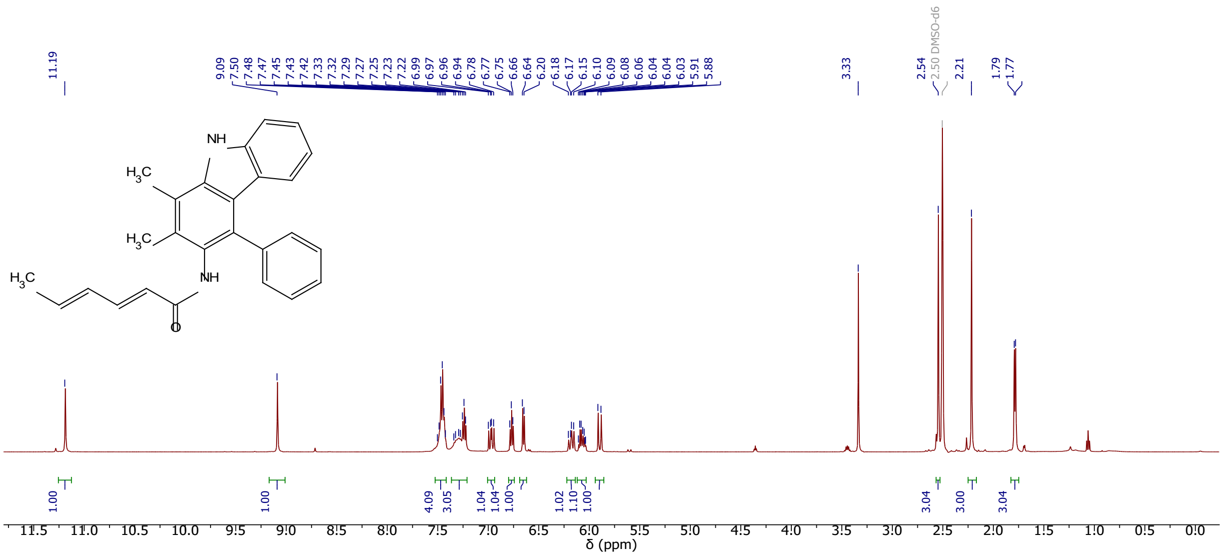

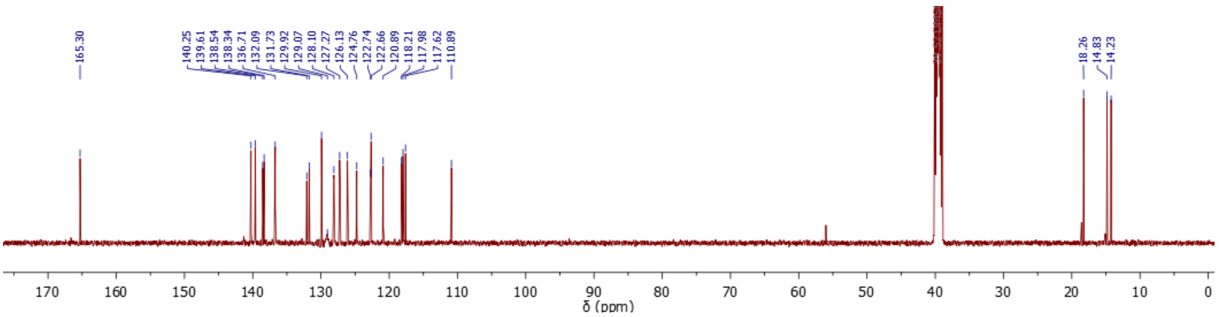

N-(1,2-Dimethyl-4-phenyl-9*H*-carbazol-3-yl)-4-methoxybenzamide (8i)

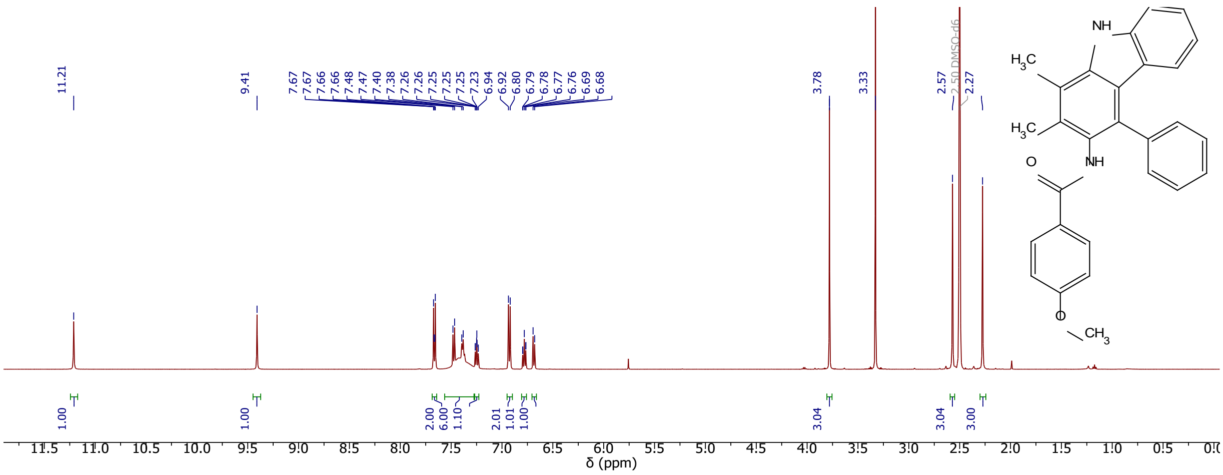

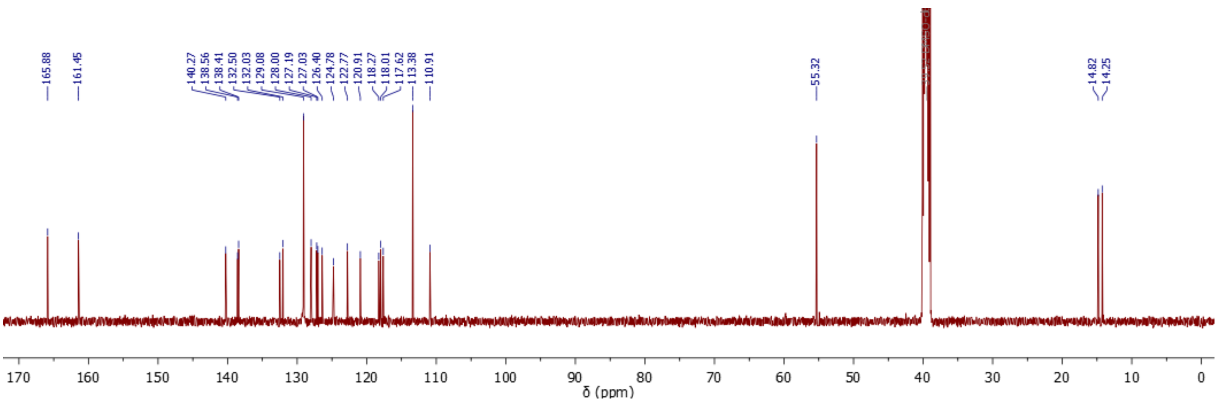

N-(1,2-Dimethyl-4-phenyl-9*H*-carbazol-3-yl)-4-(trifluoromethyl)benzamide (8j)

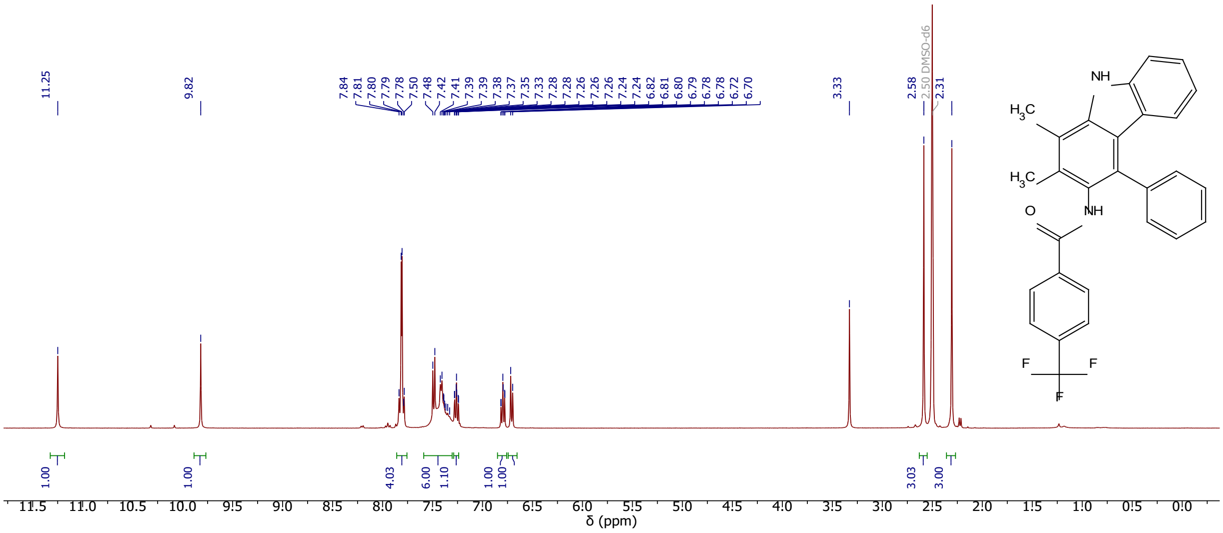

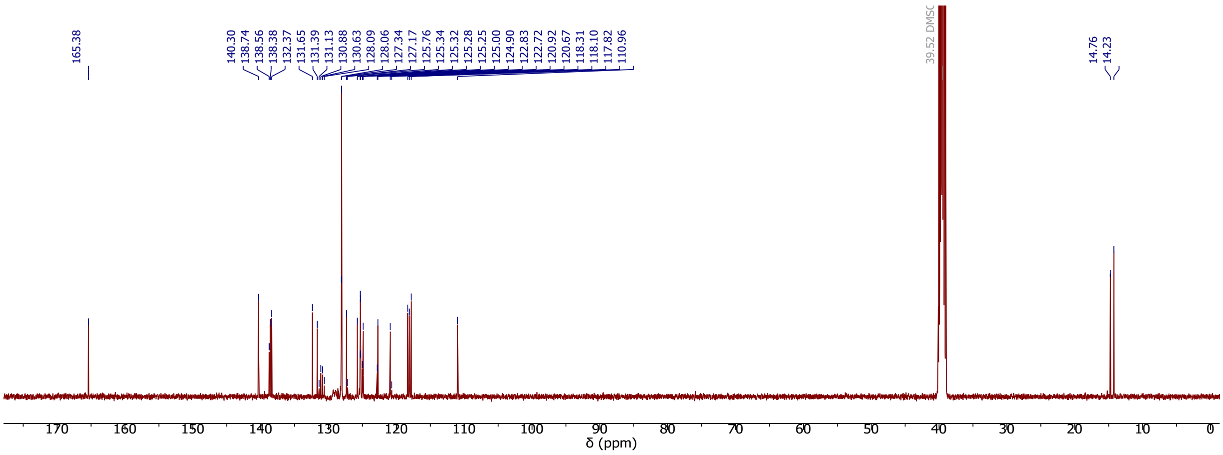

N-(1,2-Dimethyl-4-phenyl-9*H*-carbazol-3-yl)-2,4,6-trimethylbenzamide (8k)

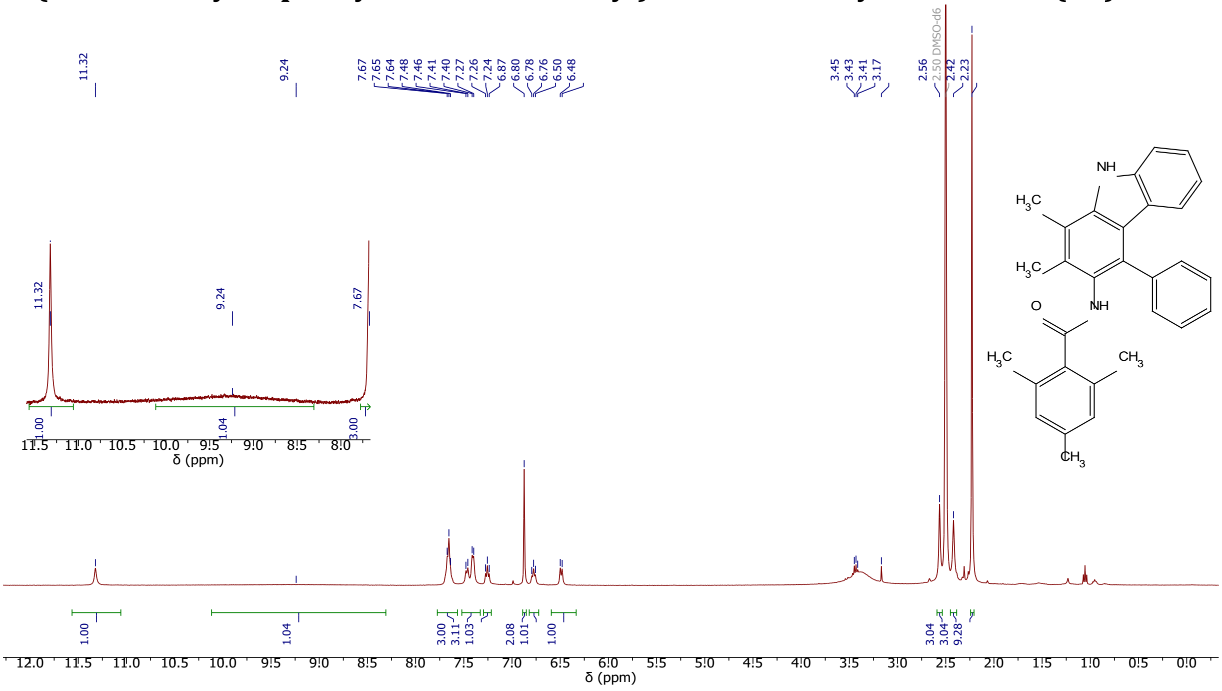

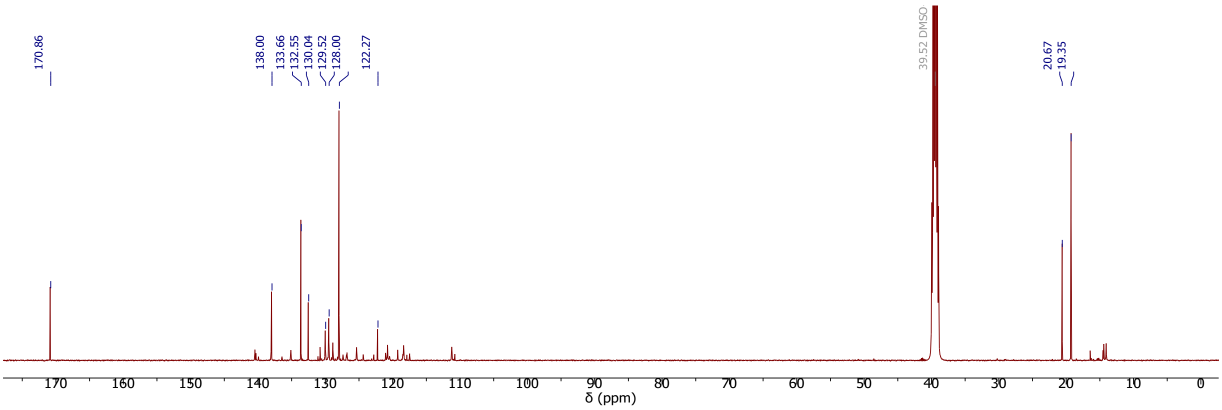

5-(Chloromethyl)-7,8-dimethyl-9*H*-indolo[3,2-*a*]phenanthridine (9d)

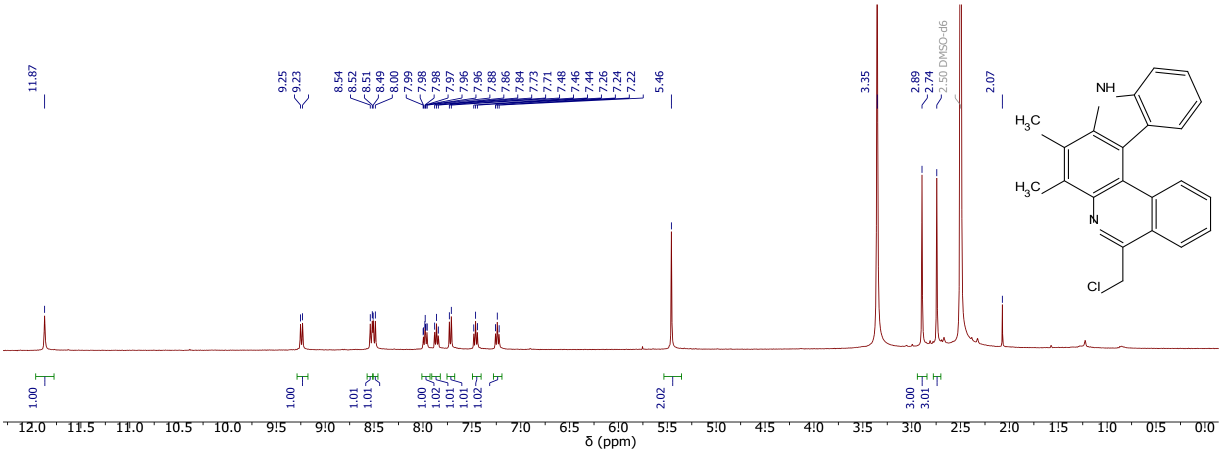

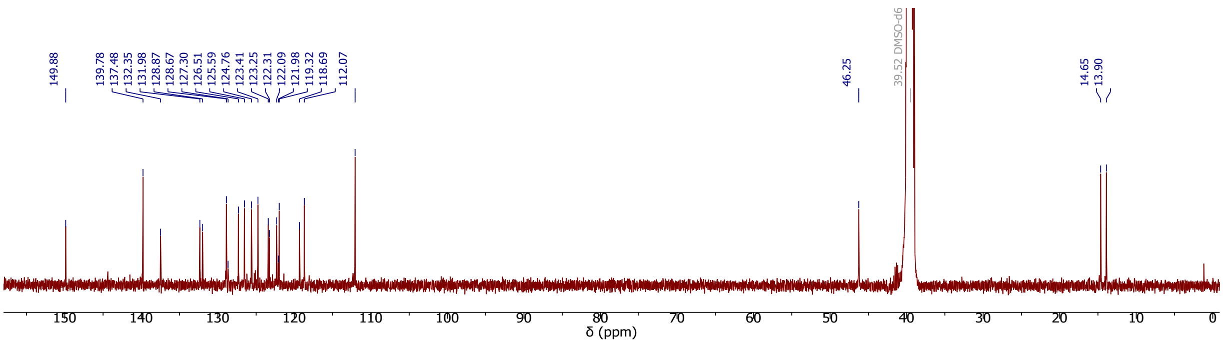

5-(Azidomethyl)-7,8-dimethyl-9*H*-indolo[3,2-*a*]phenanthridine (9e)

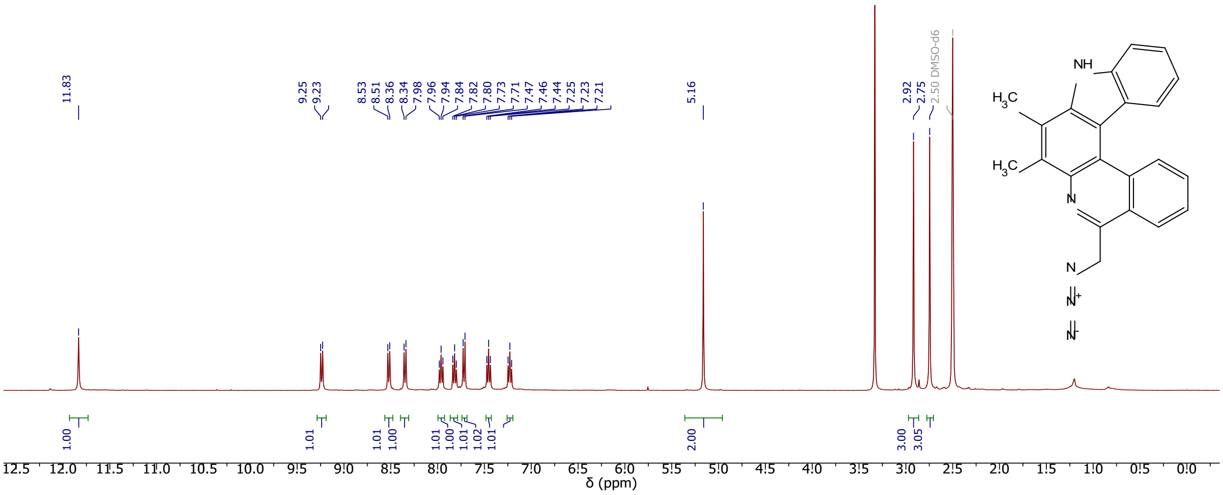

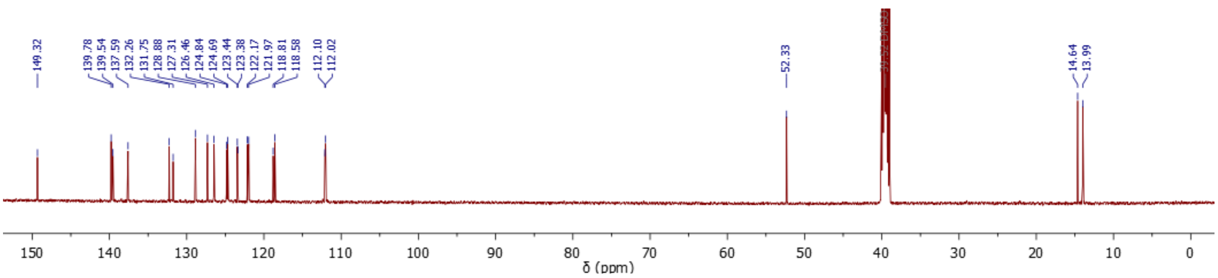

7,8-Dimethyl-5-[(1*E*,3*E*)-penta-1,3-dien-1-yl]-9*H*-indolo[3,2-*a*]phenanthridine (9g)

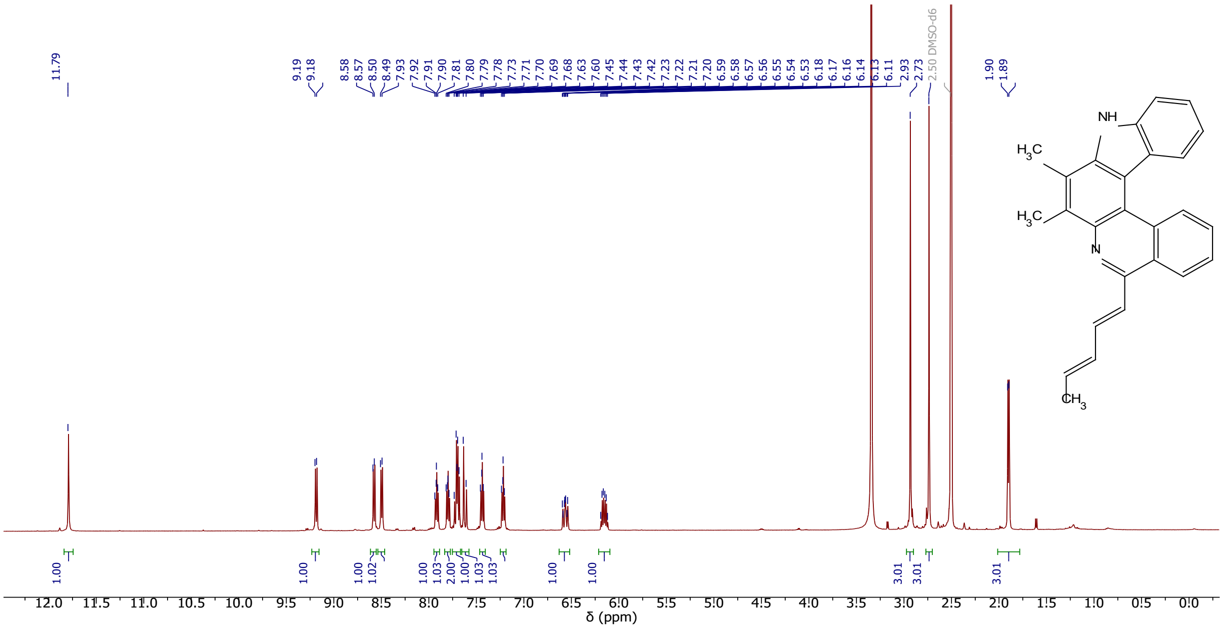

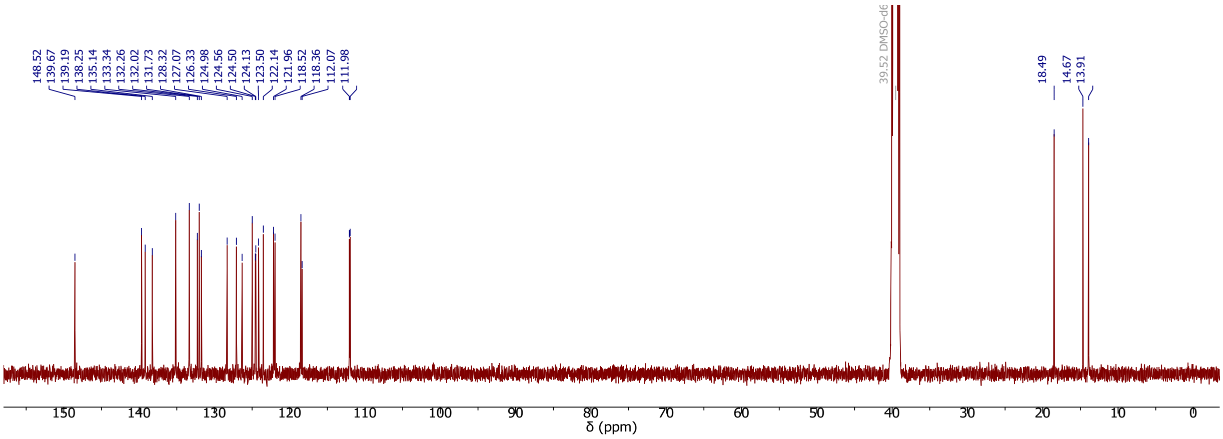

5-(4-Methoxyphenyl)-7,8-dimethyl-9*H*-indolo[3,2-*a*]phenanthridine (9i)

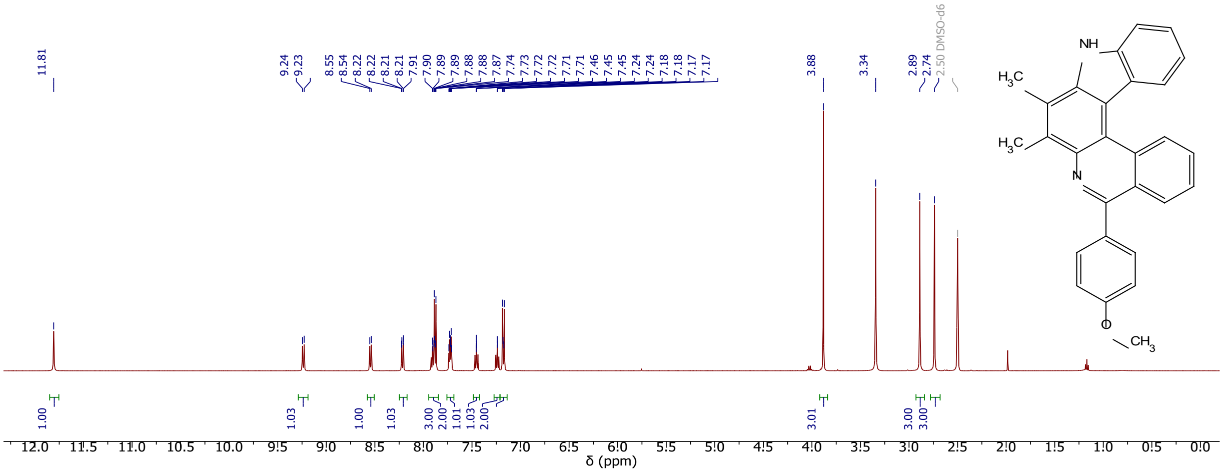

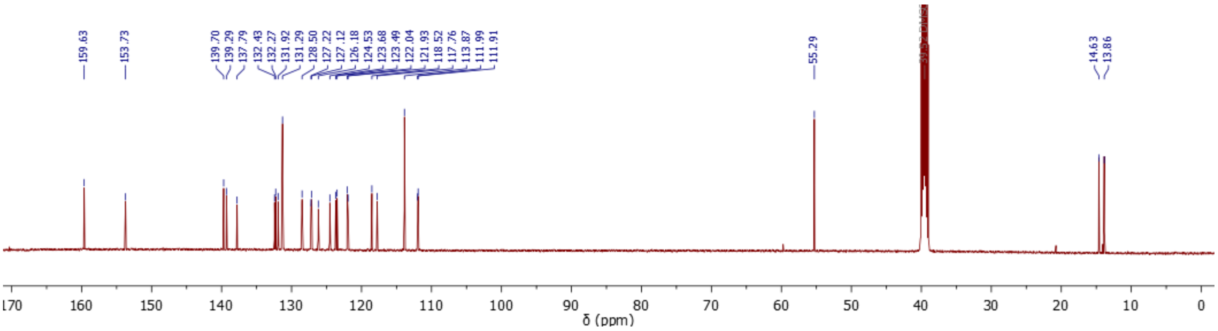

7,8-Dimethyl-5-[4-(trifluoromethyl)phenyl]-9*H*-indolo[3,2-*a*]phenanthridine (9j)

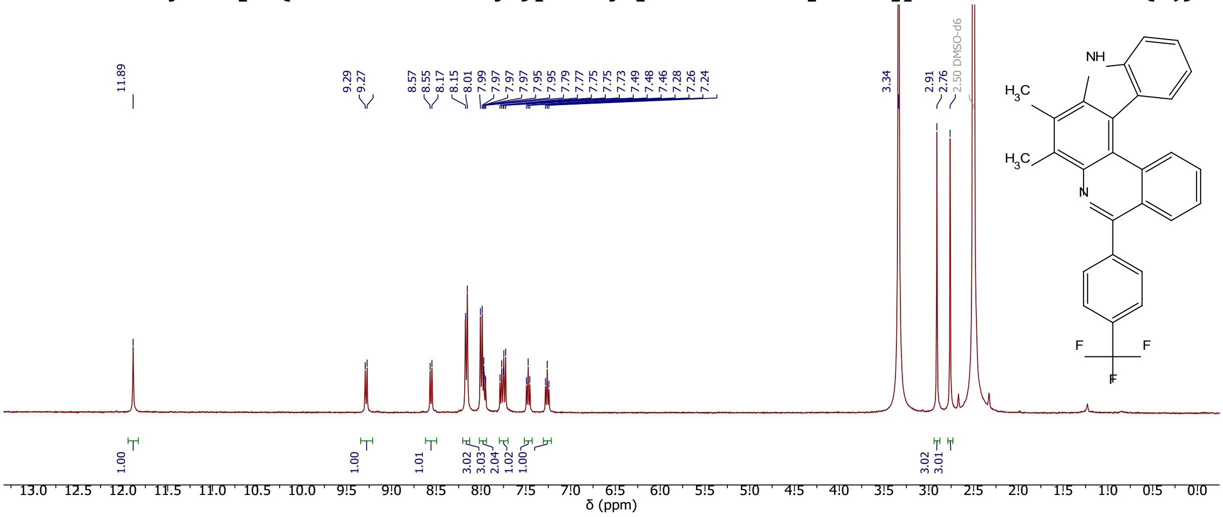

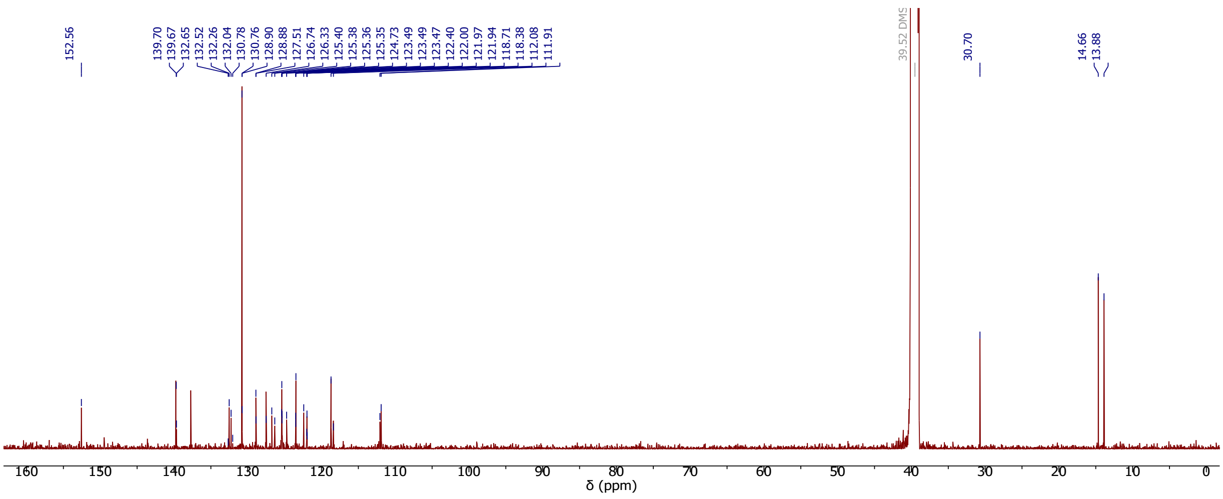
152.56
139.70
139.67
132.65
132.52
132.26
132.04
130.78
130.76
128.90
128.88
127.51
126.74
126.33
125.40
125.38
125.36
125.35
124.73
123.49
123.47
122.40
122.00
121.97
121.94
118.71
118.38
112.08
111.91
39.52 DMS
30.70
14.66
13.88
δ (ppm)

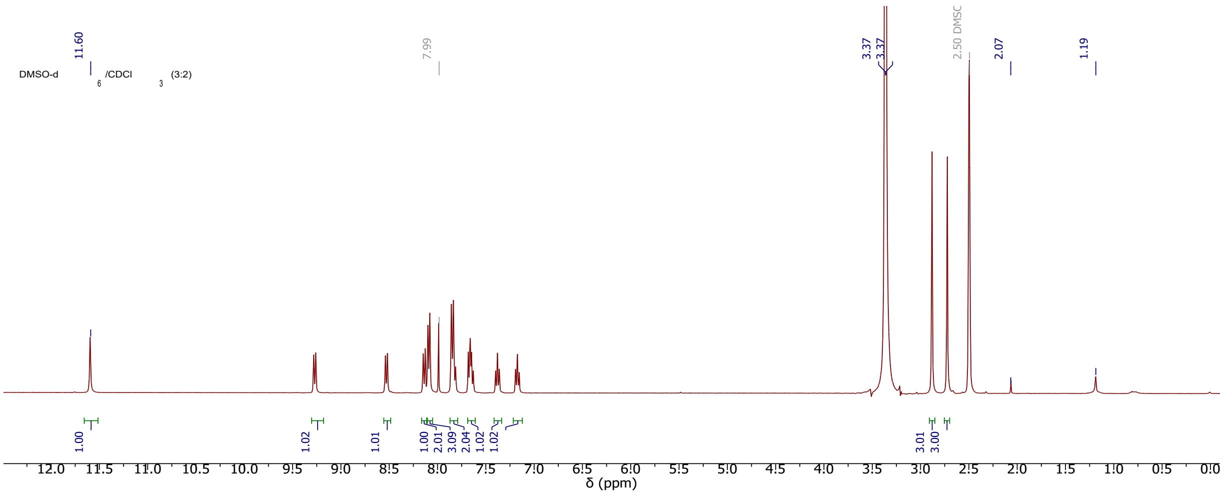
DMSO-d6/CDCl3 (3:2)
11.60
7.99
3.37
3.37
2.50 DMSO
2.07
1.19
1.00
1.02
1.01
1.00
2.01
3.09
2.04
1.02
1.02
3.01
3.00
δ (ppm)

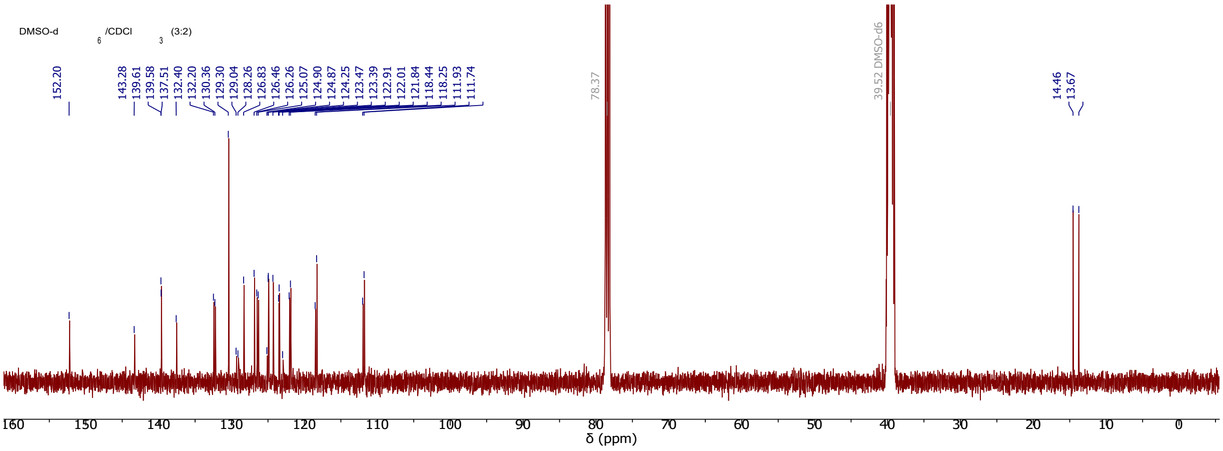
DMSO-d6/CDCl3 (3:2)
152.20
143.28
139.61
139.58
137.51
132.40
132.20
130.36
129.30
129.04
128.26
126.83
126.46
126.26
125.07
124.90
124.87
124.25
123.47
123.39
122.91
122.01
121.84
118.44
118.25
111.93
111.74
78.37
39.52 DMSO-d6
14.46
13.67
δ (ppm)

5-Mesityl-7,8-dimethyl-9*H*-indolo[3,2-*a*]phenanthridine (9k)

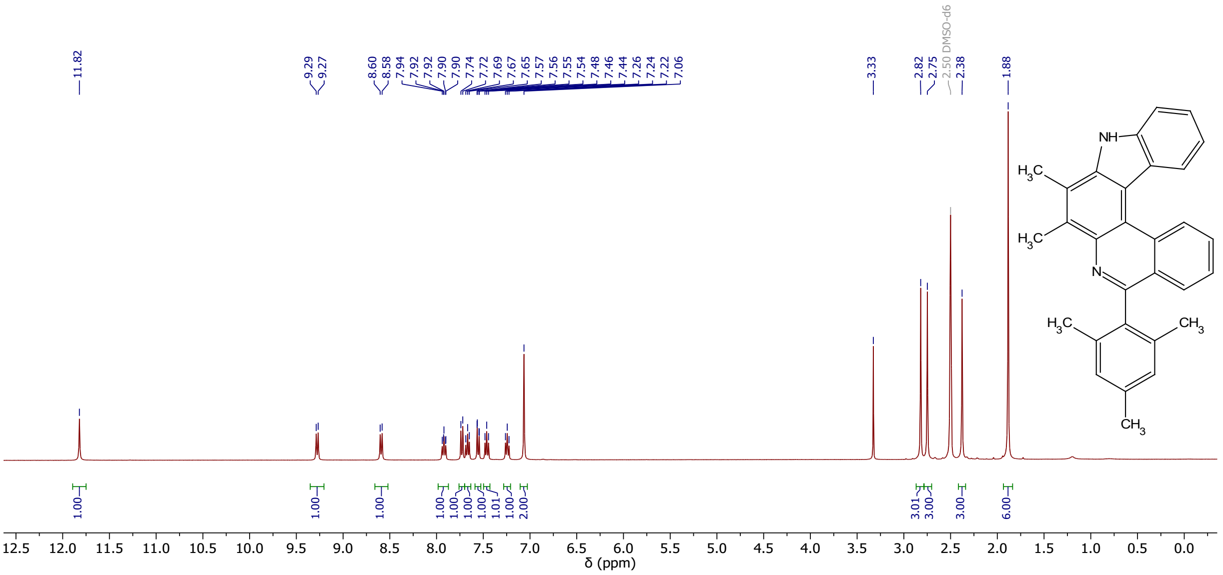

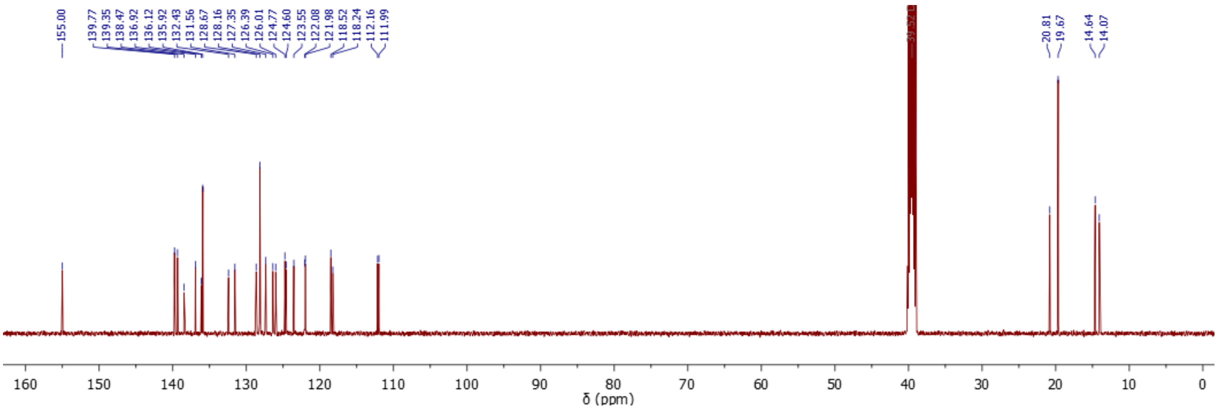

9*H*-Cinnolino[3,4-*c*]carbazole (4)

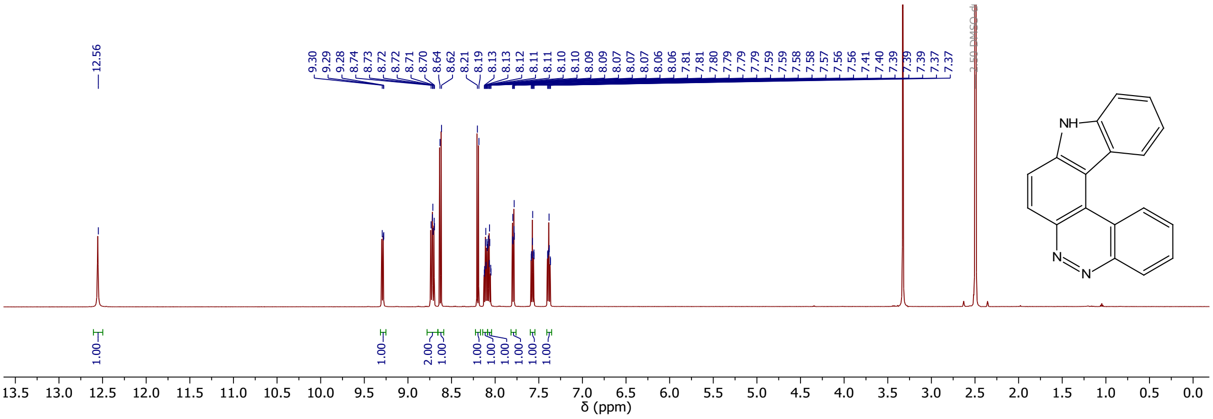

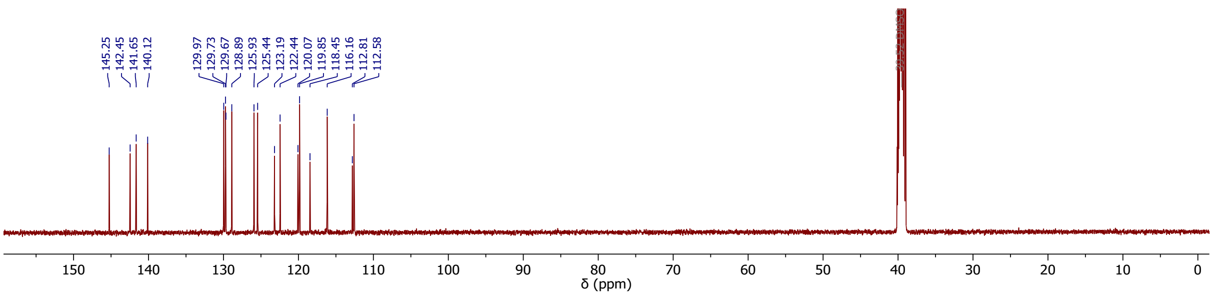

5-Methyl-6-phenyl-9*H*-indolo[2,3-*k*]phenanthridin-5-ium tetrafluoroborate (10)

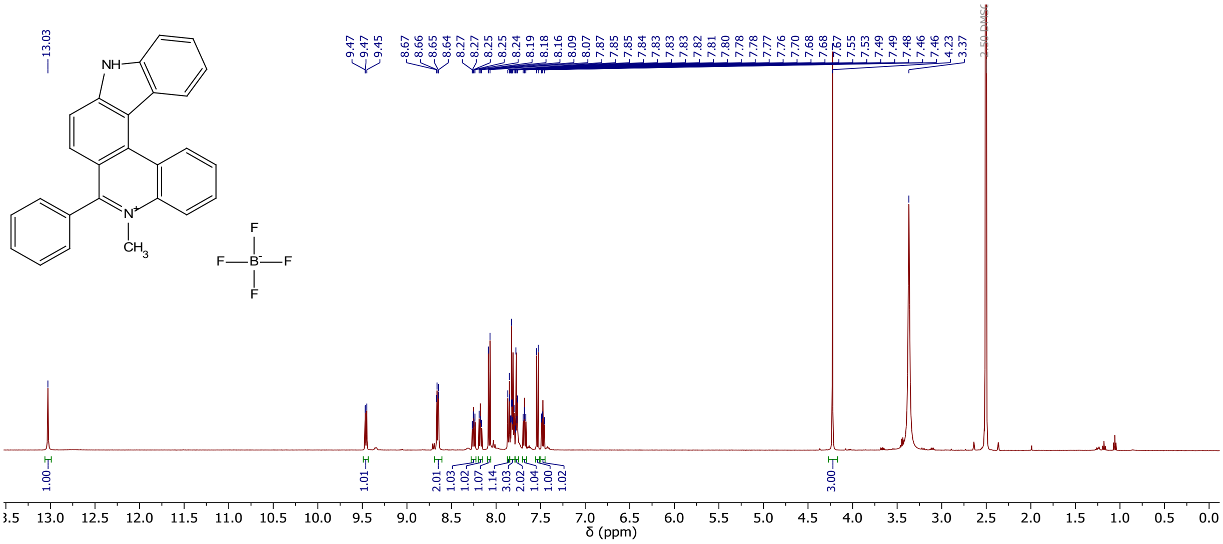

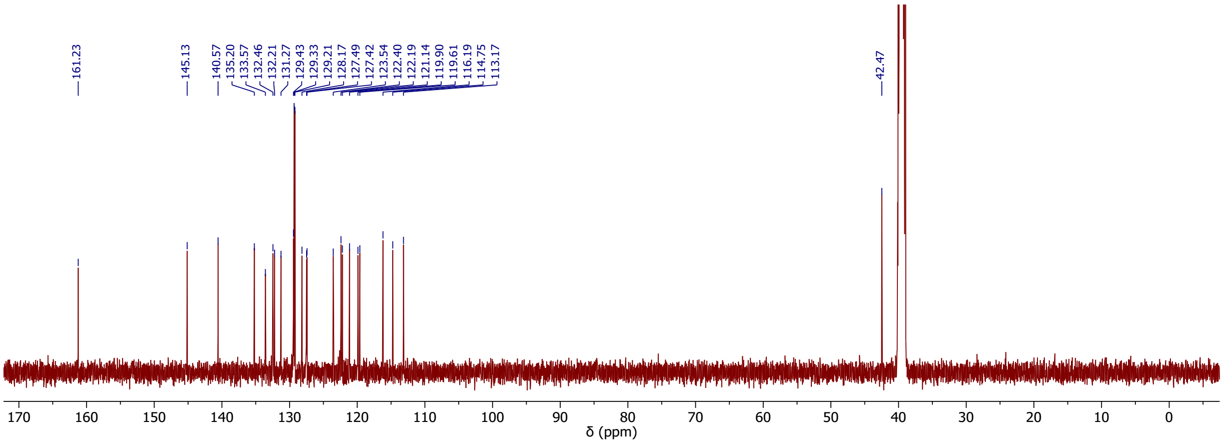

7,8-Dimethyl-5-((4-phenyl-1*H*-1,2,3-triazol-1-yl)methyl)-9*H*-indolo[3,2-*a*]phenanthridine (11)

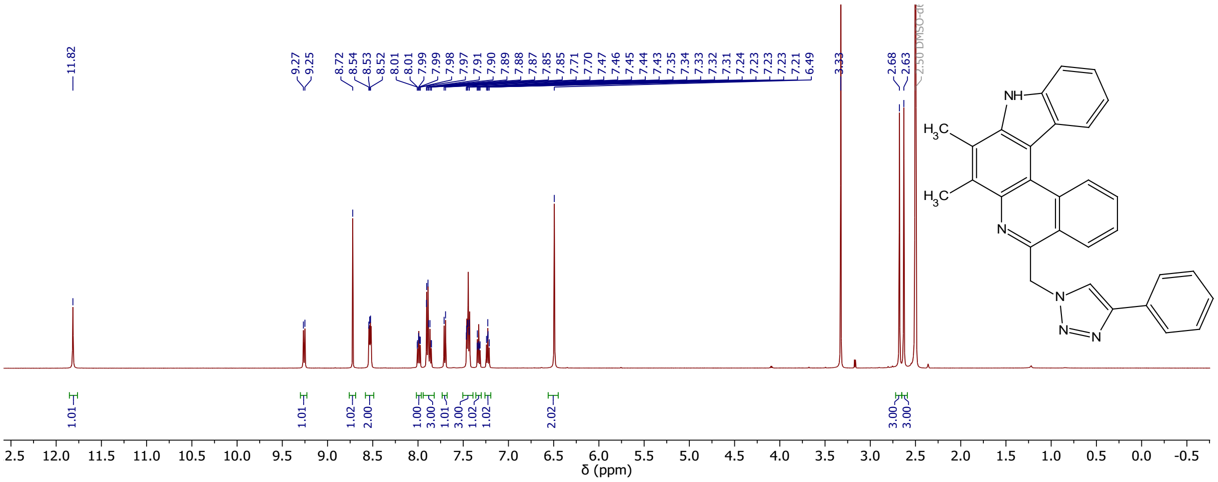

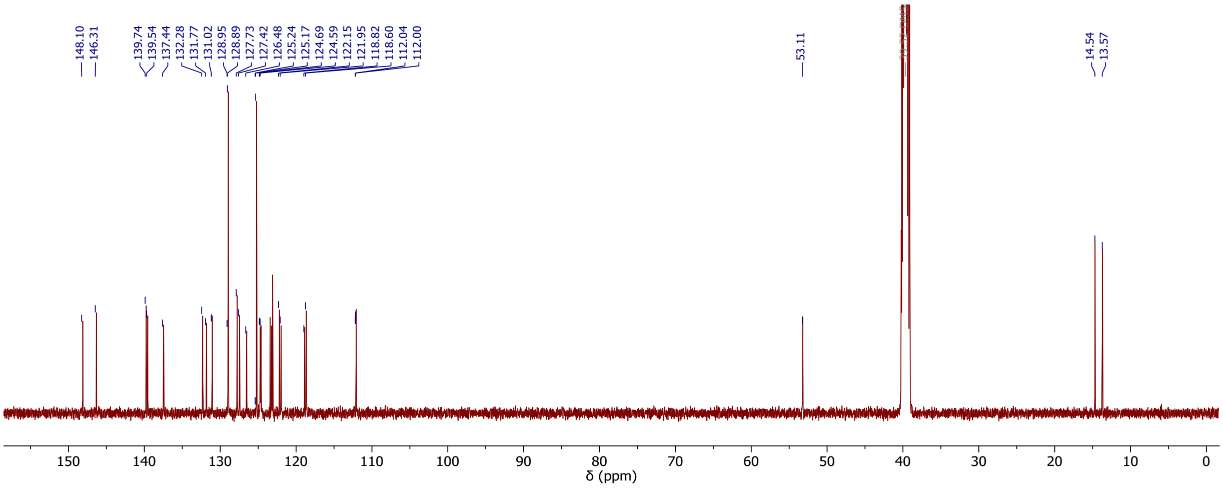

9-Butyl-6-phenyl-9*H*-indolo[2,3-*k*]phenanthridine (13)

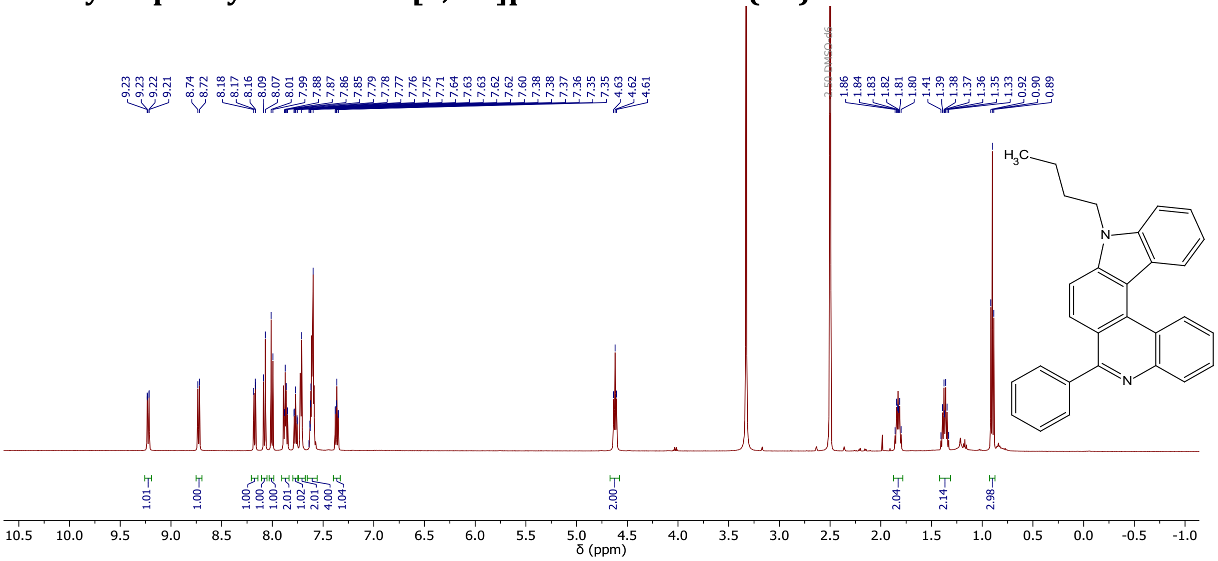

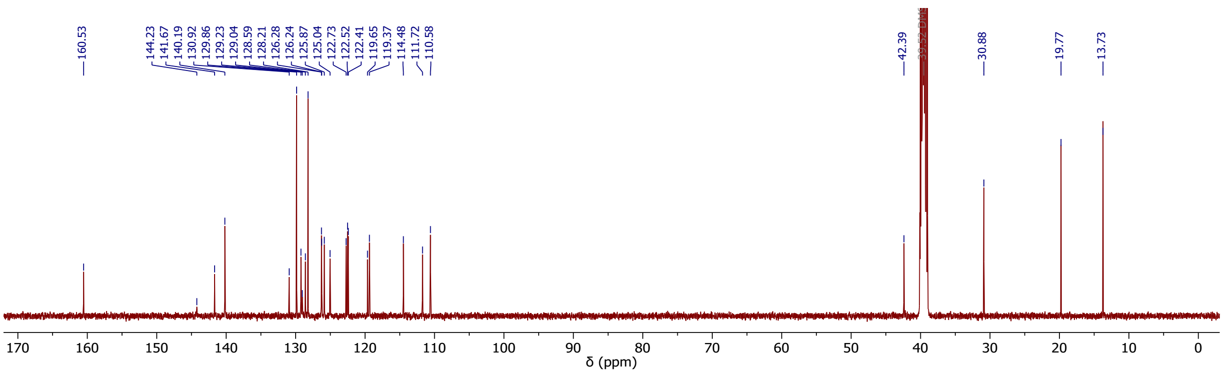

9-Hexyl-6-phenyl-9*H*-indolo[2,3-*k*]phenanthridine (14)

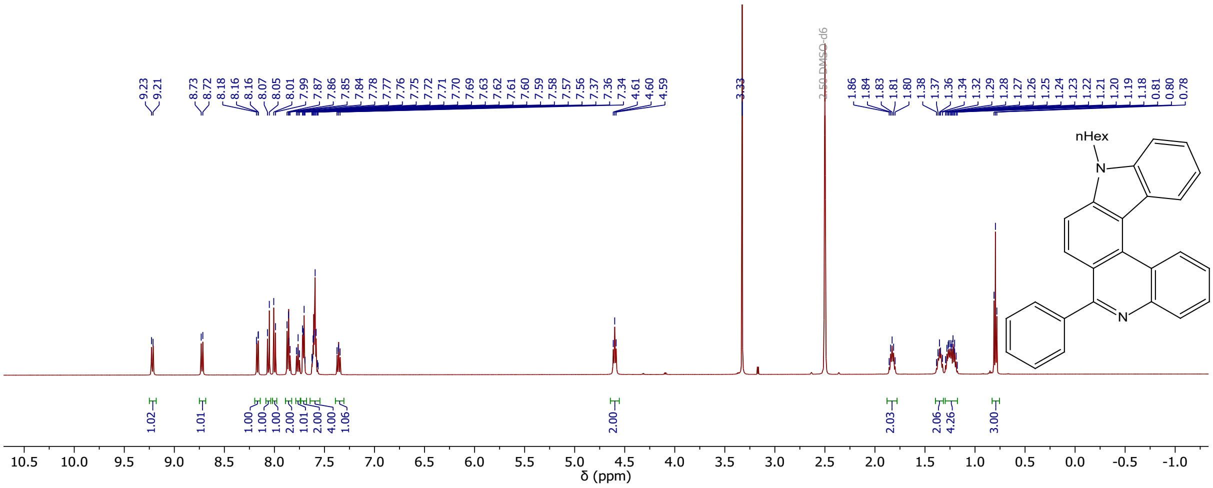

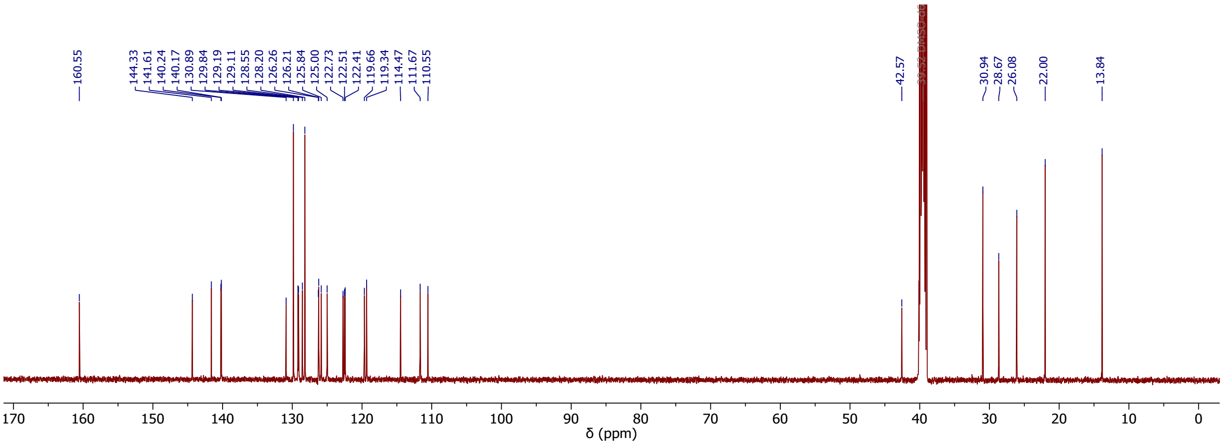

6,9-Diphenyl-9*H*-indolo[2,3-*k*]phenanthridine (12)

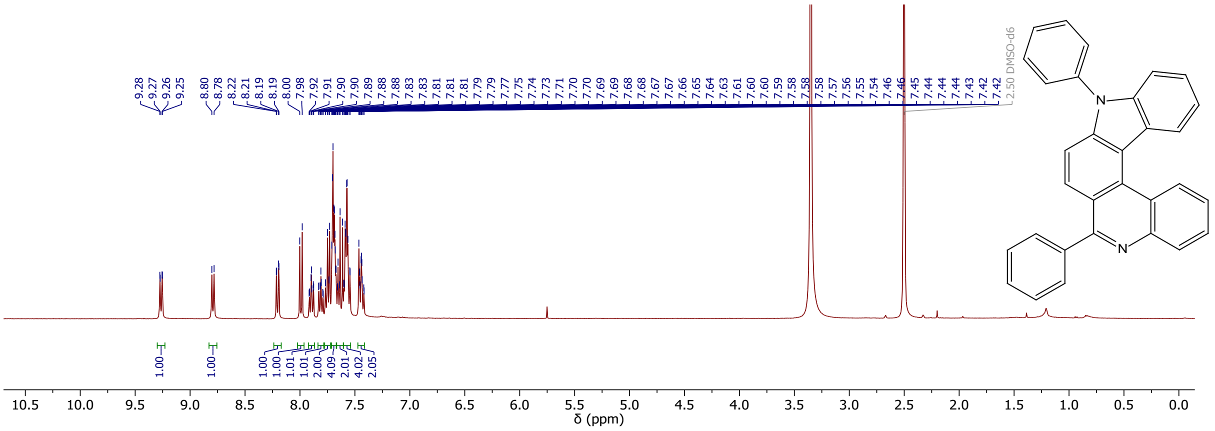

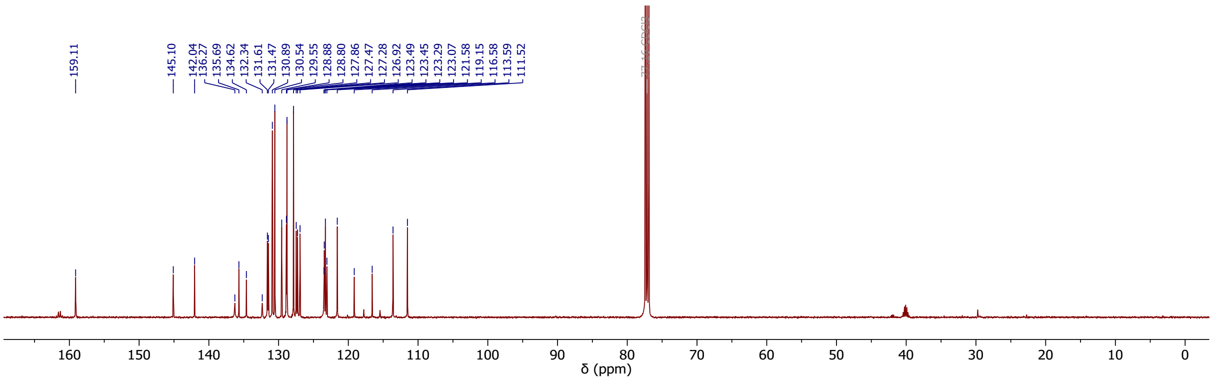

9-Hexyl-6-(pyridin-2-yl)-9*H*-indolo[2,3-*k*]phenanthridine (16)

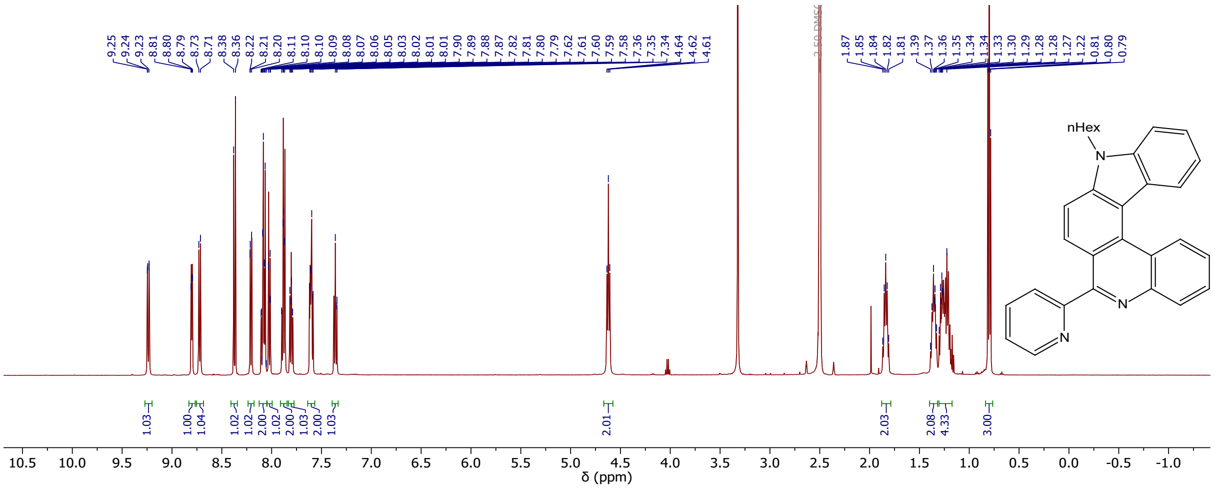

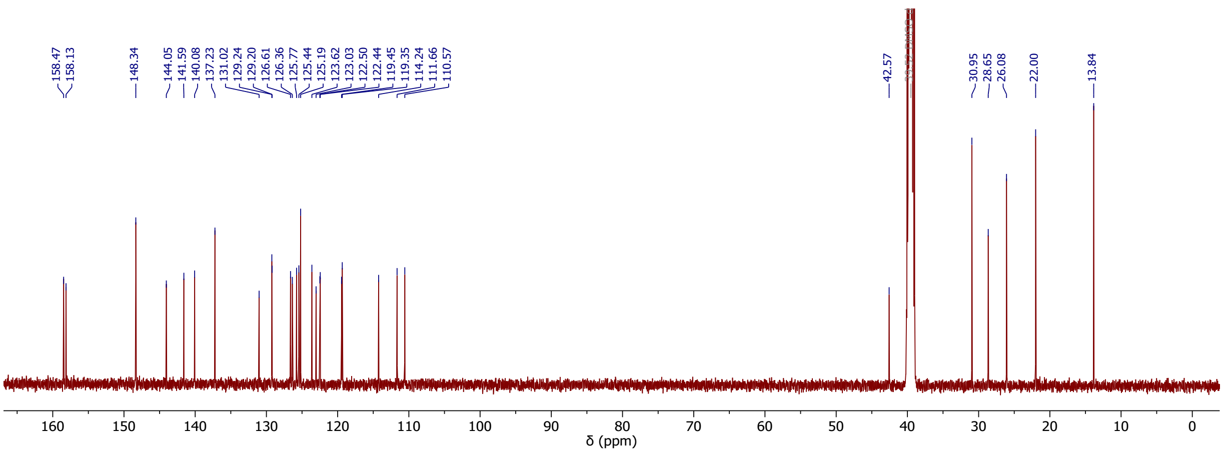

9-Hexyl-6-(naphthalen-1-yl)-9-indolo[2,3-*k*]phenanthridine (17)

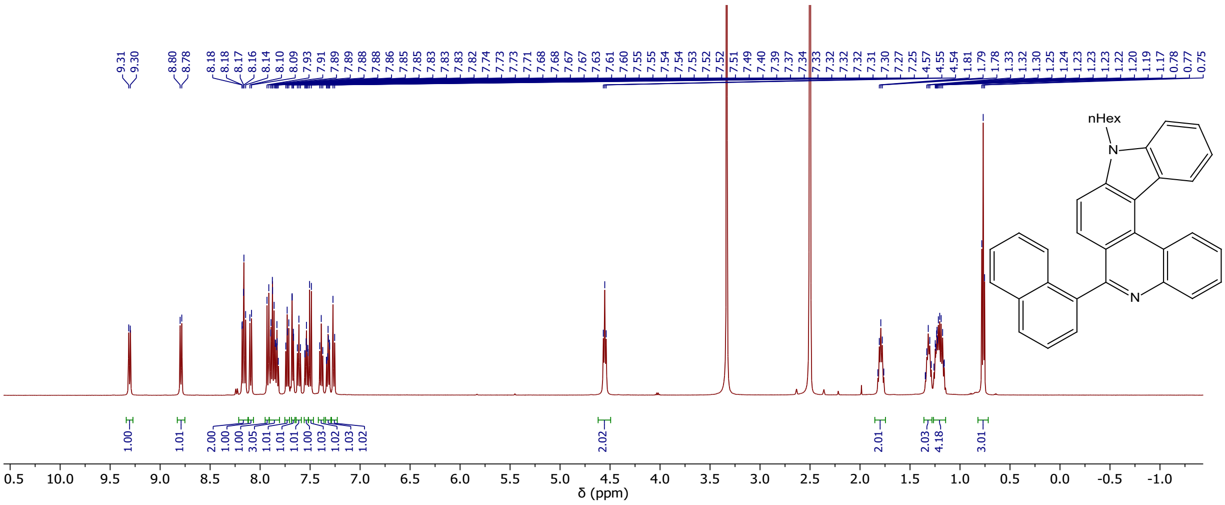

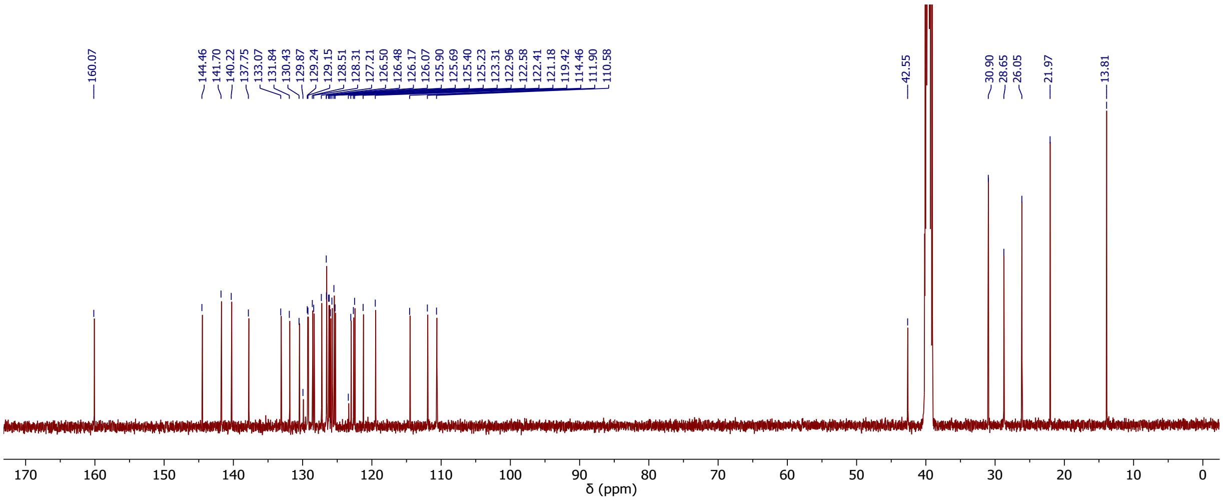

9-Hexyl-7,8-dimethyl-5-phenyl-9*H*-indolo[3,2-*a*]phenanthridine

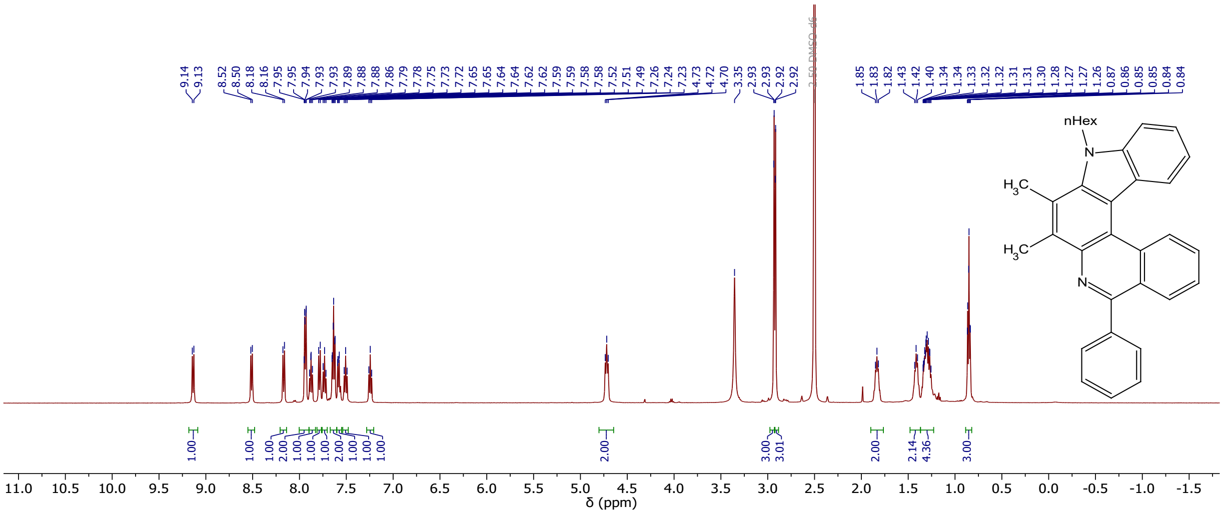

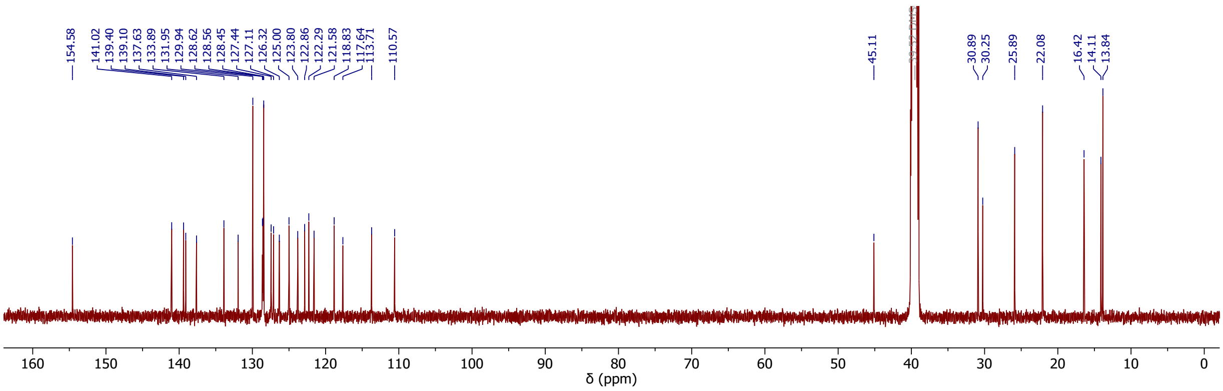

9-Hexyl-6-[2-(4,4,5,5-tetramethyl-1,3,2-dioxaborolan-2-yl)phenyl]-9*H*-indolo[2,3-*k*]phenanthridine (19)

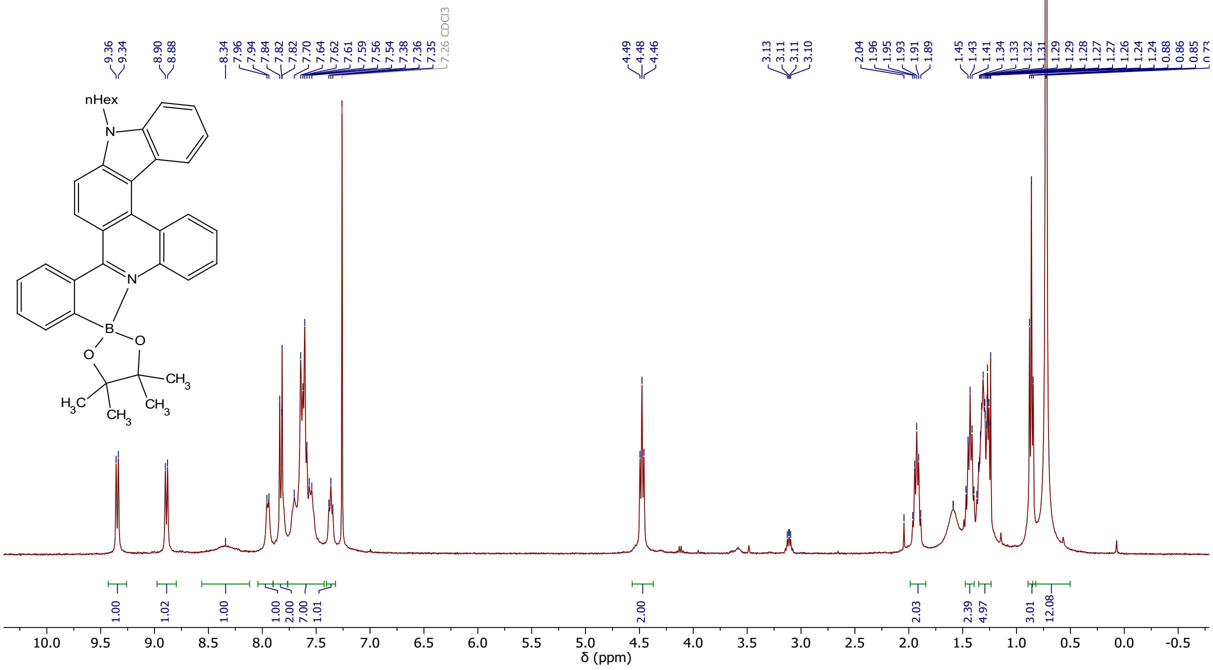

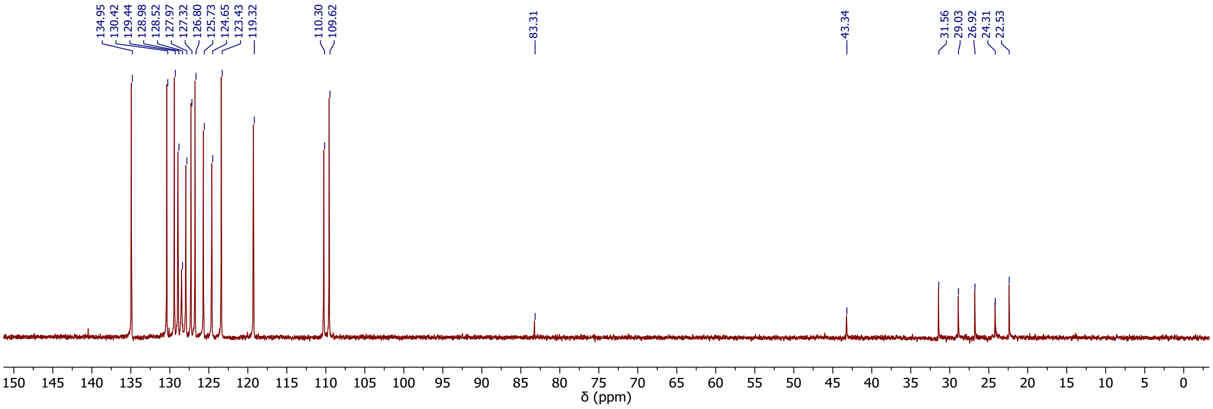

6-(2-(Dimethylboranyl)phenyl)-9-hexyl-9*H*-indolo[2,3-*k*]phenanthridine (20)

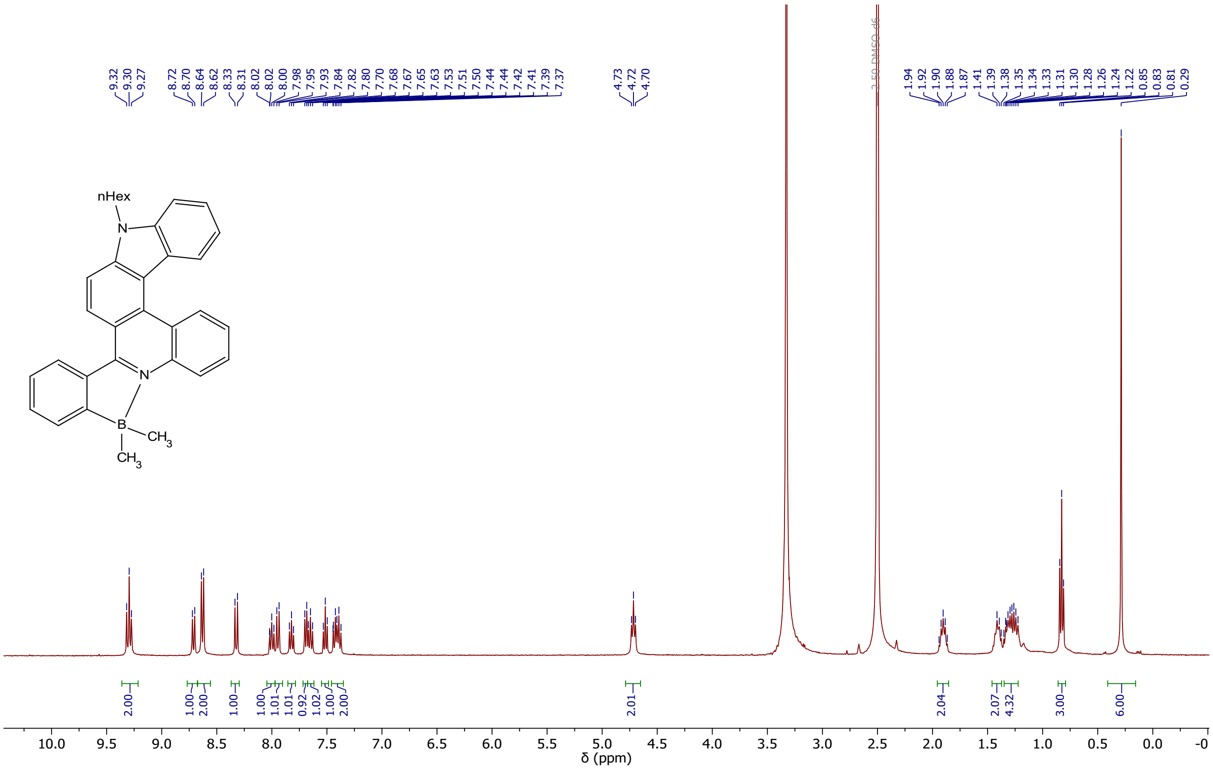

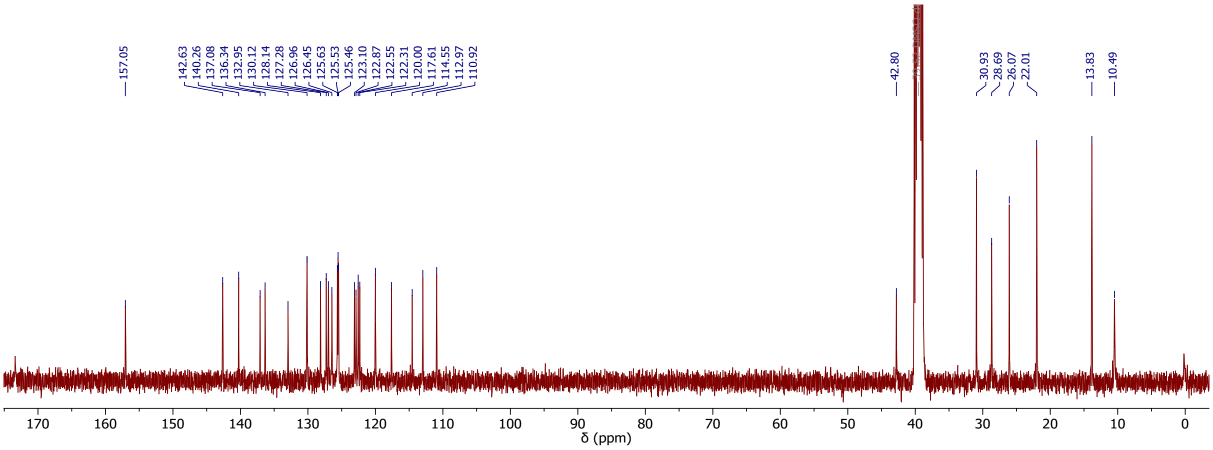

4-Bromo-2-(9*H*-carbazol-4-yl)aniline

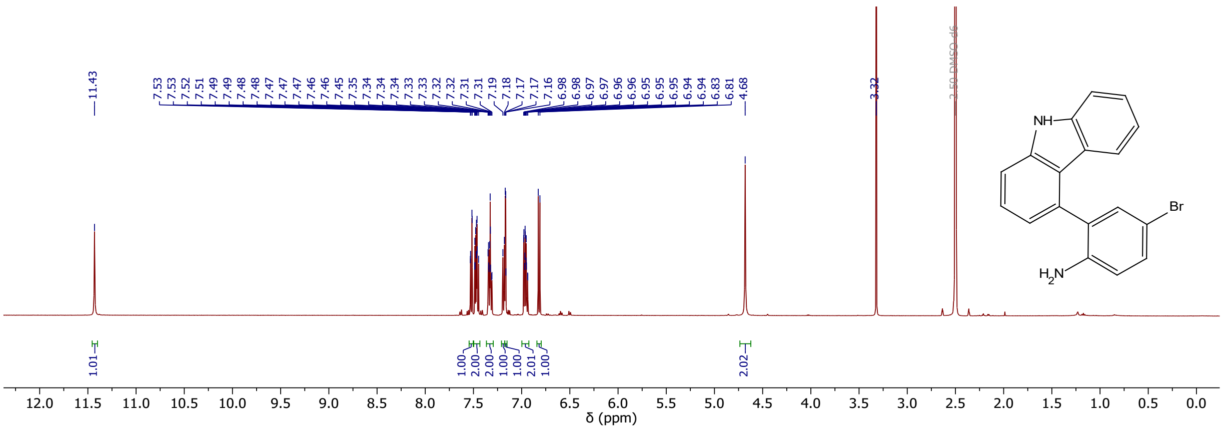

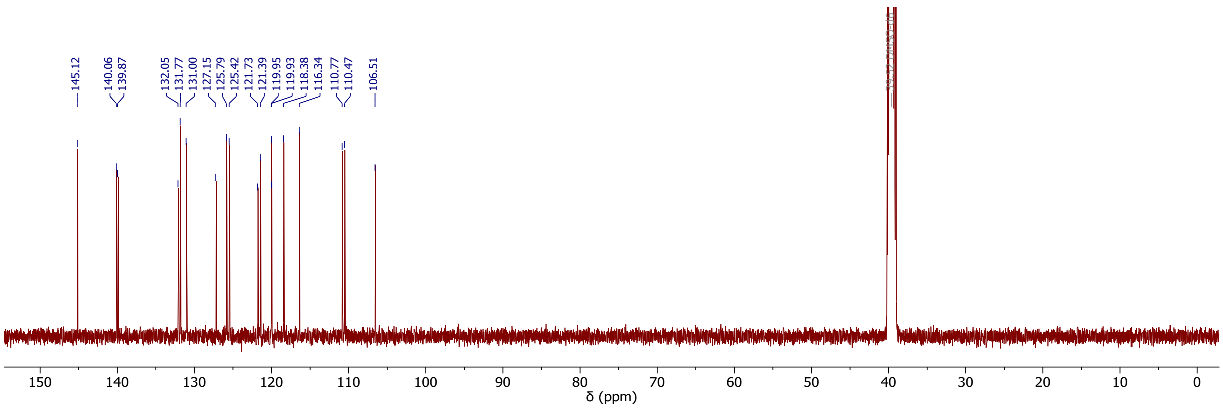

N-(4-Bromo-2-(9*H*-carbazol-4-yl)phenyl)benzamide

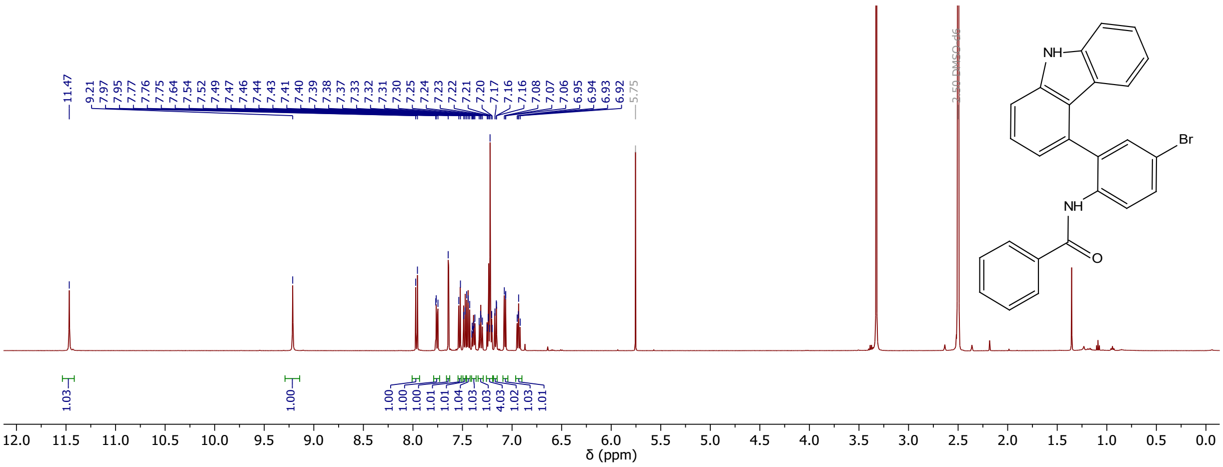

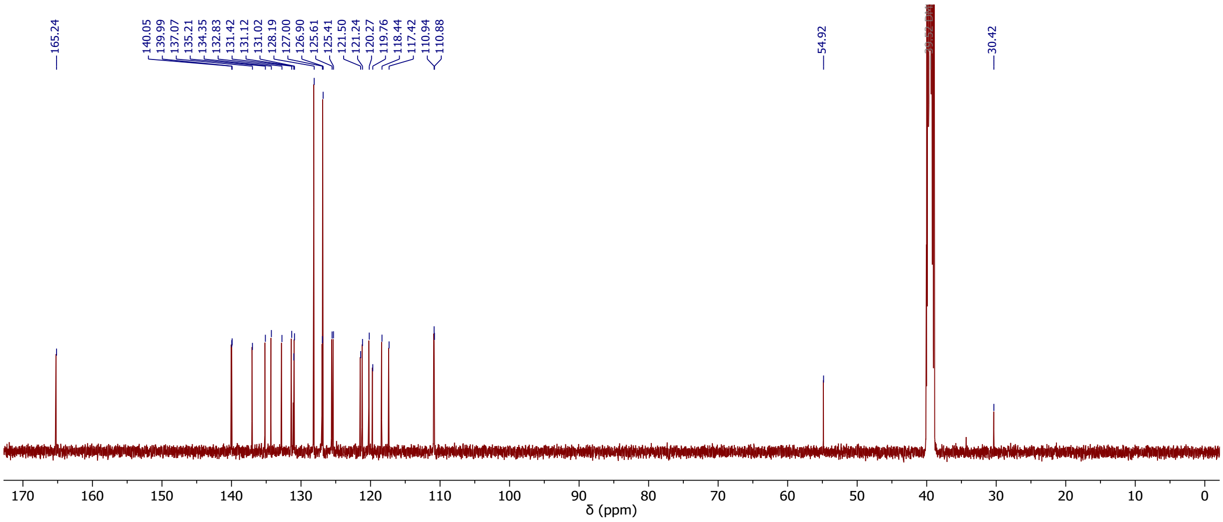

2-Bromo-6-phenyl-9*H*-indolo[2,3-*k*]phenanthridine (6)

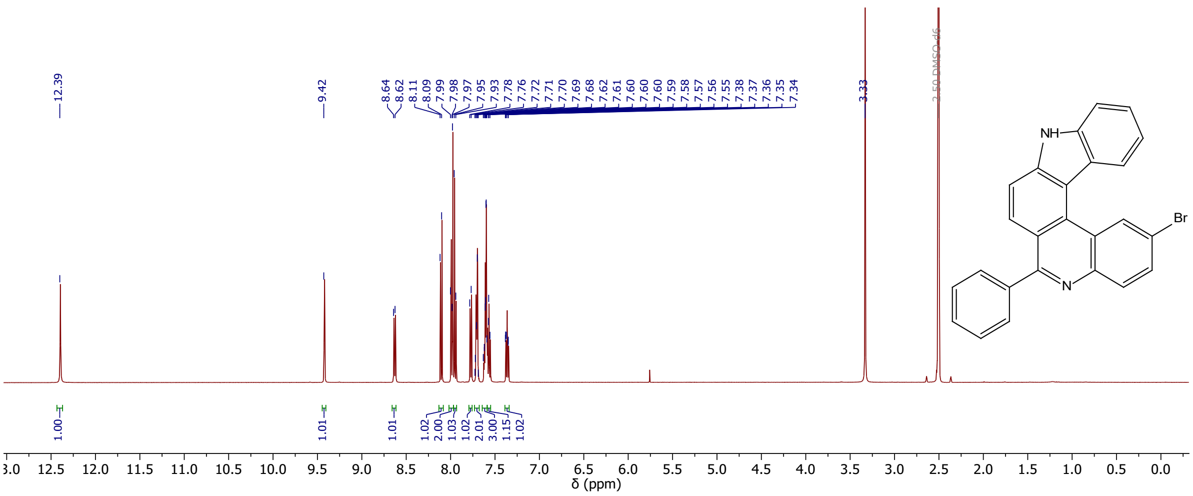

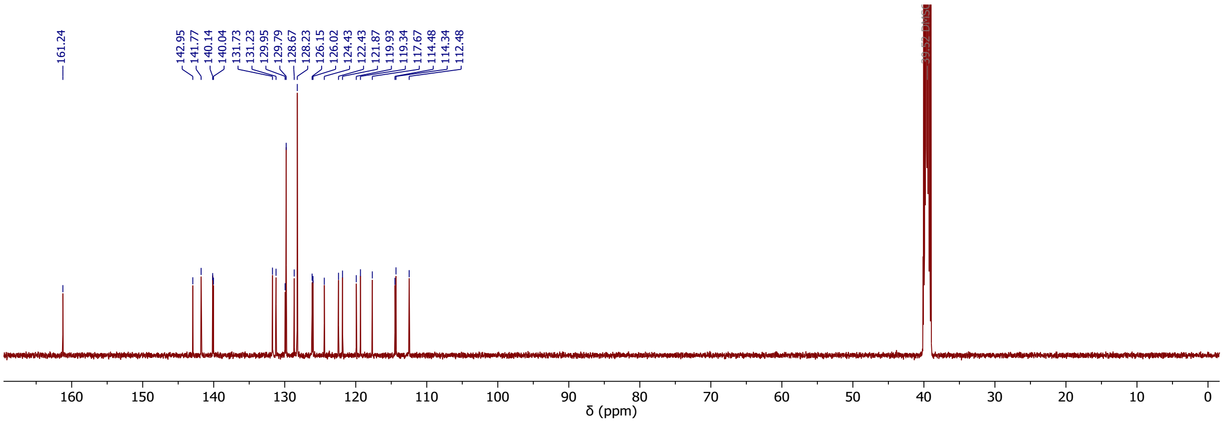

2-Bromo-9-hexyl-6-phenyl-9*H*-indolo[2,3-*k*]phenanthridine (15)

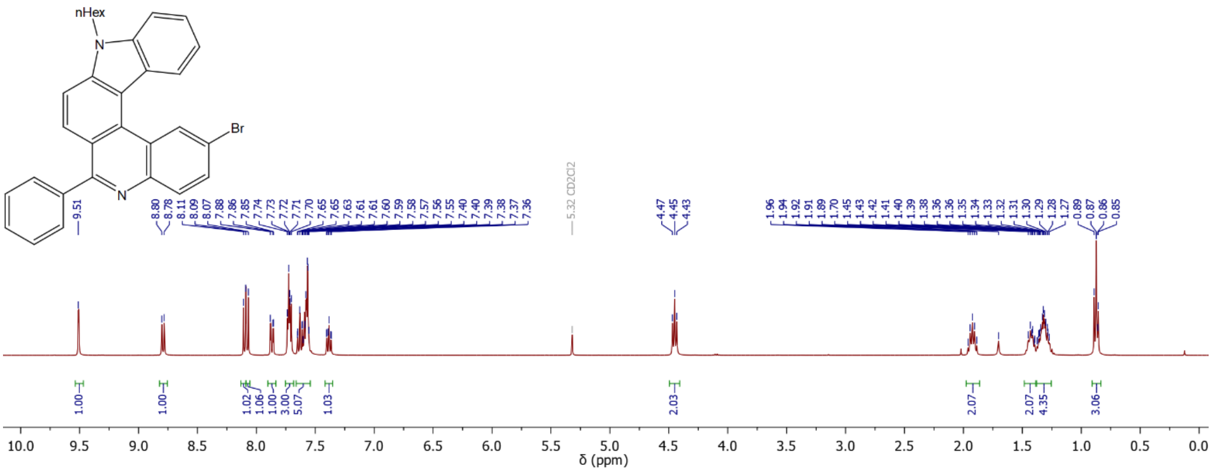

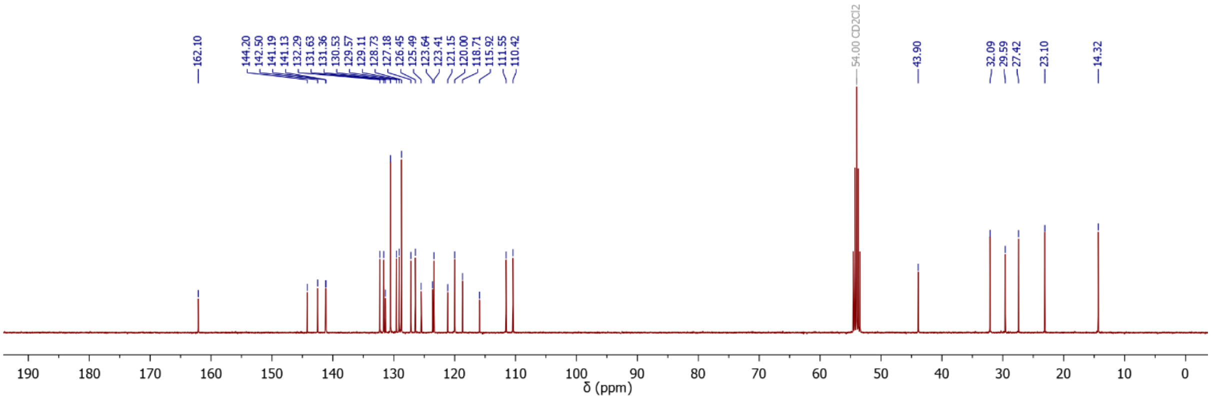

2-Azido-9-hexyl-6-phenyl-9*H*-indolo[2,3-*k*]phenanthridine (23)

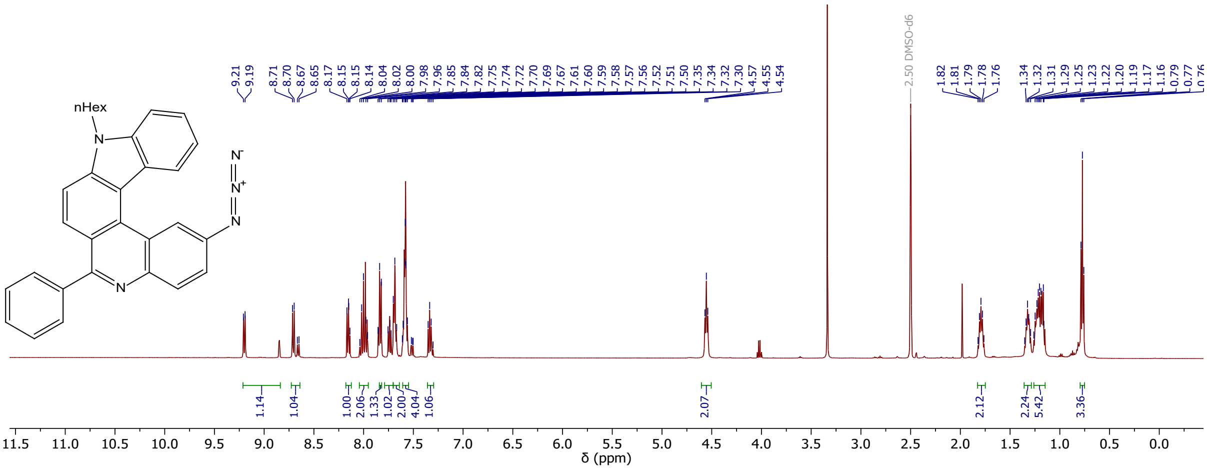

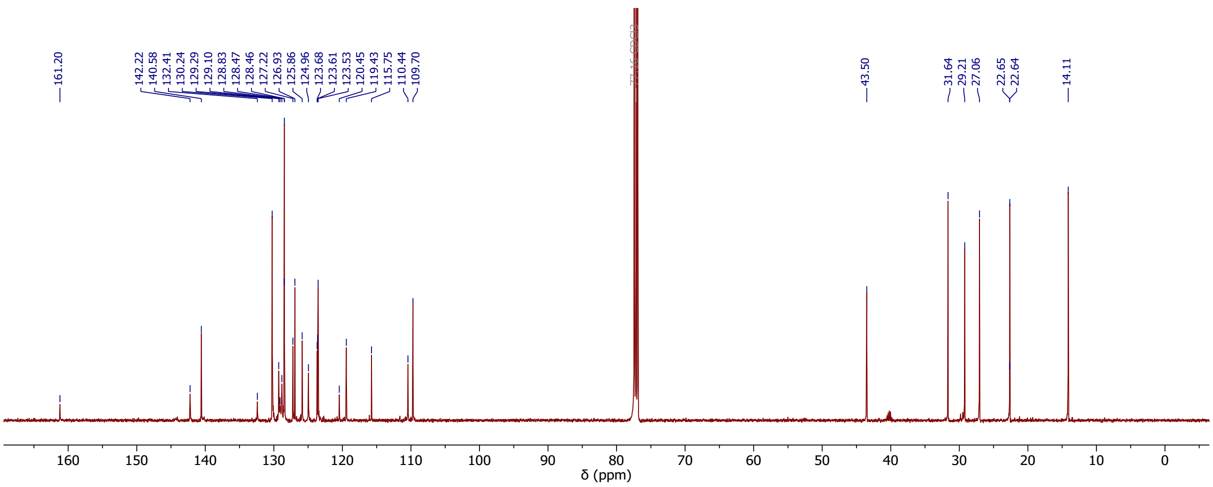

9-Hexyl-6-phenyl-2-((3-(1,2,2-triphenylvinyl)phenyl)ethynyl)-9*H*-indolo[2,3-*k*]phenanthridine (22)

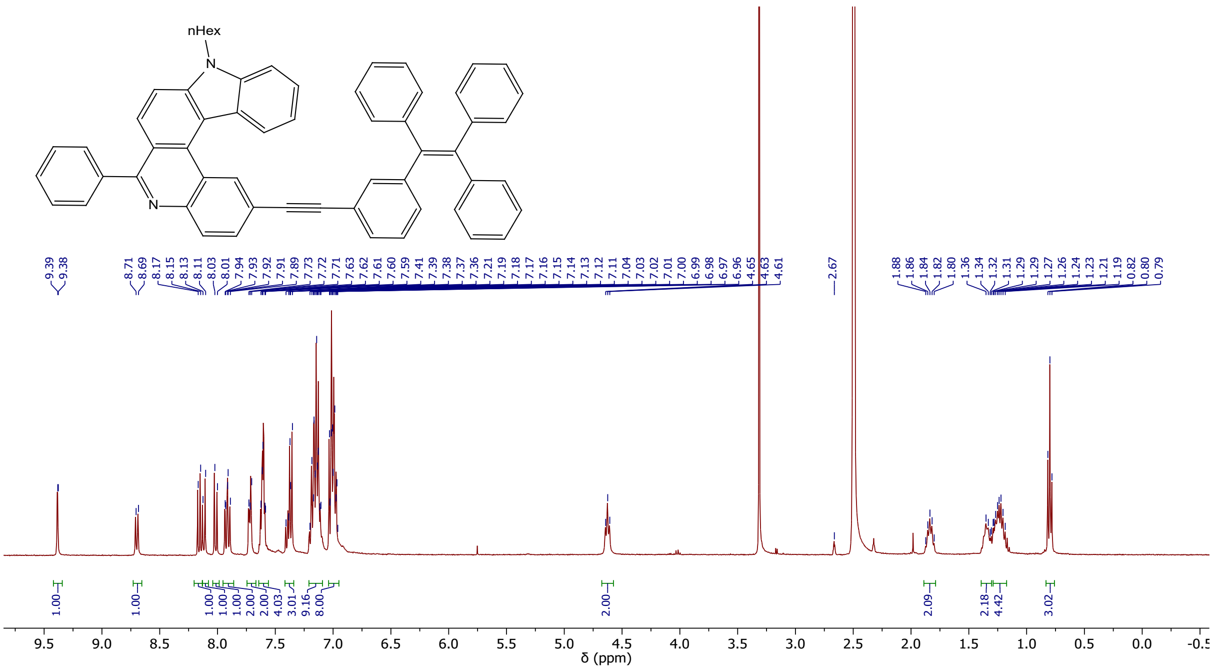

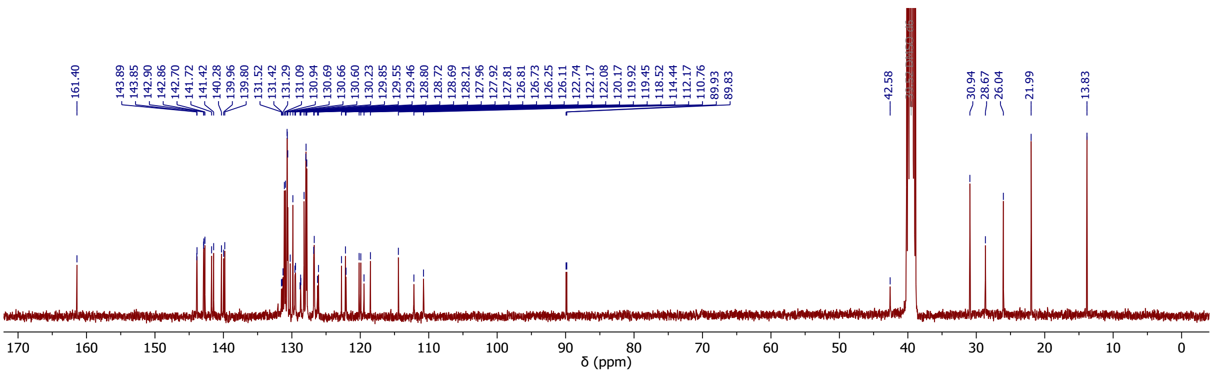

9-Hexyl-6-phenyl-2-(4-phenyl-1*H*-1,2,3-triazol-1-yl)-9*H*-indolo[2,3-*k*]phenanthridine (24)

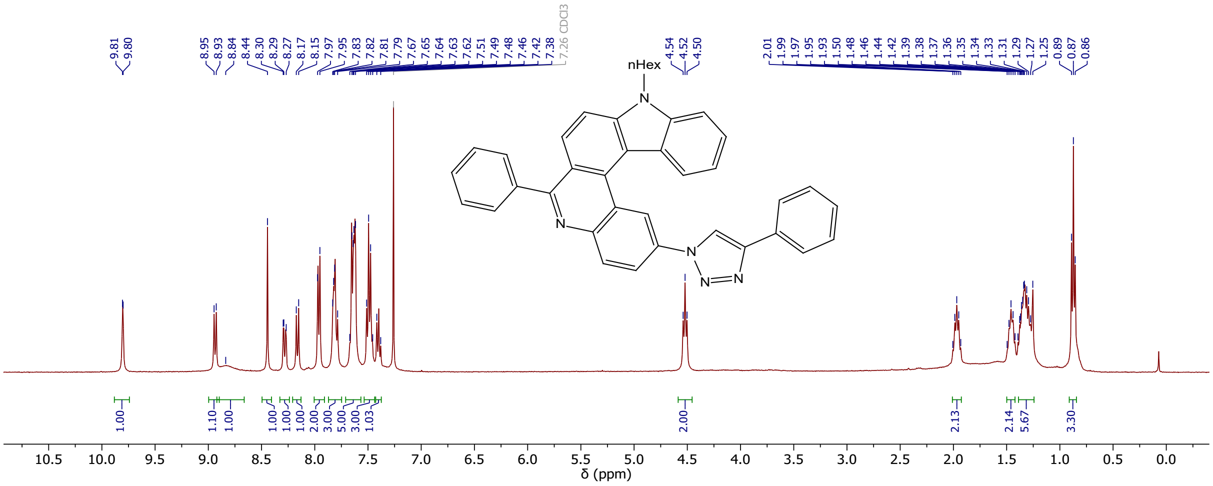

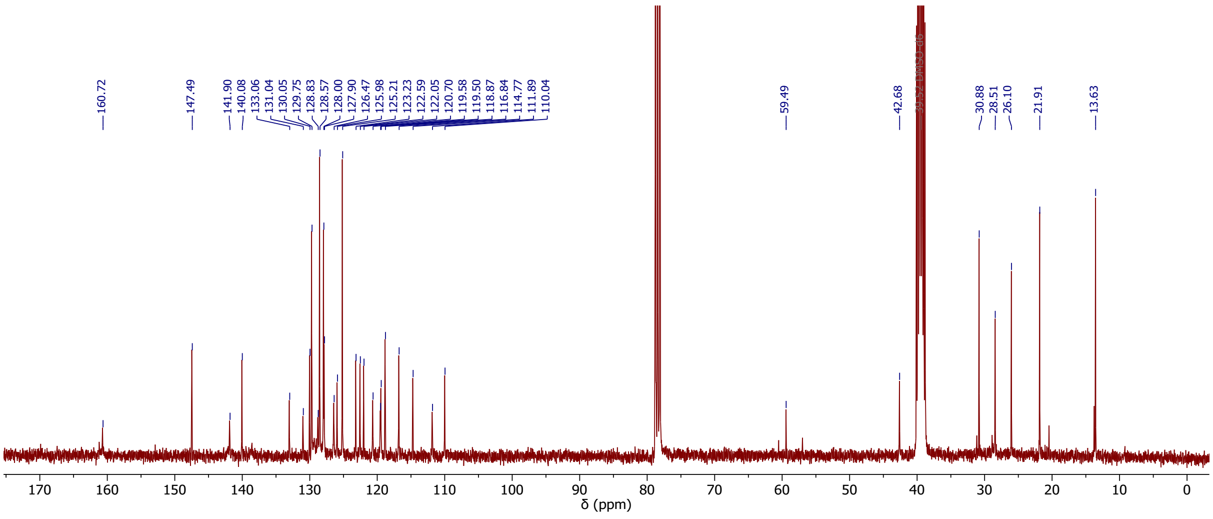

9-Hexyl-6-phenyl-2-(4-(4-(1,2,2-triphenylvinyl)phenyl)-1*H*-1,2,3-triazol-1-yl)-9*H*-indolo[2,3-*k*]phenanthridin (25)

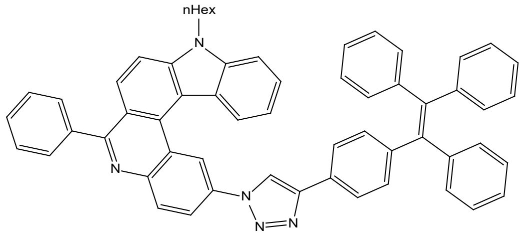

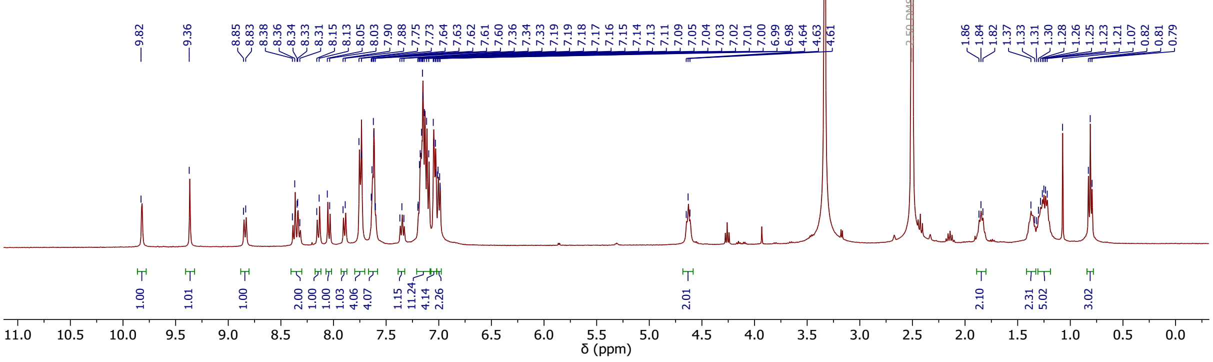

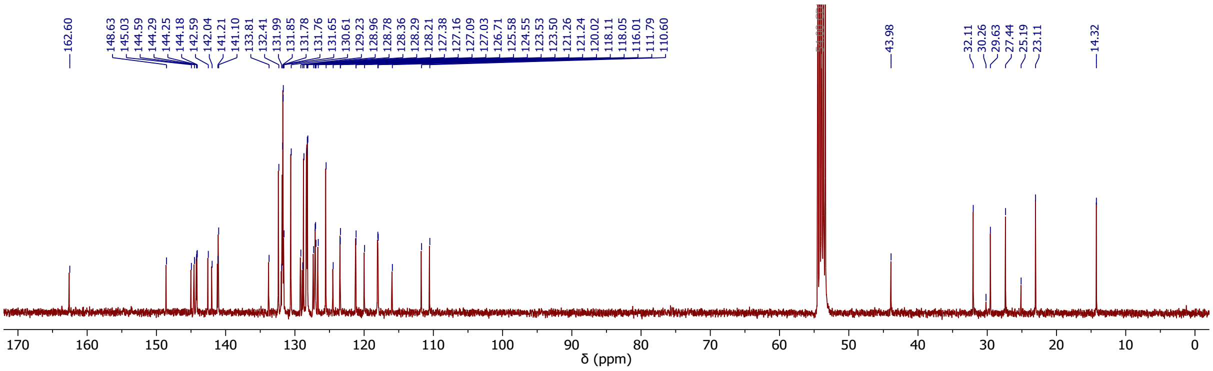

7.2 Computational Data

7.2.1 Indolocarbazole (ICz, 5)

5 [(*M*)-enantiomer]

1\1\GINC-N0330\FOpt\RPBE1PBE\def2TZVP\C18H12N2\KA_PT6974\15-Jul-2024\0
\\# opt=tight pbe1pbe scrf=(solvent=dichloromethane) def2tzvp empirica
ldispersion=gd3bj\\Title Card Required\\0,1\H,-0.4085066864,-0.5164625
862,0.1027177197\H,0.0506313236,0.2403458153,5.0074310723\H,1.03675035
45,-0.0212034349,6.5117667456\H,1.4250577034,0.2779188576,8.9025238342
\H,4.3831207906,-2.8362260693,8.9028398198\H,1.5934715802,-2.358238616
8,0.5536081356\H,-1.5924751385,1.4935588305,3.7091104814\C,3.650997621
6,-2.2300875528,8.3816709199\C,2.9182861157,-1.2635534879,9.0435558612
\C,1.9815551733,-0.4788406201,8.3623959202\C,1.7565104617,-0.653277445
8,7.0095829497\C,2.5159291485,-2.0795076502,4.9341994363\C,-0.01938782
96,0.0686161321,3.9439465534\C,-0.9551190973,0.7733898326,3.2098644208
\C,-1.0923816933,0.5690695655,1.8326818309\C,-0.301764967,-0.348932787
3,1.1683469038\C,0.6357142859,-1.0573899468,1.9127314261\C,0.805662948
,-0.8626336694,3.3083741771\C,1.855095101,-1.763591223,3.726238411\C,2
.2524823917,-2.463743764,2.5640641661\C,3.2379150134,-3.4522494161,2.5
489097314\C,3.8680949096,-3.7743386894,3.7304323676\C,3.5024069481,-3.
091349197,4.8915733381\C,3.4225852424,-2.3988345122,7.0199664168\N,1.5
128809892,-2.0159067447,1.494310276\H,4.6348702337,-4.5393011373,3.758
8717312\H,3.4979943522,-3.9576282409,1.6264826121\C,2.470469736,-1.625
959498,6.3052834091\N,4.0216608282,-3.2665963222,6.1531618859\H,4.7457
729949,-3.9197469963,6.3932914138\H,-1.8323491104,1.1351557251,1.27869
70024\H,3.0756214604,-1.108842004,10.1047884603\\Version=ES64L-G16RevC
.01\State=1-A\HF=-802.0905851\RMSD=2.077e-09\RMSF=7.901e-07\Dipole=0.9
15187,-1.1321731,-0.7967651\Quadrupole=-3.8074135,-4.5233169,8.3307304
,-11.4429389,4.5848719,2.3172204\PG=C01 [X(C18H12N2)]\\@

5 [transition state]

1\1\GINC-N1305\SP\RPBE1PBE\def2TZVP\C18H12N2\KA_PT6974\28-Oct-2024\0\\
# sp scrf=(solvent=dichloromethane) def2tzvp pbe1pbe empiricaldispersi
on=gd3bj\\Title Card Required\\0,1\H,0,5.143138,0.164091,0.000011\H,0,
0.906342,-2.460238,-0.000007\H,0,-0.906343,-2.460238,-0.00001\H,0,-3.0
65753,-3.595199,-0.000004\H,0,-5.143138,0.164092,0.000011\H,0,3.408047
,2.30607,-0.000007\H,0,3.065752,-3.5952,-0.000003\C,0,-4.220043,-0.404
173,0.000007\C,0,-4.228402,-1.785803,0.000005\C,0,-3.032754,-2.512138,
-0.000002\C,0,-1.809214,-1.868661,-0.000004\C,0,-0.706478,0.519845,-0.
000004\C,0,1.809213,-1.868661,-0.000004\C,0,3.032753,-2.512138,-0.0000
02\C,0,4.228401,-1.785804,0.000005\C,0,4.220043,-0.404174,0.000006\C,0
,2.985961,0.237632,0.000002\C,0,1.756934,-0.472522,-0.000002\C,0,0.706
478,0.519845,-0.000004\C,0,1.357465,1.774965,-0.000002\C,0,0.688566,3.
000084,0.\C,0,-0.688566,3.000084,0.\C,0,-1.357464,1.774965,-0.000002\C
,0,-2.985961,0.237632,0.000002\N,0,2.718371,1.576034,0.000001\H,0,-1.2
42868,3.93103,0.000004\H,0,1.242868,3.931031,0.000003\C,0,-1.756935,-0
.472522,-0.000002\N,0,-2.71837,1.576034,0.000001\H,0,-3.408046,2.30607
1,-0.000008\H,0,5.175282,-2.313362,0.000008\H,0,-5.175283,-2.313362,0.
000008\\Version=ES64L-G16RevC.01\State=1-A\HF=-802.0905632\RMSD=5.830e
-09\Dipole=0.0000002,1.658116,-0.0000066\Quadrupole=10.1828988,6.40274
81,-16.5856469,0.0000085,0.0000101,-0.0000187\PG=C01 [X(C18H12N2)]\\@

5 [S_1]

1\1\GINC-N1212\FOpt\RPBE1PBE TDA-FC\def2TZVP\C18H12N2\KA_PT6974\28-Oct
-2024\0\\# opt def2tzvp pbe1pbe TDA=(singlet,root=1,nstates=50) scrf=(
cpcm,solvent=dichloromethane) empiricaldispersion=gd3bj\\Title Card Re
quired\\0,1\H,-5.1272,0.201262,-0.074748\H,-0.923826,-2.483208,0.15978
9\H,0.924167,-2.48304,-0.164354\H,3.105641,-3.59147,-0.142494\H,5.1270
56,0.201321,0.080033\H,-3.376597,2.307271,-0.089004\H,-3.105317,-3.591
647,0.141468\C,4.215361,-0.382676,0.038775\C,4.247352,-1.788399,-0.019

051\C,3.066069,-2.509365,-0.087019\C,1.816947,-1.880789,-0.089519\C,0.
701587,0.496664,0.001761\C,-1.816749,-1.880921,0.086995\C,-3.065866,-2
.509509,0.086567\C,-4.247298,-1.788495,0.021751\C,-4.215422,-0.382755,
-0.035662\C,-2.980105,0.223654,-0.034225\C,-1.750634,-0.485138,0.01361
1\C,-0.701605,0.496632,-0.004048\C,-1.349282,1.787814,-0.035214\C,-0.6
97488,3.022132,-0.023674\C,0.697341,3.022148,0.023343\C,1.349185,1.787
851,0.034235\C,2.980052,0.223738,0.034809\N,-2.685471,1.574623,-0.0646
37\H,1.263871,3.944209,0.043616\H,-1.264067,3.944184,-0.042941\C,1.750
672,-0.485061,-0.015215\N,2.685344,1.574691,0.065147\H,3.3764,2.307347
,0.091174\H,-5.200057,-2.303333,0.022486\H,5.200109,-2.30324,-0.017915
\\Version=ES64L-G16RevC.01\State=1-A\HF=-802.0863579\RMSD=7.331e-09\RM
SF=6.996e-06\Dipole=-0.0001352,2.0737318,0.0022094\PG=C01 [X(C18H12N2)
]\\@

5 [T_1]

1\1\GINC-N1038\FOpt\RPBE1PBE TDA-FC\def2TZVP\C18H12N2\KA_PT6974\13-Nov
-2024\0\\# opt def2tzvp pbe1pbe TDA=(triplets,nstates=10) scrf=(cpcm,s
olvent=dichloromethane) empiricaldispersion=gd3bj\\Title Card Required
\\0,1\H,0.0484847486,-0.0076473901,-0.011554225\H,-0.0529434828,-0.004
4098801,4.9774315032\H,0.9674945077,0.0214667579,6.5631007059\H,1.1884
314758,0.0244599085,8.9988664068\H,5.3114017563,-1.1691740613,8.710922
8025\H,2.685745257,-0.6455022671,0.3776128644\H,-2.0793182188,0.477865
1946,3.69563921\C,4.3655728557,-0.9184497697,8.2451008775\C,3.23965744
14,-0.5730929449,9.0167229819\C,2.0466653499,-0.2470880425,8.394295591
5\C,1.9137336392,-0.2553331037,7.0033717013\C,3.2693149245,-0.72416248
29,4.8058798383\C,-0.0006821917,0.0042990402,3.8989984424\C,-1.1578299
563,0.2713243252,3.1627136786\C,-1.1584475617,0.2770660971,1.778195509
9\C,0.0279067826,0.0011795611,1.0719326394\C,1.1577371498,-0.260582559
4,1.806616084\C,1.1983204837,-0.2632912372,3.2261799348\C,2.5419288534
,-0.5745092729,3.6049254965\C,3.2803312179,-0.7719512795,2.3750439642\
C,4.6321843064,-1.1198941817,2.2806461314\C,5.3436469385,-1.2973964124
,3.4683928703\C,4.6617614165,-1.0989842822,4.6738580226\C,4.2225233688
,-0.921936824,6.8796993376\N,2.4313852057,-0.5691747527,1.3492165413\H
,6.389797489,-1.5734290832,3.469626412\H,5.0982163267,-1.2500234246,1.
3129131269\C,3.0122047463,-0.6050017775,6.2077651873\N,5.1760816565,-1
.2117095562,5.9135827245\H,6.1318182546,-1.4603608907,6.1117714416\H,-
2.0699389436,0.4881623769,1.2330310268\H,3.3140417667,-0.5592043323,10
.0969343226\\Version=ES64L-G16RevC.01\State=1-A\HF=-802.0850023\RMSD=8
.929e-09\RMSF=1.705e-05\Dipole=1.6637371,-0.4468576,-1.0633585\PG=C01
[X(C18H12N2)]\\@

5·H^+ [protonated]

1\1\GINC-N0301\FOpt\RPBE1PBE\def2TZVP\C18H13N2(1+)\KA_PT6974\14-Oct-20
24\0\\# opt=tight scrf=(cpcm,solvent=dichloromethane) def2tzvp pbe1pbe
\\Title Card Required\\1,1\H,-5.1397372479,0.1344961247,-0.0691886885\
H,-0.8809854636,-2.4347493741,0.162517356\H,0.9054091503,-2.4736459477
,-0.1753645109\H,3.062651919,-3.5990229168,-0.1655763019\H,5.130992014
,0.1568021267,0.0841339593\H,-3.2231662248,2.0998481959,0.7096868808\H
,-3.0284326103,-3.5955252087,0.1657456448\C,4.2104838768,-0.4130708941
,0.0394170984\C,4.2202358338,-1.7918707447,-0.0273737003\C,3.026613851
4,-2.5182716534,-0.1001891157\C,1.8024990616,-1.8791726896,-0.09519127
05\C,0.703979666,0.5135609078,0.0032244815\C,-1.7889763558,-1.85828929
15,0.0907842977\C,-3.0128187081,-2.5142072943,0.1018019697\C,-4.213537
2743,-1.8185360222,0.0368859225\C,-4.2159859466,-0.4298452987,-0.03028
38792\C,-2.9897046668,0.1843698645,-0.0384531585\C,-1.7624105658,-0.47
10433324,0.0087806839\C,-0.6990657205,0.5374036835,-0.0112874451\C,-1.
3093235631,1.7878562644,-0.0365369655\C,-0.6689544734,3.0107644154,-0.
0155848998\C,0.7109082074,3.0035868121,0.0345803248\C,1.3655979063,1.7
752993241,0.0417019739\C,2.9741194017,0.2232233307,0.0400622389\N,-2.7
687708735,1.6325340392,-0.0801845055\H,1.2697738144,3.9303757861,0.061
3787121\H,-1.2234436053,3.9407629728,-0.0321747931\C,1.7500191048,-0.4
845209411,-0.0126874702\N,2.7112431256,1.5664034799,0.0755185086\H,3.4

061809739,2.2930078316,0.0975131975\H,-5.1537194406,-2.3552409981,0.04
61748071\H,5.1673527299,-2.3186654579,-0.0312131072\H,-3.1627946362,2.
0446656659,-0.9315577356\\Version=ES64L-G16RevC.01\State=1-A\HF=-802.4
577651\RMSD=4.959e-09\RMSF=2.733e-06\Dipole=-3.3599902,2.8620023,-0.07
32637\Quadrupole=25.8770811,4.5127745,-30.3898556,-4.5126886,1.0204521
,-0.3040552\PG=C01 [X(C18H13N2)]\\@

7.2.2 Cinnolinocarbazole (4)

4 [(*M*)-enantiomer]

1\1\GINC-N1021\SP\RPBE1PBE\def2TZVP\C18H11N3\KA_PT6974\28-Oct-2024\0\\
# sp scrf=(solvent=dichloromethane) def2tzvp pbe1pbe empiricaldispersi
on=gd3bj\\Title Card Required\\0,1\H,0,-4.943311,-0.921124,0.441768\H,
0,-0.29851,-1.952308,-0.900582\H,0,0.867134,-2.323086,0.654868\H,0,2.9
9847,-3.498887,0.779943\H,0,5.143292,0.084119,-0.218844\H,0,-1.996388,
-3.706044,-0.960582\C,0,4.212332,-0.446379,-0.05775\C,0,4.193514,-1.79
274,0.249766\C,0,2.986121,-2.451602,0.502569\C,0,1.775899,-1.789843,0.
414997\C,0,0.729981,0.589262,-0.063054\C,0,-1.295121,-1.741223,-0.5431
83\C,0,-2.257364,-2.719045,-0.596991\C,0,-3.576758,-2.450445,-0.200827
\C,0,-3.924214,-1.181241,0.181177\C,0,-2.958103,-0.160891,0.205371\C,0
,-1.607805,-0.447116,-0.091437\C,0,-0.683791,0.641642,-0.000933\C,0,-1
.285993,1.917088,0.111691\C,0,-0.534767,3.107414,-0.023002\C,0,0.80965
1,3.064319,-0.243443\C,0,1.427582,1.804993,-0.237265\C,0,2.99286,0.215
437,-0.128046\H,0,1.394534,3.966921,-0.371837\H,0,-1.068558,4.04704,0.
047797\C,0,1.749488,-0.436661,0.05885\N,0,2.761983,1.552509,-0.323676\
H,0,3.475828,2.255506,-0.408871\H,0,-4.321449,-3.236679,-0.230722\H,0,
5.127013,-2.338863,0.317359\N,0,-3.413267,1.106623,0.472501\N,0,-2.620
02,2.095952,0.36814\\Version=ES64L-G16RevC.01\State=1-A\HF=-856.157310
4\RMSD=4.372e-09\Dipole=2.7452314,-0.551449,-0.5703985\Quadrupole=1.30
61509,7.8703496,-9.1765005,17.9833331,0.9143437,-2.0906557\PG=C01 [X(C
18H11N3)]\\@

4 [transition state]

1\1\GINC-N1617\SP\RPBE1PBE\def2TZVP\C18H11N3\KA_PT6974\28-Oct-2024\0\\
# sp scrf=(solvent=dichloromethane) def2tzvp pbe1pbe empiricaldispersi
on=gd3bj\\Title Card Required\\0,1\H,0,5.070512,-0.785768,-0.000011\H,
0,0.345267,-2.135418,0.000035\H,0,-1.128485,-2.556228,-0.000112\H,0,-3
.273645,-3.534574,-0.000094\H,0,-5.177334,0.308476,0.000058\H,0,2.0682
04,-3.850796,0.000088\C,0,-4.291671,-0.316076,0.000026\C,0,-4.375419,-
1.690769,-0.000011\C,0,-3.207364,-2.453198,-0.000058\C,0,-1.955158,-1.
86749,-0.000063\C,0,-0.729192,0.536371,-0.000007\C,0,1.372474,-1.84211
3,0.000035\C,0,2.350386,-2.804478,0.00006\C,0,3.704295,-2.444583,0.000
044\C,0,4.039024,-1.117104,0.000004\C,0,3.042211,-0.126403,-0.000017\C
,0,1.669744,-0.471898,-0.000002\C,0,0.705224,0.596596,-0.000015\C,0,1.
304459,1.887074,-0.000022\C,0,0.555048,3.083569,0.000001\C,0,-0.800308
,3.044905,0.000033\C,0,-1.407111,1.783901,0.000025\C,0,-3.027187,0.259
041,0.00002\H,0,-1.405361,3.943527,0.000061\H,0,1.106308,4.015067,-0.0
00003\C,0,-1.80623,-0.46925,-0.000017\N,0,-2.747606,1.592847,0.000044\
H,0,-3.430497,2.330581,0.000056\H,0,4.473202,-3.207633,0.000062\H,0,-5
.343482,-2.177133,-0.000007\N,0,3.496019,1.165125,-0.000048\N,0,2.6567
25,2.116503,-0.000047\\Version=ES64L-G16RevC.01\State=1-A\HF=-856.1511
056\RMSD=6.739e-09\Dipole=-2.8096155,-0.5513175,0.0000705\Quadrupole=1
.5355141,8.6378931,-10.1734072,-18.3406765,0.0000771,0.0003684\PG=C01
[X(C18H11N3)]\\@

4 [S_1]

1\1\GINC-N0932\FOpt\RPBE1PBE TDA-FC\def2TZVP\C18H11N3\KA_PT6974\14-Oct
-2024\0\\# opt tda=(nstates=50,root=1,singlet) scrf=(cpcm,solvent=dich
loromethane) def2tzvp pbe1pbe\\Title Card Required\\0,1\H,-4.952667582

,-0.9648865497,-0.596666526\H,-0.3585478531,-1.853156876,1.0330001912\
H,0.7446281166,-2.3085711076,-0.6121575145\H,2.8260835605,-3.573013992
8,-0.7635112747\H,5.1273582679,-0.0688555969,0.1669031979\H,-2.0381462
568,-3.6490307512,1.0620904055\C,4.172928526,-0.5620015578,0.023820055
1\C,4.0964370416,-1.9086673441,-0.2724105067\C,2.8604108589,-2.5226175
329,-0.4993706861\C,1.6797020004,-1.810001916,-0.3988512853\C,0.730002
9466,0.6141515618,0.0583074744\C,-1.3465910941,-1.660852338,0.63874407
06\C,-2.3009793304,-2.6755539507,0.667459966\C,-3.5824453759,-2.437843
5074,0.1788606919\C,-3.9447429381,-1.1826795966,-0.2674797263\C,-2.993
7438858,-0.1498544608,-0.2401783341\C,-1.6290156629,-0.3999115578,0.13
1283077\C,-0.6765473207,0.7009759328,0.0114225895\C,-1.2201537508,2.01
79864542,-0.0956158848\C,-0.4398947845,3.1807695355,0.0446528518\C,0.9
135944914,3.0771151647,0.2405863256\C,1.4835027566,1.8060035807,0.2196
74704\C,2.9821470748,0.1522173557,0.1069941904\H,1.5280540235,3.960431
2684,0.367488059\H,-0.930293831,4.1443932518,-0.0043243915\C,1.7119254
618,-0.4529504549,-0.0584244276\N,2.8135640694,1.4948802939,0.29807817
96\H,3.5583922799,2.1677738443,0.3522161109\H,-4.3164866454,-3.2357610
18,0.1741341897\H,5.0072111126,-2.4914883082,-0.3487919558\N,-3.281529
1431,1.1300266061,-0.5177626219\N,-2.5544331336,2.1052035673,-0.319542
1946\\Version=ES64L-G16RevC.01\State=1-A\HF=-856.0996414\RMSD=9.046e-0
9\RMSF=7.694e-06\Dipole=1.7111305,0.4931225,0.2154351\PG=C01 [X(C18H11
N3)]\\@

4 [T_1]

1\1\GINC-N0233\FOpt\RPBE1PBE TDA-FC\def2TZVP\C18H11N3\KA_PT6974\13-Nov
-2024\0\\# opt def2tzvp pbe1pbe TDA=(triplets,nstates=10) scrf=(cpcm,s
olvent=dichloromethane) empiricaldispersion=gd3bj\\Title Card Required
\\0,1\H,-4.9047473869,-1.0486252477,0.6159267278\H,-0.3073056458,-1.85
67356508,-1.0267405449\H,0.7989776135,-2.2817698825,0.6561031221\H,2.9
115714899,-3.5034110181,0.7952998444\H,5.129553155,0.043145531,-0.1767
077246\H,-1.9751220391,-3.6524025454,-1.1338463123\C,4.1867159089,-0.4
677977319,-0.0217668184\C,4.1397885818,-1.8145638605,0.2838247174\C,2.
9199150046,-2.4541468873,0.5255420195\C,1.7233891354,-1.7676226658,0.4
329005403\C,0.721803426,0.6265727503,-0.0329478102\C,-1.299848487,-1.6
827714958,-0.6358504388\C,-2.2472887975,-2.6954461464,-0.7052878013\C,
-3.5338078432,-2.488148173,-0.2245227187\C,-3.8958080121,-1.248268261,
0.2768043593\C,-2.955036171,-0.2261739764,0.3028538427\C,-1.6124667627
,-0.4408094443,-0.0929263267\C,-0.6811623752,0.6817741654,0.0400596063
\C,-1.2726458099,1.9709830392,0.0979224498\C,-0.5311911439,3.147329591
9,-0.1072344106\C,0.8271884165,3.0865322924,-0.2917591096\C,1.43947015
29,1.8356928141,-0.2250109627\C,2.9811586703,0.2190671805,-0.100207789
6\H,1.4117464583,3.9844278637,-0.4510045881\H,-1.0533968688,4.09573492
93,-0.1019216676\C,1.7277022274,-0.4138394401,0.0835677587\N,2.7752803
722,1.5575574645,-0.3049259265\H,3.501768133,2.2461995407,-0.396080329
1\H,-4.2653694819,-3.2863942705,-0.264513809\H,5.0631159589,-2.3774621
252,0.3555162186\N,-3.2334366278,1.0380337199,0.7841430982\N,-2.630248
2517,2.0279859399,0.2485427837\\Version=ES64L-G16RevC.01\State=1-A\HF=
-856.1238387\RMSD=9.207e-09\RMSF=2.361e-05\Dipole=2.0003101,0.0150082,
-0.4794867\PG=C01 [X(C18H11N3)]\\@

4·H⁺ [protonated]

1\1\GINC-N0907\FOpt\RPBE1PBE\def2TZVP\C18H12N3(1+)\KA_PT6974\16-Oct-20
24\0\\# opt pbe1pbe/def2tzvp scrf=(cpcm,solvent=dichloromethane) empir
icaldispersion=gd3bj\\Title Card Required\\1,1\H,4.9436236152,-0.88445
00804,-0.4343418602\H,0.2859550466,-1.9191635945,0.9087277458\H,-0.847
5192899,-2.3096374592,-0.6884848192\H,-2.9675894344,-3.5016964256,-0.8
125995551\H,-5.1438121474,0.0514457965,0.2229438368\H,1.9955347698,-3.
6645463079,0.9615401145\C,-4.2092255386,-0.4696594991,0.0555385416\C,-
4.1763199691,-1.8138898655,-0.2624149521\C,-2.9642432982,-2.457177802,
-0.5260466642\C,-1.7588370042,-1.7860211285,-0.438334321\C,-0.74300804
34,0.6031099696,0.0553948094\C,1.282132073,-1.7040229999,0.5550027662\
C,2.2491806342,-2.6749269027,0.6025424009\C,3.5677564597,-2.4002279733
,0.2044421678\C,3.9215276603,-1.1330175558,-0.1740926642\C,2.938444785
7,-0.1362650112,-0.1882800161\C,1.5871077447,-0.4075410301,0.107534164
6\C,0.6558724579,0.6745918523,0.0123834567\C,1.2396972436,1.9846154954
,-0.0891450423\C,0.4660174297,3.1703682131,0.0551715254\C,-0.869616877
1,3.0930529055,0.2690797057\C,-1.4623986352,1.8151685462,0.2427854827\
C,-2.995694831,0.1970415827,0.1268917895\H,-1.4771102889,3.9787877904,
0.4044466301\H,0.9836031773,4.1183669602,-0.0084974301\C,-1.7492010264
,-0.4378236681,-0.0695074696\N,-2.7765980488,1.5424639039,0.3280557807
\H,-3.5006915008,2.2324336877,0.4466186918\H,4.3140727827,-3.184037555
2,0.2302676615\H,-5.1037057567,-2.3694263448,-0.3310644301\N,3.2948414
454,1.1551654094,-0.4412754056\N,2.5331240074,2.1810727011,-0.34110613
45\H,4.2615400372,1.3628723001,-0.6654745074\\Version=ES64L-G16RevC.01
\State=1-A\HF=-856.6026088\RMSD=5.795e-09\RMSF=7.794e-06\Dipole=1.4341
693,0.8785985,0.0472326\Quadrupole=27.2121583,0.8771137,-28.0892721,-9
.35714,-4.5616153,0.5176873\PG=C01 [X(C18H12N3)]\\@

7.2.3 Indolo[2,3-*k*]phenanthridines

3 [R = H, (*M*)-enantiomer]

1\1\GINC-N1607\FOpt\RPBE1PBE\def2TZVP\C19H12N2\KA_PT6974\06-Aug-2024\0
\\# opt pbe1pbe/def2tzvp scrf=(cpcm,solvent=dichloromethane) empirical
dispersion=gd3bj\\Title Card Required\\0,1\H,-5.3514327006,-3.01143414
39,0.014403181\H,-3.4072884494,1.5145552737,0.7699635541\H,-2.39800678
95,1.8640633896,-0.9241574343\H,-2.2848260174,4.302540236,-0.993369627
4\H,-0.1841215007,5.4742506,-0.3630561977\H,1.830888367,4.1370603524,0
.2020426561\H,-6.7851895182,-1.1026207698,0.6930706471\H,-2.7246507453
,-3.6890155067,-0.381671641\H,-5.7903961697,1.1202859271,1.0971687176\
C,0.8812570586,3.658897904,-0.0080186529\C,-0.2330318145,4.3920363615,
-0.3325216184\C,-1.4212975851,3.7328640781,-0.6705000685\C,-1.49285234
53,2.3619429113,-0.6095561938\C,-0.3903562631,1.5867501021,-0.21111234
02\C,-0.415962286,0.1480949338,-0.1348264809\C,-3.8117210809,0.5352627
963,0.5572747203\C,-5.1602409876,0.3069964986,0.7573350443\C,-5.723026
141,-0.9541667501,0.5376855761\C,-4.9347841009,-2.0205523719,0.1513232
91\C,-3.577162795,-1.7869954476,-0.0300923808\C,-2.9871033508,-0.50793
36924,0.1196884393\C,-1.5645903226,-0.6832670943,-0.1189433372\C,-1.37
94846008,-2.0708358768,-0.314325137\C,-0.123783885,-2.6838363261,-0.40
76188924\C,0.9751085518,-1.8881042839,-0.2529940816\C,0.8459445473,-0.
4893934602,-0.1102719372\C,0.8395132302,2.2545370498,0.0181136966\N,-2
.5911759462,-2.6947743853,-0.3153469539\N,2.0261138046,1.5940424672,0.
2127097274\C,2.009804958,0.3049139906,0.0916240148\H,1.9696876041,-2.3
200321991,-0.2499048431\H,-0.0379835559,-3.7548365687,-0.5452710958\H,
2.9642960299,-0.2130729451,0.1780988281\\Version=ES64L-G16RevC.01\Stat
e=1-A\HF=-840.1573852\RMSD=5.065e-09\RMSF=1.708e-05\Dipole=-1.3252052,
-2.0283361,-0.2906124\Quadrupole=-4.8540862,16.2610859,-11.4069997,-3.
4094708,-0.9549651,1.0745283\PG=C01 [X(C19H12N2)]\\@

3 [R = H, transition state]

1\1\GINC-N1537\SP\RPBE1PBE\def2TZVP\C19H12N2\KA_PT6974\28-Oct-2024\0\\
# sp scrf=(solvent=dichloromethane) def2tzvp pbe1pbe empiricaldispersi
on=gd3bj\\Title Card Required\\0,1\H,0,-5.177508,0.310999,-0.000002\H,
0,-1.131643,-2.551561,-0.000001\H,0,0.315077,-2.087275,-0.000003\H,0,1
.963815,-3.859163,0.000004\H,0,4.39494,-3.296531,0.000006\H,0,5.068837
,-0.900936,0.\H,0,-5.345102,-2.175496,-0.000003\H,0,-3.438436,2.32998,
-0.000005\H,0,-3.272183,-3.530909,-0.000002\C,0,4.02527,-1.192431,0.\C
,0,3.648694,-2.510757,0.000004\C,0,2.287097,-2.824736,0.000003\C,0,1.3
48864,-1.821763,0.000001\C,0,1.683784,-0.460866,0.\C,0,0.712472,0.6170
45,-0.000001\C,0,-1.956643,-1.861979,-0.000001\C,0,-3.208206,-2.449357
,-0.000002\C,0,-4.377142,-1.688829,-0.000002\C,0,-4.292402,-0.314478,-
0.000001\C,0,-3.027918,0.26133,0.\C,0,-1.804085,-0.463363,0.\C,0,-0.72
594,0.545717,0.000001\C,0,-1.412325,1.790674,0.000002\C,0,-0.820308,3.
056105,0.000005\C,0,0.536236,3.103619,0.000002\C,0,1.293332,1.914724,-
0.000002\C,0,3.074499,-0.157467,-0.000002\N,0,-2.753459,1.594454,0.000
001\N,0,3.574737,1.116475,-0.000005\C,0,2.707106,2.072179,-0.000005\H,
0,1.061165,4.052017,0.000003\H,0,-1.433977,3.94885,0.000011\H,0,3.0917
55,3.091328,-0.000008\\Version=ES64L-G16RevC.01\State=1-A\HF=-840.1492
169\RMSD=4.095e-09\Dipole=-2.365295,0.781321,-0.0000012\Quadrupole=0.0
408745,12.4262409,-12.4671154,-9.6900548,0.0000464,-0.0000003\PG=C01 [
X(C19H12N2)]\\@

3 [R = H, S_1]

1\1\GINC-N1307\FOpt\RPBE1PBE TDA-FC\def2TZVP\C19H12N2\KA_PT6974\14-Oct
-2024\0\\# opt def2tzvp pbe1pbe TDA=(singlet,root=1,nstates=50) scrf=(
cpcm,solvent=dichloromethane) empiricaldispersion=gd3bj\\Title Card Re
quired\\0,1\H,0.0359323281,0.0478166474,0.0163656698\H,-0.0661379339,-
0.1342050099,5.0083374466\H,2.0228391493,0.0471641363,5.4120328225\H,2
.0263205203,0.9896356058,7.6709791495\H,2.2830291386,-0.4778220527,9.6
540371336\H,2.6480931593,-2.9263988881,9.3222772774\H,-1.2941092552,1.
6919403515,1.3332627072\H,1.5826226744,-2.2212102813,0.3281074971\H,-1
.3567642742,1.5676726657,3.7926057955\C,2.4681902334,-2.267694778,8.48
0748412\C,2.2754827814,-0.9055188447,8.6588006594\C,2.1187561597,-0.08
29248298,7.5411867618\C,2.0934931034,-0.615092806,6.2631364499\C,2.203
6969403,-2.0071640045,6.0491048886\C,2.1348940113,-2.6370845063,4.7847
280255\C,0.0035665583,-0.0746201075,3.9311884172\C,-0.7355898538,0.873
988333,3.2388677399\C,-0.7081785572,0.9406501351,1.848457908\C,0.04286
92778,0.0246766001,1.0992176315\C,0.7668368495,-0.9163843886,1.7900918
18\C,0.8149000426,-0.9729683575,3.2129131669\C,1.6629630679,-2.0628253
447,3.5603203899\C,2.0070642677,-2.7066209632,2.3203678816\C,2.6429390
718,-3.9523445279,2.2416322572\C,2.8795640399,-4.597367967,3.440751754
5\C,2.6235541101,-3.986433211,4.6882230824\C,2.4743771225,-2.836639743
4,7.2003929161\N,1.5143696219,-1.9700464519,1.3008922614\N,2.7635313,-
4.1615406116,7.090684609\C,2.8773313401,-4.6758754582,5.8832490075\H,3
.2950835326,-5.5992698205,3.4420038378\H,2.8642408445,-4.411921008,1.2
877028607\H,3.1879145925,-5.717547152,5.8254296363\\Version=ES64L-G16R
evC.01\State=1-A\HF=-840.1521966\RMSD=9.677e-09\RMSF=5.318e-05\Dipole=
-0.6357019,0.6701109,-3.4851252\PG=C01 [X(C19H12N2)]\\@

3 [R = H, T_1]

1\1\GINC-N1429\FOpt\RPBE1PBE TDA-FC\def2TZVP\C19H12N2\KA_PT6974\13-Nov
-2024\0\\# opt def2tzvp pbe1pbe TDA=(triplets,nstates=10) scrf=(cpcm,s
olvent=dichloromethane) empiricaldispersion=gd3bj\\Title Card Required
\\0,1\H,-5.1141309648,-0.0372620555,0.3679755799\H,-0.7695682747,-2.15
45822182,-0.9508290649\H,0.1956140617,-1.8237825826,0.9996293481\H,1.7
948161011,-3.6660458937,1.1347697129\H,4.1458973871,-3.3506755601,0.39
51752192\H,4.8988697778,-1.1138982536,-0.3854234137\H,-5.0083159107,-2
.4063921274,-0.3889917322\H,-3.5145323539,2.2058114382,0.6370257189\H,
-2.8719507131,-3.4261550384,-1.0601110494\C,3.865123455,-1.2911487157,
-0.1121075537\C,3.4436499553,-2.52864006,0.3270443854\C,2.1076198241,-

2.7101145319,0.730611945\C,1.2050000157,-1.6863269324,0.6383218188\C,1
.5890126498,-0.4130420991,0.1286789296\C,0.7101635601,0.6679046019,-0.
024329566\C,-1.694190952,-1.7068800335,-0.6141844761\C,-2.8781988605,-
2.4084219787,-0.688116662\C,-4.0963584904,-1.8263488167,-0.3155587836\
C,-4.1653756925,-0.4956596441,0.1175282917\C,-2.9893168708,0.212622977
5,0.1899762605\C,-1.7122044996,-0.3742731424,-0.1237976457\C,-0.744617
703,0.6150835242,0.0834764785\C,-1.4539982773,1.8418131787,0.369151085
6\C,-0.8623183467,3.0710283241,0.322759317\C,0.5224424399,3.1158737669
,-0.0527187662\C,1.276377602,1.9564828,-0.2221487795\C,2.9789043888,-0
.2127142735,-0.188825818\N,-2.7815980676,1.5263515324,0.5276601168\N,3
.4762222651,1.0054198823,-0.5560644193\C,2.6689838592,2.0221209867,-0.
5294498306\H,1.0075825393,4.0772442567,-0.1745706203\H,-1.4247278441,3
.9822916053,0.4772970732\H,3.0915169397,2.9963850828,-0.7714181\\Versi
on=ES64L-G16RevC.01\State=1-A\HF=-840.1444915\RMSD=2.144e-09\RMSF=1.58
9e-05\Dipole=-2.3681846,0.5624626,0.3778826\PG=C01 [X(C19H12N2)]\\@

3a [R = CH_3]

1\1\GINC-N1240\FOpt\RPBE1PBE\def2TZVP\C20H14N2\KA_PT6974\17-May-2023\0
\\# opt pbe1pbe/def2tzvp scrf=(cpcm,solvent=dichloromethane) empirical
dispersion=gd3bj\\Title Card Required\\0,1\H,0.011639899,-0.0081444795
,0.028001434\H,0.0199841821,-0.0162350654,5.0118559573\H,2.0136969598,
-0.0226254125,5.4062577408\H,2.081957028,0.970671601,7.6374382626\H,2.
3084880869,-0.48114254,9.6436576544\H,2.6022064544,-2.9296170186,9.345
9654485\H,-1.247901572,1.703326023,1.3092994524\H,1.4747236669,-2.3033
920117,0.3730181907\H,-1.259445816,1.6537768859,3.7774166637\C,2.43056
90481,-2.2780184614,8.4970915384\C,2.2791187802,-0.9221270362,8.654022
1665\C,2.1339772168,-0.1060499023,7.5258101979\C,2.072991899,-0.666031
8771,6.2717791948\C,2.152896722,-2.0564687198,6.0871330477\C,2.1053781
594,-2.6974465783,4.7984154021\C,0.0668288071,-0.0096035757,3.93211293
02\C,-0.6688996333,0.9276503037,3.2314617503\C,-0.6703468937,0.9507369
371,1.8332003872\C,0.0348371896,0.0076658896,1.1112386926\C,0.75617677
58,-0.9432146236,1.8230129529\C,0.8325018154,-0.9535067945,3.237650015
4\C,1.64095366,-2.107101149,3.5953333649\C,1.927334899,-2.7620501108,2
.3783452844\C,2.5439436311,-4.0146399012,2.3071340568\C,2.8266287112,-
4.646600926,3.485697087\C,2.6067564709,-4.0195313485,4.7333069826\C,2.
413973259,-2.8624878134,7.2191813998\N,1.4450224638,-2.0238653567,1.33
81891997\N,2.7097593483,-4.1966419334,7.1347602337\C,2.8598163648,-4.7
305955792,5.9593324459\H,3.2419980761,-5.6450442215,3.4619709959\H,2.7
393969487,-4.4853247021,1.3512974632\C,3.2848846617,-6.1654809409,5.92
14849852\H,3.3638381379,-6.5390810351,6.9403822682\H,4.2561526912,-6.2
766050738,5.4314809582\H,2.5714591325,-6.7827395941,5.3698242415\\Vers
ion=ES64L-G16RevC.01\State=1-A\HF=-879.444364\RMSD=7.252e-09\RMSF=1.28
9e-05\Dipole=-0.1044524,0.0233756,-2.3822879\Quadrupole=-9.8197005,0.8
994524,8.9202481,-5.0438244,-0.6590506,8.2046994\PG=C01 [X(C20H14N2)]\
\@

3a·H^+ [R = CH_3]

1\1\GINC-N1526\FOpt\RPBE1PBE\def2TZVP\C20H15N2(1+)\KA_VR6109\27-Nov-20
23\0\\# opt=tight pbe1pbe/def2tzvp scrf=(cpcm,solvent=dichloromethane)
geom=connectivity empiricaldispersion=gd3bj\\Title Card Required\\1,1
\H,0.010329702,-0.0029253762,0.0165861024\H,-0.0174837904,-0.015449026
7,5.0021272042\H,2.0047037064,0.0026410361,5.3704499887\H,2.0646738453
,1.0040153299,7.5995106879\H,2.2789610163,-0.4321653009,9.6126946717\H
,2.5701985781,-2.8726672682,9.3493515269\H,-1.2601070805,1.7047825576,
1.2934538009\H,1.4994132395,-2.2782520039,0.3640962444\H,-1.2893023005
,1.6514621415,3.759141688\C,2.4060692092,-2.2370428942,8.4870039834\C,
2.2545308171,-0.878694459,8.6265921286\C,2.1174454188,-0.0723064585,7.
4925022807\C,2.0627271785,-0.6345256742,6.239560786\C,2.1399609655,-2.
0242911548,6.0624610954\C,2.09185701,-2.6799582441,4.7782036154\C,0.04
0017977,-0.0071628204,3.9230265052\C,-0.6924193023,0.9279971539,3.2172
026404\C,-0.6839216291,0.953005233,1.8193613792\C,0.0270444399,0.01291
72633,1.0994938438\C,0.745239654,-0.933269017,1.8173442227\C,0.8119933

612,-0.9451333653,3.2293306326\C,1.6205654669,-2.0990258027,3.58669020
49\C,1.9187386511,-2.7517917151,2.3630438062\C,2.5535492459,-3.9983268
446,2.2837546813\C,2.841456801,-4.6340303588,3.4526647354\C,2.60862795
03,-4.0078804176,4.7025925443\C,2.3835994,-2.7971215206,7.2090723766\N
,1.4390035665,-2.0177756311,1.3343644006\N,2.664513006,-4.1351940126,7
.054303174\C,2.8473789408,-4.7303882425,5.890275847\H,3.2642640153,-5.
6287136478,3.4271793287\H,2.7548452211,-4.4594284941,1.3252264883\C,3.
2688496479,-6.1541894044,5.9119020881\H,3.3358406632,-6.5309360267,6.9
312821128\H,4.2477697663,-6.2631078524,5.4405119219\H,2.5616025576,-6.
7725827176,5.3573246912\H,2.8114479696,-4.6782162936,7.8954002445\\Ver
sion=ES64L-G16RevC.01\State=1-A\HF=-879.895996\RMSD=4.362e-09\RMSF=2.2
54e-06\Dipole=1.3181493,-2.8549818,1.0550682\Quadrupole=-24.931967,4.4
803266,20.4516403,-14.7319648,8.0541454,-7.2484895\PG=C01 [X(C20H15N2)
]\\@

3b [R = *t*Bu]

1\1\GINC-N0940\FOpt\RPBE1PBE\def2TZVP\C23H20N2\KA_PT6974\21-May-2023\0
\\# opt=tight pbe1pbe/def2tzvp scrf=(cpcm,solvent=dichloromethane) emp
iricaldispersion=gd3bj\\Title Card Required\\0,1\H,0.,0.,0.\H,0.,0.,4.
98449639\H,2.0547232181,0.,5.3003675634\H,2.1751677326,1.0561906442,7.
5003968534\H,2.3441077442,-0.3404630533,9.5500624172\H,2.5219606642,-2
.8091534082,9.3293668723\H,-1.2505886726,1.7221909311,1.2760551444\H,1
.4463576377,-2.3069487082,0.3523716553\H,-1.2672130617,1.6774204139,3.
7441849241\C,2.3797718863,-2.1765416481,8.4609016755\C,2.2914793422,-0
.8110575452,8.5751303253\C,2.1786298747,-0.0247062989,7.4215885904\C,2
.0893988369,-0.6186993227,6.185230115\C,2.1090208211,-2.0170494073,6.0
466407026\C,2.0460808479,-2.7015782328,4.7834582347\C,0.0450347936,0.0
031809632,3.9044374108\C,-0.6818442432,0.9451805506,3.2007916503\C,-0.
6801023685,0.9659209418,1.8024122146\C,0.0201760306,0.0168644556,1.083
3181134\C,0.7327366126,-0.9383755888,1.7979081093\C,0.8042297002,-0.94
76845375,3.2127861181\C,1.5995994639,-2.1096216279,3.5740565096\C,1.88
65438221,-2.7643731664,2.3608940389\C,2.5002915224,-4.0170094686,2.307
2517714\C,2.7525775912,-4.6515107323,3.4911499701\C,2.5146203225,-4.04
24185422,4.7478716004\C,2.3330709238,-2.7960124043,7.1997591234\N,1.41
58587577,-2.0247279532,1.3165916615\N,2.5564777381,-4.1412867184,7.149
0209026\C,2.704368382,-4.7406935357,6.0063881891\H,3.1649163661,-5.644
9933958,3.4483912456\H,2.7183298571,-4.492907332,1.3589137435\C,3.0462
647967,-6.2350351224,6.0928477111\C,2.0033029792,-7.0920749551,5.36236
10769\C,3.0346275576,-6.6917678589,7.5517708691\C,4.46538168,-6.494425
198,5.5666796918\H,2.2450923426,-8.148675708,5.5024747505\H,1.01112428
25,-6.9172088627,5.7862282852\H,1.9421600246,-6.9051207601,4.292365213
3\H,4.706917785,-7.55331597,5.6896614078\H,4.6059519483,-6.2425071662,
4.5171581319\H,5.1901617533,-5.9158849604,6.1448518433\H,3.7683078035,
-6.1507260479,8.1490799399\H,2.0564342612,-6.5408605129,8.0097860168\H
,3.273737762,-7.7578742233,7.5844140934\\Version=ES64L-G16RevC.01\Stat
e=1-A\HF=-997.2781864\RMSD=5.735e-09\RMSF=7.818e-07\Dipole=-0.0491345,
-0.0149372,-2.2393115\Quadrupole=-10.6451127,0.3687454,10.2763673,-4.8
365875,0.6619593,4.7985479\PG=C01 [X(C23H20N2)]\\@

3c [R = CF_3]

1\1\GINC-N0502\FOpt\RPBE1PBE\def2TZVP\C20H11F3N2\KA_PT6974\21-May-2023
\0\\# opt=tight pbe1pbe/def2tzvp scrf=(cpcm,solvent=dichloromethane) e
mpiricaldispersion=gd3bj\\Title Card Required\\0,1\H,-0.0000809819,-0.
0001047657,-0.000018146\H,-0.0001236381,0.000079888,4.9843706278\H,2.0
11341247,-0.0000584722,5.3539838744\H,2.0796372737,1.0113620316,7.5746
924853\H,2.2939659523,-0.4148321727,9.5976491503\H,2.579524611,-2.8702
502559,9.3291172105\H,-1.2631524943,1.7124615214,1.2773907875\H,1.4694
459014,-2.2891079534,0.3492884837\H,-1.278447823,1.6661731009,3.745124
8828\C,2.412038452,-2.2257782489,8.4744823335\C,2.2653295197,-0.868809
7011,8.6142812046\C,2.1268403865,-0.0665953165,7.4742911848\C,2.066413
219,-0.6357270852,6.225096326\C,2.1419002245,-2.0283497211,6.055061750
1\C,2.0909906121,-2.6795256053,4.7722207189\C,0.0492323168,0.004979954

7,3.9047422498\C,-0.6860681309,0.9400437875,3.2013223334\C,-0.68542944
33,0.9612296224,1.8028951584\C,0.0214063255,0.0181663541,1.0831235951\
C,0.7428722526,-0.9300101919,1.7978045349\C,0.8172097937,-0.9383123659
,3.21199196\C,1.6263179715,-2.090961738,3.5703456179\C,1.9159857634,-2
.7454661835,2.354244454\C,2.5381728783,-3.9959009738,2.284626663\C,2.8
207721381,-4.6326042904,3.4582518215\C,2.593471964,-4.0057888348,4.707
7622687\C,2.3950531569,-2.8178797144,7.2004390724\N,1.4331434358,-2.01
15016522,1.3149424645\N,2.6879219353,-4.1496716387,7.1231391081\C,2.83
08220402,-4.6740009724,5.9515685124\C,3.2559420593,-6.1344093424,5.960
8432347\H,3.2391240392,-5.6278157813,3.4222944272\H,2.7386820196,-4.46
45790119,1.3290616255\F,3.3666162412,-6.623269168,7.1889488956\F,2.378
1600213,-6.9126387301,5.3047854901\F,4.448601235,-6.3005634124,5.36102
56704\\Version=ES64L-G16RevC.01\State=1-A\HF=-1177.0352413\RMSD=2.985e
-09\RMSF=5.577e-07\Dipole=-0.6108002,1.6897118,-2.3880823\Quadrupole=-
9.3131264,-7.1509641,16.4640905,-1.3865921,0.0146672,8.5911025\PG=C01
[X(C20H11F3N2)]\\@

3d [R = CH_2Cl]

1\1\GINC-N0327\FOpt\RPBE1PBE\def2TZVP\C20H13Cl1N2\KA_PT6974\17-May-202
3\0\\# opt=tight pbe1pbe/def2tzvp scrf=(cpcm,solvent=dichloromethane)
empiricaldispersion=gd3bj\\Title Card Required\\0,1\H,0.0160346764,-0.
0096432821,0.0270077804\H,0.0203220687,-0.0165179317,5.0110540521\H,2.
0199144004,-0.023068984,5.4019824668\H,2.0788293654,0.9711440233,7.632
0406499\H,2.291287714,-0.4751709882,9.6422176741\H,2.5832373301,-2.925
6425101,9.3517440163\H,-1.2437078827,1.7021830835,1.3070932726\H,1.485
3143093,-2.3018778397,0.3729230708\H,-1.2570071666,1.6534393062,3.7750
459445\C,2.4170396478,-2.2748640919,8.501392016\C,2.2667835617,-0.9191
301207,8.653980915\C,2.1295088936,-0.1057689436,7.5221586385\C,2.07433
24759,-0.6656763177,6.2682521342\C,2.1536167739,-2.0564666956,6.085831
371\C,2.1075026535,-2.6973391106,4.7974669445\C,0.0684030481,-0.010212
6783,3.9313835691\C,-0.6662388515,0.9269808283,3.2298511531\C,-0.66667
62281,0.9496072469,1.8315132214\C,0.0385151531,0.0062885303,1.11016960
15\C,0.7594631909,-0.9441478597,1.8227880378\C,0.8343588792,-0.9541498
386,3.2372789326\C,1.643188465,-2.1070034252,3.5951053996\C,1.93195746
39,-2.7613688094,2.3784557144\C,2.5519964087,-4.0129533564,2.306875631
7\C,2.8353050979,-4.6467905353,3.4829538345\C,2.610523773,-4.020078031
9,4.7308593867\C,2.40703478,-2.8588845029,7.2230738781\N,1.4491658307,
-2.0251145112,1.3387845631\N,2.7033619943,-4.1908452408,7.1384684555\C
,2.8556165988,-4.7164785929,5.9610216043\H,3.2662951865,-5.6384284818,
3.454119391\H,2.751497805,-4.4809941359,1.3506496472\C,3.2737855266,-6
.1540019082,5.9587004067\H,3.1285298847,-6.5678416752,6.9518839973\H,2
.7467726076,-6.7544316405,5.2208842306\Cl,5.0265530431,-6.3145591762,5
.5870186297\\Version=ES64L-G16RevC.01\State=1-A\HF=-1338.9098019\RMSD=
3.071e-09\RMSF=3.116e-07\Dipole=-1.0839049,0.5838887,-2.1958178\Quadru
pole=-14.2524281,0.010465,14.2419631,1.58937,-0.3710992,6.1462796\PG=C
01 [X(C20H13Cl1N2)]\\@

3e [R = CH_2N_3]

1\1\GINC-N0521\FOpt\RPBE1PBE\def2TZVP\C20H13N5\KA_PT6974\21-May-2023\0
\\# opt=tight pbe1pbe/def2tzvp scrf=(cpcm,solvent=dichloromethane) emp
iricaldispersion=gd3bj\\Title Card Required\\0,1\H,0.0123964155,-0.022
5330005,0.0276045344\H,0.0224479407,-0.0150739965,5.0116079457\H,2.018
888928,-0.0247617023,5.394451338\H,2.115821611,0.9835009036,7.61619409
3\H,2.3646956411,-0.4504088213,9.6315213802\H,2.6494036013,-2.90277684
05,9.3508648509\H,-1.2398923102,1.6973861447,1.3041219631\H,1.47424901
22,-2.3189774214,0.3788384898\H,-1.2507839575,1.6556774539,3.772233602
2\C,2.469411263,-2.2571347529,8.4993751048\C,2.3224245028,-0.900295236
5,8.6465491035\C,2.164565207,-0.0941359209,7.5124501723\C,2.0872307774
,-0.6622274009,6.2634252099\C,2.1632198491,-2.0543467629,6.0879965397\
C,2.1017284109,-2.7024330699,4.8037526991\C,0.0696882864,-0.0116827629
,3.9318496425\C,-0.6627911965,0.9258815849,3.2284780919\C,-0.664674818
8,0.9445089445,1.8301074749\C,0.0366378073,-0.0032548855,1.1106989188\

C,0.7554160854,-0.9538738979,1.8252200003\C,0.8322369222,-0.9597735077
,3.2396604592\C,1.6370353007,-2.114622695,3.6001742043\C,1.9224030644,
-2.7735854688,2.3851665538\C,2.540827196,-4.0261769947,2.3165227831\C,
2.8217532487,-4.6579456539,3.4943904484\C,2.5991064882,-4.0276664937,4
.7409252709\C,2.4345809914,-2.8496531185,7.2257191427\N,1.4407031433,-
2.0387212244,1.3437405048\N,2.7211905645,-4.1851867771,7.1460436675\C,
2.8510324192,-4.7205344392,5.9710042904\C,3.2763698298,-6.16196103,5.9
664296763\H,3.2286129111,-5.6592589176,3.4693874949\H,2.7383656512,-4.
497550247,1.3615514795\H,3.4966049976,-6.4443433643,6.9973589187\H,4.1
83854893,-6.2950940901,5.3670874005\N,2.1910892231,-7.0028467674,5.429
7267589\N,2.4434001664,-8.190928744,5.3451112874\N,2.5746961211,-9.303
7635645,5.22686904\\Version=ES64L-G16RevC.01\State=1-A\HF=-1042.916723
4\RMSD=2.134e-09\RMSF=7.200e-07\Dipole=0.3452293,1.1515543,-2.0567877\
Quadrupole=-4.7657782,-11.1983593,15.9641376,-7.1360367,1.37859,4.2948
293\PG=C01 [X(C20H13N5)]\\@

3f [R = (*E*)-MeCH=CH]

1\1\GINC-N0923\FOpt\RPBE1PBE\def2TZVP\C22H16N2\KA_PT6974\17-May-2023\0
\\# opt pbe1pbe/def2tzvp scrf=(cpcm,solvent=dichloromethane) empirical
dispersion=gd3bj\\Title Card Required\\0,1\H,0.013898078,-0.0041573626
,0.030541511\H,0.0248520826,-0.0192636161,5.0151273199\H,2.0448824415,
-0.0307017985,5.3698410914\H,2.1768731664,0.9925293783,7.5842507345\H,
2.4105411597,-0.4347253141,9.6073798226\H,2.6375841134,-2.8929853825,9
.3437498345\H,-1.2357554045,1.7120951365,1.3147030732\H,1.4694208689,-
2.306878214,0.372520326\H,-1.2465827295,1.6602923676,3.7825550262\C,2.
4697568658,-2.2502245048,8.4874446798\C,2.3534111912,-0.8898264045,8.6
253247936\C,2.204060356,-0.0864858996,7.4873371105\C,2.1077827327,-0.6
629089149,6.2433911418\C,2.1569334149,-2.0572831381,6.0775749632\C,2.0
84105498,-2.7132935655,4.7992980699\C,0.0691342991,-0.0118925252,3.935
1094965\C,-0.6609510873,0.9307744155,3.2358336878\C,-0.6626185397,0.95
53618768,1.8374850271\C,0.0370419205,0.0094153492,1.1138266292\C,0.753
1088347,-0.9464959298,1.8240409944\C,0.8284008773,-0.9592680729,3.2386
054091\C,1.6279781756,-2.1189193831,3.5949424509\C,1.9148682212,-2.771
3738916,2.378106401\C,2.5379499217,-4.0207340324,2.3095711608\C,2.8054
879232,-4.6589740381,3.4879905036\C,2.5640236438,-4.0453971868,4.73992
98553\C,2.419107787,-2.8546342471,7.2171315008\N,1.4371765709,-2.03008
77348,1.3383345144\N,2.6614216766,-4.1925664976,7.1477295087\C,2.78997
4119,-4.7554108746,5.9770468143\C,3.1388653445,-6.1821087648,5.9542029
065\H,2.7542315059,-4.4847851421,1.355013532\C,3.7002918388,-6.8187598
017,6.9823196033\C,4.0419030535,-8.2633568863,6.9982281915\H,3.9247177
505,-6.2436257544,7.8771152771\H,3.2401948728,-5.6479089755,3.45499811
\H,3.5201879754,-8.7734956309,7.8147615363\H,5.1114808192,-8.407861908
2,7.181581947\H,3.7799774118,-8.754434661,6.0595200383\H,2.9200843455,
-6.7447892433,5.0536871777\\Version=ES64L-G16RevC.01\State=1-A\HF=-956
.7788214\RMSD=6.482e-09\RMSF=4.483e-06\Dipole=0.0439684,-0.2168745,-2.
1523273\Quadrupole=-12.8031863,2.5968619,10.2063244,-7.1319038,2.29751
13,3.2469187\PG=C01 [X(C22H16N2)]\\@

3g [R = (*E*,*E*)-Me-(CH=CH)$_2$]

1\1\GINC-N0940\FOpt\RPBE1PBE\def2TZVP\C24H18N2\KA_PT6974\21-May-2023\0
\\# opt=tight pbe1pbe/def2tzvp scrf=(cpcm,solvent=dichloromethane) emp
iricaldispersion=gd3bj\\Title Card Required\\0,1\H,0.0007933619,-0.000
0600568,-0.0003182073\H,0.0001822606,0.0006970124,4.9843457437\H,2.023
8345616,-0.0001750014,5.3394225408\H,2.1379410637,1.0341591833,7.54981
16258\H,2.3579932933,-0.3828496042,9.5817578933\H,2.5930877739,-2.8418
568927,9.3320388534\H,-1.2584670964,1.7154224693,1.2754744291\H,1.4644
586576,-2.2978408701,0.3532903185\H,-1.2749394125,1.6714514617,3.74341
95285\C,2.4298392181,-2.2035116647,8.4715282675\C,2.309456725,-0.84297
71169,8.6015831661\C,2.1676199525,-0.0452536871,7.4583763383\C,2.08174
0053,-0.6279964775,6.2165109362\C,2.1341654409,-2.0228892817,6.0579189
939\C,2.0702834557,-2.6854528635,4.7826074972\C,0.0468067748,0.0048343

743,3.9044184013\C,-0.685229515,0.9424476144,3.2004065958\C,-0.6836546
882,0.9625730775,1.8020098278\C,0.0214772712,0.0170866422,1.083016116\
C,0.7397581345,-0.9336835839,1.7979573823\C,0.8115671375,-0.9416343533
,3.2128172214\C,1.6152542169,-2.0966861302,3.5749867288\C,1.9079126125
,-2.7514730728,2.3611883159\C,2.5359956796,-3.9983526564,2.2989805068\
C,2.8025712439,-4.6318889189,3.4801446823\C,2.5557319955,-4.0162079076
,4.7300492766\C,2.3907170166,-2.814689365,7.2031931194\N,1.4300560989,
-2.015824663,1.3175636019\N,2.6397866334,-4.1493278564,7.1411217031\C,
2.7773456381,-4.7206869261,5.9730902646\C,3.1265486793,-6.1400021794,5
.9642712502\H,2.7566709179,-4.4644606443,1.3464333346\C,3.5461863246,-
6.8078032686,7.051404485\C,3.8717342651,-8.2090437535,7.0585297462\H,3
.6478755193,-6.2627088995,7.986323811\H,3.2409094929,-5.6189601728,3.4
47611154\H,3.0306074153,-6.6872774246,5.0346618759\C,4.2939308184,-8.8
614689999,8.1489113708\C,4.6379034459,-10.3046631944,8.1997485127\H,4.
3958312173,-8.3004676718,9.0765798354\H,3.7630023809,-8.7526070569,6.1
21198437\H,4.0157254853,-10.8284719255,8.932994871\H,5.6750879696,-10.
4475136295,8.5206807791\H,4.5067393728,-10.7848644779,7.2286125654\\Ve
rsion=ES64L-G16RevC.01\State=1-A\HF=-1034.1214756\RMSD=8.679e-09\RMSF=
3.971e-07\Dipole=0.1062205,-0.4569529,-1.9968987\Quadrupole=-16.654172
,4.6296309,12.0245411,-9.1578614,4.2458448,-1.8312563\PG=C01 [X(C24H18
N2)]\\@

3h [R = Ph]

1\1\GINC-N1114\FOpt\RPBE1PBE\def2TZVP\C25H16N2\KA_PT6974\21-May-2023\0
\\# opt=tight pbe1pbe/def2tzvp scrf=(cpcm,solvent=dichloromethane) emp
iricaldispersion=gd3bj\\Title Card Required\\0,1\H,0.0484802628,0.0021
496963,0.0238311174\H,0.0325525685,-0.0129445466,5.0068836935\H,2.0276
62993,-0.0343311071,5.426643509\H,1.9644440333,0.9315351805,7.67179509
65\H,2.0499732057,-0.545664803,9.6696458488\H,2.3619581495,-2.99081718
56,9.3618185976\H,-1.2083950251,1.7183659153,1.3014095071\H,1.49021681
5,-2.305880676,0.3724823152\H,-1.2323447906,1.6641192185,3.769588383\C
,2.2413629497,-2.3287713763,8.5123530578\C,2.0826993774,-0.9750330166,
8.6750960267\C,2.016189931,-0.1439986315,7.5494343886\C,2.0309663198,-
0.6873823865,6.2870925664\C,2.1125985456,-2.0760973878,6.0915954151\C,
2.1092741136,-2.7060537372,4.7976278359\C,0.0852248825,-0.0056418211,3
.9276444578\C,-0.6427731659,0.9362273916,3.2249499265\C,-0.6373497975,
0.9620036521,1.8269677656\C,0.0664790145,0.016265113,1.107183168\C,0.7
792366483,-0.9392659827,1.8210359088\C,0.8497133634,-0.9524677302,3.23
59126341\C,1.6537548095,-2.1100758311,3.5943921932\C,1.938818335,-2.76
45771561,2.3770418487\C,2.5298824014,-4.0292144073,2.3064156477\C,2.81
23662617,-4.6628546944,3.4829078638\C,2.6208078885,-4.0271858049,4.732
181693\C,2.3070977852,-2.8956363991,7.2272873526\N,1.4644687434,-2.023
0413811,1.33685239\N,2.6380397004,-4.2185080053,7.1398406778\C,2.86982
20815,-4.7279105326,5.9648890144\H,3.1877247562,-5.676261105,3.4618267
774\H,2.6984570814,-4.511423963,1.3512300678\C,3.3775622461,-6.1232480
82,5.9671873249\C,2.6674763386,-7.1096379906,6.6468387268\C,4.58477793
07,-6.4578216342,5.3572770624\C,3.1439454705,-8.4102603715,6.699020372
3\H,1.7358217999,-6.8467930977,7.1340156921\C,5.0684840337,-7.75604816
28,5.4215651347\H,5.1581787112,-5.6931001724,4.8455566498\C,4.34617131
52,-8.7371345967,6.0862303798\H,2.5759141081,-9.1710488041,7.222032546
9\H,6.0148135154,-8.0003186937,4.9529363306\H,4.7213817513,-9.75299422
16,6.1304847607\\Version=ES64L-G16RevC.01\State=1-A\HF=-1071.0303376\R
MSD=5.000e-09\RMSF=6.358e-07\Dipole=-0.1244268,0.156115,-2.3156006\Qua
drupole=-10.3894538,1.9653002,8.4241536,-6.2804882,-1.5673635,2.395816
7\PG=C01 [X(C25H16N2)]\\@

3i [R = $MeOC_6H_4$]

1\1\GINC-N0502\FOpt\RPBE1PBE\def2TZVP\C26H18N2O1\KA_PT6974\21-May-2023
\0\\# opt=tight pbe1pbe/def2tzvp scrf=(cpcm,solvent=dichloromethane) e
mpiricaldispersion=gd3bj\\Title Card Required\\0,1\H,0.0555695395,0.01
02755688,0.0270705372\H,0.0378839189,-0.0158773567,5.0099371509\H,2.03
12587121,-0.0420831737,5.4341752966\H,1.9465648487,0.9133767517,7.6836

214939\H,2.0116525047,-0.5742920649,9.6747217949\H,2.3299018961,-3.017
3003345,9.357816126\H,-1.2016342561,1.7245035339,1.3082560916\H,1.4951
892983,-2.2963425317,0.3695466982\H,-1.2259407741,1.6641583686,3.77629
49165\C,2.215997134,-2.3513804002,8.5103539418\C,2.0545286795,-0.99852
8222,8.6783123551\C,1.9994045741,-0.1615462966,7.5564237024\C,2.026625
1899,-0.6990974323,6.2916654118\C,2.1096734763,-2.0867536363,6.0904046
676\C,2.1137954898,-2.7118950256,4.7940763645\C,0.0909401575,-0.006499
8494,3.9307522085\C,-0.6365435675,0.937360809,3.2299814081\C,-0.630899
4507,0.9667029086,1.8320997744\C,0.0729711636,0.0224224075,1.110458815
8\C,0.7850724062,-0.934931647,1.8223770408\C,0.8553019975,-0.952265927
4,3.2372715558\C,1.6589402492,-2.1115972442,3.592430641\C,1.9428513935
,-2.7627656052,2.3731385058\C,2.5286590731,-4.0293933001,2.2993061881\
C,2.8113567635,-4.6658604931,3.4743701248\C,2.6261714029,-4.0324765514
,4.7256941525\C,2.2958614425,-2.9121757229,7.223111010\N,1.4693417675
,-2.0175649633,1.3349764182\N,2.636948029,-4.2317003331,7.1323108506\C
,2.8796161219,-4.738006051,5.9568974367\H,3.1790255818,-5.6818500553,3
.4500312312\H,2.6911866796,-4.5116013141,1.3429599855\C,3.3996549208,-
6.1254652496,5.955862161\C,2.7406052986,-7.1099953286,6.6812056012\C,4
.5851989489,-6.470764528,5.3036159628\C,3.2207803187,-8.4100093457,6.7
443232058\C,5.0850029814,-7.7554013398,5.3692825909\C,4.4016933387,-8.
7390222283,6.0843417166\H,2.6714297372,-9.151690788,7.3080180011\H,6.0
125652212,-8.017725206,4.8744685328\H,1.8282400087,-6.8539346913,7.206
9841402\H,5.1379910781,-5.71649546,4.7552660334\O,4.9569918849,-9.9692
944779,6.0845517597\H,3.2952353611,-11.1720262312,6.414339677\C,4.3000
634011,-10.9902217541,6.8072468451\H,4.9035312599,-11.8866938627,6.681
4417117\H,4.2337737716,-10.7445048523,7.8713297695\\Version=ES64L-G16R
evC.01\State=1-A\HF=-1185.4802953\RMSD=3.205e-09\RMSF=4.062e-07\Dipole
=-0.4341565,-0.4187823,-1.9314022\Quadrupole=-17.4764909,9.3157284,8.1
607625,-3.6391906,0.0970694,-6.4919055\PG=C01 [X(C26H18N2O1)]\\@

3j [R = $F_3CC_6H_4$]

1\1\GINC-N1105\FOpt\RPBE1PBE\def2TZVP\C26H15F3N2\KA_PT6974\21-May-2023
\0\\# opt=tight pbe1pbe/def2tzvp scrf=(cpcm,solvent=dichloromethane) e
mpiricaldispersion=gd3bj\\Title Card Required\\0,1\H,0.0579775523,0.00
05411386,0.0229957295\H,0.0297980705,-0.0208031948,5.0062343395\H,2.02
31904925,-0.0363496924,5.4317331816\H,1.9526835162,0.923730788,7.67891
236\H,2.0437187051,-0.5566372863,9.6736714881\H,2.3681197511,-2.999661
7506,9.3615548087\H,-1.2112091285,1.708975092,1.3002249739\H,1.5154618
76,-2.295180301,0.3707058664\H,-1.2402064398,1.651339837,3.7680582217\
C,2.2453680858,-2.3365135782,8.5133366217\C,2.0796581984,-0.984028257,
8.6784460957\C,2.0100777145,-0.1512263192,7.5542321301\C,2.029029067,-
0.6913408823,6.2906357481\C,2.1183965755,-2.0791269437,6.0921581771\C,
2.1200619346,-2.7051894256,4.7963044862\C,0.0853851168,-0.0118292227,3
.9271591308\C,-0.6455625217,0.9272532439,3.2239081524\C,-0.6370943391,
0.9550372737,1.8259024528\C,0.0734525233,0.0142327892,1.1064349224\C,0
.7897432084,-0.9380961308,1.820849755\C,0.8563020866,-0.9537570626,3.2
357758536\C,1.6648117772,-2.1083236618,3.5938124258\C,1.9568750644,-2.
7585305279,2.3755565905\C,2.5546434993,-4.0199249263,2.3025444977\C,2.
8376019169,-4.6556813108,3.4775226059\C,2.6384195376,-4.0238553765,4.7
277000653\C,2.314948699,-2.8996597051,7.2270085574\N,1.48172042,-2.017
3537623,1.3362704616\N,2.6500631025,-4.2213024402,7.1362108282\C,2.884
8692545,-4.7234958201,5.9595138914\H,3.2174318816,-5.6674020449,3.4522
775652\H,2.7270987831,-4.4979422327,1.345927062\C,3.3982533857,-6.1171
576441,5.9630636595\C,2.6877130778,-7.1057400398,6.6380260942\C,4.6108
844956,-6.4450893135,5.3620332146\C,3.1662423374,-8.4024961648,6.69547
81176\C,5.1013742475,-7.7380620342,5.4266143743\C,4.3753192946,-8.7175
239741,6.0894354646\H,2.6029488741,-9.1660903194,7.2172926541\H,6.0521
917072,-7.9798609083,4.968625928\C,4.8733947158,-10.129637471,6.111844
8505\H,1.7532209872,-6.8490608302,7.121438351\H,5.1870309301,-5.679460
2389,4.8563750181\F,4.496833152,-10.7840335194,7.2188750638\F,6.209680
3371,-10.1997193973,6.046877575\F,4.4023488557,-10.841270972,5.0694706
787\\Version=ES64L-G16RevC.01\State=1-A\HF=-1407.9138415\RMSD=1.951e-0

```
9\RMSF=4.437e-07\Dipole=-0.6256086,1.5947005,-2.35803\Quadrupole=-5.61
35036,-12.294134,17.9076375,1.6911097,-0.8854996,-0.0512159\PG=C01 [X(
C26H15F3N2)]\\@
```

3k [R = mesityl]

```
1\1\GINC-N1313\FOpt\RPBE1PBE\def2TZVP\C28H22N2\KA_PT6974\21-May-2023\0
\\# opt=tight pbe1pbe/def2tzvp scrf=(cpcm,solvent=dichloromethane) emp
iricaldispersion=gd3bj\\Title Card Required\\0,1\H,-0.0005767943,-0.06
54440097,0.0301093402\H,0.0347473264,-0.0280527038,5.0137652947\H,2.01
96295536,-0.0345985989,5.4149777043\H,2.0940483036,0.9635148436,7.6433
520167\H,2.3441469386,-0.4809110487,9.6516902157\H,2.6531514876,-2.928
6827093,9.3583207198\H,-1.2572428397,1.6551199504,1.3031225388\H,1.474
1498646,-2.3500598225,0.3869613883\H,-1.2542436573,1.6280099,3.7714952
484\C,2.4721268477,-2.2797584342,8.5093719667\C,2.3118861897,-0.924995
9078,8.6635989233\C,2.1534484411,-0.1130158913,7.5337210408\C,2.088877
789,-0.6756097294,6.281444801\C,2.1788271044,-2.0660408771,6.098578847
3\C,2.1268940352,-2.7077670932,4.8108714058\C,0.0765540638,-0.03098961
73,3.9338214098\C,-0.6651128004,0.8983321625,3.2287851133\C,-0.6747639
451,0.9087446543,1.8304176797\C,0.0285361593,-0.0392264432,1.113031995
2\C,0.7564441331,-0.9813577209,1.8293864797\C,0.8405057162,-0.97929026
28,3.2435275995\C,1.6543356834,-2.1273070601,3.6063770989\C,1.93750639
33,-2.7914374017,2.3929277424\C,2.560589427,-4.0422122757,2.324579544\
C,2.8541429186,-4.6648117005,3.5043583824\C,2.6349484323,-4.027366797,
4.7469200635\C,2.4517310891,-2.8671188504,7.2327181246\N,1.4458946756,
-2.0642304368,1.3502370199\N,2.7562184858,-4.199517581,7.1503589323\C,
2.8980314478,-4.7285812127,5.9721422689\H,3.2734093071,-5.6632063473,3
.5022716272\H,2.751932887,-4.5160441509,1.3693974181\C,3.3279162819,-6
.1543303957,5.9258622538\C,2.3691751182,-7.1688722535,5.8641132305\C,4
.6902085848,-6.4640025673,5.9592959288\C,2.7946313964,-8.4924880082,5.
8390921531\C,5.0756533029,-7.7993211049,5.9292959822\C,4.1430831292,-8
.828479134,5.8663506086\H,2.0501698238,-9.2821879963,5.7987510955\H,6.
1341369197,-8.0409356473,5.9579876674\H,0.6120997172,-6.2705657213,6.7
185073957\C,0.9069801579,-6.8404917242,5.8337108195\H,0.6563644553,-6.
2269355598,4.9637313793\H,0.3048163394,-7.7484327806,5.7939062336\H,3.
8244014306,-10.9255027515,6.222678838\C,4.5811737151,-10.2601349659,5.
8036891329\H,4.751566949,-10.5697306301,4.767794399\H,5.5156235083,-10
.4135605637,6.3464845689\H,5.6169144536,-4.7975242162,6.9511484592\C,5
.7191153709,-5.3763020148,6.0296088845\H,5.6093617735,-4.6717679864,5.
2002828327\H,6.7270634892,-5.7902160595,5.9938462292\\Version=ES64L-G1
6RevC.01\State=1-A\HF=-1188.8862949\RMSD=3.392e-09\RMSF=3.483e-07\Dipo
le=-0.1689521,0.1676946,-2.3701741\Quadrupole=-8.9071998,1.7982024,7.1
089974,-5.395196,2.2362343,2.0054623\PG=C01 [X(C28H22N2)]\\@
```

3l [R = 3-pyridinyl]

```
1\1\GINC-N1039\FOpt\RPBE1PBE\def2TZVP\C24H15N3\KA_PT6974\21-May-2023\0
\\# opt=tight pbe1pbe/def2tzvp scrf=(cpcm,solvent=dichloromethane) emp
iricaldispersion=gd3bj\\Title Card Required\\0,1\H,0.0605176042,-0.036
1036266,-0.0224348283\H,0.0639035935,0.1470148608,4.9573922165\H,2.052
3865,-0.0137695364,5.374084761\H,2.0784904408,1.0425838111,7.578437350
1\H,2.0635548359,-0.3550233929,9.634153585\H,2.1851144764,-2.827515673
8,9.4246379301\H,-1.0523738732,1.8225889019,1.1873670236\H,1.319129162
6,-2.4341622896,0.4158782741\H,-1.0704328868,1.8679173272,3.6556878536
\C,2.1099323714,-2.1926207595,8.5496693688\C,2.0566054371,-0.825361215
1,8.6578472821\C,2.0466407525,-0.0377557301,7.4994970946\C,2.011192114
2,-0.6303580626,6.2599922188\C,1.9844718365,-2.0279023908,6.1203600948
\C,1.9237481796,-2.7061702721,4.852365239\C,0.1129058294,0.107650652,3
.878689221\C,-0.5418380162,1.0751295007,3.1398592567\C,-0.5401459904,1
.0451146106,1.7419047547\C,0.0842428592,0.0194252915,1.0594482485\C,0.
7228931232,-0.9600477585,1.8100359449\C,0.7980427488,-0.9227019945,3.2
241244754\C,1.5103094497,-2.1246996472,3.627348553\C,1.7380031656,-2.8
47321214,2.436428404\C,2.2277812909,-4.1562986961,2.4149292027\C,2.46
51744208,-4.7633659641,3.6150045421\C,2.3296705563,-4.0651889863,4.837
```

8667732\C,2.1229260657,-2.8131804615,7.2881975984\N,1.3188139488,-2.11
27477048,1.3684976149\N,2.3511524815,-4.1599886552,7.2526207382\C,2.53
18596285,-4.7300800071,6.0970292627\H,2.7564934913,-5.8041711813,3.630
3642281\H,2.3531829045,-4.687582098,1.479416499\C,2.9273858562,-6.1573
94183,6.1579828352\C,2.1548729844,-7.0545378145,6.8924640194\C,4.08562
08187,-6.6443781629,5.5635402863\C,4.4113252067,-7.9805297172,5.721816
7734\C,3.5638739802,-8.7872138295,6.4646715234\H,5.3112889653,-8.39152
78818,5.2817779026\H,1.2493812944,-6.7003556218,7.3763570812\H,4.73324
25908,-5.9821847696,5.0002643478\N,2.4515968904,-8.3395582335,7.043361
6589\H,3.7912876217,-9.8399566957,6.6041836642\\Version=ES64L-G16RevC.
01\State=1-A\HF=-1087.0615975\RMSD=5.305e-09\RMSF=5.835e-07\Dipole=0.4
805815,0.9362305,-2.9447737\Quadrupole=-4.4529404,-5.0738647,9.5268051
,-10.9702182,0.6640031,11.4907298\PG=C01 [X(C24H15N3)]\\@

3m [R = 2-pyridinyl]

1\1\GINC-N0320\FOpt\RPBE1PBE\def2TZVP\C24H15N3\KA_PT6974\11-Sep-2024\0
\\# opt=tight pbe1pbe/def2tzvp scrf=(cpcm,solvent=dichloromethane) emp
iricaldispersion=gd3bj\\Title Card Required\\0,1\H,-0.0412555299,-0.06
63290925,0.0072178805\H,-0.0111380908,-0.0230443952,4.9904860402\H,1.9
823631295,-0.0072411919,5.3905370859\H,1.977303021,0.9897787849,7.6213
256114\H,2.1648510579,-0.4540085874,9.6363768733\H,2.5072871163,-2.898
529027,9.35328916\H,-1.3146201682,1.6435718916,1.2766809879\H,1.447024
677,-2.3431750468,0.3692221188\H,-1.3147478117,1.6187161727,3.74523836
31\C,2.3473267184,-2.2509641156,8.4992570246\C,2.1701383477,-0.8980600
255,8.6478174879\C,2.0464048062,-0.0862850638,7.5130015388\C,2.0274605
983,-0.6478681286,6.25895237\C,2.1307871373,-2.0376759305,6.0796104635
\C,2.1082744311,-2.6819131611,4.7927505262\C,0.0318507261,-0.026952350
7,3.9107262781\C,-0.7179795341,0.8942872757,3.2038665303\C,-0.72592952
9,0.9032461501,1.8055738111\C,-0.0131592188,-0.0389909045,1.0900830023
\C,0.7224476438,-0.9736582447,1.8083738557\C,0.8055712258,-0.968777934
1,3.2224861468\C,1.6311178728,-2.1083802575,3.5873026152\C,1.918293457
4,-2.7723029564,2.3750209242\C,2.5386896443,-4.0239159493,2.3113119658
\C,2.8399536613,-4.6410348943,3.4916523991\C,2.636271014,-3.996450972,
4.7343882057\C,2.375511879,-2.8363362429,7.2214911433\N,1.4219057454,-
2.0513657852,1.3309445866\N,2.7116401722,-4.1596163685,7.1440027376\C,
2.9018166288,-4.6778624411,5.9690346957\H,3.2458905511,-5.6436010749,3
.4789094739\H,2.7174212803,-4.5078740593,1.3589177375\C,3.405845587,-6
.0831836446,5.9658385759\C,4.6838132478,-6.3731455704,5.4990335089\C,5
.1360943318,-7.6799734303,5.5626169976\C,4.2940562378,-8.651359631,6.0
789404603\H,6.130727662,-7.9344459863,5.2157661653\H,5.3104571506,-5.5
827783153,5.1038841399\H,4.6013909202,-9.687401704,6.1460788971\C,3.03
59040774,-8.2646937032,6.514141209\H,2.3483317982,-9.0000622924,6.9221
007861\N,2.5929572388,-7.0115288364,6.4673800223\\Version=ES64L-G16Rev
C.01\State=1-A\HF=-1087.0605997\RMSD=5.300e-09\RMSF=8.656e-07\Dipole=0
.7868485,0.0301124,-2.7209178\Quadrupole=-8.6850503,4.4714166,4.213633
7,-13.6431295,1.6138782,5.0637036\PG=C01 [X(C24H15N3)]\\@

3n [R = 1-naphthalenyl]

1\1\GINC-N0704\FOpt\RPBE1PBE\def2TZVP\C29H18N2\KA_PT6974\16-Sep-2024\0
\\# opt pbe1pbe/def2tzvp scrf=(cpcm,solvent=dichloromethane) empirical
dispersion=gd3bj\\Title Card Required\\0,1\H,-0.8768782608,-0.84879435
71,0.3765617075\H,0.0846330514,0.0069514605,5.1918840741\H,2.112431024
1,0.0773714961,5.1824190663\H,2.6179002625,1.4482735428,7.1387468622\H
,3.3323523213,0.3769691723,9.2656763913\H,3.6482125974,-2.0864554765,9
.3403783357\H,-1.9318753342,1.0529728651,1.5705918326\H,0.7226060897,-
3.0398278367,0.8110415529\H,-1.4693761387,1.4324785651,3.965424729\C,3
.2749270247,-1.5947138119,8.4497288658\C,3.1079638257,-0.2331330059,8.
398535528\C,2.6888374798,0.3689632624,7.2056998661\C,2.3818721909,-0.4
033611811,6.1111532545\C,2.4781839125,-1.8047420424,6.1545581318\C,2.1
921078118,-2.6595119766,5.0319825748\C,-0.0757341726,-0.173043454,4.13
82555649\C,-0.9667694627,0.6252806658,3.4459399354\C,-1.2364437829,0.4
053126875,2.091458807\C,-0.6470560408,-0.6455202543,1.4158831653\C,0.2

333322059,-1.4547847038,2.123626745\C,0.5785730554,-1.2190592614,3.477
2539783\C,1.4838453445,-2.2887705752,3.8605835399\C,1.5608456348,-3.14
23467958,2.7390662803\C,2.212233994,-4.3804930251,2.7559608627\C,2.736
7708574,-4.8001413769,3.94472355\C,2.7172230733,-3.9729486261,5.091507
0722\C,3.0050266436,-2.3960200814,7.3270717366\N,0.8591025057,-2.59983
51705,1.7045627271\N,3.313038265,-3.7264177822,7.4180604442\C,3.218626
6902,-4.4573446236,6.3476682101\H,3.1917861662,-5.7793771869,4.0165568
278\H,2.2515061079,-5.0005313881,1.8686886139\C,3.6555444398,-5.874010
5085,6.4775591012\C,4.9883156337,-6.1445194011,6.6590447151\C,3.189477
7391,-8.277504052,6.5586292784\C,5.452482899,-7.4667026678,6.779404304
\H,5.6928811171,-5.3217547799,6.7008352209\C,4.5723786959,-8.511322945
,6.7245245963\H,6.5122508775,-7.6498950327,6.9124365767\H,4.9217155936
,-9.5344160011,6.8130162362\H,2.6324256586,-10.3576837098,6.609773164\
C,2.2610710581,-9.3424718696,6.5187959733\C,0.9226890006,-9.1021676957
,6.3735273502\C,2.7158808244,-6.9406876841,6.4359231535\C,0.4512440107
,-7.7793417428,6.2676471499\H,0.2204605327,-9.9272022675,6.3450483548\
C,1.3246363887,-6.7265229479,6.2993532773\H,-0.6118440142,-7.595641927
5,6.1635665751\H,0.9509517287,-5.7126627821,6.2215583041\\Version=ES64
L-G16RevC.01\State=1-A\HF=-1224.5556377\RMSD=5.190e-09\RMSF=2.149e-06\
Dipole=-0.6515011,-0.2334815,-2.2994919\Quadrupole=-6.361973,4.6678491
,1.6941239,-4.2465256,6.2875057,2.8671514\PG=C01 [X(C29H18N2)]\\@

10

1\1\GINC-N1214\FOpt\RPBE1PBE\def2TZVP\C26H19N2(1+)\KA_PT6974\03-Oct-20
24\0\\# opt pbe1pbe scrf=(cpcm,solvent=dichloromethane) def2tzvp empir
icaldispersion=gd3bj\\Title Card Required\\1,1\H,-0.2878042534,-0.2419
231994,0.0891252698\H,-0.0572158343,-0.0706634237,5.0678529808\H,2.024
903237,-0.0121733172,5.2768449134\H,2.2172719891,1.134281601,7.4336356
32\H,2.5703297181,-0.1899010124,9.5077933268\H,2.8362688308,-2.6060667
572,9.3884430519\H,-1.550716256,1.4669438623,1.3698210189\H,1.31676559
56,-2.4450893536,0.4380121462\H,-1.4501071744,1.5080928996,3.833513800
2\C,2.6166381779,-2.0553388342,8.4863071205\C,2.4780837121,-0.6885399,
8.5510182175\C,2.2670328694,0.0535296536,7.3886067552\C,2.1391280999,-
0.589047288,6.1820681111\C,2.213345421,-1.9862870279,6.0855537181\C,2.
1056315218,-2.6796183111,4.8302227701\C,-0.0612819954,-0.1027187767,3.
9873368014\C,-0.8594341685,0.782309381,3.2878677619\C,-0.9234171232,0.
7541743634,1.8915526354\C,-0.2171156264,-0.1876111965,1.1686026054\C,0
.5695251886,-1.0823118193,1.880464983\C,0.7028861893,-1.0413048143,3.2
869592965\C,1.5667168215,-2.1534071636,3.6440110641\C,1.8403807129,-2.
834015197,2.4303569933\C,2.5439513344,-4.0444508897,2.3648226171\C,2.9
048409997,-4.6303466118,3.5394453679\C,2.6633444557,-3.9870502403,4.78
22369678\C,2.5188471071,-2.7103029434,7.2539040652\N,1.2839563579,-2.1
563492265,1.4016414886\N,2.7664719685,-4.0772101624,7.1671672702\C,2.9
306152448,-4.6713850848,5.986851755\H,3.3913423166,-5.5952959612,3.533
5812597\H,2.7482214375,-4.5178549039,1.4129610851\C,3.3709447423,-6.08
18185573,5.9570005621\C,2.4910184371,-7.0803802554,5.5495586471\C,4.67
8328856,-6.4051677063,6.3107772252\C,2.9156911102,-8.3990689988,5.5107
361067\H,1.4755051411,-6.8230111514,5.2724213381\C,5.1005931114,-7.723
3466892,6.2548925089\H,5.363915239,-5.6244532864,6.6193620493\C,4.2195
443137,-8.7207867987,5.8598021487\H,2.2262099962,-9.1756966447,5.20285
54586\H,6.1207823463,-7.9715316318,6.5213326933\H,4.5512067544,-9.7515
158505,5.8221814336\C,2.8575123957,-4.8369966452,8.4137103768\H,3.7925
821627,-4.6165780816,8.9284390064\H,2.0149039333,-4.5678237377,9.04711
94675\H,2.8115576775,-5.8961027946,8.1908612575\\Version=ES64L-G16RevC
.01\State=1-A\HF=-1110.750273\RMSD=3.113e-09\RMSF=8.764e-06\Dipole=0.7
053337,-1.2741225,0.6446819\Quadrupole=-22.1988819,4.8049139,17.393968
,-13.6023387,11.3792126,-4.3426655\PG=C01 [X(C26H19N2)]\\@

19’

1\1\GINC-N0812\FOpt\RPBE1PBE\def2TZVP\C31H27B1N2O2\KA_PT6974\04-Oct-20
24\0\\# opt=tight pbe1pbe/def2tzvp scrf=(cpcm,solvent=dichloromethane)
empiricaldispersion=gd3bj\\Title\\0,1\B,-2.5913630079,0.0762256125,-0

.007294318\C,-0.1715888127,-0.9142825019,-0.995640395\C,-0.4966380515,
1.3326121174,-0.4388512079\C,1.230325781,-0.7550206516,-0.9942364538\C
,0.9037584372,1.555507551,-0.2880002529\C,1.782317985,0.4453489731,-0.
4232397956\N,-0.985854273,0.1120432173,-0.5903124404\C,-1.5938014922,2
.3166950928,-0.3906234229\C,-1.5450038984,3.6844351635,-0.6505735012\C
,-2.8137116482,1.6610229687,-0.1852891825\C,-2.7263914124,4.4126018577
,-0.6436470741\H,-0.6255805371,4.1891739508,-0.9129614675\C,-3.9790678
623,2.4088898349,-0.1733314028\C,-3.9374105528,3.7825175938,-0.3908583
41\H,-2.6999668349,5.4753557352,-0.854080724\H,-4.9374163455,1.9252987
,-0.0170976585\H,-4.8545811233,4.3613465907,-0.3892528018\O,-2.5623771
68,-0.3344327536,1.3729951906\O,-3.4065944492,-0.8648149201,-0.7121515
851\C,-4.1260008961,-1.6471695859,0.2398327888\C,-3.2206146196,-1.5865
066274,1.5146736278\C,-2.1815507955,-2.6996169604,1.5762984864\H,-1.48
75550942,-2.4803855053,2.390843129\H,-2.6437228886,-3.6691375887,1.774
9684718\H,-1.6052703946,-2.7742745972,0.6554051415\C,-3.9930921547,-1.
5808681879,2.8226775733\H,-4.5873596278,-2.4918682364,2.929387566\H,-3
.2906356053,-1.5378515964,3.6580857472\H,-4.6572944451,-0.720127058,2.
892289284\C,-4.3311689608,-3.0427396778,-0.317431277\H,-4.8374951356,-
3.678679819,0.4127155913\H,-4.9565327163,-2.992284557,-1.211610046\H,-
3.3868096965,-3.5156942973,-0.5860504863\C,-5.4857758589,-0.9957180753
,0.4611287401\H,-5.9828840401,-0.8853210559,-0.5047875963\H,-6.1237306
489,-1.6000388986,1.1090903269\H,-5.3816080417,-0.0065350493,0.9086953
848\C,-0.7445972217,-2.074296321,-1.5385785527\H,-1.8223206181,-2.1501
856837,-1.5461180892\C,2.0082090566,-1.725992136,-1.6421595635\H,3.069
029962,-1.5559100494,-1.754864329\C,3.1266026286,0.6059428596,-0.02547
58561\C,1.404430305,2.8166090658,0.1159992627\H,0.7194426734,3.6319064
555,0.2814433181\C,1.4365714073,-2.8509503365,-2.1860478021\H,2.056469
3638,-3.5800285205,-2.6938628365\C,0.052426152,-3.0356477457,-2.110563
1326\H,-0.4015258539,-3.9181531377,-2.5459585971\C,3.5799778608,1.9058
981925,0.2936411601\C,2.7321813678,3.0153838395,0.3602503903\H,3.10718
60478,3.9839981318,0.666834039\C,4.2529592914,-0.27683137,0.2324113627
\H,5.4435857459,2.6483308711,0.9219487798\C,5.3314418705,0.557833669,0
.6101278773\N,4.8996571676,1.8610746423,0.6125515984\C,4.4485282121,-1
.6602114394,0.3033981648\H,3.6416210503,-2.3488508958,0.0971145564\C,6
.5818791201,0.0663370147,0.9616454173\H,7.3882127949,0.7374300398,1.23
24126475\C,5.6846005539,-2.1582810033,0.6703978343\H,5.8287986795,-3.2
307153485,0.7242944589\C,6.7484229741,-1.3049082855,0.978305894\H,7.70
96272948,-1.7232332671,1.2527053782\\Version=ES64L-G16RevC.01\State=1-
A\HF=-1481.4204095\RMSD=9.996e-09\RMSF=6.852e-07\Dipole=3.3906423,0.94
46683,0.0905336\Quadrupole=11.6954395,10.1467843,-21.8422238,12.611742
,4.1713243,3.9435582\PG=C01 [X(C31H27B1N2O2)]\\@

6 [(*M*)-enantiomer]

1\1\GINC-N1220\FOpt\RPBE1PBE\def2TZVP\C25H15Br1N2\KA_PT6974\24-Jan-202
4\0\\# opt=tight pbe1pbe/def2tzvp scrf=(cpcm,solvent=dichloromethane)
empiricaldispersion=gd3bj\\Title Card Required\\0,1\H,3.2073815242,5.2
316578107,-0.1039916719\H,2.9220934729,0.4066293044,1.1074341867\H,2.2
268691078,-0.4014298492,-0.626128851\H,-0.8853819764,-3.9137019852,0.9
022162783\H,5.1671688556,4.0310147311,0.8325352377\H,0.5428053456,4.89
3583915,-0.6507097577\H,4.9856355523,1.6497219415,1.4591674359\C,-0.17
98667578,-3.1455568284,0.6089426974\C,1.1405807575,-3.4489762865,0.394
3960562\C,1.9930142779,-2.4376688443,-0.0596114634\C,1.5587823299,-1.1
472967252,-0.2240514093\C,0.2296135042,-0.8068472249,0.0717409479\C,-0
.289725586,0.529841999,-0.0440783101\C,2.9703325356,1.4400396257,0.795
0371647\C,4.1365421434,2.1501584443,1.0091976116\C,4.2371828941,3.5011
747139,0.6631350403\C,3.1549101824,4.1756222499,0.1328454865\C,1.98059
43205,3.4591902829,-0.0621089606\C,1.8654584097,2.0746309483,0.2145929
752\C,0.4903332618,1.7116525806,-0.0891418166\C,-0.1515019682,2.917825
3216,-0.443355936\C,-1.5333758299,3.0266130281,-0.6224549736\C,-2.2907
262009,1.9091101578,-0.4144009487\C,-1.6992817686,0.6549055136,-0.1344
211484\C,-0.6697827867,-1.8428851805,0.4095972406\N,0.7615593515,3.927
6715737,-0.478189769\N,-2.0201228767,-1.6596319405,0.4792243985\C,-2.5

107739426,-0.5027706277,0.1377540067\H,-3.3680813988,1.9830376226,-0.4
597930767\H,-1.9890795733,3.9807575483,-0.8576745825\C,-3.9923946027,-
0.4326152595,0.0860035971\C,-4.7354886149,-0.8232882851,1.1969740628\C
,-4.6606662495,-0.0446275477,-1.0733607222\C,-6.1208784635,-0.80707109
35,1.1574298696\H,-4.215693006,-1.138973436,2.0939186347\C,-6.04679764
54,-0.0425533365,-1.1173827009\H,-4.0924360455,0.2384253797,-1.9522002
242\C,-6.780415607,-0.4167446987,-0.0003222785\H,-6.687847521,-1.10273
03797,2.0326752889\H,-6.5545908992,0.2497353567,-2.0292194184\H,-7.863
7024612,-0.4084184359,-0.0332594498\Br,3.7886164486,-2.8680750776,-0.4
741246667\H,1.5109137161,-4.4560085966,0.5356020791\\Version=ES64L-G16
RevC.01\State=1-A\HF=-3644.2948658\RMSD=9.471e-09\RMSF=6.757e-07\Dipol
e=-0.5677276,2.6249701,-0.3069279\Quadrupole=-0.9445462,11.7813709,-10
.8368247,0.1869021,3.3414089,-6.9576027\PG=C01 [X(C25H15Br1N2)]\\@

7.2.4 Indolo[3,2-*a*]phenanthridines

9 [R = H, (*M*)-enantiomer]

1\1\GINC-N1520\FOpt\RPBE1PBE\def2TZVP\C19H12N2\KA_PT6974\06-Aug-2024\0
\\# opt pbe1pbe/def2tzvp scrf=(cpcm,solvent=dichloromethane) empirical
dispersion=gd3bj\\Title Card Required\\0,1\H,6.3791337185,0.3779929014
,0.5346616821\H,2.4264702839,-2.6546673525,0.6524444415\H,1.5924689995
,-2.3365107452,-1.1625169391\H,0.3307877118,-4.3834415582,-1.571225920
5\H,-2.1236249421,-4.4490481121,-1.2090151026\H,-3.3115405789,-2.37538
41276,-0.558009819\H,6.6352606191,-2.0331941492,1.0608287992\H,4.45839
48251,2.2861584366,0.0890919125\H,4.6559117359,-3.5064709664,1.1498056
986\C,-2.2309142289,-2.3890305778,-0.6522915853\C,-1.5731150281,-3.534
3523287,-1.0244477556\C,-0.1844475244,-3.5002068149,-1.2115529088\C,0.
5319698599,-2.3543221492,-0.9607480963\C,-0.1058242393,-1.1865424183,-
0.5071748454\C,0.5731837333,0.0543489336,-0.2373922711\C,3.272922018,-
1.9867776768,0.5770673882\C,4.5350319903,-2.4656342559,0.8738231236\C,
5.6556744206,-1.6294958133,0.8329474725\C,5.5242284575,-0.287953273,0.
5324184252\C,4.2492999505,0.1905902187,0.2531928705\C,3.1033907536,-0.
6434042394,0.2205692406\C,1.974207171,0.2228301611,-0.0699729479\C,2.4
997114693,1.5306699573,-0.1104841306\C,1.7034619322,2.6777651758,-0.19
70743309\C,0.3482113883,2.5062155484,-0.1891262308\C,-0.2342868969,1.2
186054126,-0.1903836797\C,-1.516204266,-1.2022088554,-0.4215516502\N,3
.8585491818,1.4821828989,0.0255506285\N,-1.6052586044,1.1783657101,-0.
1441534809\C,-2.1950183917,0.0287481848,-0.1939745935\H,-3.2806939019,
0.0164243186,-0.1110909303\H,-0.3245708176,3.3550914693,-0.1895744315\
H,2.1504968903,3.6644636868,-0.2177554831\\Version=ES64L-G16RevC.01\St
ate=1-A\HF=-840.1553353\RMSD=9.935e-09\RMSF=7.543e-06\Dipole=1.2936395
,-0.1511658,-0.0912798\Quadrupole=5.1805817,6.987662,-12.1682437,14.48
56411,2.0554721,1.6480253\PG=C01 [X(C19H12N2)]\\@

9 [R = H, transition state]

1\1\GINC-N0713\SP\RPBE1PBE\def2TZVP\C19H12N2\KA_PT6974\28-Oct-2024\0\\
# sp scrf=(solvent=dichloromethane) def2tzvp pbe1pbe empiricaldispersi
on=gd3bj\\Title Card Required\\0,1\H,0,-5.174209,0.304131,0.000021\H,0
,-1.127734,-2.553612,-0.000022\H,0,0.312815,-2.105757,-0.000015\H,0,1.
971268,-3.861579,-0.000009\H,0,4.398264,-3.30228,0.000009\H,0,5.075098
,-0.914762,0.000017\H,0,-5.33895,-2.182919,0.000012\H,0,-3.44227,2.324
232,0.000016\H,0,-3.261855,-3.534033,-0.000011\C,0,4.027377,-1.196012,
0.00001\C,0,3.654225,-2.514916,0.000006\C,0,2.290804,-2.825763,-0.0000
04\C,0,1.343374,-1.830628,-0.000008\C,0,1.677003,-0.469326,-0.000003\C
,0,0.7228,0.622967,-0.000005\C,0,-1.950714,-1.862055,-0.000011\C,0,-3.
200878,-2.452257,-0.000005\C,0,-4.371823,-1.694517,0.000007\C,0,-4.288
448,-0.320574,0.000013\C,0,-3.024806,0.258692,0.000006\C,0,-1.797139,-
0.462671,-0.000003\C,0,-0.720875,0.55066,-0.000005\C,0,-1.412155,1.789
102,-0.000002\C,0,-0.821274,3.053272,-0.000007\C,0,0.534676,3.104941,-
0.000007\C,0,1.308526,1.924971,-0.000004\C,0,3.063735,-0.174678,0.0000

05\N,0,-2.75576,1.590486,0.000006\N,0,2.658614,2.173058,0.000003\C,0,3
.479787,1.180138,0.000007\H,0,-1.435042,3.946194,-0.00001\H,0,1.071685
,4.044731,-0.000009\H,0,4.545774,1.402771,0.000013\\Version=ES64L-G16R
evC.01\State=1-A\HF=-840.1462069\RMSD=2.929e-09\Dipole=-1.2409208,-0.2
855974,0.0000069\Quadrupole=10.5722073,3.2980298,-13.8702371,-13.64443
85,-0.0000471,0.0000589\PG=C01 [X(C19H12N2)]\\@

9 [R = H, S$_1$]

1\1\GINC-N0703\FOpt\RPBE1PBE TDA-FC\def2TZVP\C19H12N2\KA_PT6974\16-Oct
-2024\0\\# opt def2tzvp pbe1pbe TDA=(singlet,root=1,nstates=50) scrf=(
cpcm,solvent=dichloromethane) empiricaldispersion=gd3bj\\Title Card Re
quired\\0,1\H,-5.1171612196,0.0339140263,0.248562565\H,-0.7970692851,-
2.2502477982,-0.7819441998\H,0.246717898,-1.8722701673,1.0370166803\H,
1.874754527,-3.6893462296,1.1138731219\H,4.2029671577,-3.3499633975,0.
3220855607\H,4.907156372,-1.0925403499,-0.4527671856\H,-5.0632845807,-
2.3810602675,-0.3899954949\H,-3.4826020909,2.2159484847,0.5323178112\H
,-2.9210352588,-3.4698998028,-0.9268586849\C,3.8786767019,-1.281105963
3,-0.1629275221\C,3.4894763715,-2.5366680238,0.2778999154\C,2.17163158
56,-2.727876958,0.7099065662\C,1.2410312413,-1.7085323701,0.6469693567
\C,1.588306455,-0.4402329488,0.1315045655\C,0.6993077593,0.6586022483,
0.0090533819\C,-1.7163767836,-1.7474167931,-0.5165971449\C,-2.92926572
86,-2.4337102589,-0.6097802581\C,-4.139741855,-1.820797632,-0.31396967
59\C,-4.178950654,-0.4710760392,0.0534668181\C,-2.9768669329,0.2004187
547,0.1324487509\C,-1.714960646,-0.4134634697,-0.1013977218\C,-0.71622
96272,0.6118816456,0.064123259\C,-1.4344835534,1.8320354618,0.28835662
26\C,-0.8437333178,3.0943907418,0.3082919481\C,0.5147670832,3.14667126
59,0.0229829176\C,1.2840080938,1.9817628914,-0.1574628367\C,2.96160350
44,-0.2281420814,-0.2144057141\N,-2.7549193221,1.535218489,0.380437726
1\N,2.5896470956,2.1474708039,-0.4819611626\C,3.3612431206,1.087772100
7,-0.5526769153\H,4.3954925335,1.2562196023,-0.8473156858\H,1.02948113
03,4.0949895694,-0.0674639175\H,-1.4310047751,3.9891604652,0.468475552
7\\Version=ES64L-G16RevC.01\State=1-A\HF=-840.1509637\RMSD=3.995e-09\R
MSF=2.575e-05\Dipole=-2.2643972,-0.2014335,0.3513349\PG=C01 [X(C19H12N
2)]\\@

9 [R = H, T$_1$]

1\1\GINC-N0927\FOpt\RPBE1PBE TDA-FC\def2TZVP\C19H12N2\KA_PT6974\13-Nov
-2024\0\\# opt def2tzvp pbe1pbe TDA=(triplets,nstates=10) scrf=(cpcm,s
olvent=dichloromethane) empiricaldispersion=gd3bj\\Title Card Required
\\0,1\H,-5.1153076531,0.0391825951,0.0535772367\H,-0.7511122639,-2.287
9406956,-0.570841773\H,0.2990178808,-1.7860664467,1.1993574907\H,1.893
2752276,-3.6788860675,1.1971754917\H,4.1430163089,-3.3972780434,0.1938
161647\H,4.8305682389,-1.189190871,-0.6899728458\H,-5.0267742324,-2.38
82433462,-0.4632690103\H,-3.5023833206,2.2397465616,0.3534184796\H,-2.
8580342,-3.5125463389,-0.8026756227\C,3.8302248381,-1.3333419995,-0.29
71419892\C,3.444893988,-2.5679691496,0.2123292311\C,2.1888153519,-2.73
10334128,0.7649872241\C,1.2725232754,-1.6547675285,0.74690712\C,1.6002
077791,-0.4448809775,0.1737755709\C,0.6887363942,0.6892866288,0.061825
7457\C,-1.6858090971,-1.7707473284,-0.4060132975\C,-2.8804815731,-2.46
04839758,-0.5452443077\C,-4.1075981424,-1.8240127095,-0.3595468953\C,-
4.1673559756,-0.4720701247,-0.0630122582\C,-2.9674414129,0.2098552745,
0.0574826175\C,-1.7072143413,-0.4169297179,-0.0739844812\C,-0.70323438
03,0.6316891579,0.0775484461\C,-1.4447811793,1.8412025381,0.2393090747
\C,-0.8432369124,3.1112466414,0.3295654377\C,0.50089369,3.1819912525,0
.1652291574\C,1.3039647077,2.0036121698,-0.0567373217\C,2.9374627191,-
0.2350416556,-0.278915217\N,-2.7686243665,1.5566905778,0.264069018\N,2
.5647990206,2.1888274134,-0.383536794\C,3.3335953732,1.0599549383,-0.6
119502446\H,4.3395484207,1.2411743869,-0.9750039065\H,1.0218830928,4.1
313350729,0.1694342011\H,-1.4454542561,3.9977411802,0.4882872569\\Vers
ion=ES64L-G16RevC.01\State=1-A\HF=-840.1401166\RMSD=5.463e-09\RMSF=1.2
52e-05\Dipole=-2.7295944,-0.0662551,0.3168757\PG=C01 [X(C19H12N2)]\\@

9a [R = CH_3]

1\1\GINC-N1817\FOpt\RPBE1PBE\def2TZVP\C22H18N2\KA_PT6974\07-Nov-2023\0
\\# opt pbe1pbe/def2tzvp scrf=(cpcm,solvent=dichloromethane) empirical
dispersion=gd3bj\\Title Card Required\\0,1\H,0.0283465509,-0.008020954
3,0.0212016829\H,0.0133647542,-0.0200395531,5.0050607669\H,2.029863245
8,-0.0251795718,5.384407993\H,2.1058839517,0.985995877,7.6059840482\H,
2.3345337832,-0.4430533252,9.6263781197\H,2.6168845996,-2.8700705015,9
.3714261933\H,-1.2629334134,1.6840222683,1.297396991\H,1.5053057889,-2
.2900441235,0.375061136\H,-1.2828601025,1.634486463,3.7659341267\C,2.4
508765061,-2.2521354682,8.4979688444\C,2.3027787931,-0.8946176447,8.64
19285494\C,2.1562300454,-0.0920276249,7.504138268\C,2.0920981079,-0.66
05740507,6.2553402239\C,2.1695368078,-2.0543134994,6.0840391851\C,2.12
03469283,-2.6901487257,4.7944599302\C,0.0640345222,-0.0129992606,3.925
1316918\C,-0.6810987464,0.9159326557,3.2219659414\C,-0.6773969786,0.93
93105543,1.8238403305\C,0.0453376907,0.0063159491,1.1048362682\C,0.777
5031936,-0.93497021,1.8181050269\C,0.8441632075,-0.9472893983,3.233948
17\C,1.6658156194,-2.090043666,3.5937967916\C,1.9760225356,-2.73621967
88,2.3858138353\C,2.6228251357,-3.9774703187,2.2894337514\C,2.89755807
42,-4.6416482899,3.4672631452\C,2.6269702816,-4.0095708943,4.721416550
8\C,2.4206495319,-2.8490208023,7.22522001\N,1.4896873673,-2.0009730071
,1.3369793935\H,3.5485624513,-6.4231282443,4.4462597681\C,3.4902764272
,-6.0178213176,3.4399481682\H,2.8940753355,-6.6933857046,2.8215936016\
H,4.4999089004,-6.0030123396,3.0180904436\H,1.9911122792,-4.8989460259
,0.4515029694\C,2.9082186668,-4.5441505832,0.9346758363\H,3.3470255526
,-3.7868660281,0.2789046649\H,3.6006497347,-5.3817579787,0.9809357538\
N,2.8749340493,-4.7465664104,5.8488704411\C,2.7214912069,-4.2396564414
,7.0324593307\C,2.9295688549,-5.1434783724,8.2064759714\H,3.7781506628
,-4.8185801089,8.8152299873\H,2.0519255957,-5.1559371446,8.8583899905\
H,3.1219231001,-6.1545317453,7.8525590775\\Version=ES64L-G16RevC.01\St
ate=1-A\HF=-958.0106678\RMSD=7.041e-09\RMSF=1.966e-06\Dipole=-0.017496
8,0.2166766,-0.8844683\Quadrupole=-12.0722013,-6.223186,18.2953873,-2.
7351937,1.0278053,4.3100757\PG=C01 [X(C22H18N2)]\\@

9a·H$^+$ [R = CH_3]

1\1\GINC-N1806\FOpt\RPBE1PBE\def2TZVP\C22H19N2(1+)\KA_VR6109\27-Nov-20
23\0\\# opt=tight pbe1pbe/def2tzvp scrf=(cpcm,solvent=dichloromethane)
geom=connectivity empiricaldispersion=gd3bj\\Title Card Required\\1,1
\H,0.0192705051,-0.0008395411,-0.011363026\H,-0.0044283345,0.019540363
8,4.9741200774\H,1.9900755342,-0.0111941563,5.3647459352\H,2.034905781
,0.9931926822,7.5869352997\H,2.267256094,-0.4250819967,9.6104844534\H,
2.6116051449,-2.8447225063,9.3633879072\H,-1.2492457906,1.714607859,1.
2552904258\H,1.4806184104,-2.2853836249,0.3538008522\H,-1.2741788476,1
.6786533945,3.7223559792\C,2.436273963,-2.2299241127,8.4904106498\C,2.
255221972,-0.8799058877,8.6281395515\C,2.1058922383,-0.0831818478,7.48
49956374\C,2.0609345627,-0.6467644229,6.2337091022\C,2.1640950411,-2.0
354958456,6.0606048943\C,2.1030689962,-2.6609256844,4.7662109286\C,0.0
544548509,0.0207279453,3.8949983251\C,-0.6781922128,0.9512465976,3.184
5300441\C,-0.6724426226,0.966136477,1.7854454901\C,0.0369027676,0.0214
022416,1.07170429\C,0.7578873866,-0.9220978376,1.7934187564\C,0.825985
793,-0.9242642311,3.2072591172\C,1.6364062856,-2.0717273743,3.56874338
4\C,1.937315038,-2.7324420708,2.3622867703\C,2.5740719837,-3.983498817
2,2.2572571941\C,2.8607546202,-4.6418174852,3.4356516545\C,2.612515569
6,-3.968157549,4.6591360078\C,2.4312954224,-2.8207871965,7.2118655671\
N,1.4534595513,-2.0027861042,1.3179979105\H,2.7580302011,-6.7129607846
,4.0147132972\C,3.4257630312,-6.0286727035,3.4806713824\H,3.55636688,-
6.4444628357,2.4867114311\H,4.4034855746,-6.05580667,3.9719001183\H,1.
8984065709,-4.8505446616,0.4159233944\C,2.8309490527,-4.5263351285,0.8
88826282\H,3.2698870216,-3.7552033154,0.2505696336\H,3.5143652247,-5.3
707198697,0.8895422397\N,2.9028763191,-4.6423097515,5.8241476181\C,2.7
854114996,-4.1806855435,7.0501668514\C,3.0893366575,-5.0965828148,8.17
62898354\H,3.9303709299,-4.717308079,8.7600883609\H,2.229428957,-5.171

6413995,8.844713043\H,3.3387158206,-6.0946119771,7.8188543539\H,3.2174
133599,-5.5988505121,5.7208242879\\Version=ES64L-G16RevC.01\State=1-A\
HF=-958.4613369\RMSD=4.247e-09\RMSF=1.452e-06\Dipole=1.452375,-2.88344
43,1.3143937\Quadrupole=-25.1265173,3.1981876,21.9283296,-14.2366142,7
.1831741,-3.79118\PG=C01 [X(C22H19N2)]\\@

9b [R = *t*Bu]

1\1\GINC-N0421\FOpt\RPBE1PBE\def2TZVP\C25H24N2\KA_PT6974\20-Apr-2023\0
\\# opt pbe1pbe/def2tzvp scrf=(cpcm,solvent=dichloromethane) empirical
dispersion=gd3bj\\Title Card Required\\0,1\H,0.0500034811,0.003115436,
0.0237777707\H,0.0270176914,-0.0089406384,5.0083765896\H,2.0722213749,
-0.0325822264,5.3489339041\H,2.1617163702,1.0375187212,7.5467888856\H,
2.3828620933,-0.363063297,9.5947630955\H,2.6307401816,-2.7641772759,9.
3991105921\H,-1.2600701009,1.6815577265,1.297250915\H,1.5548857606,-2.
2622397457,0.3806553569\H,-1.2831809193,1.6341943204,3.7655769171\C,2.
4725579379,-2.1893535731,8.5010409787\C,2.3415032795,-0.8278073775,8.6
16750919\C,2.2000300582,-0.0428566535,7.4684602705\C,2.1286539573,-0.6
436034618,6.2375926675\C,2.1936963799,-2.0416638085,6.0959955701\C,2.1
454531343,-2.6664598406,4.802436209\C,0.0761921118,-0.0028765559,3.928
1874911\C,-0.6751367188,0.9196694351,3.2232791324\C,-0.6691458848,0.94
20972822,1.8250745405\C,0.0639940396,0.0161266043,1.1074248906\C,0.803
2153723,-0.918654368,1.8221720584\C,0.8640383171,-0.9313631633,3.23807
66739\C,1.6943930441,-2.0661693086,3.6007769501\C,2.0197403498,-2.7075
895346,2.3936136199\C,2.6853300455,-3.9403223279,2.2993939859\C,2.9582
584982,-4.6030017076,3.4780774702\C,2.6633523927,-3.9764691574,4.72922
12716\C,2.4350999653,-2.8337851499,7.2481749808\N,1.5293855446,-1.9766
436656,1.3434667761\H,3.6108221428,-6.3737356811,4.470561031\C,3.56923
70689,-5.9709330337,3.4618636022\H,2.9955302546,-6.6547370993,2.831439
1281\H,4.5874267657,-5.9426953073,3.0615439582\H,2.0869045259,-4.89282
75726,0.4653745196\C,2.990589575,-4.5040061756,0.9476942223\H,3.405937
9071,-3.7374139184,0.2877532131\H,3.710237937,-5.3182108188,1.00087216
36\N,2.8820987706,-4.7126387113,5.8567664832\C,2.711002602,-4.24083806
08,7.051429005\C,2.8604313065,-5.2469075454,8.1968595958\C,1.608720786
,-5.2493336182,9.0833388965\H,1.4155327537,-4.2971989799,9.5741077789\
H,0.7262126889,-5.5009615418,8.4894625184\H,1.7171029921,-6.009553091,
9.861108891\C,3.0075134443,-6.6598294122,7.6339698853\H,2.1474040976,-
6.9366240686,7.0222916615\H,3.8995386906,-6.7544281949,7.0146907691\H,
3.0839771056,-7.3640171241,8.4665804316\C,4.1267590183,-4.9620242849,9
.0163763715\H,4.1248193787,-3.9901136608,9.5068268697\H,4.2371669414,-
5.7256735591,9.7908161659\H,5.0082860781,-5.0069326204,8.372005617\\Ve
rsion=ES64L-G16RevC.01\State=1-A\HF=-1075.8464698\RMSD=5.639e-09\RMSF=
4.247e-06\Dipole=0.0231824,0.1227667,-0.9739906\Quadrupole=-12.5251322
,-6.1878697,18.7130019,-2.9348253,0.9836154,3.1894147\PG=C01 [X(C25H24
N2)]\\@

9c [R = CF_3]

1\1\GINC-N0502\FOpt\RPBE1PBE\def2TZVP\C20H11F3N2\KA_PT6974\21-May-2023
\0\\# opt=tight pbe1pbe/def2tzvp scrf=(cpcm,solvent=dichloromethane) e
mpiricaldispersion=gd3bj\\Title Card Required\\0,1\H,-0.0000809819,-0.
0001047657,-0.000018146\H,-0.0001236381,0.000079888,4.9843706278\H,2.0
11341247,-0.0000584722,5.3539838744\H,2.0796372737,1.0113620316,7.5746
924853\H,2.2939659523,-0.4148321727,9.5976491503\H,2.579524611,-2.8702
502559,9.3291172105\H,-1.2631524943,1.7124615214,1.2773907875\H,1.4694
459014,-2.2891079534,0.3492884837\H,-1.278447823,1.6661731009,3.745124
8828\C,2.412038452,-2.2257782489,8.4744823335\C,2.2653295197,-0.868809
7011,8.6142812046\C,2.1268403865,-0.0665953165,7.4742911848\C,2.066413
219,-0.6357270852,6.225096326\C,2.1419002245,-2.0283497211,6.055061750
1\C,2.0909906121,-2.6795256053,4.7722207189\C,0.0492323168,0.004979954
7,3.9047422498\C,-0.6860681309,0.9400437875,3.2013223334\C,-0.68542944
33,0.9612296224,1.8028951584\C,0.0214063255,0.0181663541,1.0831235951\
C,0.7428722526,-0.9300101919,1.7978045349\C,0.8172097937,-0.9383123659
,3.21199196\C,1.6263179715,-2.090961738,3.5703456179\C,1.9159857634,-2

.7454661835,2.354244454\C,2.5381728783,-3.9959009738,2.284626663\C,2.8
207721381,-4.6326042904,3.4582518215\C,2.593471964,-4.0057888348,4.707
7622687\C,2.3950531569,-2.8178797144,7.2004390724\N,1.4331434358,-2.01
15016522,1.3149424645\N,2.6879219353,-4.1496716387,7.1231391081\C,2.83
08220402,-4.6740009724,5.9515685124\C,3.2559420593,-6.1344093424,5.960
8432347\H,3.2391240392,-5.6278157813,3.4222944272\H,2.7386820196,-4.46
45790119,1.3290616255\F,3.3666162412,-6.623269168,7.1889488956\F,2.378
1600213,-6.9126387301,5.3047854901\F,4.448601235,-6.3005634124,5.36102
56704\\Version=ES64L-G16RevC.01\State=1-A\HF=-1177.0352413\RMSD=2.985e
-09\RMSF=5.577e-07\Dipole=-0.6108002,1.6897118,-2.3880823\Quadrupole=-
9.3131264,-7.1509641,16.4640905,-1.3865921,0.0146672,8.5911025\PG=C01
[X(C20H11F3N2)]\\@

9d [R = CH_2Cl]

1\1\GINC-N1512\FOpt\RPBE1PBE\def2TZVP\C22H17Cl1N2\KA_PT6974\24-May-202
3\0\\# opt=tight pbe1pbe/def2tzvp scrf=(cpcm,solvent=dichloromethane)
empiricaldispersion=gd3bj\\Title Card Required\\0,1\H,0.0839722241,-0.
1388481375,-0.0424628817\H,-0.001990948,0.1419637402,4.9327836077\H,2.
0044220796,0.0190931435,5.3405716405\H,2.1203293525,1.1536585065,7.499
9696044\H,2.2244548153,-0.1635514468,9.6041963574\H,2.3563253112,-2.60
90887876,9.502191916\H,-1.0987354274,1.7126058184,1.1109538524\H,1.398
2438965,-2.491621421,0.4673061531\H,-1.1577274075,1.808557102,3.577241
0892\C,2.2318002848,-2.0401442009,8.5896406101\C,2.1738626835,-0.67061
57694,8.6481223824\C,2.0976743811,0.0706952481,7.4620557745\C,2.011731
6088,-0.5652114789,6.2484576146\C,1.9969487009,-1.968951625,6.16138262
25\C,1.9186321104,-2.675027842,4.9106814995\C,0.0647654656,0.082107454
8,3.8554327091\C,-0.6014357294,1.0186619469,3.0864898179\C,-0.57604643
21,0.9601006869,1.6897472748\C,0.0871832544,-0.0625101588,1.0385840464
\C,0.7393090408,-1.009666696,1.8182871243\C,0.7858471567,-0.9432460069
,3.2328712228\C,1.5195479544,-2.1178905676,3.6718826788\C,1.7983261608
,-2.855052117,2.5079964656\C,2.3577818344,-4.1428758281,2.4906625688\C
,2.5729425811,-4.755030254,3.7070132779\C,2.3325453758,-4.0310365643,4
.9176676623\C,2.177737743,-2.708424068,7.3530210804\N,1.378494778,-2.1
517282831,1.4126334772\H,3.1128035465,-6.5066297151,4.8033501361\C,3.0
707718929,-6.1667992932,3.7723367173\H,2.423022285,-6.8410756255,3.206
6938602\H,4.0739259278,-6.2487815997,3.3437789885\H,1.6808242844,-5.04
18517165,0.6594574359\C,2.6179610966,-4.7999971091,1.1727096652\H,3.18
63418241,-4.1380323101,0.5128288273\H,3.1821160009,-5.7232759008,1.280
6017344\N,2.5109470763,-4.7119704651,6.0869738901\C,2.3805957771,-4.12
0102232,7.2328804746\C,2.5102275799,-4.993664424,8.4377261079\Cl,4.156
1089842,-4.8760816848,9.1662332003\H,1.8107710903,-4.7322604246,9.2286
264479\H,2.3784189895,-6.0320725248,8.1491324282\\Version=ES64L-G16Rev
C.01\State=1-A\HF=-1417.4767547\RMSD=7.130e-09\RMSF=5.942e-07\Dipole=-
0.8679176,0.4063357,-1.7715125\Quadrupole=-13.7280445,-1.684804,15.412
8484,0.9288455,-5.5341535,8.1544309\PG=C01 [X(C22H17Cl1N2)]\\@

9e [R = CH_2N_3]

1\1\GINC-N0940\FOpt\RPBE1PBE\def2TZVP\C22H17N5\KA_PT6974\21-May-2023\0
\\# opt=tight pbe1pbe/def2tzvp scrf=(cpcm,solvent=dichloromethane) emp
iricaldispersion=gd3bj\\Title Card Required\\0,1\H,0.0000229337,0.0000
072844,0.0000519257\H,-0.0000383703,-0.0000097635,2.0494788342\H,2.397
7399305,-0.0000216712,2.5137338246\H,3.5596235286,2.1146872304,3.10710
04275\H,2.2686467505,4.180479331,3.3615186571\H,-2.3734338536,-2.97286
13573,-1.9501748451\H,-5.3338103751,0.0224723452,0.6143663154\H,-0.190
6704734,-1.9064705538,-1.5094383903\C,1.7699265753,3.2755425196,3.0376
700238\C,2.4952997345,2.117489144,2.904778255\C,1.8427403234,0.9301570
635,2.5525403529\C,0.4984518488,0.9318876384,2.2708606389\C,-0.2562727
576,2.1168951546,2.3251129548\C,-1.6672753556,2.1609401699,2.043360004
6\C,-0.9682364667,-0.4565444353,-0.1507799777\C,-1.0789969457,-1.52864
97584,-1.0171028809\C,-2.3160284069,-2.1276037577,-1.2743265798\C,-3.3
542369737,-0.548717786,0.1601599856\C,-2.1086646561,0.0413308067,0.490
1729276\C,-2.407199408,1.1586765131,1.3694363735\C,-3.8089760211,1.220

8241849,1.4506831412\C,-4.5282995956,2.2524320857,2.0733685539\C,-3.80
55194,3.3170361314,2.5710589668\C,-2.3773850216,3.2820030054,2.5353017
512\C,0.3865154856,3.2888319149,2.7854261736\N,-4.3516686616,0.1620794
698,0.7732520365\H,-3.7847534016,5.2628900976,3.4516852078\C,-4.505312
5999,4.5043139741,3.1595056254\H,-5.2054343181,4.9448387905,2.44513890
27\H,-5.0835032569,4.2205597956,4.0436650253\H,-6.441194781,2.33726805
6,1.0970519726\C,-6.0220709793,2.1827617986,2.0973776502\H,-6.36223002
49,1.2022956846,2.4429413865\H,-6.4548633189,2.9335542169,2.7543997044
\N,-1.7225084328,4.392933447,2.9926130676\C,-0.4345839988,4.4319862049
,3.05644172\C,0.2142679779,5.731307696,3.4717363328\N,-0.7212138807,6.
7802156713,3.8486435475\N,-2.2375345095,7.6328850141,2.2842411168\C,-3
.47105182,-1.6373905269,-0.6954240196\H,-4.4398530928,-2.0710393576,-0
.9144608795\N,-1.489359485,7.171145156,2.9873935984\H,0.8416524987,5.5
73647159,4.3495894898\H,0.8754987882,6.0798257164,2.6681817593\\Versio
n=ES64L-G16RevC.01\State=1-A\HF=-1121.4855003\RMSD=7.502e-09\RMSF=7.47
0e-07\Dipole=-0.1351736,-1.631029,-0.3638439\Quadrupole=22.8568729,-13
.7623372,-9.0945357,10.0952526,5.3769089,2.5790197\PG=C01 [X(C22H17N5)
]\\@

12f [R = (*E*)-MeCH=CH]

1\1\GINC-N1535\FOpt\RPBE1PBE\def2TZVP\C24H20N2\KA_PT6974\21-May-2023\0
\\# opt=tight pbe1pbe/def2tzvp scrf=(cpcm,solvent=dichloromethane) emp
iricaldispersion=gd3bj\\Title Card Required\\0,1\H,0.0003065896,0.0000
327171,0.000003279\H,-0.0001134935,0.0000340588,4.9845513399\H,2.03653
35167,0.0000831523,5.3346511595\H,2.15822425,1.0450641498,7.5394885521
\H,2.4289979645,-0.3595433874,9.5733064737\H,2.6861500447,-2.778539926
,9.3533719626\H,-1.2980037642,1.6866360602,1.2756041825\H,1.4993412916
,-2.2688428296,0.3553422488\H,-1.3102165825,1.6443706281,3.7441452206\
C,2.4933619024,-2.1843585552,8.470306895\C,2.3644469866,-0.823612501,8
.5963100216\C,2.1971296309,-0.0346503948,7.4521297214\C,2.1062201434,-
0.6217377858,6.2148279804\C,2.1706083095,-2.0187614495,6.0617760578\C,
2.1161135204,-2.6572000044,4.7752973068\C,0.0456003812,0.0041981782,3.
9042526977\C,-0.7063787129,0.9272243521,3.2006207385\C,-0.7067892405,0
.9466323383,1.8023239128\C,0.0197976952,0.0164010012,1.0834824785\C,0.
7594189176,-0.918626458,1.797144349\C,0.8282608195,-0.927486602,3.2127
176458\C,1.6576626803,-2.0636473481,3.5737826831\C,1.9732350878,-2.709
2862609,2.3668673568\C,2.6353008214,-3.9443351296,2.2726903068\C,2.914
8531667,-4.6042530704,3.4502927805\C,2.6313851399,-3.975070421,4.70587
81536\C,2.4233065781,-2.8067300097,7.2099632805\N,1.4786676021,-1.9809
754941,1.3176216633\H,3.5832279338,-6.3732014061,4.438000753\C,3.52493
79434,-5.9726041281,3.4295728381\H,2.9405746764,-6.6585440845,2.811356
9292\H,4.5363632116,-5.9461581587,3.0126403929\H,2.0195138887,-4.87774
19928,0.4352714632\C,2.9307377377,-4.5095030749,0.9195048582\H,3.36131
37292,-3.7481078163,0.2631335096\H,3.6339305167,-5.3379111939,0.969511
8912\N,2.8599804396,-4.7085166763,5.8285915863\C,2.6965343035,-4.20838
97489,7.0198624479\C,2.8439986446,-5.1059047447,8.1711803552\H,2.49385
9201,-4.7459291539,9.1325453325\C,3.362855908,-6.3324464281,8.10287145
3\C,3.4935171302,-7.261204357,9.254182332\H,3.7162900299,-6.6839448787
,7.13655854\H,2.9486813906,-8.1921452295,9.0649292523\H,3.1140291713,-
6.8202805915,10.1776372998\H,4.5395471632,-7.5454760428,9.4085930314\\
Version=ES64L-G16RevC.01\State=1-A\HF=-1035.3458034\RMSD=2.590e-09\RMS
F=3.285e-07\Dipole=0.0964961,-0.0679979,-0.9331957\Quadrupole=-14.5982
025,-3.6606419,18.2588444,-4.7997248,1.6622677,1.3949948\PG=C01 [X(C24
H20N2)]\\@

12g [R = (*E*,*E*)-Me(CH=CH)$_2$]

1\1\GINC-N1121\FOpt\RPBE1PBE\def2TZVP\C26H22N2\KA_PT6974\24-May-2023\0
\\# opt=tight pbe1pbe/def2tzvp scrf=(cpcm,solvent=dichloromethane) emp
iricaldispersion=gd3bj\\Title Card Required\\0,1\H,0.3022879311,-0.158
587189,-0.1355579683\H,0.1333768402,0.3524567317,4.8201417493\H,2.1308
185792,0.0494382092,5.2690710385\H,2.346055118,1.2841021301,7.36669739
71\H,2.2932118966,0.0725152115,9.5377731068\H,2.1417389251,-2.36218489

33,9.5669505358\H,-0.7341870709,1.840604018,0.9062847216\H,1.385966886
5,-2.5958111267,0.5106163862\H,-0.8372961626,2.0542444593,3.3636344595
\C,2.0887206242,-1.8420706961,8.6201465742\C,2.1889553912,-0.473094095
5,8.6074534069\C,2.2034305746,0.209771349,7.3856635776\C,2.0608416858,
-0.479878359,6.2077826322\C,1.8917691064,-1.8764758387,6.19369692\C,1.
7746140187,-2.6249062564,4.9723907569\C,0.2150205941,0.2379191958,3.74
80546905\C,-0.3456578471,1.1952388095,2.9223460373\C,-0.295245327,1.07
09675165,1.5304276242\C,0.2872290879,-0.0352042801,0.9411433352\C,0.83
29440744,-1.0014410663,1.777389175\C,0.8513490915,-0.8761415929,3.1889
768643\C,1.4641433158,-2.0903162481,3.6986237031\C,1.7069827092,-2.899
4782548,2.5762317297\C,2.1556206729,-4.229465309,2.6331293757\C,2.2824
46649,-4.8020267178,3.8802900208\C,2.0631183649,-4.0102600331,5.055082
0275\C,1.9636698326,-2.5727250669,7.4239386928\N,1.3777993248,-2.21182
7873,1.4389433749\H,2.6323828294,-6.5391271831,5.0680546786\C,2.657957
4012,-6.2455682851,4.0221063493\H,1.9779772778,-6.8901432298,3.4594462
004\H,3.6662546835,-6.4292338916,3.6389319181\H,1.4532063496,-5.207127
2036,0.8514806765\C,2.3964113735,-4.9687041731,1.3552626159\H,2.990774
3541,-4.3671190595,0.6616899029\H,2.9268195148,-5.9039327251,1.5195400
908\N,2.1250095854,-4.6517429462,6.2504324861\C,1.9991896385,-4.013813
8527,7.3818209445\C,1.9378185816,-4.8021322458,8.608302785\H,1.7346750
631,-4.2796509775,9.5357022101\C,2.0928578077,-6.1357854984,8.65387671
4\C,2.0103127774,-6.9151846482,9.8600723427\H,2.2931824178,-6.66576607
78,7.7260610068\C,2.1700509553,-8.2442987422,9.8940701372\C,2.09193601
61,-9.0819796543,11.1176435479\H,1.8063016288,-6.3804361123,10.7867139
678\H,1.8855007542,-8.4806996776,12.0047397689\H,3.0277853481,-9.62741
79476,11.2787786671\H,1.3078322456,-9.8405980704,11.0230622819\H,2.373
4805977,-8.7619970919,8.9578888222\\Version=ES64L-G16RevC.01\State=1-A
\HF=-1112.688755\RMSD=2.370e-09\RMSF=5.698e-07\Dipole=0.0871137,-0.352
728,-0.8705145\Quadrupole=-19.8371472,0.3546709,19.4824763,-2.6657725,
0.6076055,-0.246176\PG=C01 [X(C26H22N2)]\\@

12h [R = Ph]

1\1\GINC-N1616\FOpt\RPBE1PBE\def2TZVP\C27H20N2\KA_PT6974\22-Apr-2023\0
\\# opt pbe1pbe/def2tzvp scrf=(cpcm,solvent=dichloromethane) empirical
dispersion=gd3bj\\Title Card Required\\0,1\H,0.0375301153,-0.005926433
3,0.022930755\H,0.0047237831,-0.0280778088,5.0078655442\H,2.047380824,
-0.0318031913,5.3684844554\H,2.2119669305,1.0051567049,7.5731986566\H,
2.512749534,-0.403285858,9.5986325062\H,2.7161018201,-2.8387959521,9.3
669791208\H,-1.2611259493,1.6803432435,1.2975162273\H,1.5322395465,-2.
2802279729,0.3785555363\H,-1.2896262169,1.6286519572,3.7654151633\C,2.
5112408916,-2.2297105828,8.4964316094\C,2.4125663393,-0.8667696768,8.6
243842146\C,2.2324883629,-0.0749294145,7.4840388685\C,2.1156728409,-0.
6572936251,6.2462356606\C,2.1574110561,-2.0544945791,6.0901619794\C,2.
1062171639,-2.6908786293,4.8015267191\C,0.056655314,-0.0192590113,3.92
78026805\C,-0.6858514005,0.9105317678,3.2231522392\C,-0.6768052049,0.9
354098813,1.8249557735\C,0.0501579771,0.0050868833,1.1065768219\C,0.78
12201504,-0.9361350356,1.8210587109\C,0.8389044438,-0.951360699,3.2367
746134\C,1.660545008,-2.0920378249,3.5989887558\C,1.9861318385,-2.7331
434012,2.3917566432\C,2.6510937928,-3.9664304904,2.2983541365\C,2.9216
707588,-4.6304297657,3.4765508325\C,2.6233813711,-4.0069101995,4.72925
4637\C,2.3966099952,-2.8475721634,7.2374128979\N,1.5015357561,-1.99872
31347,1.3424415167\H,3.5875474391,-6.4013999739,4.464128217\C,3.535265
2544,-5.9970630743,3.4568565243\H,2.9575641144,-6.6824656069,2.8318687
023\H,4.5495378752,-5.9659287079,3.0473128808\H,2.0554803388,-4.915309
6416,0.4624773103\C,2.9589154616,-4.5283589739,0.9466385646\H,3.376003
8896,-3.7609473366,0.2887946065\H,3.6775872483,-5.3433138135,0.9996276
148\N,2.8293854383,-4.7499531427,5.8549490543\C,2.6413266229,-4.250597
7469,7.0393542277\H,4.6871947737,-7.7663405962,9.2436793504\C,3.873915
679,-7.0496709697,9.2326373664\C,2.9310866469,-7.0514110376,10.2526959
892\C,3.7786989743,-6.1325110901,8.1987768186\C,1.8933086515,-6.130368
5253,10.2304983324\H,3.0035266728,-7.7705815313,11.0603602241\C,2.7481
233681,-5.1940209349,8.1750216247\H,4.5057646319,-6.1304455475,7.39549

25267\C,1.8048363329,-5.204635942,9.2017097293\H,1.1456259337,-6.13412
31301,11.0152607011\H,0.983376451,-4.4976768574,9.1830436975\\Version=
ES64L-G16RevC.01\State=1-A\HF=-1149.5988284\RMSD=5.974e-09\RMSF=3.126e
-06\Dipole=0.0061869,0.2665675,-1.0946458\Quadrupole=-13.2366688,-6.81
20798,20.0487486,-5.2087065,-0.7435589,0.5982118\PG=C01 [X(C27H20N2)]\
\@

12i [R = $MeOC_6H_4$]

1\1\GINC-N0214\FOpt\RPBE1PBE\def2TZVP\C28H22N2O1\KA_PT6974\21-May-2023
\0\\# opt=tight pbe1pbe/def2tzvp scrf=(cpcm,solvent=dichloromethane) e
mpiricaldispersion=gd3bj\\Title Card Required\\0,1\H,0.0002935302,0.00
0155867,-0.0000889903\H,-0.0001367977,-0.0000317007,4.9850661847\H,2.0
445393555,0.0000329179,5.3294947163\H,2.2415062626,1.0517104415,7.5243
974045\H,2.5780606217,-0.3434770002,9.5536413005\H,2.7760887472,-2.780
2349625,9.3342933121\H,-1.2910968453,1.6914347258,1.275360531\H,1.4978
937985,-2.2731450646,0.3563896068\H,-1.3038596043,1.6506315132,3.74360
50893\C,2.5551982102,-2.1772442055,8.4634916392\C,2.4601470949,-0.8132
066631,8.5843596494\C,2.2608476117,-0.0289744442,7.4421895655\C,2.1254
90576,-0.6197694718,6.2102044033\C,2.1652812676,-2.0179951603,6.062468
7292\C,2.1026065116,-2.6613438068,4.7776737041\C,0.0444620052,0.004125
207,3.9046127846\C,-0.7032051972,0.9303709273,3.2006931056\C,-0.703121
2031,0.9490411541,1.8023460271\C,0.019697271,0.0158505798,1.0834100361
\C,0.7556158061,-0.9221229853,1.7971885209\C,0.8226595742,-0.930705509
5,3.2127158187\C,1.6476003898,-2.0688439955,3.5751400286\C,1.965427579
7,-2.715312419,2.3691594281\C,2.6306510681,-3.9484308056,2.2773490465\
C,2.9112548145,-4.6058933026,3.4569378412\C,2.6205338167,-3.9768980361
,4.7090257407\C,2.4180785322,-2.8042939839,7.2114228354\N,1.4733139661
,-1.9864309328,1.3189307645\H,3.5956055268,-6.3655474088,4.4509764647\
C,3.5277165814,-5.9714691694,3.4405674272\H,2.941784081,-6.6645618344,
2.8317925647\H,4.5354058082,-5.9424968774,3.0151226602\H,2.0158303689,
-4.8685960166,0.4334716203\C,2.9286099178,-4.5142310068,0.9250662531\H
,3.3735826516,-3.7566540717,0.273782471\H,3.6201686421,-5.3521444993,0
.977488226\N,2.8309639473,-4.7157333034,5.8367462351\C,2.6539885074,-4
.2113999078,7.0217271934\H,4.6680421624,-7.766733814,9.1782927586\C,3.
8712347848,-7.0358206988,9.2013577512\C,2.9569858961,-7.0037159537,10.
2516767509\C,3.7609468789,-6.1162979989,8.1696170974\C,1.9427982032,-6
.0461131636,10.248949509\C,2.7655851657,-5.1451593749,8.1608905802\H,4
.4673627286,-6.1473311226,7.3486187617\C,1.8552467039,-5.1299029687,9.
220869844\H,1.2266521321,-6.0407252531,11.0620928409\H,1.0497359668,-4
.4047158368,9.2282371321\O,2.9730265172,-7.8521027498,11.3012741564\C,
3.9794992562,-8.8430854003,11.3347451422\H,3.8125856631,-9.4135382879,
12.2460064985\H,3.9086836613,-9.5110391631,10.4710956209\H,4.977408014
3,-8.3955235101,11.3654649665\\Version=ES64L-G16RevC.01\State=1-A\HF=-
1264.0488748\RMSD=4.464e-09\RMSF=6.082e-07\Dipole=0.5378798,-0.30439,-
0.9977981\Quadrupole=-14.8117612,-1.9383541,16.7501153,-11.0196767,5.2
84879,-4.5279378\PG=C01 [X(C28H22N2O1)]\\@

12j [R = $F_3CC_6H_4$]

1\1\GINC-N1837\FOpt\RPBE1PBE\def2TZVP\C28H19F3N2\KA_PT6974\17-May-2023
\0\\# opt=tight pbe1pbe/def2tzvp scrf=(cpcm,solvent=dichloromethane) e
mpiricaldispersion=gd3bj\\Title Card Required\\0,1\H,0.0434416618,-0.0
070067881,0.0226934675\H,-0.00012771,-0.0324071392,5.0076080107\H,2.04
21411675,-0.0340750516,5.371571988\H,2.198124703,1.001075011,7.5778588
685\H,2.4954999236,-0.4082865762,9.6028154884\H,2.7056563778,-2.840941
0271,9.3715689523\H,-1.2602642379,1.6765229507,1.2956913271\H,1.540586
4442,-2.2805955887,0.3802517773\H,-1.2941631773,1.6231298873,3.7633290
389\C,2.5005839887,-2.2337191188,8.4997145135\C,2.3989479068,-0.871215
8305,8.6280122551\C,2.2208940262,-0.0788198814,7.4876509927\C,2.109019
8649,-0.6600928179,6.2490104616\C,2.1537814205,-2.0570188306,6.0918890
911\C,2.1061435993,-2.6923698371,4.8026759018\C,0.0541137031,-0.022865
774,3.9276901864\C,-0.6882464135,0.9061668956,3.2219678399\C,-0.676190
4662,0.931940142,1.8238595732\C,0.0534827046,0.0030822309,1.1063431582

\C,0.7839076758,-0.9376399582,1.8219231241\C,0.8388125543,-0.953451635
6,3.2375480515\C,1.6610469253,-2.0934849904,3.6006137974\C,1.989478106
4,-2.733683974,2.3934411363\C,2.6559671148,-3.9663361863,2.2989225054\
C,2.926692236,-4.6310948139,3.4763136242\C,2.6260274732,-4.0079853559,
4.7288442246\C,2.3908619551,-2.850369415,7.2395624387\N,1.5062525786,-
1.9997089501,1.3442628478\H,3.603473046,-6.3976920397,4.4654413657\C,3
.5423436193,-5.9968078776,3.4573490479\H,2.9602481472,-6.6856905345,2.
8403219687\H,4.552896424,-5.9655327336,3.0391736247\H,2.0549909099,-4.
8699135477,0.4429257207\C,2.9646515259,-4.5230703402,0.9454378006\H,3.
4188033474,-3.7624403492,0.3041028349\H,3.6523003384,-5.3641123235,0.9
98072361\N,2.8318407942,-4.7500858307,5.8540823908\C,2.6389790323,-4.2
508708824,7.0374251328\H,4.6573511761,-7.8042356635,9.2025280237\C,3.8
557481347,-7.0760027811,9.200788546\C,3.7626603545,-6.1504754228,8.178
2357873\C,1.8845412314,-6.1414713477,10.2188837488\C,2.7419257356,-5.2
012346819,8.1675597767\H,4.4847900326,-6.153334383,7.3715080512\C,1.80
31165374,-5.2105288548,9.1974908038\H,1.1404791007,-6.1483655082,11.00
53885777\H,0.9861417019,-4.499373272,9.188897453\C,2.9139912737,-7.071
7610652,10.2224747209\C,3.0406845825,-8.0446132677,11.3533444573\F,1.8
601252053,-8.3221067546,11.9225787569\F,3.8353988889,-7.5714404409,12.
3330233069\F,3.5744716859,-9.2105926521,10.9630809054\\Version=ES64L-G
16RevC.01\State=1-A\HF=-1486.482521\RMSD=4.446e-09\RMSF=4.381e-07\Dipo
le=-0.145132,1.2790537,-2.2913479\Quadrupole=-5.8206612,-9.4167796,15.
2374408,-3.6181891,-3.3120081,12.4607841\PG=C01 [X(C28H19F3N2)]\\@

12k [R = mesityl]

1\1\GINC-N1236\FOpt\RPBE1PBE\def2TZVP\C30H26N2\KA_PT6974\20-Jun-2023\0
\\# opt=tight pbe1pbe/def2tzvp scrf=(cpcm,solvent=dichloromethane) emp
iricaldispersion=gd3bj\\Title Card Required\\0,1\H,-0.0019023881,0.001
7140385,0.0018955573\H,0.0050204154,0.0034418037,4.9859292158\H,2.0230
637163,0.0001855901,5.3633498111\H,2.1221449798,1.0077731157,7.5843580
07\H,2.3624864736,-0.4197940379,9.6034243784\H,2.628389058,-2.86269911
25,9.339755987\H,-1.2805460565,1.7018936527,1.2788197492\H,1.470693146
1,-2.2840178839,0.3558817762\H,-1.289794331,1.6599550673,3.747434598\C
,2.4547077786,-2.230992537,8.4764412554\C,2.3173600587,-0.8732328021,8
.6203736507\C,2.1648202236,-0.0705449019,7.4820440183\C,2.0873032405,-
0.6368464662,6.2329974819\C,2.1551565865,-2.0313385527,6.0608738427\C,
2.0992994698,-2.6724030106,4.7745751497\C,0.0510073475,0.0074617448,3.
9057785794\C,-0.6933186929,0.9373184101,3.2030856939\C,-0.6956324482,0
.9564517079,1.8048815035\C,0.0201107311,0.0185356751,1.0853248959\C,0.
7520082652,-0.9232800419,1.7982103267\C,0.8242967868,-0.9316037268,3.2
135587321\C,1.6431689105,-2.0759754741,3.5732058862\C,1.9477186151,-2.
7268475375,2.3658450605\C,2.5940707699,-3.9692326576,2.2708796799\C,2.
8710771845,-4.6300147336,3.4495927582\C,2.6033713956,-3.9943141841,4.7
024380603\C,2.4065079499,-2.8245866414,7.2028008158\N,1.4588974176,-1.
9934457361,1.3174760872\H,3.5121620993,-6.4117438483,4.4339668418\C,3.
4638750304,-6.0060145982,3.4272147289\H,2.8746026409,-6.6809800547,2.8
018588522\H,4.4780240664,-5.990099486,3.016230548\H,1.9640095039,-4.92
38793656,0.4491076507\C,2.8775856204,-4.5426328035,0.9185968472\H,3.29
33658914,-3.7835918329,0.25027053\H,3.5890688929,-5.3641624468,0.96703
81861\N,2.8507710654,-4.7295779559,5.8298436966\C,2.6971257772,-4.2142
849836,7.0095147506\H,5.3649309752,-6.4842785136,10.0437128754\C,4.358
7733818,-6.2512281353,9.7076312056\C,3.2727233365,-6.8021629426,10.377
7435626\C,4.1937537533,-5.4074417503,8.6147728983\C,1.9955052724,-6.49
2444779,9.9247333128\C,2.8986798017,-5.1082928885,8.182304217\C,1.7892
365786,-5.6489811796,8.8389745332\H,1.1336951992,-6.9209775314,10.4280
071036\C,5.3880031447,-4.8314727787,7.9155215948\C,0.397936595,-5.3349
747495,8.376967044\C,3.4722311563,-7.6846257211,11.5722920249\H,0.1725
438696,-4.270816829,8.4910348791\H,-0.3387659673,-5.8995501466,8.94900
32074\H,0.2683909796,-5.5740114348,7.3183405129\H,5.4675093988,-5.2123
635323,6.8943669497\H,6.3069083092,-5.0790851268,8.4476292042\H,5.3172
984019,-3.7428270131,7.8410924879\H,2.6689980396,-8.4180867247,11.6626
057374\H,3.4803922297,-7.0949166673,12.4943432502\H,4.4230903433,-8.21

75694259,11.5180253629\\Version=ES64L-G16RevC.01\State=1-A\HF=-1267.45
29275\RMSD=4.630e-09\RMSF=6.518e-07\Dipole=-0.0471177,0.3093878,-1.050
9654\Quadrupole=-11.8223698,-8.9466492,20.7690191,-2.8186055,1.721389,
-0.3795957\PG=C01 [X(C30H26N2)]\\@

12l [R = 3-pyridinyl]

1\1\GINC-N1039\FOpt\RPBE1PBE\def2TZVP\C26H19N3\KA_PT6974\21-May-2023\0
\\# opt=tight pbe1pbe/def2tzvp scrf=(cpcm,solvent=dichloromethane) emp
iricaldispersion=gd3bj\\Title Card Required\\0,1\H,0.0000790201,0.0000
370349,0.0000194652\H,0.0003367815,0.0000732999,4.9850925481\H,2.04417
32632,0.0000372237,5.3318083666\H,2.2173118955,1.0456590051,7.53158766
25\H,2.5320398233,-0.3534224861,9.561046602\H,2.7468649946,-2.78643975
65,9.3391910672\H,-1.2904983187,1.6916452618,1.2758770407\H,1.49875104
54,-2.2712668872,0.3553035215\H,-1.302671194,1.6505859688,3.7440257131
\C,2.532342375,-2.1840509069,8.4661709405\C,2.4288859186,-0.8211452931
,8.5892181973\C,2.2407446953,-0.0346340016,7.4464370335\C,2.119234605,
-0.6219340352,6.211440276\C,2.1640736864,-2.0194883602,6.060440712\C,2
.1039731782,-2.6621853772,4.7752392661\C,0.0455466193,0.0042007984,3.9
047215561\C,-0.7020873397,0.9304567122,3.2009176771\C,-0.7023438858,0.
9492626684,1.8026172255\C,0.0200411235,0.0160316892,1.0834703991\C,0.7
561942029,-0.9216072959,1.7973291139\C,0.8237300133,-0.9304170539,3.21
25089411\C,1.6483165042,-2.0693429571,3.5738649752\C,1.9651620915,-2.7
160957193,2.3669703678\C,2.6292622004,-3.9500190844,2.2735198187\C,2.9
085224277,-4.6091642524,3.4522003722\C,2.6208043367,-3.9790081954,4.70
38840709\C,2.4139548402,-2.8062671702,7.2097591274\N,1.4730337569,-1.9
866353504,1.3184612538\H,3.5796559771,-6.3776856125,4.4420532843\C,3.5
209319165,-5.9762473796,3.4340296403\H,2.9388305567,-6.6632374846,2.81
50087311\H,4.5326278825,-5.9470575772,3.0181219745\H,2.0189135926,-4.9
044181774,0.4457083449\C,2.9265602122,-4.5175250817,0.9219060044\H,3.3
404884464,-3.7532610935,0.2584775986\H,3.6440126803,-5.3336242134,0.97
29625975\N,2.8363513128,-4.7160757536,5.8312108711\C,3.1024605505,-7.0
053418458,10.1153365521\C,3.8188451921,-6.0811657237,8.1650598896\C,2.
0333649463,-6.1274643995,10.1970002573\H,4.5431933421,-6.0780115366,7.
356169464\C,1.8736722172,-5.1814161096,9.1990858124\H,1.3363904305,-6.
1902033754,11.0233635401\H,1.0383849548,-4.4910988611,9.223799627\H,3.
2546009064,-7.7579635531,10.8833654975\C,2.7842878405,-5.1458055469,8.
1484561972\C,2.6602963684,-4.2077757883,7.0135268156\N,3.9863150522,-6
.9880251758,9.1189903341\\Version=ES64L-G16RevC.01\State=1-A\HF=-1165.
6302197\RMSD=2.530e-09\RMSF=1.290e-07\Dipole=-0.8797417,1.1134351,-1.2
262478\Quadrupole=-13.8168445,-12.0536382,25.8704827,3.600301,-6.82145
46,6.3279472\PG=C01 [X(C26H19N3)]\\@

12m [R = 2-pyridinyl]

1\1\GINC-N0334\FOpt\RPBE1PBE\def2TZVP\C26H19N3\KA_PT6974\11-Sep-2024\0
\\# opt=tight pbe1pbe/def2tzvp scrf=(cpcm,solvent=dichloromethane) emp
iricaldispersion=gd3bj\\Title Card Required\\0,1\H,-0.011329868,0.0170
735165,0.0104577469\H,0.0224699472,0.0124229858,4.9951432736\H,2.06344
20635,-0.0007690034,5.3366502009\H,2.2306567963,1.0320554692,7.5430471
479\H,2.52529104,-0.3788510261,9.5672018963\H,2.7372694825,-2.81325375
95,9.3327334599\H,-1.2824568188,1.7158433127,1.2964615526\H,1.47486230
23,-2.2636859714,0.3531752393\H,-1.2782916511,1.6720392897,3.764706650
6\C,2.534399535,-2.2032262912,8.4618309329\C,2.4303731877,-0.841191110
8,8.591936026\C,2.2520673167,-0.047806623,7.4519003102\C,2.1345139214,
-0.6277374642,6.2130685744\C,2.1763616181,-2.0246761671,6.0548809724\C
,2.1094497789,-2.663531824,4.7683897511\C,0.0608186921,0.0172616249,3.
9145661977\C,-0.6858571568,0.9488501908,3.2167200077\C,-0.6954635571,0
.9692493466,1.8185315693\C,0.0161229748,0.0320747031,1.0937646629\C,0.
7511213772,-0.9108481156,1.8017689458\C,0.8287855749,-0.9214839257,3.2
164740154\C,1.6492350118,-2.0658582381,3.5708142303\C,1.9525249555,-2.
7135908752,2.3610210602\C,2.6070447848,-3.952141192,2.260720975\C,2.89
27658561,-4.6136477202,3.4363452351\C,2.6183235089,-3.9835050124,4.691
3651759\C,2.4252849123,-2.8173506537,7.2002768964\N,1.4573921681,-1.98

01217727,1.3168296227\H,3.5936204402,-6.3717351176,4.4229927979\C,3.49
97371746,-5.9830705763,3.4126694204\H,2.8929997355,-6.6755925144,2.823
7702134\H,4.4952142772,-5.9622814513,2.9593211041\H,1.9704012717,-4.88
73548664,0.4323314273\C,2.8875914294,-4.5199922011,0.9057112444\H,3.31
3147121,-3.7613220691,0.2430609146\H,3.5888886432,-5.3502958472,0.9503
84476\N,2.8442347001,-4.7230664365,5.8153026188\C,3.0293569397,-6.9679
933681,10.1644897669\C,1.9170342185,-6.1433712594,10.1340228983\C,1.80
27487193,-5.218251701,9.1108331306\H,1.1471721721,-6.22033389,10.89282
78192\H,0.9440245976,-4.5613373505,9.0478172462\H,3.1665031658,-7.7043
03195,10.9464246808\C,2.8019102147,-5.1553917368,8.142449365\C,2.67997
39818,-4.2147638611,6.996420701\N,3.8715434538,-5.9523878173,8.1656820
069\C,3.9741606171,-6.8290400395,9.1584561212\H,4.861157263,-7.4564468
301,9.1526057815\\Version=ES64L-G16RevC.01\State=1-A\HF=-1165.6287241\
RMSD=9.690e-09\RMSF=5.456e-07\Dipole=-0.8153937,0.3076872,-0.4549959\Q
uadrupole=-19.3043908,-7.7615254,27.0659162,-0.9671546,-2.1189989,-3.8
546652\PG=C01 [X(C26H19N3)]\\@

12n [R = 1-naphthalenyl]

1\1\GINC-N0337\FOpt\RPBE1PBE\def2TZVP\C31H22N2\KA_PT6974\11-Sep-2024\0
\\# opt=tight pbe1pbe/def2tzvp scrf=(cpcm,solvent=dichloromethane) emp
iricaldispersion=gd3bj\\Title Card Required\\0,1\H,0.0883055003,0.0541
231884,-0.0113198134\H,0.145571179,0.1746151636,4.9715270079\H,2.17449
20798,0.0453993084,5.3174375785\H,2.4015959917,1.1098026377,7.50294699
08\H,2.6022102141,-0.2728903188,9.5576822306\H,2.6701426321,-2.7278770
169,9.3694692523\H,-1.0667814111,1.8627497297,1.2346822994\H,1.4227110
825,-2.3097450138,0.3857610365\H,-1.0533075239,1.8813606213,3.70328382
02\C,2.5170045859,-2.1174016998,8.4882238004\C,2.489268754,-0.74971056
68,8.5913378901\C,2.3615802098,0.0288126083,7.4335102055\C,2.211138556
2,-0.567441202,6.2058538986\C,2.1725342967,-1.9677929481,6.0763164951\
C,2.0588680002,-2.6343110129,4.8069490351\C,0.179441935,0.1496006488,3
.8911140089\C,-0.5103602572,1.108535061,3.1720270407\C,-0.5256387497,1
.093969873,1.7738300314\C,0.1216314373,0.0953323423,1.0711850575\C,0.7
987306366,-0.8741412981,1.8007250533\C,0.8831234173,-0.8535805369,3.21
51180826\C,1.6306809539,-2.0388185577,3.5959651775\C,1.8845158094,-2.7
352939395,2.4023525038\C,2.4542706051,-4.0163773314,2.3318922736\C,2.7
013008584,-4.6655535768,3.5235130394\C,2.480673338,-3.9857778216,4.762
5818469\C,2.381080411,-2.7459592476,7.2373878025\N,1.4326653361,-1.998
0978968,1.3407915628\H,3.2536076135,-6.455013909,4.5475893569\C,3.2092
767418,-6.0751379957,3.5305705576\H,2.5668574994,-6.7304622714,2.93735
29739\H,4.2133336309,-6.1327639079,3.0993932671\H,1.7480458041,-4.9492
311338,0.5274063224\C,2.6898534257,-4.6345277066,0.9903146768\H,3.1633
593251,-3.9218515261,0.3093155282\H,3.333783436,-5.5089739231,1.052667
3591\N,2.6768991705,-4.7096937849,5.9055069199\C,2.5549376659,-4.16093
1942,7.0748992197\H,5.0397821814,-7.0621140021,9.6728822293\C,4.086903
6174,-6.5615055733,9.5467082187\C,3.084743114,-6.7440340238,10.4586972
364\C,3.8905025648,-5.7150471807,8.4416385444\C,1.8388458351,-6.099931
0394,10.2943412023\H,3.2287940481,-7.390501519,11.3177119782\C,2.70283
52479,-5.0522403792,8.2537667492\H,4.6910646158,-5.5696729394,7.725550
3363\C,1.6351505424,-5.2439231425,9.1748792258\H,-1.2312796475,-5.8462
407957,11.7415150352\C,-0.4283897638,-5.6869520909,11.0311444362\C,-0.
6384779792,-4.8567250499,9.9132547043\C,0.7823230359,-6.2962082031,11.
2125676862\C,0.3658931938,-4.6421549798,9.0090740915\H,-1.6047425162,-
4.3878246856,9.7674841944\H,0.9502786771,-6.9465030094,12.064516495\H,
0.1903173353,-4.0059846115,8.1502704871\\Version=ES64L-G16RevC.01\Stat
e=1-A\HF=-1303.1227399\RMSD=9.509e-09\RMSF=3.220e-07\Dipole=-0.04964,0
.2981522,-1.1001982\Quadrupole=-12.0312991,-9.6277174,21.6590164,-4.83
49754,-0.4790432,-1.5379582\PG=C01 [X(C31H22N2)]\\@

7.2.5 Pyridine

Pyridine

1\1\GINC-N0332\FOpt\RPBE1PBE\def2TZVP\C5H5N1\KA_PT6974\08-Aug-2024\0\\
# opt pbe1pbe/def2tzvp scrf=(cpcm,solvent=dichloromethane) empiricaldi
spersion=gd3bj\\Title Card Required\\0,1\C,0.0131935581,0.0000292257,0
.0108256996\C,0.0095699075,-0.0000627948,1.3975957145\C,1.2260831762,-
0.0003039702,2.0613592307\C,2.3909913191,-0.000407903,1.3107239334\C,2
.2863178814,-0.0003036563,-0.0720944906\N,1.1245101718,-0.0000972012,-
0.7226256666\H,1.2655619958,-0.0003585161,3.1444323458\H,-0.9244730373
,0.0001935362,-0.5377125826\H,-0.9278500335,0.0000622315,1.9396609054\
H,3.365440101,-0.0005418792,1.7829958526\H,3.181513981,-0.0003711593,-
0.6875069365\\Version=ES64L-G16RevC.01\State=1-A\HF=-248.0888753\RMSD=
6.418e-09\RMSF=2.001e-04\Dipole=0.041571,-0.0000464,1.1394851\Quadrupo
le=4.3138116,-2.8072955,-1.5065161,-0.0009618,-0.2126843,-0.000009\PG=
C01 [X(C5H5N1)]\\@

Pyridine·H+

1\1\GINC-N0333\FOpt\RPBE1PBE\def2TZVP\C5H6N1(1+)\KA_PT6974\08-Aug-2024
\0\\# opt pbe1pbe/def2tzvp scrf=(cpcm,solvent=dichloromethane) empiric
aldispersion=gd3bj\\Title Card Required\\1,1\C,-0.0267156642,0.0000070
923,0.0152274738\C,-0.0011310065,-0.0000642369,1.3896650721\C,1.225275
3068,-0.0002544305,2.0393412937\C,2.4010711141,-0.0003995175,1.3020170
89\C,2.326416569,-0.0003296809,-0.0706317508\N,1.1266712543,-0.0001240
329,-0.6629671501\H,1.2647496422,-0.0003252362,3.1213437059\H,-0.93453
35756,0.0001645627,-0.5703775655\H,-0.9327492388,0.0000354877,1.937603
9386\H,3.3701492088,-0.0005649722,1.780596238\H,3.1891432675,-0.000420
1473,-0.720841011\H,1.0897137024,-0.0000775866,-1.6758953007\\Version=
ES64L-G16RevC.01\State=1-A\HF=-248.5303165\RMSD=4.794e-09\RMSF=9.237e-
05\Dipole=-0.0356538,0.0000407,-0.9772231\Quadrupole=2.6249713,-8.4337
156,5.8087443,-0.0015799,0.1163167,-0.0006851\PG=C01 [X(C5H6N1)]\\@

8. References

18. M. Jian, Z. Song, X. Chen, J. Zhao, B. Xu, Z. Chi, *Chem. Eng. J. (Amsterdam, Neth.)* **2022**, **429**, 132346.
27. I. Marten, J. Podlech, *Org. Lett.* **2024**, *26*, 1148-1153.
29. J.-H. Lee, S.-J. Jung, S.-K. Kang, K.-Y. Kim, D.-J. Kim, J.-S. Choi, D.-H. Choi, S.-J. Eum, J.-D. Lee (Heesung Material Ltd.), US 10446765 B2, **2019**.
30. W. C. P. Tsang, R. H. Munday, G. Brasche, N. Zheng, S. L. Buchwald, *J. Org. Chem.* **2008**, *73*, 7603-7610.
31. J. R. Dunetz, Y. Xiang, A. Baldwin, J. Ringling, *Org. Lett.* **2011**, *13*, 5048-5051.
32. T. R. Papo, D. Jaganyi, A. Mambanda, *J. Coord. Chem.* **2022**, *75*, 2557-2573.
34. J. Xi, Q.-L. Dong, G.-S. Liu, S. Wang, L. Chen, Z.-J. Yao, *Synlett* **2010**, 1674-1678.
35. R. Heckershoff, L. Eberle, N. Richert, C. Delavier, M. Bruckschlegel, M. R. Schäfer, P. Krämer, F. Rominger, M. Rudolph, A. S. K. Hashmi, *Org. Chem. Front.* **2023**, *10*, 12-21.
36. Y. Shen, Z. Shang, Y. Yang, S. Zhu, X. Qian, P. Shi, J. Zheng, Y. Yang, *J. Org. Chem.* **2015**, *80*, 5906-5911.
37. A. Polley, K. Varalaxmi, A. Nandi, R. Jana, *J. Org. Chem.* **2021**, *10*, 1207-1215.
39. X. Zhang, E. L. Clennan, N. Arulsamy, *Org. Lett.* **2014**, *16*, 4610-4613.
40. S. Herzog, PhD thesis, Karlsruher Institut für Technologie (KIT), **2023**; doi: 10.5445/IR/1000164911.
41. J.-S. Bae, J.-E. Kim, H.-Y. Jang, J.-G. Kim, J.-G. Jang, S.-K. Hong, T.-Y. Park, D.-W. Lee (LG Chem, Ltd.), EP2332911B1, **2009**.
42. T. Deng, W. Yan, X. Liu, G. Hu, W. Xiao, S. Mao, J. Lin, Y. Jiao, Y. Jin, *Org. Lett.* **2022**, *24*, 1502-1506.
45. X. Zhang, F. Rauch, J. Niedens, R. B. da Silva, A. Friedrich, A. Nowak-Król, S. J. Garden, T. B. Marder, *J. Am. Chem. Soc.* **2022**, *144*, 22316-22324.
47. T. S. Reddy, S. Lee, M.-S. Choi, *Dyes Pigm.* **2019**, *168*, 49-58.
51. C. Goedicke, H. Stegemeyer, *Tetrahedron Lett.* **1970**, *11*, 937-940.
62. J. Ho, M. L. Coote, *Theor. Chem. Acc.* **2010**, *125*, 3-21.
63. A. Gero, J. J. Markham, *J. Org. Chem.* **1951**, *16*, 1835-1838.
64. M. Gaus, Q. Cui, M. Elstner, *J. Chem. Theory Comput.* **2011**, *7*, 931-948.
66. G. M. Sheldrick, *Acta Crystallogr., Sect. C: Struct. Chem.* **2015**, *71*, 3-8.
67. G. M. Sheldrick, SHELXL (Version 2014/7), **2013**.
68. O. V. Dolomanov, L. J. Bourhis, R. J. Gildea, J. A. K. Howard, H. Puschmann, *J. Appl. Crystallogr.* **2009**, *42*, 339-341.
69. L. Fra, A. Millán, J. A. Souto, K. Muñiz, *Angew. Chem. Int. Ed.* **2014**, *53*, 7349-7353.
70. J. Miguel-Ávila, M. Tomás-Gamasa, J. L. Mascareñas, *Angew. Chem. Int. Ed.* **2020**, *59*, 17628-17633.
71. M. J. Frisch, G. W. Trucks, H. B. Schlegel, G. E. Scuseria, M. A. Robb, J. R. Cheeseman, G. Scalmani, V. Barone, G. A. Petersson, H. Nakatsuji, X. Li, M. Caricato, A. V. Marenich,

J. Bloino, B. G. Janesko, R. Gomperts, B. Mennucci, H. P. Hratchian, J. V. Ortiz, A. F. Izmaylov, J. L. Sonnenberg, Williams, F. Ding, F. Lipparini, F. Egidi, J. Goings, B. Peng, A. Petrone, T. Henderson, D. Ranasinghe, V. G. Zakrzewski, J. Gao, N. Rega, G. Zheng, W. Liang, M. Hada, M. Ehara, K. Toyota, R. Fukuda, J. Hasegawa, M. Ishida, T. Nakajima, Y. Honda, O. Kitao, H. Nakai, T. Vreven, K. Throssell, J. A. Montgomery Jr., J. E. Peralta, F. Ogliaro, M. J. Bearpark, J. J. Heyd, E. N. Brothers, K. N. Kudin, V. N. Staroverov, T. A. Keith, R. Kobayashi, J. Normand, K. Raghavachari, A. P. Rendell, J. C. Burant, S. S. Iyengar, J. Tomasi, M. Cossi, J. M. Millam, M. Klene, C. Adamo, R. Cammi, J. W. Ochterski, R. L. Martin, K. Morokuma, O. Farkas, J. B. Foresman, D. J. Fox, Gaussian 16, Revision C.01, Gaussian, Inc., Wallingford, CT, **2016**.

72. J. P. Perdew, K. Burke, M. Ernzerhof, *Phys. Rev. Lett.* **1996**, *77*, 3865-3868; erratum: J. P. Perdew, K. Burke, M. Ernzerhof, *Phys. Rev. Lett.* **1997**, *78*, 1396.
73. C. Adamo, V. Barone, *J. Chem. Phys.* **1999**, *110*, 6158-6170.
74. F. Weigend, R. Ahlrichs, *Phys. Chem. Chem. Phys.* **2005**, *7*, 3297-3305.
75. F. Weigend, *Phys. Chem. Chem. Phys.* **2006**, *8*, 1057-1065.
76. S. Grimme, J. Antony, S. Ehrlich, H. Krieg, *J. Chem. Phys.* **2010**, *132*, 154104.
77. S. Grimme, S. Ehrlich, L. Goerigk, *J. Comp. Chem.* **2011**, *32*, 1456-1465.
78. A. Klamt, G. Schüürmann, *J. Chem. Soc., Perkin Trans. 2* **1993**, 799-805
79. V. Barone, M. Cossi, *J. Phys. Chem. A* **1998**, *102*, 1995-2001.
80. M. Cossi, N. Rega, G. Scalmani, V. Barone, *J. Comput. Chem.* **2003**, *24*, 669-681.
81. B. G. Johnson, M. J. Frisch, *Chem. Phys. Lett.* **1993**, *216*, 133-140.
82. B. G. Johnson, M. J. Fisch, *J. Chem. Phys.* **1994**, *100*, 7429-7442.
83. R. E. Stratmann, J. C. Burant, G. E. Scuseria, M. J. Frisch, *J. Chem. Phys.* **1997**, *106*, 10175-10183.
84. R. E. Stratmann, G. E. Scuseria, M. J. Frisch, *J. Chem. Phys.* **1998**, *109*, 8218-8224.
85. R. Bauernschmitt, R. Ahlrichs, *Chem. Phys. Lett.* **1996**, *256*, 454-464.
86. M. E. Casida, C. Jamorski, K. C. Casida, D. R. Salahub, *J. Chem. Phys.* **1998**, *108*, 4439-4449.
87. M. Page, J. W. McIver, Jr., *J. Chem. Phys.* **1988**, *88*, 922-935.
88. M. Page, C. Doubleday, J. W. McIver, Jr., *J. Chem. Phys.* **1990**, *93*, 5634-5642.
89. R. Dennington, T. Keith, J. Millam, GaussView (Version 6.1.1), Semichem Inc., Shawnee Mission, KS, **2019**.
90. C. F. Macrae, I. Sovago, S. J. Cottrell, P. T. A. Galek, P. McCabe, E. Pidcock, M. Platings, G. P. Shields, J. S. Stevens, M. Towler, P. A. Wood, *J. Appl. Crystallogr.* **2020**, *53*, 226-235.
91. N. M. O'Boyle, A. L. Tenderholt, K. M. Langner, *J. Comput. Chem.* **2008**, *29*, 839-845.
